# Iridium-Catalyzed Diorganosilylene Insertion into C(sp^2^)–O Bond of Oxacycles Using Aryldiorganosilanes as Silylene Transfer Reagents

**DOI:** 10.1021/acscentsci.6c00159

**Published:** 2026-05-05

**Authors:** Yunhao Song, Michinori Suginome

**Affiliations:** † Department of Synthetic Chemistry and Biological Chemistry, Graduate School of Engineering, 12918 Kyoto University, Nishikyo-ku, Kyoto 615-8510, Japan; ‡ Department of Chemical Science and Engineering, Graduate School of Engineering, 12918 Kyoto University, Nishikyo-ku, Kyoto 615-8510, Japan

## Abstract

Silylene transfer reactions, i.e., silylene cycloaddition and insertion reactions, allow single-step formation of two silicon-containing σ bonds. This has attracted much attention in synthetic organosilicon chemistry, whereas its development has been significantly hampered by the lack of synthetically viable, readily accessible silylene precursors. We herein report a new silylene insertion reaction using aryldiorganosilanes (ArR^1^R^2^SiH) as silylene transfer reagents, in which a diorganosilylene unit is inserted into the O–C­(sp^2^) bond of unsaturated oxacycles, such as benzofurans, enabled by iridium catalysts with extrusion of Ar–H as the only byproduct. A variety of silylenes, including diaryl-, (aryl)­(alkyl)-, dialkyl-, and even (aryl)­(alkoxy)­silylene units, are transferred to give ring-enlarged oxasilacycles. This protocol is extended to the germylene insertion reaction using Ph_3_GeH as a source of the diphenylgermylene group. The new silylene insertion is not limited to unsaturated oxacycles, including benzofurans and furans containing various functional groups, but is also applicable even to an acyclic enol ether, albeit in low yield. The complete silylene insertion process consists of two sequential silylene insertion processes, i.e., intermolecular silylene insertion into the C­(sp^2^)–H bond α to the oxygen atom, followed by intramolecular silylene insertion into the C–O bond. Formal silicon atom transfer is demonstrated using PhSiH_3_ as a source of the silicon atom, which in the first step undergoes cyclative double C–H silylation, followed by the present Ir-catalyzed silylene insertion to benzofuran. Using 5-(2-diphenyl­hydro­silyl­ethyl)­benzofuran as a monomer, silylene-inserting polymerization has been demonstrated.

## Graphical abstract

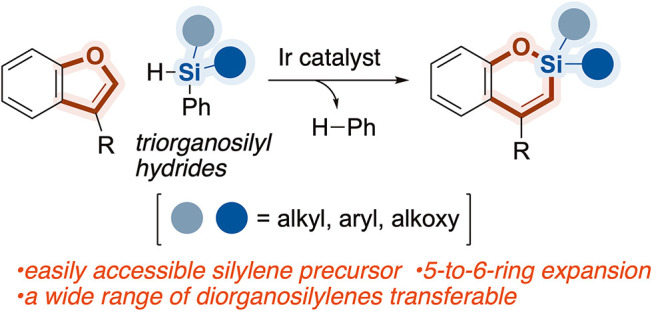

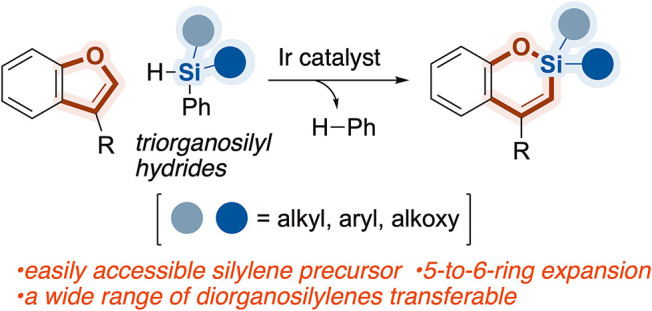

## Introduction

Silylene is a highly reactive, transient divalent silicon species, attracting much attention in organosilicon chemistry.^1,2^ Its unique reactivity is generally characterized by simultaneous formation of two silicon-containing σ bonds through cycloadditions with unsaturated organic reactants^3,4^ or insertions into σ bonds (Figure 1A, right),^5–9^ unlike other silylation reactions, e.g., hydrosilylation and C–H silylation, which result in the formation of a single silicon-containing σ bond. In association with the current emerging concept of skeletal editing,^10,11^ ring-expanding silylene insertion to readily available cyclic organic molecules is highly attractive as a new practical synthetic strategy for new cyclic organosilicon compounds,^12–15^ which are expected to play new roles in broad research areas, including pharmaceutical, agrochemical, and functional materials.^16–19^

**Figure 1:**
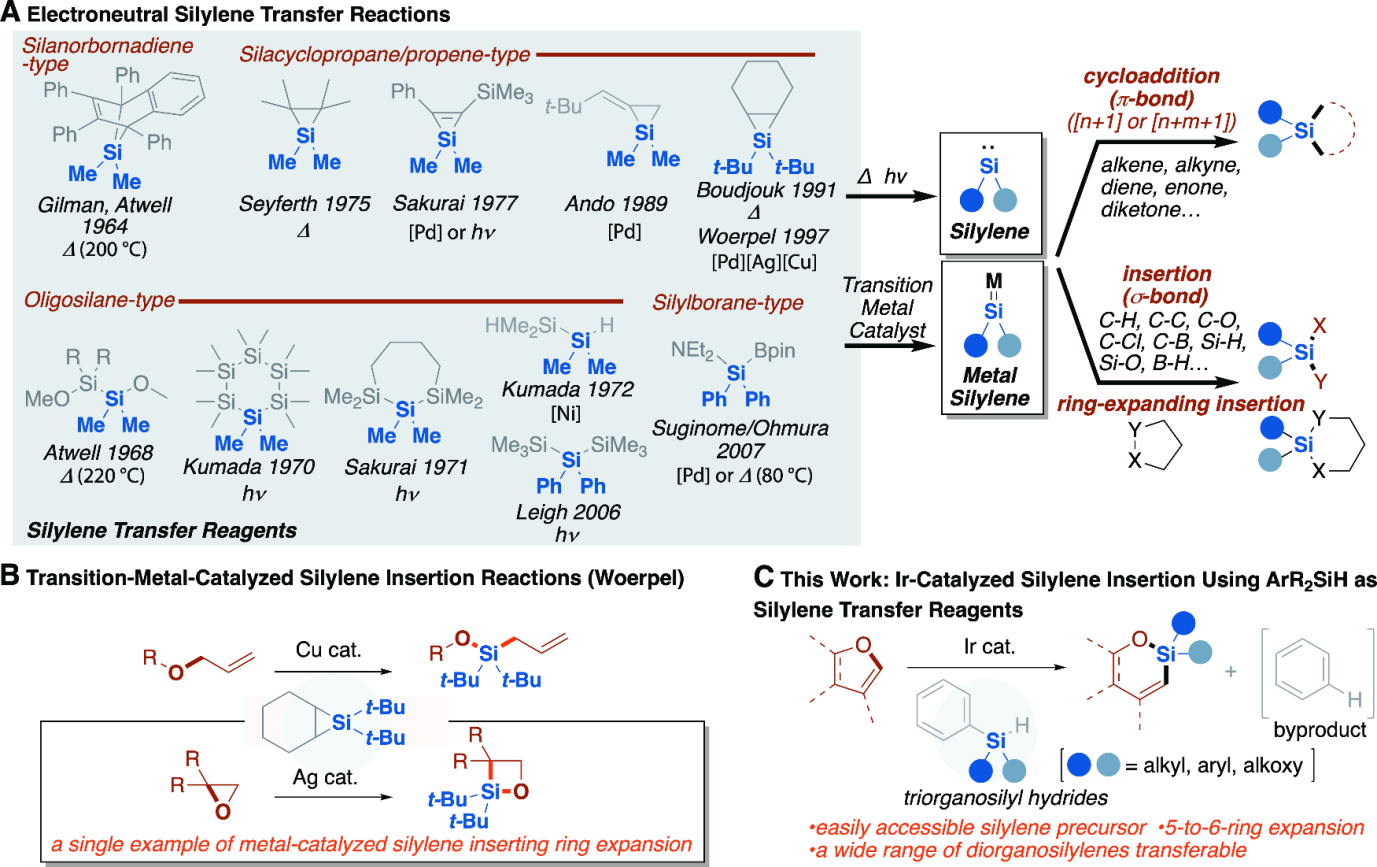
**Silylene transfer reactions.** (A) Silylene and metal silylene precursors in silylene transfer reactions. (B) Transition-metal-catalyzed silylene insertion reactions. (C) This work: iridium-catalyzed silylene insertion with furan derivatives using ArR_2_SiH as silylene transfer reagents.

Despite its potential, the utilization of silylene species in synthetic organosilicon chemistry has remained undeveloped. This can be ascribed to the lack of synthetically viable protocols for the generation of silylene species from readily accessible precursors.^20^ Although diorganosilylenes are generated from dihalodiorgaosilane derivatives under strongly reductive reaction conditions,^21^ the wide application of this protocol in silylene transfer reaction is difficult. To allow for the use of milder and redox-neutral reaction conditions, a variety of silylene precursors that enable thermal and photochemical silylene extrusion have been explored.^22–29^ However, those silylene precursors generally possess elaborated molecular structures such as 7-silanorbornadiene,^22^ silacyclopropane,^23–26^ and oligosilane structures,^27–29^ which are typically unstable or not readily accessible (Figure 1A, left).

To overcome the difficulties in generating free silylene species, the transition-metal-catalyzed silylene transfer reaction has attracted increasing interest (Figure 1A, left).^30–34^ In the early studies, hydrodisilanes were utilized as the source of silylene with loss of hydrosilanes in nickel- and platinum-catalyzed cycloaddition reactions.^30,31^ In the seminal studies by Woerpel’s group, silylene was transferred from silacyclopropanes in the presence of palladium, copper, and silver catalysts, leading to the development of a variety of cycloaddition reactions.^32–35^ It has been reported that only the transfer of di-*t*-butylsilylene was possible with the silacyclopropane-based silylene transfer reaction, thus diminishing synthetic viability, because of the instability of less sterically demanding silacyclopropanes.^36^ We separately reported silylene cycloaddition reactions using silylboranes bearing a dialkylamino group on the silicon atom as stable precursors for the transfer of dimethyl- and diphenylsilylene using palladium and rhodium catalysts.^37–41^ These demonstrations showed the wide scope of silylene transfer reaction in synthetic organosilicon chemistry, although the precursors, i.e., hydrodisilanes, silylboranes, and silacyclopropanes, are still not instantly accessible and are therefore not suitable for practical syntheses.^42^ It seems likely that the development of more readily accessible silylene transfer reagents expands the scope of the transition-metal-catalyzed silylene transfer reaction significantly, opening a new dimension of synthetic organosilicon chemistry.^20,43^ It should be noted that despite a wide variety of transition-metal-catalyzed cycloaddition reactions, the insertion reaction is limited to two reports by Woerpel’s group (Figure 1B).^33,34^

We herein report iridium-catalyzed ring-expanding silylene insertion into O–C­(sp^2^) bonds of cyclic vinyl ethers, including benzofurans and furans, which afford cyclic vinylsilanes containing O–Si–C­(sp^2^) linkages such as 2-silabenzopyrans and 2-silapyrans (Figure 1C). The most unusual feature of this reaction is the use of aryldiorganosilyl hydrides (ArR_2_SiH) as structurally simple, thermally and photochemically stable, readily accessible, and modifiable silylene precursors. From such simple precursors, iridium silylene intermediates are efficiently generated through α-elimination of Ar and H groups with formation of Ar–H as a single byproduct. Of note is that various types of silylene units can be transferred: diaryl, arylalkyl, dialkyl, and even arylalkoxysilylene are successfully accommodated.

## Results and Discussion

### Reaction Development

In the course of our study on the iridium-catalyzed reactions of benzofuran (**1a**) with 1 equiv of dimethyl­(*o*-tolyl)­silyl hydride (**A1**), we unexpectedly found the formation of 2-sila-2,2-dimethyl-2*H*-1-benzopyran (**2aA**) (Figure 2A), in which dimethylsilylene is inserted into the σ bond between the 1-oxygen and 2-carbon atoms of benzofuran with the formation of toluene as a byproduct. Our extensive optimization study revealed that **2aA** was formed in 90% yield in the presence of [IrCl­(cod)]_2_ (2 mol %) and DTBM-SEGPHOS (4 mol %) at 80 °C in toluene after 12 h. We observed a significant effect of the bidentate phosphine ligands: SEGPHOS-type diphosphine ligands bearing 3,5-dimethylphenyl (DM) and phenyl groups on the phosphorus atoms resulted in 38% and 14% yields of **2aA**, whereas BINAP, MeO-BIPHEP, and Garphos ligands bearing DTBM groups afforded 25%, 78%, and 59% yields, respectively. Without phosphine ligands, there was no formation of **2aA**, but disproportionation of **A1** was observed (see Tables S1–S4 for more detailed optimization results).

**Figure 2:**
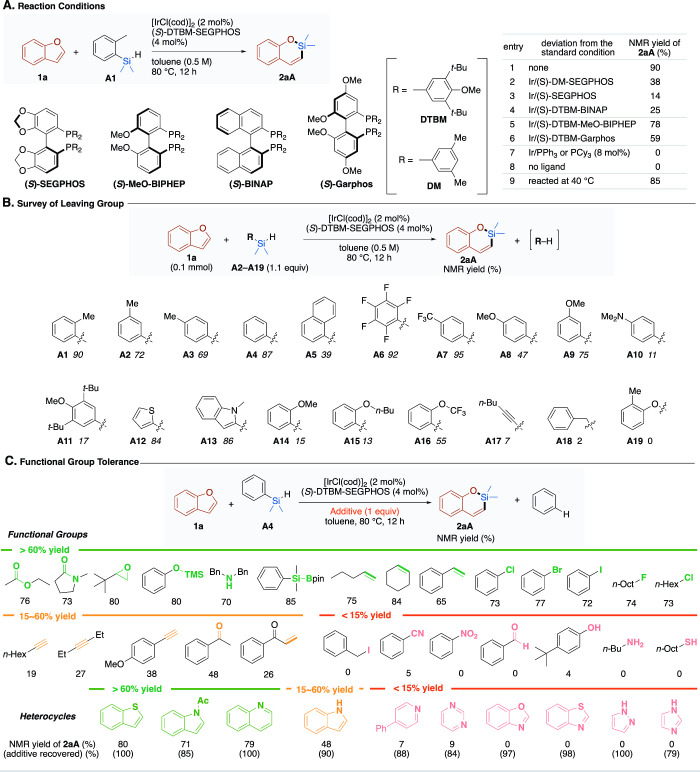
**Reaction development.** (A) Ligand screening with benzofuran (**1a**), dimethyl­(*o*-tolyl)­silane (**A1**), [IrCl­(cod)]_2_, and phosphine ligands (4 mol % P) at 80 °C for 12 h. (B) Screening of eliminating organic groups on hydrosilanes with **1a** (0.1 mmol), RMe_2_SiH **A1**–**A19** (1.1 equiv), [IrCl­(cod)]_2_ (2 mol %), and (*S*)-DTBM-SEGPHOS (4 mol %) in toluene at 80 °C for 12 h. (C) Functional group tolerance test under the standard conditions with 1 equiv of the additives. Yields of **2aA** are given with those of the recovered additives in parentheses.

Inspired by the unprecedented silylene-inserting ring expansion of benzofuran, in which the tolyl group and hydride are α-eliminated from the silicon atom, various organodimethylsilyl hydrides (RMe_2_SiH) **A1**–**A19** were subjected to the reaction with benzofuran under the optimized reaction conditions (Figure 2B). It was revealed that a wide array of aryl groups, including substituted phenyl groups and even heteroaryl groups, afforded product **2aA** in high yields. Some aryldimethylsilyl hydrides containing electron-rich (**A8**, **A10**, **A11**) or sterically demanding (**A5**, **A11**, **A14**–**A16**) aryl groups gave poorer yields of **2aA**. Silyl hydrides bearing heteroaryl groups such as 2-thienyl (**A12**) and *N*-methylindol-2-yl (**A13**) afforded **2aA** in high yields. By contrast, alkynyl (**A17**), benzyl (**A18**), and aryloxy (**A19**) groups were found to be poor leaving groups in this reaction. It should be remarked that the formation of α-eliminated Ar–H was generally observed, as exemplified by the quantification of benzene in 57% NMR yield in the reaction of **A4** (see Figure S1). It looked that nonbulky, electron-neutral or -poor aryl groups were the leaving groups of choice. In consideration of both elimination ability and synthetic accessibility, we chose the phenyl group as the standard leaving group on the silicon atom in the following examinations.

The additive-based functional group tolerance test was carried out (Figure 2C).^44^ The iridium-catalyzed silylene insertion was compatible with a variety of functional groups, as exemplified by high yield formation of **2aA** in the presence of ester, amide, epoxide, phenolic silyl ether, secondary amine, and silylborane additives. Of note was the compatibility with terminal, internal, and styrenic C=C bonds, which are susceptible not only to cycloaddition with free silylene but also to hydrosilylation. Furthermore, various aryl (X: Cl, Br, I) and alkyl (X = F and Cl) halides were tolerant to the reaction, although benzylic iodide was not compatible. Alkynes and ketones, including an enone, were compatible, even though the yields of **2aA** were low to moderate (19–48%). By contrast, the presence of benzonitrile, nitrobenzene, benzaldehyde, phenols, butylamine, and octanethiol strongly inhibited the formation of **2aA** (<5%). Compatibility of the silylene insertion with various heterocycles were also examined by determining both the yields of silylene insertion products **2aA** and the recovered heterocyclic additives. Thiophene, quinoline, and indole derivatives gave essentially no or only a little effect on the reaction, while unprotected indole showed slight inhibition of the silylene insertion. Pyridine, pyrimidine, pyrazole, imidazole, benzoxazole, and benzothiazole derivatives, all of which contain sp^2^ nitrogen atoms, showed significant deactivation of the catalyst, resulting in essentially no reaction.

### Scope of the Reaction

Various diorganophenylsilyl hydrides (PhR_2_SiH) were subjected to the reactions with benzofuran (Figure 3A). Dialkylsilylenes, including methylcyclohexyl and butyl-*t*-butylsilylene, were successfully transferred from the corresponding aryldialkylsilyl hydrides (**2aB**–**2aD**). Upon using diphenylalkylsilyl hydrides, one of the phenyl groups is α-eliminated with hydride to lead to the transfer of arylalkylsilylenes (**2aE**–**2aH**). Their insertion proceeded enantioselectively depending on the bulkiness of the alkyl substituents: the product **2aG** formed through insertion of bulky *t*-butylphenylsilylene showed 82% ee, whereas transfer of isopropylphenylsilylene proceeded with 14% ee (**2aF**). Of note is that reactions of diphenyl­(alkoxy)­silyl hydrides led to the selective insertion of phenyl­(alkoxy)­silylene through α-elimination of Ph–H, giving **2aI**. Even though the alkoxy group generally serves as a more efficient leaving group in a variety of reactions, the phenyl group undergoes α-elimination selectively in this reaction. Use of Ph_3_SiH **J** as a source of silylene gave the product **2aJ** via insertion of diphenylsilylene in good yield. Interestingly, triarylsilyl hydrides **K** and **L**, in which the silicon atom is involved as a part of the silacyclic rings, underwent the silylene insertion with retention of the silafluorene (**2aK**) and tribenzosilepin (**2aL**) structures through selective elimination of the phenyl group. Reaction of Ph_2_(DTBM)­SiH proceeded through selective elimination of the less sterically demanding phenyl group rather than the bulky DTBM group, giving **2aM** selectively in 67% yield.

**Figure 3:**
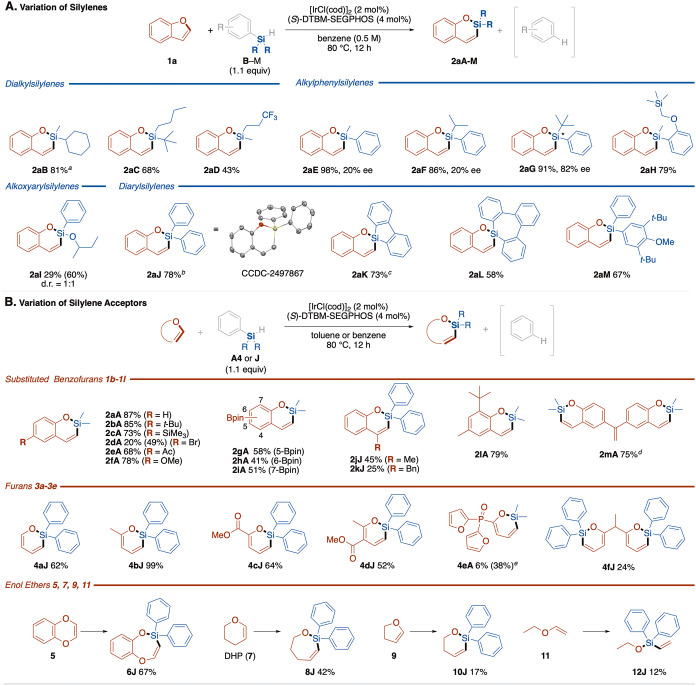
**Substrate scope of (A) silylenes and (B) silylene acceptors.** Isolated yields are shown, with NMR yields in parentheses. Reaction conditions: furan derivatives **1**–**11** (0.2 mmol), hydrosilanes **A**–**M** (0.22 mmol, 1.1 equiv), [IrCl­(cod)]_2_ (2 mol %), and (*S*)-DTBM-SEGPHOS (4 mol %) in benzene or toluene (400 μL, 0.5 M) at 80 °C for 12 h. Notes: *^a^* *m*-Methoxyphenylmethylcyclohexylsilane (**B9**) as a silylene source. *^b^* **1a** (140 mg, 1.2 mmol) and **J4** (318 mg, 1.23 mmol). *^c^* 1.7 equiv of **1a**. *^d^* 3.6 equiv of **A4**. *^e^* 3 equiv of **A4** in benzene/AcOEt (2:1 v/v, 600 μL) at 110 °C for 12 h.

The scope of silylene-accepting substrates was then screened (Figure 3B). Benzofurans having various functional groups such as *t-*Bu, SiMe_3_, Br, Ac, OMe, and Bpin in the six-membered ring afforded the corresponding products **2bA**–**2iA** in good yields. The reaction of 3-methylbenzofurans proceeded sluggishly to provide the corresponding product **2jJ** in 45% yield, whereas 3-benzylbenzofuran gave **2 kJ** only in low yield. The observation that no reaction took place with 2-methylbenzofuran may be relevant to the mechanism of the reaction as discussed below (see Figure S2). Double silylene insertion was achieved in good yield, affording **2mA** by reacting benzofuran **1m** and an excess amount of hydrosilane **A4**.

The silylene insertion is not limited to benzofuran derivatives but extended to other cyclic structures containing O–C­(sp^2^) bonds (Figure 3B). Various furans **3a**–**3f**, including unsubstituted, 2-methyl-, and methoxycarbonyl-substituted furans, underwent the silylene insertion in moderate to good yields, giving the corresponding 2-silapyrans **4**. In the reaction of trifurylphosphine oxide, a product **4eA** derived from the insertion of dimethylsilylene into one of the three furyl rings was formed, albeit in moderate yield. Moreover, an unprecedented silicon-inserting six- to seven-membered ring expansion was achieved in the reactions of six-membered cyclic enol ethers, i.e., 1,4-benzodioxin (**5**) and 3,4-dihydro-2*H*-pyran (**7**), affording **6J** and **8J**, respectively.^45^ The attempted reaction of 2,3-dihydrofuran (**9**) resulted in low-yield formation of silylene insertion product **10J**. Of note is that the linear vinyl ether **11** also reacted at the vinylic C­(sp^2^)–O bond, giving diphenylsilylene-inserted product **12J**, indicating ring structure is not necessary to the silylene acceptors.

### Synthetic Applications

We further demonstrated silylene-insertion-based spiro-ring-forming coupling of the highly functionalized benzofuran derivative khellin (**2m**), which is known as an herbal folk medicine, with the 4-silapiperidine ring, which is contained in a silicon-based drug (Figure 4A).^46^ The silylene precursor **N**, which carries phenyl and hydride groups on the silicon atom, was quickly prepared by double hydrosilylation of *N*,*N*-divinyl-*p*-toluenesulfonylamide with PhSiH_3_.^47^ Its reaction with **2m** afforded highly functionalized silaspirocyclic **2mN** in 36% yield. Furthermore, formal “silicon atom transfer”, in which only the silicon atom of PhSiH_3_ is transferred to the final product, was demonstrated by harnessing the present silylene insertion with cyclizative double C–H silylation reported by S. Chang (Figure 4B).^48^ In the initial step, *N*-phenylpiperidine was reacted with PhSiH_3_ in the presence of B­(C_6_F_5_)_3_ catalyst to obtain tricyclic **P**, which has Ph and hydride groups on the silicon atom. Reaction of **P** with benzofuran under the standard reaction conditions for silylene insertion resulted in the formation of polycyclic **2aP** in high yield, in which the silicon atom is located at the spiro junction. Through two steps, only the silicon atom of PhSiH_3_ is retained in the product, being regarded as a formal silicon atom transfer using PhSiH_3_ as a source of the silicon atom. We also found that the reaction was extended to germylene insertion: reaction of benzofuran with Ph_3_GeH under the identical reaction conditions provided 2-germabenzopyran in high yield (Figure 4C). The unique features of the present silylene insertion including use of stable and readily accessible silylene precursors enables silylene-inserting polymerization of AB-type monomer **14**, which has a silylene-extruding diphenylhydrosilane group and a silylene-accepting benzofuran moiety connected by an alkyl chain (Figure 4D). **14** was stable and purifiable, successfully reacting under the standard conditions, giving soluble polymer **15** in 73% yield (*M*_w_/*M*_n_ = 2.1). The time course of the polymerization was followed using analytical GPC, and it was observed that the quickly formed oligomers were gradually linked together, affording monodispersed **15**, which was isolated by reprecipitation from CHCl_3_/hexane.

**Figure 4:**
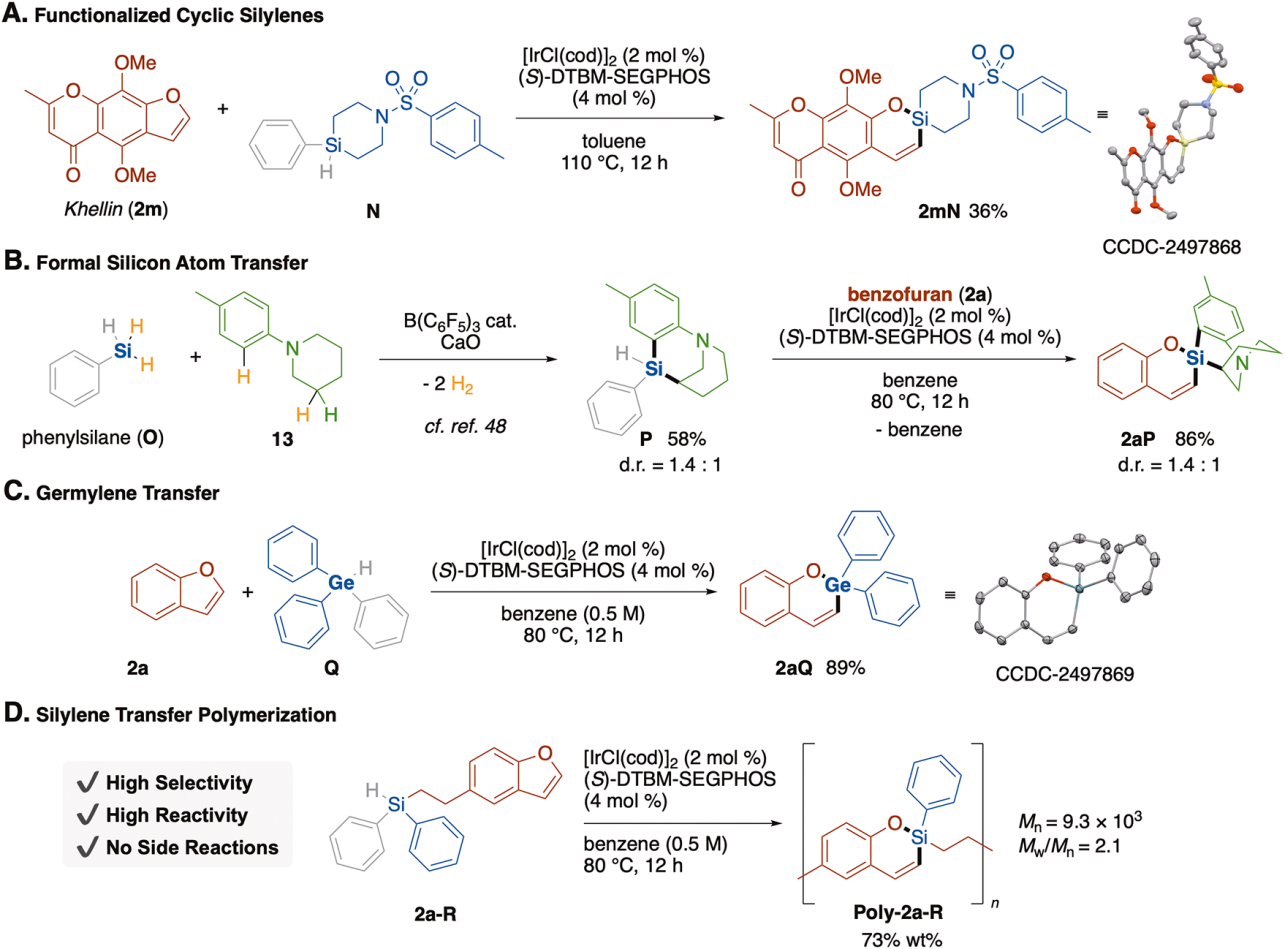
**Synthetic applications.** (A) Application to a natural medicine molecule. (B) Two-step formal silicon atom transfer reaction. (C) Germylene insertion reaction. (D) Silylene-inserting polymerization.

### Reaction Mechanism

We propose the mechanism of the present ring-expanding silylene insertion as shown in Figure 5A. It consists of two σ-bond activation steps, i.e., C–H activation at the 2-position of the furan ring and ring-expanding C­(sp^2^)–O bond activation. It should be remarked that the Ir­(I) catalyst, especially when it exists as a monomeric state, is highly active for oxidative addition of both C–H and Si–H bonds as demonstrated by NMR measurements (see Figures S12–S17). It should also be noted that in the absence of benzofuran, Ph_3_SiH underwent exchange of the Ph group with aromatic solvents: in *o*-xylene, mono-, bis-, and tris­(3,4-xylyl)-substituted triarylhydrosilanes were obtained in 5%, 23%, and 37% yield (Figure 5C­(a)). This aryl exchange reaction can be regarded as silylene transfer to the solvent C–H bond, indicating that the silyliridium hydride species **A** generated through oxidative addition of the Si–H bond further inserts into an aromatic C–H bond, leading to the silylene transfer. It is therefore presumed that furan, when it exists, undergoes selective activation at its 2-C–H bond, which is most susceptible to activation, resulting in the formation of (silyl)­(furyl)­iridium dihydride **B**.^49^ The involvement of the activation of the benzofuran 2-C–H bond is also supported by the attempted reactions of 2-substituted benzofurans, which resulted in no reaction (see Figure S2). Thus generated **B** undergoes exchange of the phenyl group on the silicon atom and the furyl group on the iridium atom, leading to the reductive elimination of Ph–H (benzene) with formation of (furyl)­dimethylsilyliridium hydride **C**. The furyl group in **C** migrates back to the iridium atom to form dimethylsilylene intermediate **D**,^50^ whose formation is favored by the coordination of the furan oxygen to the silicon atom. Subsequent C–O/Ir=Si metathesis affords seven-membered cyclic vinylideneiridium intermediate **E**, which undergoes 1,2-silyl migration followed by reductive elimination of the C–H bond, giving ring-expanded product **2**.

**Figure 5:**
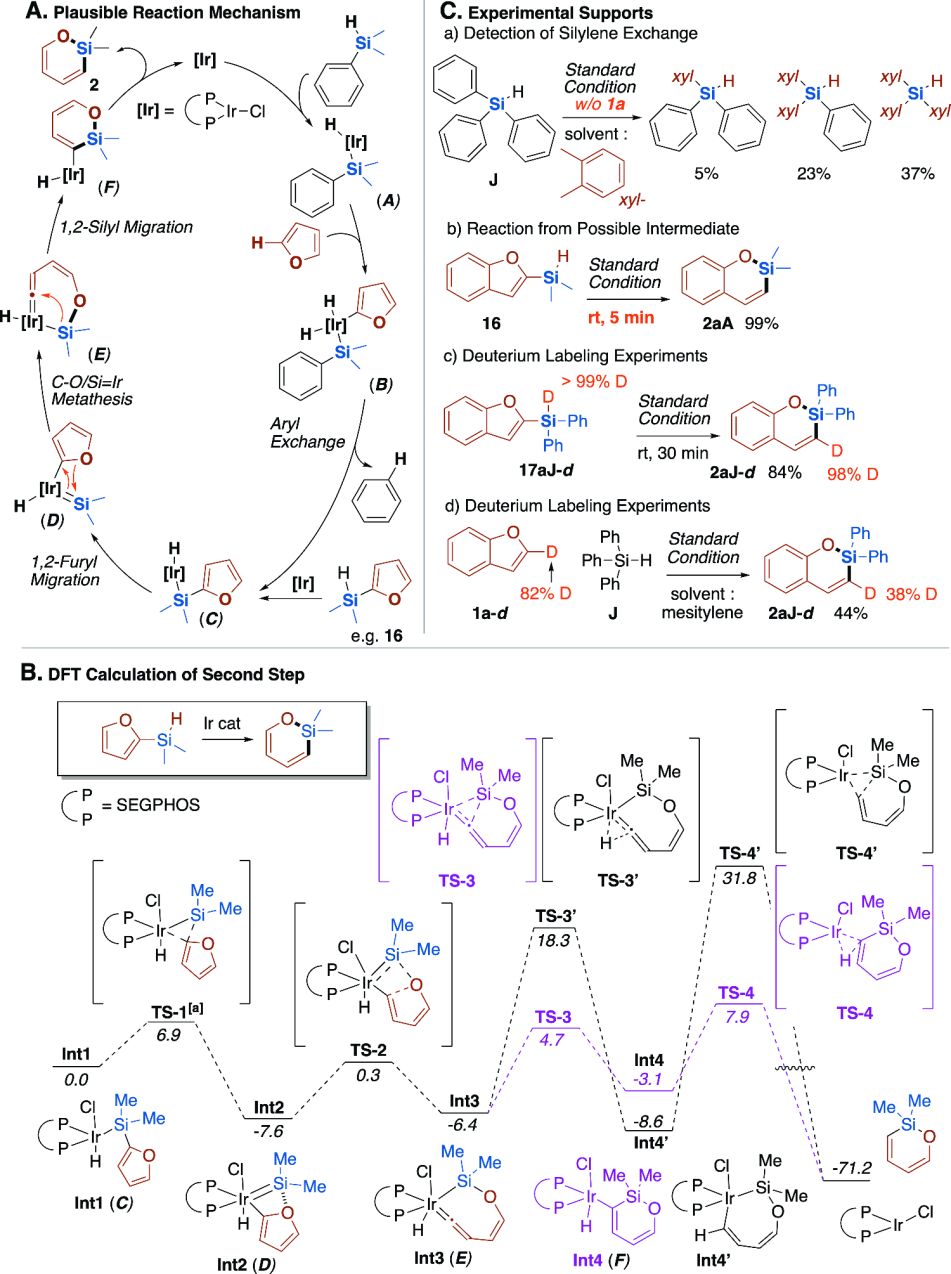
**Mechanistic investigation.** (A) Plausible reaction mechanism. (B) DFT calculation of the second step. Calculations were performed at the B3LYP-D3/6-311++G­(d,p)-SDD­(Ir)/SMD­(toluene)//B3LYP/6-31G­(d)-LANL2DZ­(Ir) level of theory. (C) Experimental mechanistic studies.

Although we could not find a feasible reaction pathway for the initial C–H activation step leading to **C** using a density functional theory (DFT) calculation, the following ring-expanding step (**C** to product **2**) could be established (Figure 5B). In the following product-forming events, 1,2-migration of the silicon atom (through **TS-3**) proceeds much faster than the 1,2-hydrogen migration (**TS-3′**).^51^

This reaction mechanism is also supported by some experiments (Figure 5C). First, separately prepared (2-benzofuryl)­dimethylsilane **16**, which is likely to undergo facile oxidative addition to give intermediate **C** directly, afforded the ring-expanding product **2aA** *at room temperature* within 5 min (Figure 5C­(b)). The skeletal rearrangement of **16** to **2aA** strongly support the intermediacy of **C**, even though **16** was never detected in the reaction mixture by the NMR monitoring of the reaction. Furthermore, perfect deuterium incorporation was observed in the reaction of deuterated **17aJ-** ***d*** affording 3-deuterated **2aJ-** ***d*** (Figure 5C­(c)). These results are consistent with the DFT calculation, in which almost all transformations proceed with a small barrier, leading to the relocation of the H atom from the silicon atom to the carbon atom through intermediate **C**. These results also strongly support the involvement of intermediate **C**, whose formation is assumed to be rate-determining. It should be noted that, even though Ru-catalyzed ring-expanding rearrangement of **16** was recently reported,^14^ the proposed mechanism does not involve a silylruthenium intermediate like **C** but proceeds through regioselective Ru–H addition to the furan 2,3-C=C bond. Although deuterium labeling experiments of the whole STR reaction at an elevated temperature were not feasible because of the rapid H/D scrambling even with the solvents, ligands on precatalyst, and substrates, the selective formation of 3-deuterio-**2aA** from 2-deuteriobenzofuran showed good agreement with the proposed mechanism (Figure 5C­(d)).

## Conclusion

We have established a new iridium-catalyzed insertion of silylene using aryldiorganosilyl hydrides (ArR_2_SiH) as readily available, stable silylene precursors, which allow transfer of various silylenes, including dialkyl-, diaryl-, (aryl)­(alkyl)-, and (aryl)­(alkoxy)­silylenes with elimination of Ar–H as easily removable byproducts. In addition to various benzofuran derivatives, various C­(sp^2^)–O bonds in the cyclic and even acyclic vinyl ethers undergo the silylene insertion, leading to the formation of silyl enol ethers including oxasilacycles. The new reaction was extended to formal silicon atom transfer using PhSiH_3_ as the source of silicon atom and to germylene insertion using Ph_3_GeH as a source of diphenylgermylene. The reaction consists of two silylene transfer processes: the initial intermolecular silylene insertion into the 2-C­(sp^2^)–H bond of the furan ring is followed by fast intramolecular silylene migration into the C­(sp^2^)–O bond with ring expansion. We demonstrated unprecedented silylene-inserting polymerization using 5-(2-diphenylsilylethyl)­benzofuran as an AB-type monomer. The present silylene insertion is synthetically valuable not only because the reaction provides new efficient access to silacyclic compounds but also because it may serve as a new way of harnessing two functional motifs in an orthogonal manner, such as click reactions. With this reaction, a new chemical space of silicon-containing heterocycles will be significantly expanded.

## Supplementary Material



## Notes

Experimental procedures and spectral data for the new compounds (PDF)
