# Supplementary material for: Iridium-Catalyzed Diorganosilylene Insertion into C(sp2)–O Bond of Oxacycles Using Aryldiorganosilanes as Silylene Transfer Reagents
Source: ACS Cent Sci. 2026 May 5;12(5):649–57. doi: 10.1021/acscentsci.6c00159 (PMC13220184; doi:10.1021/acscentsci.6c00159)
Supplement: Supplementary file 1 [file oc6c00159_si_001.pdf]

Supplementary Materials for

Iridium-Catalyzed Diorganosilylene Insertion into C(sp^2)–O Bond of Oxacycles Using Aryldiorganosilanes as Silylene Transfer Reagents

Yunhao Song[1] and Michinori Suginome*[1,2]

[1] *Department of Synthetic Chemistry and Biological Chemistry, Graduate School of Engineering, Kyoto University, Nishikyo-ku, Kyoto 615-8510, Japan.*

[2] *Department of Synthetic Chemistry and Biological Chemistry, Graduate School of Engineering, Kyoto University, Nishikyo-ku, Kyoto 615-8510, Japan.*

Contents

1. General S2
2. Materials S2
3. Preparation of Substrates S3
4. Reaction Conditions S11
5. Synthetic Applications S33
6. Mechanistic Investigations S41
7. DFT Calculation S52
8. X-ray Crystallographic Analyses S67
9. References S71
10. ^{1}H and ^{13}C NMR Spectra of New Compounds S73

1. General

All iridium-catalyzed reactions were performed in glove box under an atmosphere of nitrogen with magnetic stirring. Other reactions were also carried out under an atmosphere of nitrogen with magnetic stirring unless otherwise noted. Materials were weighted by an electric balance, Sartorius CPA225D or Shimadzu AP225WD (readability: 0.01 mg). Column chromatography was performed with SiliaFlash (SILICYCLE, pH 7.0, 40-63 μm, 60 Å) or Chromatolex DIOL MB100-40/75 (Fuji Silysia Chemical, 60 μm). Latter was used mainly for purification of 2-silapyranes. Supercritical fluid chromatography (SFC) was performed by Jasco Analytical SFC system (PU-4380, PU-4185, CO-4065, AS-4350, UV-4075, CD-4095, and BP4340). 1H NMR spectra were recorded on JEOL JNM-ECZ400S (399.89 MHz) or JEOL JNM-ECZ600R (600.17 MHz) spectrometers. 13C NMR spectra were recorded on JEOL JNM-ECZ400S (100.55 MHz) or JEOL JNM-ECZ600R (150.91 MHz) spectrometers. 31P NMR spectra were recorded on JEOL JNM-ECZ400S (161.87 MHz) spectrometers. 29Si NMR spectra were recorded on JEOL JNM-ECZ400S (79.44 MHz) or JEOL JNM-ECZ600R (119.24 MHz) spectrometers. For ^{1}H NMR spectra, chem77ical shifts (δ) were reported relative to residual $CHCl_3$ (d 7.26 ppm) in $CDCl_3$ or C_6H_6 (d 7.16 ppm) in C_6D_6. For ^{13}C NMR spectra, chemical shifts (δ) were reported relative to $CDCl_3$ (d 77.16 ppm) or C_6D_6 (d 128.06 ppm). For ^{31}P NMR spectra, chemical shifts (δ) were reported ppm downfield from H3PO4 (85%). For ^{29}Si NMR spectra, chemical shifts (δ) were reported ppm downfield from tetramethylsilane (TMS). Following abbreviations were used for multiplicity: s = singlet, d = doublet, t = triplet, q = quartet, and m = multiplet. High resolution mass spectra (HRMS) were recorded on JEOL JMS-MS700 (EI), Thermo Scientific Exactive Plus (APCI, ESI) or Bruker Daltonics ultrafleXtreme (MALDI-TOF) spectrometers. The GPC analysis was carried out with TSKgel SuperMultipore HZ-M (two columns were connected in a series, THF containing 0.1 wt% triethylamine, polystyrene standards). Preparative GPC (Gel Permeation Chromatography) was performed on JAI LaboACE LC-5060 equipped with JAIGEL-1HR and -2HR columns in a series (CHCl3) and JAI LC-9225NEXT equipped with JAIGEL 1H-40 and 2H-40 in a series (CHCl3).

2. Materials

Toluene (dehydrated Super plus, Kanto), tetrahydrofuran (THF, dehydrated Super plus, Kanto, or Super dehydrated, stabilizer free, Wako) ethyl acetate (AcOEt, Super dehydrated, stabilizer free, Wako), benzene (Super dehydrated, stabilizer free, Wako), cyclohexane (Super dehydrated, stabilizer free, Wako) methyl tertiarybutylether (Super dehydrated, stabilizer free, Wako), 1,4-dioxane (Super dehydrated, stabilizer free, Wako), 1,2-dichlotoethane (Super dehydrated, stabilizer free, Wako), 1-chlorohexane (Super dehydrated, stabilizer free, Wako), acetonitrile (Super dehydrated, stabilizer free, Wako), dimethylformamide (Super dehydrated, stabilizer free, Wako) and 1,4-dioxane-d_8 (Cambridge Isotope Laboratories) were purchased and used as recieved.

$[IrCl(cod)]_2$,$[Ir(cod)_2](BF_4)$ and $[RhCl(cod)]_2$, were each prepared according to the procedure reported previously[1–3] and stored at room temperature under nitrogen atmosphere. $[Ir(cod)(OMe)]_2$ and $[Cu(OAc)_2]$ were purchased from TCI and Wako, used as received. [(S)-DTBM-SEGPHOS, TCI], [(S)-DM-SEGPHOS, TCI], [(S)-SEGPHOS, TCI], [(S)-DTBM-BINAP, TCI], [(S)-DTBM-MeO-BIPHEP, Aldrich], [(S)-DTBM-Garphos, Aldrich], [PPh_3, TCI], [PCy_3, TCI], [dtbpy, Aldrich] and [teteramethylphenatrolene, Aldrich] were used as received from commercial source. (±)-DTBM-SEGPHOS, which was used for synthesis of racemic compound, was prepared according to the reported procedures.[4–6]

3. Preparation of New Substrates

3.1. Synthesis of Hydrosilanes

(3,5-di-*tert*-butyl-4-methoxyphenyl)dimethylsilane (A11)

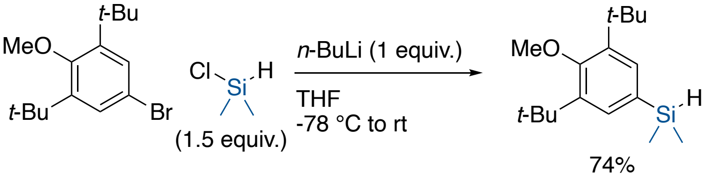

An oven dried 200 mL three-neck flask, equipped with a magnetic stirring bar and rubber septum was evacuated and backfilled with nitrogen. 5-bromo-1,3-di-*tert*-butyl-2-methoxybenzene [(synthesized by methylation of 4-bromo-2,6-di-*tert*-butylphenol, Aldrich), 3.2g, 10.8 mmol] and THF (20 mL) were charged to the flask and cooled to -78 °C. *n*-BuLi (nacalai, 1.6 M in hexane, 8 mL, 1 equiv.) was slowly added to the flask under -78 °C, then the mixture was stirred at -78 °C for 0.5 h. Chlorodimethylsilane (Aldrich, 1.64 g, 17.4 mmol, 1.5 equiv.) was added to the flask dropwise, and the mixture was stirred at room temperature, overnight. Water was added to the flask. The organic products were extracted by Et_2O, washed with Brine, and dried over Na_2SO_4. Solvent was removed under reduced pressure. The resulting crude materials were purified by column chromatography on silica gel (SiliaFlash; eluent: gradient from hexane only to hexane : Et_2O = 95 : 5) and kügelrhor distillation. (3,5-di-*tert*-butyl-4-methoxyphenyl)dimethylsilane (**A11**) (2.22 g, 7.98 mmol, 74% yield) was obtained as a white solid.

^{1}H NMR (400 MHz, $CDCl_3$) δ 7.42 (s, 2H), 4.43-4.38 (m, 1H), 3.71 (s, 3H), 1.45 (s, 18H), 0.34 (d, J = 3.7 Hz, 6H).

^{13}C NMR (101 MHz, $CDCl_3$) δ 160.8, 143.0, 132.4, 130.6, 64.2, 35.8, 32.2, -3.4.

HRMS (EI) m/z: [M]+ Calcd. for $C_{17}H_{30}OSi$, 278.2066 ; Found, 278.2062.

(2-butoxyphenyl)dimethylsilane (A15)

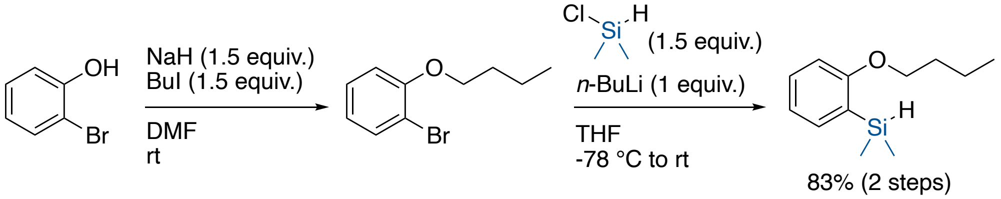

An oven dried 300 mL three-neck flask, equipped with a magnetic stirring bar and rubber septum was charged NaH (Nacalai, 860 mg, 14 mmol, 1.5 equiv.) then evacuated and backfilled with nitrogen. 2-Bromophenol (TCI, 1.96 g, 11.4 mmol) and DMF (20 mL) were charged to the flask at 0 °C. After the H_2 generation finished, butyliodide (TCI, 2.89 g, 15.8 mmol, 1.5 equiv.) was slowly added to the flask under 0 °C, then the mixture was allowed to stir at room temperature overnight. Water was added to the flask. The organic products were extracted by Et_2O, washed with Brine, and dried over Na_2SO_4. Solvent was removed under reduced pressure. The resulting crude materials were purified by column chromatography on silica gel (SiliaFlash; eluent: hexane: Et_2O = 90: 10). All the resulting material was used to the next step after removing solvent under reduced pressure.

An oven dried 200 mL three-neck flask, equipped with a magnetic stirring bar and rubber septum was evacuated and backfilled with nitrogen. 1-bromo-2-butoxybenzene and THF (20 mL) were charged to the flask and cooled to -78 °C. *n*-BuLi (nacalai, 1.6 M in hexane, 7 mL, 1 equiv.) was slowly added to the flask under -78 °C, then the mixture was stirred at -78 °C for 0.5 h. Chlorodimethylsilane (Aldrich, 1.41 g, 15.0 mmol, 1.5 equiv.) was added to the flask dropwise, and the mixture was stirred at room temperature, overnight. Water was added to the flask. The organic products were extracted by Et_2O, washed with Brine, and dried over Na_2SO_4.

Solvent was removed under reduced pressure. The resulting crude materials were purified by column chromatography on silica gel (SiliaFlash; eluent: hexane: Et_2O = 95: 5) and kügelrhor distillation. (2-butoxyphenyl)dimethylsilane (**A15**) (1.96 g, 9.43 mmol, 83% yield) was obtained as a colorless oil.

^{1}H NMR (400 MHz, $CDCl_3$) δ 7.44 (dd, J = 6.9, 1.8 Hz, 1H), 7.35 (ddd, J = 8.7, 6.9, 1.4 Hz, 1H), 6.94 (td, J = 7.3, 0.9 Hz, 1H), 6.82 (d, J = 8.2 Hz, 1H), 4.44-4.38 (m, 1H), 3.97 (t, J = 6.4 Hz, 2H), 1.83-1.76 (m, 2H), 1.58-1.49 (m, 2H), 0.99 (t, J = 7.3 Hz, 3H), 0.35 (d, J = 3.7 Hz, 6H).

^{13}C NMR (101 MHz, $CDCl_3$) δ 163.8, 135.9, 131.2, 125.5, 120.3, 110.0, 67.3, 31.5, 19.5, 14.0, -3.6.

HRMS (EI) m/z: [M]+ Calcd. for $C_{12}H_{20}OSi$, 208.1283; Found, 208.1281.

(2-(trifluoromethoxy)phenyl)dimethylsilane (A16)

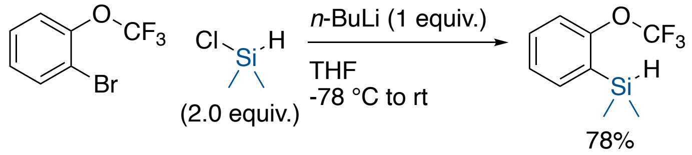

An oven dried 200 mL three-neck flask, equipped with a magnetic stirring bar and rubber septum was evacuated and backfilled with nitrogen. 1-bromo-2-(trifluoromethoxy)benzene (TCI, 1.40 g, 5.8 mmol) and THF (10 mL) were charged to the flask and cooled to -78 °C. *n*-BuLi (nacalai, 1.6 M in hexane, 4 mL, 1 equiv.) was slowly added to the flask under -78 °C, then the mixture was stirred at -78 °C for 0.5 h. Chlorodimethylsilane (Aldrich, 1.09 g, 11.6 mmol, 2 equiv.) was added to the flask dropwise, and the mixture was stirred at room temperature, overnight. Water was added to the flask. The organic products were extracted by Et_2O, washed with Brine, and dried over Na_2SO_4. Solvent was removed under reduced pressure. The resulting crude materials were purified by column chromatography on silica gel (SiliaFlash; eluent: hexane: Et_2O = 95: 5) and preparative GPC. Dimethyl(2-(trifluoromethoxy)phenyl)-silane (**A16**) (1.00 g, 4.55 mmol, 78% yield) was obtained as a colorless oil.

^{1}H NMR (400 MHz, $CDCl_3$) δ 7.54 (dd, J = 7.3, 1.8 Hz, 1H), 7.42 (td, J = 7.8, 1.7 Hz, 1H), 7.28-7.23 (m, 2H), 4.51-4.45 (m, 1H), 0.38 (d, J = 3.7 Hz, 6H).

^{13}C NMR (101 MHz, $CDCl_3$) δ 154.4, 136.3, 131.3, 129.8, 126.2, 120.7 (q, J_{CF} = 258 Hz, CF_3), 118.5, -3.8.

HRMS (EI) m/z: [M]+ Calcd. for $C_9H_{11}OF_3Si$, 220.0531; Found, 220.0526.

(*o*-tolyloxy)dimethylsilane (A19)

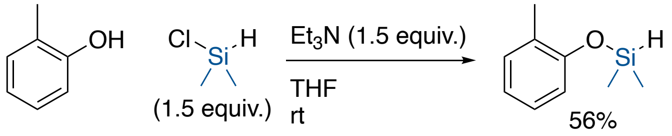

An oven dried 200 mL three-neck flask, equipped with a magnetic stirring bar and rubber septum was evacuated and backfilled with argon. *o*-cresol (TCI, 1.29 g, 11.9 mmol), Chlorodimethylsilane (Aldrich, 1.66 g, 17.7 mmol, 1.5 equiv.), Et_3N (Nacalai, 1.45 g, 14.3 mmol, 1.5 equiv.) and THF (20 mL) were charged to the flask and stirred at room temperature, overnight. THF was removed under reduced pressure and insoluble solid was filtered by oven dried celite. All the operation were performed under argon atmosphere. Resulting crude solution was further purified by kügelrhor distillation. (*o*-tolyloxy)dimethylsilane (**A19**) (1.10 g, 6.6 mmol, 56% yield) was obtained as a colorless oil. **A19** is highly unstable to moisture.

^{1}H NMR (400 MHz, $CDCl_3$) δ 7.16-7.14 (m, 1H), 7.11-7.06 (m, 1H), 6.89 (td, J = 7.4, 1.2 Hz, 1H), 6.84 (dd, J = 8.0, 1.1 Hz, 1H), 4.97-4.93 (m, 1H), 2.21 (s, 3H), 0.36 (d, J = 2.7 Hz, 6H).

^{13}C NMR (101 MHz, $CDCl_3$) δ 153.8, 131.0, 128.9, 126.8, 121.6, 118.4, 16.5, -1.1.

HRMS (EI) m/z: [M]+ Calcd. for $C_9H_{14}OSi$, 166.0814; Found, 166.0813.

cyclohexyl(3-methoxyphenyl)(methyl)silane (B)

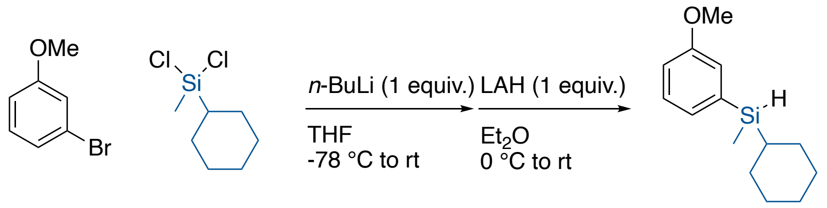

An oven dried 200 mL three-neck flask, equipped with a magnetic stirring bar and rubber septum was evacuated and backfilled with nitrogen. 1-Bromo-3-methoxybenzene (TCI, 1.71 g, 9.2 mmol) and THF (20 mL) were charged to the flask and cooled to -78 °C. *n*-BuLi (nacalai, 1.6 M in hexane, 6 mL, 1 equiv) was slowly added to the flask under -78 °C, then the mixture was stirred at -78 °C for 0.5 h. Dichlorocyclohexylmethylsilane (TCI, 2.54 g, 13.0 mmol, 1.4 equiv) was added to the flask dropwise, and the mixture was sttired at room temperature, overnight. Solvent was removed under reduced pressure after removing the insolvable solids by celite. Resulting crude mixture was used in the next step without further purification.

An oven dried 200 mL three-neck flask, equipped with a magnetic stirring bar and rubber septum was evacuated and backfilled with nitrogen. Crude chlorosilane and Et_2O (5 mL) were charged to the flask and cooled to 0 °C. LAH (Aldrich, 600 mg, 15.7 mmol, 1.7 equiv) was slowly added to the flask under 0 °C, then the mixture was stirred at room temperature overnight. Water was added to the flask. The organic products were extracted by Et_2O, washed with Brine, and dried over $MgSO_4$. Solvent was removed under reduced pressure. The resulting crude materials were purified by column chromatography on silica gel (SiliaFlash; eluent: hexane: Et_2O = 99: 1) and kügelrhor distillation. Cyclohexyl(3-methoxyphenyl)(methyl)silane (**B**) (1.52 g, 6.50 mmol, 71% yield) was obtained as a colorless oil.

^{1}H NMR (400 MHz, $CDCl_3$) δ 7.30 (dd, J = 8.2, 7.3 Hz, 1H), 7.10 (dt, J = 7.2, 1.1 Hz, 1H), 7.06 (d, J = 2.7 Hz, 1H), 6.92 (ddd, J = 8.2, 2.7, 0.9 Hz, 1H), 4.17 (qd, J = 3.8, 2.5 Hz, 1H), 3.83 (s, 3H), 1.72-1.70 (m, 5H), 1.23-1.12 (m, 5H), 0.96-0.88 (m, 1H), 0.31 (d, J = 3.7 Hz, 3H).

^{13}C NMR (101 MHz, $CDCl_3$) δ 158.9, 137.6, 129.0, 127.0, 120.2, 114.4, 55.2, 28.3, 28.0, 27.9, 26.9, 24.3, -7.7.

HRMS (EI) m/z: [M]+ Calcd. for $C_{14}H_{22}OSi$, 234.1440; Found, 234.1437.

***tert*-butyl(butyl)(phenyl)silane (C)**

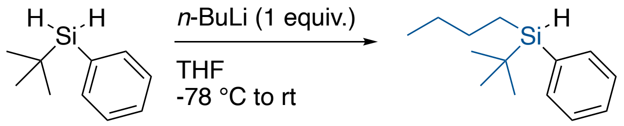

An oven dried 200 mL three-neck flask, equipped with a magnetic stirring bar and rubber septum was evacuated and backfilled with nitrogen. *tert*-butyl(phenyl)silane (synthesized by reported method,[7] 800 mg, 4.88 mmol) and THF (30 mL) were charged to the flask and cooled to -78 °C. *n*-BuLi (nacalai, 1.6 M in hexane, 10 mL, 1 equiv.) was slowly added to the flask under -78 °C, then the mixture was allowed to stir at room temperature, overnight. Water was added to the flask. The organic products were extracted by Et_2O, washed with Brine, and dried over Na_2SO_4. Solvent was removed under reduced pressure. The resulting crude materials were purified by column chromatography on silica gel (SiliaFlash; eluent: gradient from hexane only) and kügelrhor distillation. *tert*-Butyl(butyl)(phenyl)-silane (**C**) (659 mg, 2.99 mmol, 61% yield) was obtained as a white solid.

^{1}H NMR (400 MHz, $CDCl_3$) δ 7.53 (td, J = 3.9, 2.0 Hz, 2H), 7.41-7.33 (m, 3H), 4.04 (dd, J = 4.6, 3.2 Hz, 1H), 1.40-1.31 (m, 4H), 0.94 (s, 9H), 0.91-0.84 (m, 5H).

^{13}C NMR (101 MHz, $CDCl_3$) δ 135.5, 134.9, 129.2, 127.7, 27.4, 27.2, 26.4, 17.0, 13.8, 8.9.

HRMS (EI) m/z: [M]+ Calcd. for $C_{14}H_{24}Si$, 220.1647; Found, 220.1639.

methyl(phenyl)(3,3,3-trifluoropropyl)silane (**D**)

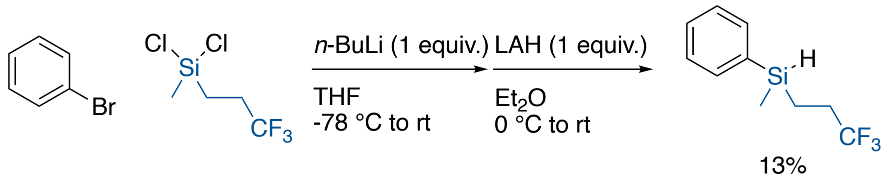

An oven dried 200 mL three-neck flask, equipped with a magnetic stirring bar and rubber septum was evacuated and backfilled with nitrogen. Bromobenzene (TCI, 1.7 g, 9.1 mmol) and THF (20 mL) were charged to the flask and cooled to -78 °C. *n*-BuLi (nacalai, 1.6 M in hexane, 6 mL, 1 equiv) was slowly added to the flask under -78 °C, then the mixture was stirred at -78 °C for 0.5 h. dichloro(methyl)(3,3,3-trifluoropropyl)silane (TCI, 1.91 g, 9.1 mmol, 1.0 equiv) was added to the flask dropwise, and the mixture was sttired at room temperature, overnight. Solvent was removed under reduced pressure after removing the insolvable solids by celite. Resulting crude mixture was used in the next step without further purification.

An oven dried 200 mL three-neck flask, equipped with a magnetic stirring bar and rubber septum was evacuated and backfilled with nitrogen. Crude chlorosilane and Et_2O (5 mL) were charged to the flask and cooled to 0 °C. LAH (Aldrich, 600 mg, 15.7 mmol, 1.7 equiv) was slowly added to the flask under 0 °C, then the mixture was stirred at room temperature overnight. Water was added to the flask. The organic products were extracted by Et_2O, washed with Brine, and dried over $MgSO_4$. Solvent was removed under reduced pressure. The resulting crude materials were purified by column chromatography on silica gel (SiliaFlash; eluent: hexane: Et_2O = 99 : 1) and kügelrhor distillation. Methyl(phenyl)(3,3,3-trifluoropropyl)silane (**D**) (259 mg, 1.19 mmol, 13% yield) was obtained as a colorless oil.

^{1}H NMR (400 MHz, $CDCl_3$) δ 7.54-7.51 (m, 2H), 7.44-7.36 (m, 3H), 4.42-4.38 (m, 1H), 2.15-1.99 (m, 2H), 1.12-0.99 (m, 2H), 0.39 (d, J = 3.7 Hz, 3H).

^{13}C NMR (101 MHz, $CDCl_3$) δ 134.5, 134.3, 129.9, 128.2, 29.3 (q, J_{CF} = 30 Hz, CF_3), 5.6, 5.6, -5.9.

HRMS (EI) m/z: [M]+ Calcd. for $C_{10}H_{13}F_3Si$, 218.0739; Found, 218.0742.

isopropyldiphenylsilane (**F**)

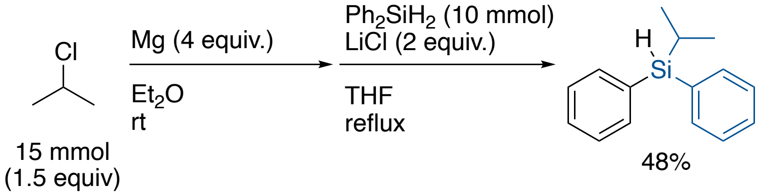

According to the reported method,[7] modified synthetic method was used. An oven dried 200 mL two-neck flask, equipped with a magnetic stirring bar, reflux condensor and rubber septum was charged with Magnesium (turnings, Nacalai, 991 mg, 41.3 mmol) then evacuated and backfilled with nitrogen. Et_2O (20 mL) were charged to the flask and 2-chloropropane (Nacalai, 1.19 g, 15 mmol) was added dropwise generating isopropylmagnesiumchloride.

An oven dried 200 mL two-neck flask, equipped with a magnetic stirring bar, reflux condensor and rubber septum was charged with LiCl (TCI, 987 mg, 23.5 mmol), diphenylsilane (BLD pharma, 1.78 g, 9.8 mmol) and THF (20 mL) in glovebox. The solution of hydrosilane was brought out of the glovebox, then isopropylmagnesiumchloride solution was added to the solution of hydrosilane by Teflon tube. The resulting mixture was heated at 80 °C for 12 h. After heating, water was added to the flask. The organic products were extracted by Et_2O, washed with Brine, and dried over Na_2SO_4. Solvent was removed under reduced pressure. The resulting crude materials were purified by column chromatography on silica gel (SiliaFlash; eluent: hexane

only) and preparative GPC. Isopropyldiphenylsilane (**F**) (1.05 g, 4.65 mmol, 48% yield) was obtained as a colorless oil. The spectrum data of the product suits to the reported literature.[8]

^{1}H NMR (400 MHz, CDCl$_3$) δ 7.66-7.63 (m, 4H), 7.47-7.39 (m, 6H), 4.76 (d, J = 3.2 Hz, 1H), 1.59-1.48 (m, 1H), 1.16 (d, J = 7.3 Hz, 6H).

^{13}C NMR (101 MHz, CDCl$_3$) δ 135.6, 134.0, 129.6, 128.0, 18.5, 11.8.

trimethyl((2-(methyl(phenyl)silyl)phenoxy)methyl)silane (**H**)

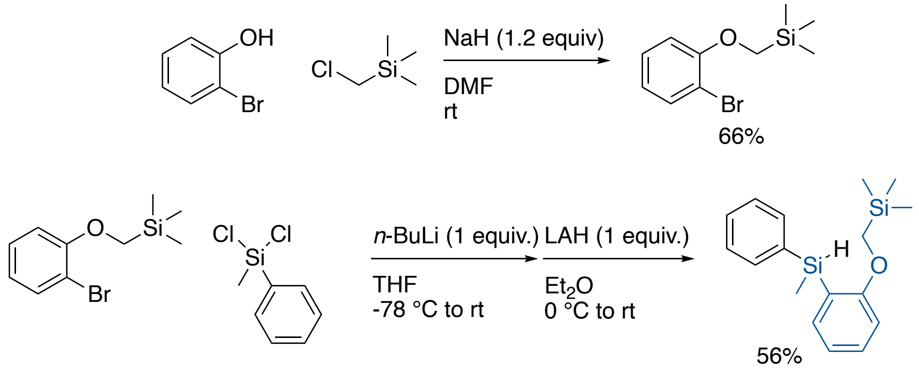

An oven dried 300 mL three-neck flask, equipped with a magnetic stirring bar and rubber septum was charged NaH (Nacalai, 1.39 g, 35 mmol, 1.1 equiv.) and DMF (Nacalai, 60 mL). The mixture cooled to 0 °C, following 2-bromophenol (TCI, 5.24 g, 30.3 mmol) was added dropwise. After H_2 generation finished, (chloromethyl)trimethylsilane (TCI, 5.89 g, 48 mmol, 1.6 equiv.) was added and the mixture was stired at room temperature overnight. Water was added to the flask. The organic products were extracted by Et_2O, washed with Brine, and dried over Na_2SO_4. Solvent was removed under reduced pressure. The resulting crude materials were purified by column chromatography on silica gel (SiliaFlash; eluent: hexane: DCM = 95 : 5) and kügelrhor distillation. ((2-bromophenoxy)methyl)-trimethylsilane (5.19 g, 20 mmol, 66 %) was obtained as a colorless oil.

An oven dried 200 mL three-neck flask, equipped with a magnetic stirring bar and rubber septum was evacuated and backfilled with nitrogen. ((2-bromophenoxy)methyl)-trimethylsilane (TCI, 1.45 g, 5.6 mmol) and THF (20 mL) were charged to the flask and cooled to -78 °C. *n*-BuLi (nacalai, 1.6 M in hexane, 4 mL, 1 equiv) was slowly added to the flask under -78 °C, then the mixture was stirred at -78 °C for 0.5 h. dichloro(methyl)(phenyl)silane (TCI, 1.6 g, 8.4 mmol, 1.5 equiv) was added to the flask dropwise, and the mixture was sttired at room temperature, overnight. Solvent was removed under reduced pressure after removing the insolvable solids by celite. Resulting crude mixture was used in the next step without further purification.

An oven dried 200 mL three-neck flask, equipped with a magnetic stirring bar and rubber septum was evacuated and backfilled with nitrogen. Crude chlorosilane and Et_2O (5 mL) were charged to the flask and cooled to 0 °C. LAH (Aldrich, 257 mg, 6.8 mmol, 1.2 equiv) was slowly added to the flask under 0 °C, then the mixture was stirred at room temperature overnight. Water was added to the flask. The organic products were extracted by Et_2O, washed with Brine, and dried over $MgSO_4$. Solvent was removed under reduced pressure. The resulting crude materials were purified by column chromatography on silica gel (SiliaFlash; eluent: hexane: DCM = 95 : 5) and kügelrhor distillation. Trimethyl((2-(methyl(phenyl)silyl)phenoxy)methyl)silane (**H**) (942 mg, 3.14 mmol, 56% yield) was obtained as a colorless oil.

^{1}H NMR (400 MHz, CDCl$_3$) δ 7.58-7.55 (m, 2H), 7.43-7.32 (m, 5H), 7.00 (d, J = 8.2 Hz, 1H), 6.94-6.91 (m, 1H), 4.97 (q, J = 3.8 Hz, 1H), 3.56 (s, 2H), 0.62 (d, J = 4.1 Hz, 3H), 0.05 (s, 9H).

^{13}C NMR (101 MHz, CDCl$_3$) δ 166.2, 136.8, 136.0, 134.9, 131.7, 129.2, 127.8, 123.2, 120.3, 109.4, 60.9, -3.0, -4.8.

HRMS (EI) m/z: [M]+ Calcd. for $C_{17}H_{24}OSi_2$, 300.1366; Found, 300.1361.

sec-butoxydiphenylsilane (I)

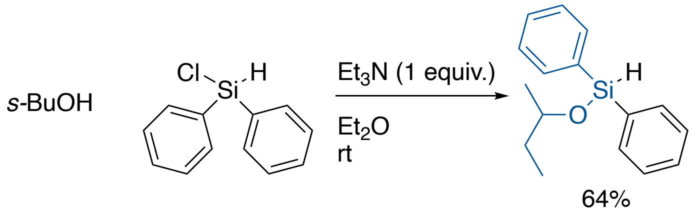

An oven dried 300 mL three-neck flask, equipped with a magnetic stirring bar and rubber septum was charged with *sec*-butylalcohol (Nacalai, 409 mg, 5.5 mmol, 2 equiv.), chlorodiphenylsilane (synthesized by reported method,[9] 696 mg, containg 30 mol% of Ph_3CH, 2.2 mmol) and Et_2O (15 mL). To the solution, Et_3N (336 mg, 3.3 mmol, 1.5 equiv) was added dropwise. The mixture was stired at room temperature overnight. Volatiles were removed under reduced pressure. Solid was filtered by celite pad. The resulting crude materials were purified by GPC. *sec*-Butoxydiphenylsilane (**I**) (367 mg, 1.43 mmol, 64 %) was obtained as a colorless oil. The spectrum data of the product suits to the reported literature.[10]

^{1}H NMR (400 MHz, $CDCl_3$) δ 7.66-7.63 (m, 4H), 7.46-7.37 (m, 6H), 5.47 (s, 1H), 3.95-3.88 (m, 1H), 1.66-1.45 (m, 2H), 1.21 (d, J = 6.4 Hz, 3H), 0.91 (t, J = 7.3 Hz, 3H).

^{13}C NMR (101 MHz, $CDCl_3$) δ 134.9, 134.7, 130.3, 128.0, 72.4, 32.1, 22.8, 10.2.

9-phenyl-9*H*-tribenzo[*b*,*d*,*f*]silepine (L)

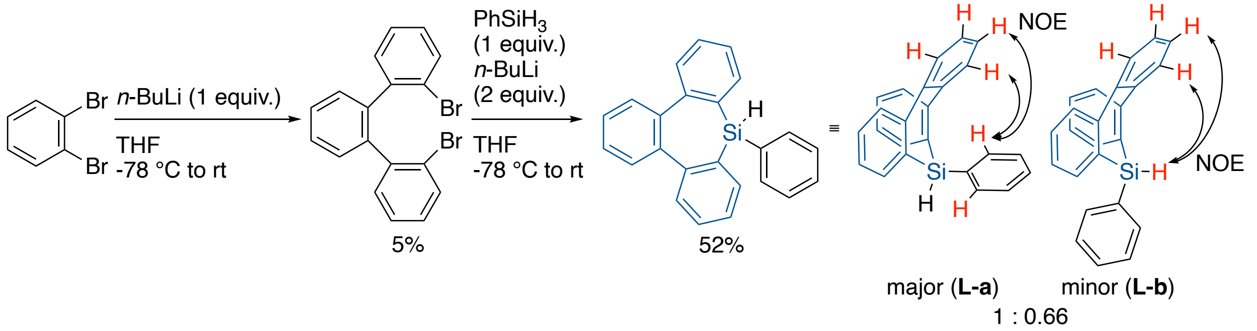

An oven dried 200 mL three-neck flask, equipped with a magnetic stirring bar and rubber septum was evacuated and backfilled with nitrogen. 1,2-dibromobenzene (TCI, 3.95 g, 16.7 mmol) and THF (30 mL) were charged to the flask and cooled to -78 °C. *n*-BuLi (nacalai, 1.6 M in hexane, 10 mL, 1 equiv.) was slowly added to the flask under -78 °C, then the mixture was stirred at -78 °C for 0.5 h afterward stirred at room temperature, overnight. Water was added to the flask. The organic products were extracted by Et_2O, washed with Brine, and dried over Na_2SO_4. Solvent was removed under reduced pressure. The resulting crude materials were purified by column chromatography on silica gel (SiliaFlash; eluent: gradient from hexane only to hexane : Et_2O = 90 : 10) (3,5- 2,2''-dibromo-1,1':2',1''-terphenyl (303.7 mg, 0.79 mmol, 5% yield) was obtained as a white solid.

According to the reported method,[11] an oven dried 200 mL three-neck flask, equipped with a magnetic stirring bar and rubber septum was evacuated and backfilled with nitrogen. (3,5- 2,2''-dibromo-1,1':2',1''-terphenyl (303.7 mg, 0.79 mmol) and THF (20 mL) were charged to the flask and cooled to -78 °C. *n*-BuLi (nacalai, 1.6 M in hexane, 1 mL, 2 equiv) was slowly added to the flask under -78 °C, then the mixture was stirred at -78 °C for 1 h. Phenylsilane (TCI, 190.5 mg, 1.71 mmol, 2.2 equiv) was added to the flask dropwise, and the mixture was sttired at room temperature, overnight. The resulting crude materials were directly purified by column chromatography on silica gel (SiliaFlash; eluent: hexane only) and preparative GPC. 9-phenyl-9*H*-tribenzo[*b*,*d*,*f*]silepine (**L**) (220.8 mg, 0.66 mmol, 52% yield, diastereomeric ratio = 1 : 0.66 (**L-a** and **L-b**)) was obtained as a white solid.

This isomerism arises from rigid seven-membered ring structure of which ring flip is prohibited. From the NOE measurements, the major isomer **L-a** possesses the phenyl group close to the terphenyl ring while minor isomer **L-b** possesses the hydride group close to the terphenyl ring whose chemical shift (5.14 ppm) was found at the higher field than **L-a** (5.51 ppm) or Ph_3SiH (5.48 ppm). It was reported that 9,9-dimethyl-9*H*-tribenzo[*b*,*d*,*f*]silepine shows two diastereotopic methyl signals in ^{1}H NMR.[12]

^{1}H NMR (400 MHz, CDCl$_3$) *δ* 8.01-7.99 (m, 1.3H, [**L-b** 2H]), 7.90 (d, J = 6.9 Hz, 2H, [**L-a** 2H]), 7.71-7.63 (m, 2H, [**L-b** 3H]), 7.59-7.49 (m, 10.5H, [**L-a** 4H, **L-b** 10H]), 7.47-7.38 (m, 4H, [**L-a** 4H]), 7.35-7.30 (m, 3H, [**L-a** 1H, **L-b** 2H]), 7.24-7.20 (m, 1H, [**L-a** 1H]), 7.16-7.15 (m, 2H, [**L-a** 2H]), 7.12-7.09 (m, 2H, [**L-a** 2H]), 5.51 (s, 1H, [**L-a** 1H]), 5.14 (s, 0.6H, [**L-b** 1H]).

^{13}C NMR (101 MHz, CDCl$_3$)

L-a : *δ* 146.8, 141.0, 138.3, 134.8, 134.2, 132.5, 132.3, 130.4, 130.3, 129.4, 127.5, 127.4, 126.7.

L-b : *δ* 146.9, 140.9, 137.7, 137.6, 132.4, 132.2, 130.8, 130.4, 130.0, 129.6, 128.7, 127.6, 126.5.

^{29}Si NMR (79 MHz, CDCl$_3$) *δ* -14.5 (**L-a**), -25.9 (**L-b**).

HRMS (EI) m/z: [M]+ Calcd. for $C_{24}H_{18}Si$, 334.1178; Found, 334.1170.

(3,5-di-*tert*-butyl-4-methoxyphenyl)diphenylsilane (M)

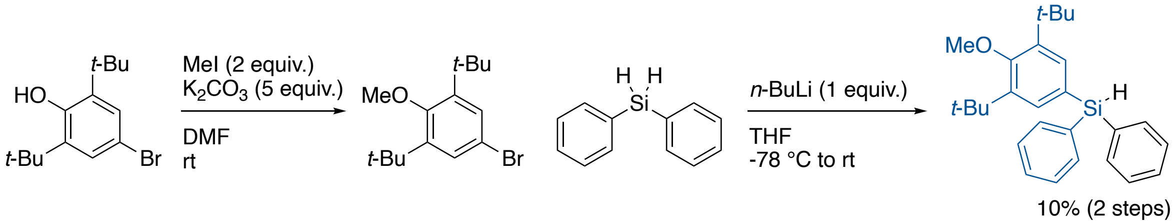

An oven dried 300 mL three-neck flask, equipped with a magnetic stirring bar and rubber septum was charged MeI (Nacalai, 1.21 g, 8.6 mmol, 4 equiv.), K_2CO_3 (Nacalai, 1.58 g, 11.4 mmol, 5 equiv.), 4-bromo-2,6-di-*tert*-butylphenol (Aldrich, 602 mg, 2.12 mmol) and DMF (Nacalai, 5 mL). The mixture was stired at room temperature overnight. Water was added to the flask. The organic products were extracted by AcOEt, washed with Brine, and dried over Na_2SO_4. Solvent was removed under reduced pressure. The resulting crude materials were purified by column chromatography on silica gel (SiliaFlash; eluent: hexane: AcOEt = 90: 10). All the resulting material was used to the next step after removing solvent under reduced pressure.

An oven dried 200 mL three-neck flask, equipped with a magnetic stirring bar and rubber septum was evacuated and backfilled with nitrogen. 5-bromo-1,3-di-*tert*-butyl-2-methoxybenzene and THF (20 mL) were charged to the flask and cooled to -78 °C. *n*-BuLi (nacalai, 1.6 M in hexane, 8 mL, 1 equiv.) was slowly added to the flask under -78 °C, then the mixture was stirred at -78 °C for 0.5 h. diphenylsilane (TCI, 317 mg, 1.74 mmol) was added to the flask dropwise, and the mixture was stirred at room temperature, overnight. Water was added to the flask. The organic products were extracted by Et_2O, washed with Brine, and dried over Na_2SO_4. Solvent was removed under reduced pressure. The resulting crude materials were purified by column chromatography on silica gel (SiliaFlash; eluent: gradient from hexane only to hexane : Et_2O = 90 : 10) and kügelrhor distillation. (3,5-di-*tert*-butyl-4-methoxyphenyl)diphenylsilane (**M**) (92.2 mg, 0.23 mmol, 10% yield over 2 steps) was obtained as a white solid.

^{1}H NMR (400 MHz, CDCl$_3$) *δ* 7.61 (dd, J = 7.5, 1.6 Hz, 4H), 7.47 (s, 2H), 7.44-7.35 (m, 6H), 5.45 (s, 1H), 3.71 (s, 3H), 1.39 (s, 18H).

^{13}C NMR (101 MHz, CDCl$_3$) *δ* 161.3, 143.2, 135.9, 134.4, 134.2, 129.7, 128.0, 126.3, 64.3, 35.8, 32.2.

HRMS (EI) m/z: [M]+ Calcd. for $C_{27}H_{34}OSi$, 402.2379; Found, 402.2375.

·Other substrates

A2, **A3**, **A5**, **A6**, **A7**, **A8**, **A9**, **A10**, **A12**, **A14**, **A17** and **A18** were synthesized according to the synthetic method of **A11**, **A15** and **A16** showing above. **A13**,[13] **E**,[14] **G**[14] and **K**[11] were each synthesized according to the reported methods. **A1**, **A4**, **J** and **O** were purchased from TCI and used as received.

3.2. Synthesis of Silylene Acceptors

5,5'-(ethene-1,1-diyl)bis(benzofuran) (1l)

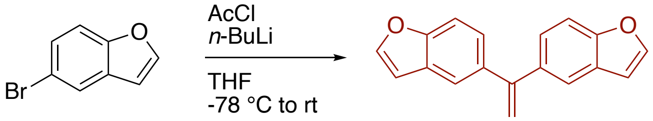

1l 29%

An oven dried 200 mL three-neck flask, equipped with a magnetic stirring bar and rubber septum was evacuated and backfilled with nitrogen. 5-Bromobenzofuran (TCI, 271mg, 1.38 mmol) and THF (20 mL) were charged to the flask and cooled to -78 °C. *n*-BuLi (nacalai, 1.6 M in hexane, 0.8 mL, 1 equiv) was slowly added to the flask under -78 °C, then the mixture was stirred at -78 °C for 0.5 h. Acetylchloride (TCI, 347 mg, 4.5 mmol, 3.2 equiv) was added to the flask dropwise, and the mixture was sttired at room temperature, overnight. Water was added to the flask. The organic products were extracted by AcOEt, washed with Brine, and dried over Na_2SO_4. Sol;vent was removed under reduced pressure. The resulting crude materials were purified by column chromatography on silica gel (SiliaFlash; eluent: hexane: DCM = 95 : 5) and kügelrhor distillation. 5,5'-(ethene-1,1-diyl)bis(benzofuran) (**1l**) (52.4 mg, 0.2 mmol, 29% yield) was obtained as a white solid.

^{1}H NMR (400 MHz, $CDCl_3$) δ 7.65 (d, J = 2.3 Hz, 2H), 7.60 (d, J = 1.4 Hz, 2H), 7.50-7.47 (m, 2H), 7.34 (dd, J = 8.2, 1.8 Hz, 2H), 6.75 (dd, J = 2.3, 0.9 Hz, 2H), 5.49 (s, 2H).

^{13}C NMR (101 MHz, $CDCl_3$) δ 154.8, 150.5, 145.6, 137.2, 127.4, 125.1, 121.2, 113.8, 111.0, 106.9.

HRMS (EI) m/z: [M]+ Calcd. for $C_{18}H_{12}O_2$, 260.0837; Found, 260.0834.

·Other substrates

1a, **1d**, **1i**, **3a**, **3b**, **3c**, **7**, **9**, **11** and **1m** (*Khellin*) were purchased from TCI and **3d** was purchased from Aldrich and used as received. **1j**,[15] **3e**,[16] and **3f**[17] were each synthesized according to the reported methods. Benzofuran **1b**,[18] **1k**,[18] **1f**,[19] **1g**[19] and **1h**[19] were synthesized according to the reported method. Benzofuran **1c** and **1e** were synthesized according to the synthetic method of **1l**, Me_3SiCl (TCI) was used as an electrophile for **1c** and DMA (Super dehydrated, stabilizer free, Wako) was used as an electrophile for **1e**. **5** was synthesized according to reported method.[20,21]

4. Reaction Conditions of Iridium-Catalyzed Diorganosilylene Insertion into C(sp^2)–O Bond of Oxacycles Using Aryldiorganosilanes as Silylene Transfer Reagents

General procedure : In a glovebox, an oven dried glass tube (outside diameter : 20 mm) equipped with a magnetic stirring bar and PTFE stopcock (J. Young) was charged with $[IrCl(cod)]_2$ (0.004 mmol, 2 mol%), (*S*)-DTBM-SEGPHOS (0.008 mmol, 4 mol%) and solvent (0.4 mL, 0.5 M). The solution was stirred at room temperature for 5 minutes, to which furan (0.2 mmol) and subsequently silane (0.22 mmol, 1.1 equiv) were added. The tube was sealed by the stopcock and was taken out from the glovebox. The mixture was stirred for 12 h at 80 °C by a heating magnetic stirrer with an aluminum heating block (hole size: 21 mm diameter x 33 mm depth). The product 1,2-oxasiline was purified by column chromatography on silica gel, preparative GPC or kügel distillation. NMR yields were determined by ^{1}H NMR of the crude mixture with diphenylmethan as the internal standard.

4.1. Detection of Eliminated Benzene

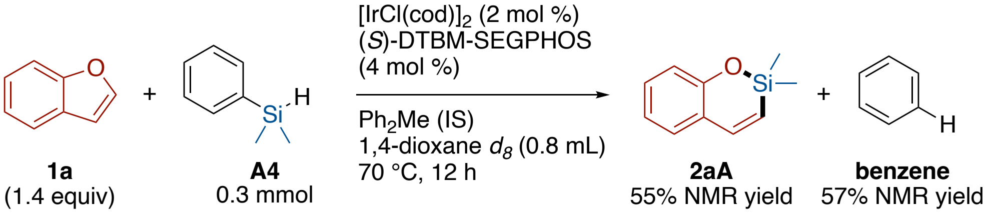

According to the **General Procedure**, a mixture of [IrCl(cod)]$_2$ (5.1 mg, 0.0075 mmol, 2 mol%), (*S*)-DTBM-SEGPHOS (15.5 mg, 0.013 mmol, 4 mol%), benzofuran **1a** (47.2 mg, 0.4 mmol, 1.4 equiv.), dimethylphenylsilane **A4** (39.9 mg, 0.29 mmol), diphenylmethane (26.4 mg, 0.16 mmol, internal standard) and 1,4-dioxane-*d*$_8$ (0.8 mL) was heated in an NMR tube equipped with PTFE stopcock (Wilmad, outside diameter : 5 mm) for 12 h at 70 °C.The NMR spectrum of the resultant mixture showed a signal of benzene at 7.296 ppm (s) (57% NMR yield) as shown in Figure S1. The product **2aA** was formed in 55% NMR yield.

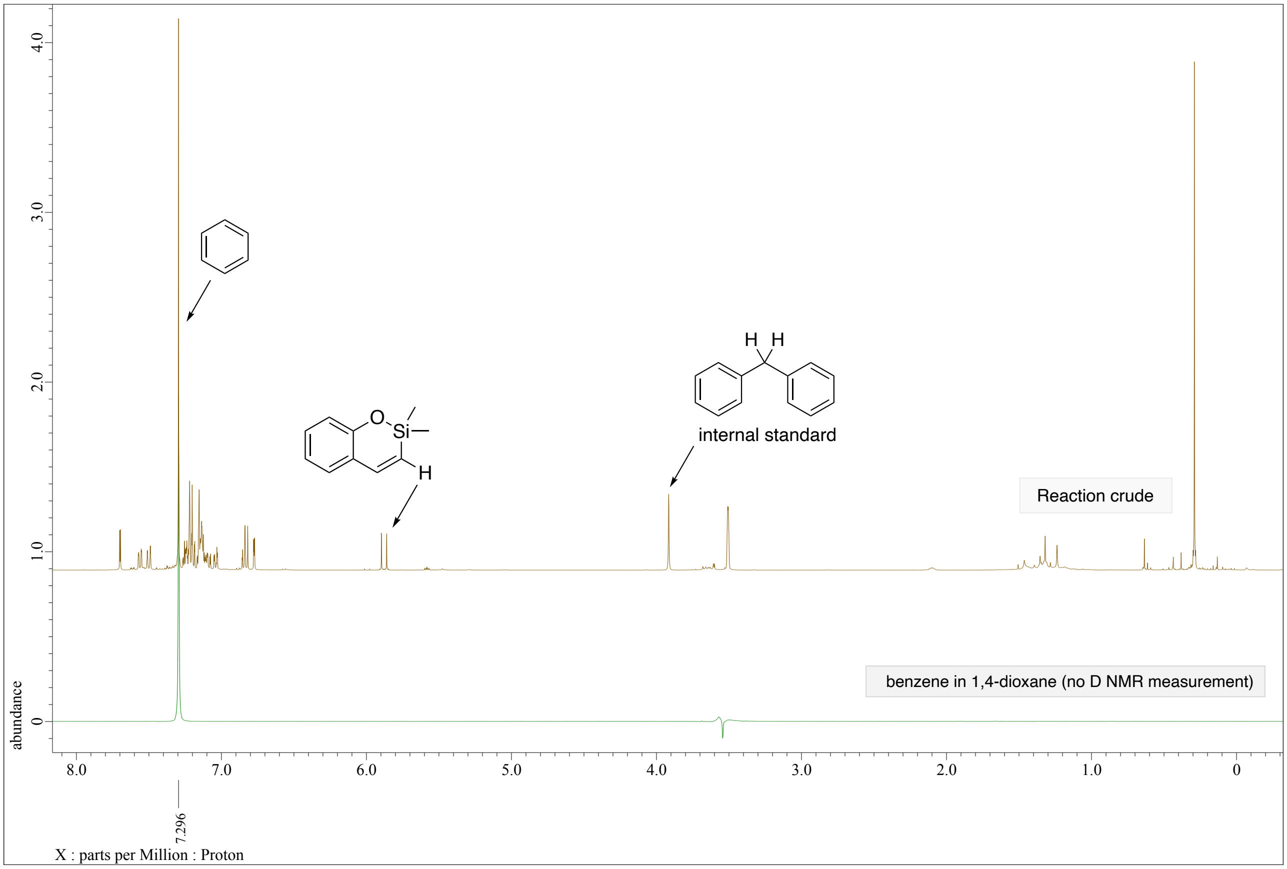

Figure S1. Detection of Eliminated Benzene.

4.2. Reaction Conditions

Table S1. Full detail of Catalyst Screening.

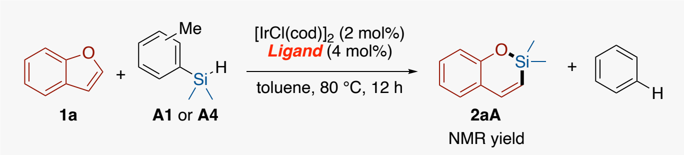

Transition Metal Precursors

entry	Transition Metals	Ligand	NMR yield of **2aA**
1	$[Ir(cod)(OMe)]_2$	(*S*)-DTBM-SEGPHOS	67%
2	$[Ir(cod)_2](BF_4)$	(*S*)-DTBM-SEGPHOS	0%
3	$[Rh(cod)Cl]_2$	(*S*)-DTBM-SEGPHOS	0%
4	$[Cu(OAc)_2]$	(*S*)-DTBM-SEGPHOS	0%

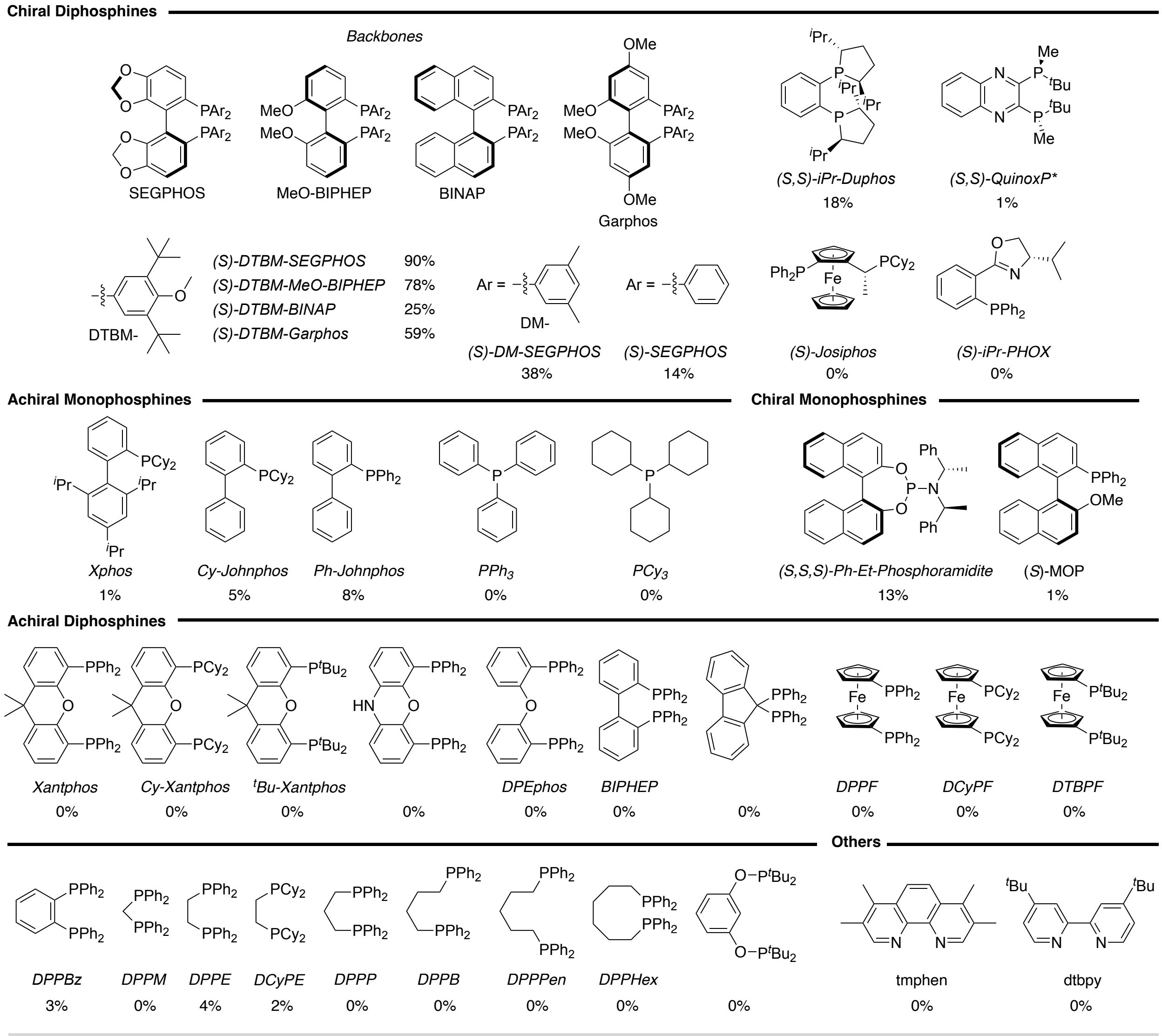

Table S2. Reaction Temperature

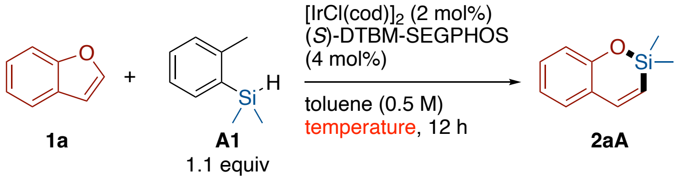

entry	temperature	NMR yield
1	20	28%
2	40	85%
3	60	91%
4	80	90%
5	110	84%
6	135	78%
7	150	73%

Table S3. Solvent Screening

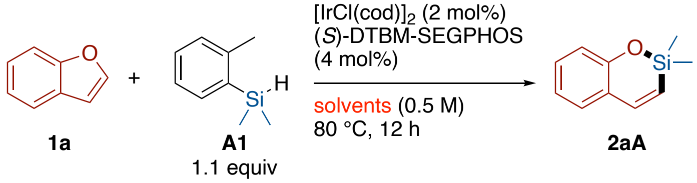

entry	solvents	NMR yield
1	AcOEt	92%
2	toluene	90%
3	benzene	87%
4	cyclohexane	82%
5	MTBE	82%
6	1,4-dioxane	77%
7	1,2-dichloroethane	68%
8	1-chlorohexane	5%
9	THF	4%
10	MeCN	2%
11	DMF	0%

Table S4. Ratio between Benzofurans and Hydrosilanes

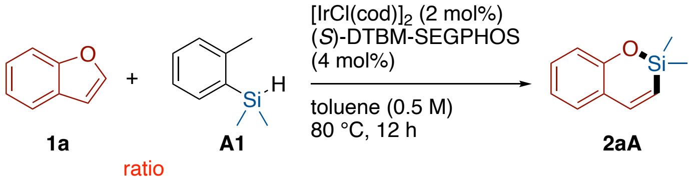

entry	ratio	NMR yield
1	1.15 : 1.00	86%
2	1.39 : 1.00	84%
3	2.01 : 1.00	85%
4	2.89 : 1.00	85%
5	1.00 : 1.08	88%
6	1.00 : 1.10	90%
7	1.00 : 1.24	89%
8	1.00 : 1.87	81%
9	1.00 : 3.44	77%
10	1.00 : 5.12	80%

Note: Benzofuran:hydrosilane = 1:1.0 ~ 1.2 is the best ratio for the reaction. Excess amount of hydrosilanes (more than 1.3 equivalent to benzofuran) would have negative influence on the reaction, while excess amount of benzofuran didn't affect the reaction largly.

4.3. Functional Group Tolerance Test

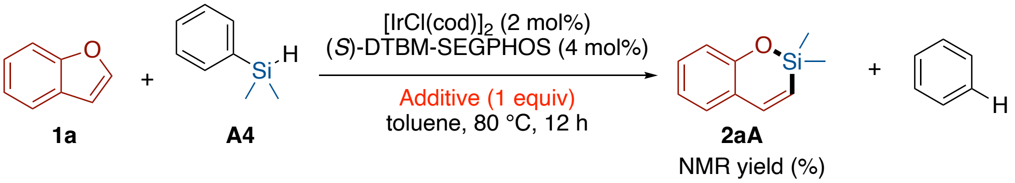

According to the **General Procedure**, a mixture of 100 μL of Ir catalyst solution (*[IrCl(cod)]$_2$ (41.28 mg, 0.0615 mmol, 2 mol%) and (S)-DTBM-SEGPHOS (144.26 mg, 0.122 mmol, 4 mol%) were dissolved in 3 mL of benzene*), 150 μL of substrate solution (*benzofuran **1a** (376.5 mg, 3.12 mmol), dimethylphenylsilane **A4** (455.58 mg, 3.35 mmol, 1.1 equiv.) and diphenylmethane (289.95 mg, 1.72 mmol, internal standard) were dissolved in 3 mL of benzene*) and additive (0.1 mmol, 1 equiv.) was heated for 12 h at 80 °C. Yield of **2aA** was measured by ^{1}H NMR.

4.4. Unsuccessful Substrates

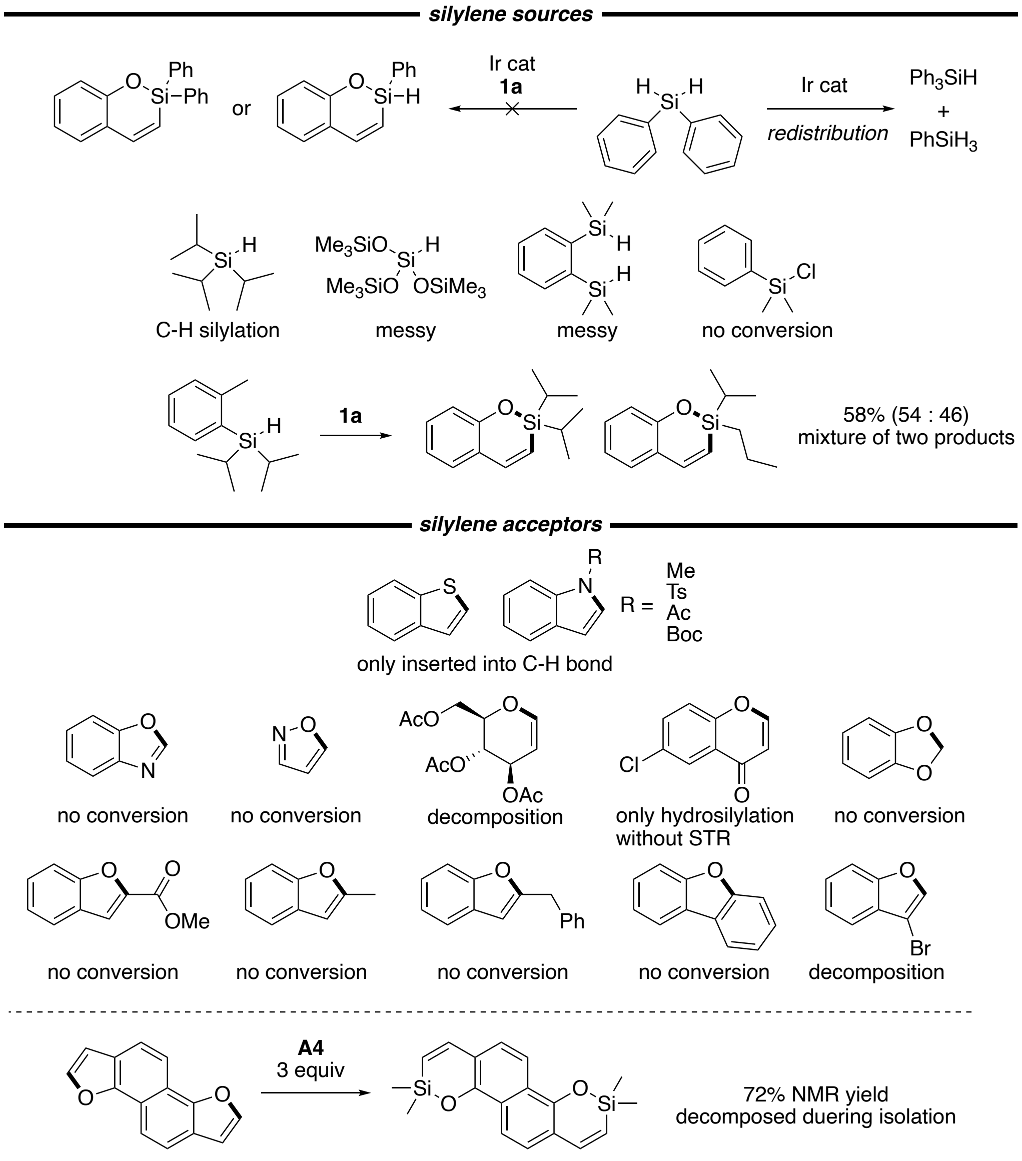

Figure S2. Unsuccessful Substrates.

4.5. Compound Data

2,2-dimethyl-2*H*-benzo[1,2]oxasiline (2aA)

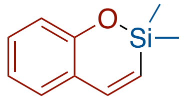

According to the **General Procedure**, a mixture of **1a** (16.1 mg, 0.136 mmol), **A4** (23.1 mg, 0.17 mmol, 1.2 equiv), $[IrCl(cod)]_2$ (1.7 mg, 0.002 mmol, 2 mol%) and (*S*)-DTBM-SEGPHOS (6.9 mg, 0.004 mmol, 4 mol%) in toluene (0.2 mL) was heated for 12 h at 80 °C. **2aA** was obtained as a colorless oil in 76% yield (18.2 mg, 0.103 mmol) after purification by column chromatography on silica gel (SiliaFlash; eluent: hexane: Et_2O = 95: 5) and kügelrhor distillation.

^{1}H NMR (400 MHz, $CDCl_3$) δ 7.26 (d, J = 14.6 Hz, 1H), 7.15 (td, J = 7.8, 1.7 Hz, 1H), 7.07 (dd, J = 7.5, 1.6 Hz, 1H), 6.92-6.87 (m, 2H), 5.91 (d, J = 14.6 Hz, 1H), 0.35 (s, 6H).

^{13}C NMR (101 MHz, $CDCl_3$) δ 153.7, 145.4, 130.6, 129.4, 124.5, 123.2, 120.7, 119.4, 1.6.

^{29}Si NMR (79 MHz, $CDCl_3$) δ 8.0.

HRMS (EI) m/z: [M]+ Calcd. for $C_{10}H_{12}OSi$, 176.0657; Found, 176.0655.

2-cyclohexyl-2-methyl-2*H*-benzo[1,2]oxasiline (2aB)

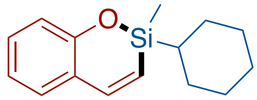

According to the **General Procedure**, a mixture of **1a** (18.0 mg, 0.15 mmol), cyclohexyl(3-methoxyphenyl)(methyl)silane **B9** (41.0 mg, 0.17 mmol, 1.1 equiv) and 130 µL of Ir catalyst stock solution ($[IrCl(cod)]_2$ (3.1 mg, 0.0046 mmol, 2 mol%), (*S*)-DTBM-SEGPHOS (9.9 mg, 0.0083 mmol, 4 mol%) and benzene (0.2 mL)) in benzene (270 µL, in total 400 µL, 0.5 M) was heated for 12 h at 80 °C. **2aB** was obtained as a colorless oil in 81% yield (29.8 mg, 0.12 mmol) after purification by column chromatography on silica gel (SiliaFlash; eluent: hexane: Et_2O = 95: 5) and preparative GPC.

^{1}H NMR (400 MHz, $CDCl_3$) δ 7.30 (d, J = 14.6 Hz, 1H), 7.15 (td, J = 7.5, 1.5 Hz, 1H), 7.06 (dd, J = 8.0, 1.6 Hz, 1H), 6.90-6.87 (m, 2H), 5.88 (d, J = 14.2 Hz, 1H), 1.75-1.68 (m, 5H), 1.23-1.16 (m, 5H), 0.90-0.82 (m, 1H), 0.31 (s, 3H).

^{13}C NMR (101 MHz, $CDCl_3$) δ 154.2, 146.1, 130.7, 129.4, 124.6, 121.6, 120.6, 119.3, 27.8, 27.6, 27.6, 26.8, 26.1, 26.0, -2.2.

HRMS (EI) m/z: [M]+ Calcd. for $C_{15}H_{20}OSi$, 244.1283; Found, 244.1282.

2-(*tert*-butyl)-2-butyl-2*H*-benzo[1,2]oxasiline (2aC)

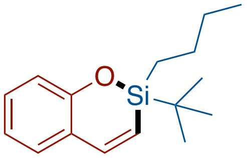

According to the **General Procedure**, a mixture of **1a** (25.0 mg, 0.21 mmol), *tert*-butyl(butyl)(phenyl)silane **C** (40.6 mg, 0.18 mmol, 0.9 equiv) and 130 µL of Ir catalyst stock solution with benzene ($[IrCl(cod)]_2$ (3.1 mg, 0.0046 mmol, 2 mol%), (*S*)-DTBM-SEGPHOS (9.9 mg, 0.0083 mmol, 4 mol%) and benzene (0.2 mL)) in benzene (270 µL, in total 400 µL, 0.5 M) was heated at 80 °C for 12 h. **2aC** was obtained as a colorless oil in 68% yield (31.9 mg, 0.12 mmol) after purification by column chromatography on silica gel (SiliaFlash; eluent: hexane: Et_2O = 95: 5) and preparative GPC.

^{1}H NMR (400 MHz, CDCl$_3$) δ 7.35 (d, J = 14.6 Hz, 1H), 7.15-7.11 (m, 1H), 7.05 (dd, J = 7.5, 1.6 Hz, 1H), 6.89-6.84 (m, 2H), 5.87 (d, J = 14.6 Hz, 1H), 1.39-1.27 (m, 4H), 0.93 (s, 9H), 0.83 (m, J = 7.1 Hz, 4H), 0.77-0.67 (m, 1H).
^{13}C NMR (101 MHz, CDCl$_3$) δ 154.6, 147.0, 130.7, 129.4, 124.6, 120.4, 120.0, 119.2, 26.3, 25.4, 25.3, 19.5, 13.8, 12.5.
HRMS (EI) m/z: [M]+ Calcd. for $C_{16}H_{24}OSi$, 260.1596; Found, 260.1594.

2-methyl-2-(3,3,3-trifluoropropyl)-2*H*-benzo[1,2]oxasiline (2aD)

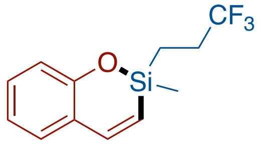

According to the **General Procedure**, a mixture of **1a** (18.6 mg, 0.158 mmol, 1.1 equiv), methyl(phenyl)(3,3,3-trifluoropropyl)silane **D** (32.7 mg, 0.149 mmol) and 130 µL of Ir catalyst stock solution ([IrCl(cod)]$_2$ (3.1 mg, 0.0046 mmol, 2 mol%), (*S*)-DTBM-SEGPHOS (9.9 mg, 0.0083 mmol, 4 mol%) and benzene (0.2 mL)) in benzene (270 µL, in total 400 µL, 0.5 M) was heated at 80 °C for 12 h. **2aD** was obtained as a colorless oil in 43% yield (16.6 mg, 0.0643) after purification by column chromatography on silica gel (SiliaFlash; eluent: hexane: Et$_2$O = 95: 5) and preperative GPC.
^{1}H NMR (400 MHz, CDCl$_3$) δ 7.34 (d, J = 14.6 Hz, 1H), 7.17 (td, J = 7.7, 1.5 Hz, 1H), 7.08 (dd, J = 7.5, 1.6 Hz, 1H), 6.93-6.87 (m, 2H), 5.85 (d, J = 14.2 Hz, 1H), 2.15-2.03 (m, 2H), 1.11-0.86 (m, 2H), 0.39 (s, 3H).
^{13}C NMR (101 MHz, CDCl$_3$) δ 153.6, 147.4, 130.9, 129.8, 129.0, 126.3, 124.1, 121.1, 120.3, 119.3, 27.7 (q, J_{CF} = 31 Hz, CF_3), 10.0, 10.0, -0.1.
^{29}Si NMR (79 MHz, CDCl$_3$) δ 7.2.
HRMS (EI) m/z: [M]+ Calcd. for $C_{12}H_{13}OF_3Si$, 258.0688; Found, 258.0682.

2-methyl-2-phenyl-2*H*-benzo[1,2]oxasiline (2aE)

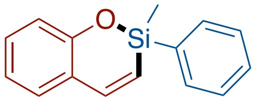

According to the **General Procedure**, a mixture of **1a** (22.4 mg, 0.19 mmol), methyldiphenylsilane **E** (42.7 mg, 0.22 mmol, 1.1 equiv), [IrCl(cod)]$_2$ (2.4 mg, 0.0036 mmol, 2 mol%) and (*S*)-DTBM-SEGPHOS (12.55 mg, 0.01 mmol, 5 mol%) in toluene (0.4 mL) was heated at 80 °C for 12 h. **2aE** was obtained as a colorless oil in 98% yield (44.3 mg, 0.19 mmol) after purification by column chromatography on silica gel (SiliaFlash; eluent: hexane: Et$_2$O = 95: 5) and preparative GPC.
^{1}H NMR (400 MHz, CDCl$_3$) δ 7.60 (dt, J = 5.9, 1.6 Hz, 2H), 7.44-7.36 (m, 4H), 7.20-7.15 (m, 1H), 7.14 (dd, J = 7.8, 1.8 Hz, 1H), 6.95-6.91 (m, 2H), 6.03 (d, J = 14.2 Hz, 1H), 0.67 (s, 3H)
^{13}C NMR (101 MHz, CDCl$_3$) δ 153.6, 146.7, 136.7, 133.8, 130.8, 130.4, 129.6, 128.1, 124.3, 121.4, 120.9, 119.5, 77.4, 77.1, 76.8, -0.7
HRMS (EI) m/z: [M]+ Calcd. for $C_{15}H_{14}OSi$, 238.0814; Found, 238.0808.

2-isopropyl-2-phenyl-2*H*-benzo[1,2]oxasiline (2aF)

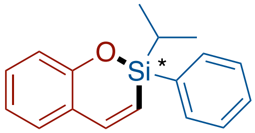

According to the **General Procedure**, a mixture of **1a** (24.4mg, 0.207 mmol), **F** (51.0 mg, 0.226 mmol, 1.1 equiv) and 400 µL of Ir catalyst stock solution ($[IrCl(cod)]_2$ (3.0 mg, 0.0044 mmol, 2 mol%), (*S*)-DTBM-SEGPHOS (9.7 mg, 0.0082 mmol, 4 mol%) and benzene (400 µL, 0.5M)) was heated at 80 °C for 12 h. **2aF** was ontained as a colorless oil in 86% yield (47.4 mg, 0.178 mmol) after purification by column chromatography on silica gel (SiliaFlash; eluent: hexane: Et_2O = 95: 5) and preparative GPC. Enantiomeric excess of **2aF** was determined to be 14% by SFC analysis [column: Daicel Chiralcel OJ-H/SFC (4.6 mm x 250 mm); eluent: CO_2 : IPA = 1%; flow rate: 3.03 mL/min; detection wavelength: 220 nmm; T_R = 19.9 min. (major), 24.5 min. (minor); Rs: 3.5]

^{1}H NMR (400 MHz, $CDCl_3$) δ 7.64 (dd, J = 7.5, 2.1 Hz, 2H), 7.49 (d, J = 14.2 Hz, 1H), 7.45-7.38 (m, 3H), 7.22-7.17 (m, 1H), 7.14 (dd, J = 7.3, 1.4 Hz, 1H), 6.99 (d, J = 8.2 Hz, 1H), 6.93 (td, J = 7.4, 1.1 Hz, 1H), 6.10 (d, J = 14.2 Hz, 1H), 1.44 (septet, J = 7.2 Hz, 1H), 1.13 (d, J = 7.3 Hz, 3H), 1.10 (d, J = 7.8 Hz, 3H).

^{13}C NMR (101 MHz, $CDCl_3$) δ 154.1, 147.6, 135.2, 134.0, 130.9, 130.2, 129.7, 128.1, 124.5, 120.8, 119.5, 119.1, 16.5, 16.3, 14.2.

HRMS (EI) m/z: [M]+ Calcd. for $C_{17}H_{18}OSi$, 266.1127; Found, 266.1126.

2-(*tert*-butyl)-2-phenyl-2*H*-benzo[1,2]oxasiline (2aG)

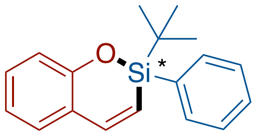

According to the **General Procedure**, a mixture of **1a** (11.9mg, 0.1 mmol), **G** (26.4 mg, 0.11 mmol, 1.1 equiv), $[IrCl(cod)]_2$ (1.7 mg, 0.0024 mmol, 2 mol%) and (*S*)-DTBM-SEGPHOS (5.9 mg, 0.0049 mmol, 4 mol%) in toluene (0.2 mL) was heated at 80 °C for 12 h. **2aG** was obtained as a white solid in 91% yield (25.6 mg, 0.09) after purification by column chromatography on silica gel (SiliaFlash; eluent: hexane: Et_2O = 95: 5). Enantiomeric excess of **2aG** was determined to be 82% by SFC analysis [column: Daicel Chiralcel OJ-H/SFC (4.6 mm x 250 mm); eluent: CO_2 : IPA = 5%; flow rate: 3.15 mL/min; detection wavelength: 220 nmm; T_R = 11.0 min. (minor), 14.2 min. (major); Rs: 3.7]

^{1}H NMR (400 MHz, $CDCl_3$) δ 7.65-7.62 (m, 2H), 7.44-7.37 (m, 4H), 7.20-7.16 (m, 1H), 7.09 (dd, J = 7.8, 1.4 Hz, 1H), 7.02 (d, J = 7.8 Hz, 1H), 6.90 (t, J = 7.3 Hz, 1H), 6.19 (d, J = 14.6 Hz, 1H), 1.03 (s, 9H).

^{13}C NMR (101 MHz, $CDCl_3$) δ 154.2, 147.3, 134.7, 133.9, 130.9, 129.9, 129.6, 127.9, 124.6, 120.8, 119.4, 118.9, 25.4, 19.5.

HRMS (EI) m/z: [M]+ Calcd. for $C_{18}H_{20}OSi$, 280.1283; Found, 280.1275.

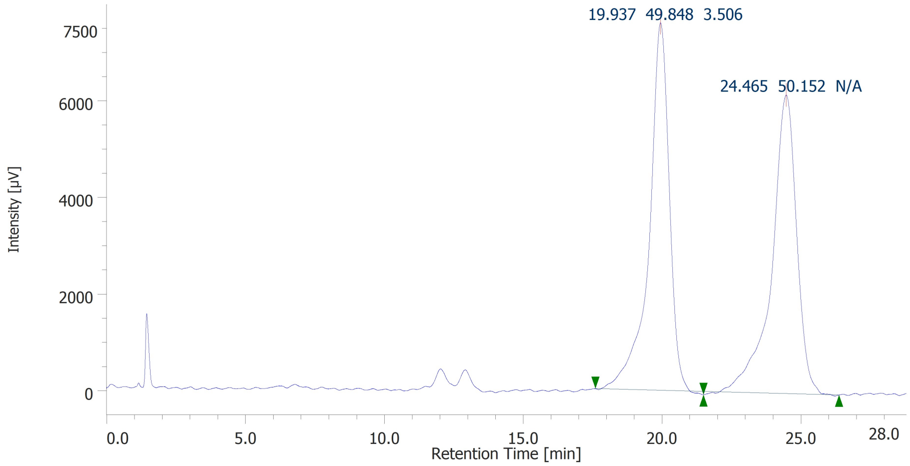

Figure S3. SFC Chart for (*rac*)-**2aF** [column: Daicel Chiralcel OJ-H/SFC (4.6 mm x 250 mm); eluent: CO_2 : IPA = 1%; flow rate: 3.03 mL/min; detection wavelength: 220 nmm; T_R = 19.9 min., 24.5 min.; Rs: 3.5].

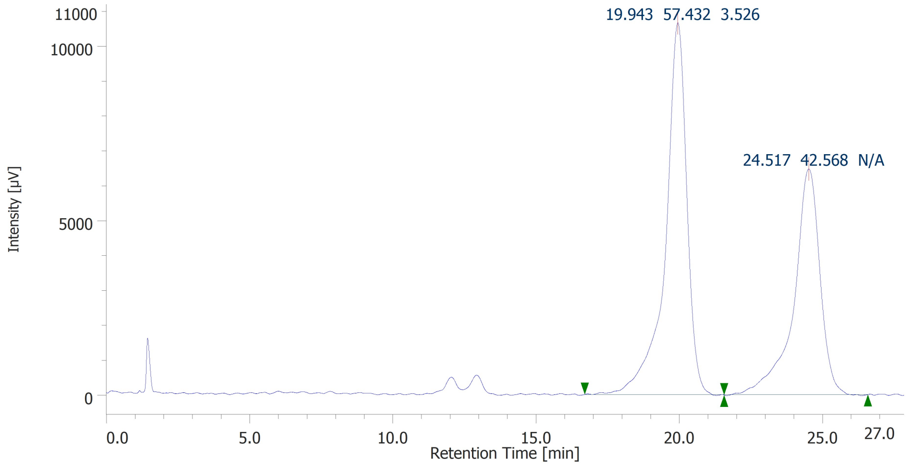

Figure S4. SFC Chart for (chiral)-**2aF** [column: Daicel Chiralcel OJ-H/SFC (4.6 mm x 250 mm); eluent: CO_2 : IPA = 1%; flow rate: 3.03 mL/min; detection wavelength: 220 nm; T_R = 19.9 min. (major), 24.5 min. (minor); Rs: 3.5].

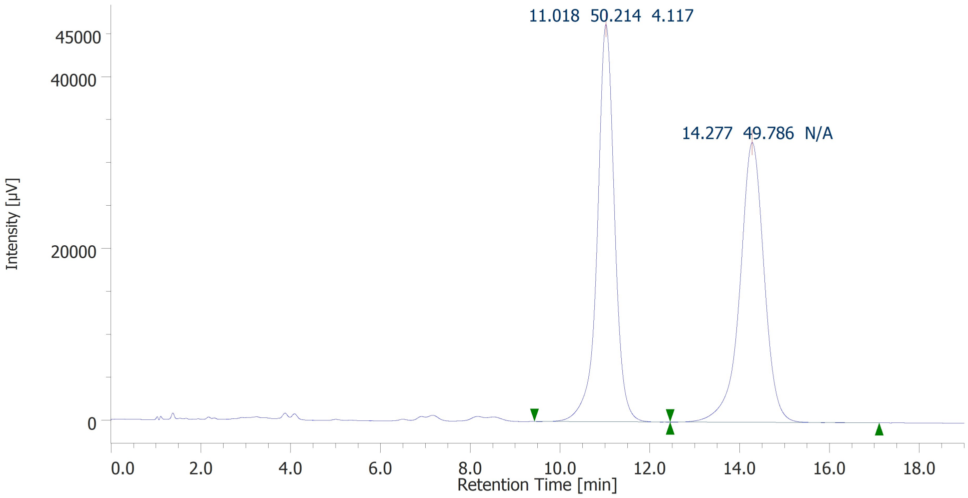

Figure S5. SFC Chart for (*rac*)-**2aG** [column: Daicel Chiralcel OJ-H/SFC (4.6 mm x 250 mm); eluent: CO_2 : IPA = 5%; flow rate: 3.15 mL/min; detection wavelength: 220 nmm; T_R = 11.0 min., 14.3 min.; Rs: 4.1].

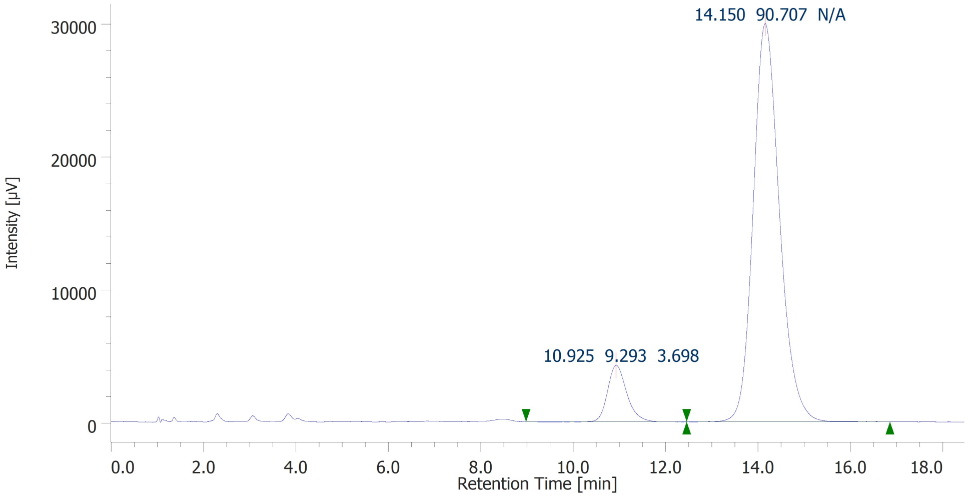

Figure S6. SFC Chart for (chiral)-**2aG** [column: Daicel Chiralcel OJ-H/SFC (4.6 mm x 250 mm); eluent: CO_2 : IPA = 5%; flow rate: 3.15 mL/min; detection wavelength: 220 nm; T_R = 11.0 min. (minor), 14.2 min. (major); Rs: 3.7].

2-methyl-2-(2-((trimethylsilyl)methoxy)phenyl)-2*H*-benzo[1,2]oxasiline (2aH)

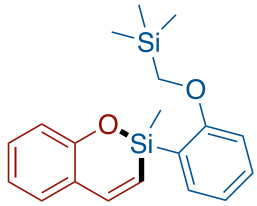

According to the **General Procedure**, a mixture of **1a** (22.6mg, 0.191 mmol), **H** (60.4 mg, 0.201 mmol, 1.1 equiv), 400 µL of Ir catalyst stock solution (containing $[IrCl(cod)]_2$ (3.0 mg, 0.0044 mmol, 2 mol%) and (*S*)-DTBM-SEGPHOS (9.7 mg, 0.0082 mmol, 4 mol%) in benzene (400 µL, 0.5M)) was heated at 80 °C for 12 h. **2aH** was obtained as a colorless oil in 79% yield (51.5 mg, 0.151 mmol) after purification by column chromatography on silica gel (SiliaFlash; eluent: hexane: Et_2O = 95: 5) and preparative GPC.

^{1}H NMR (400 MHz, $CDCl_3$) δ 7.52 (dt, J = 7.1, 1.4 Hz, 1H), 7.39 (tq, J = 7.8, 0.9 Hz, 1H), 7.31 (d, J = 14.2 Hz, 1H), 7.21 (tq, J = 7.7, 1.2 Hz, 1H), 7.09 (d, J = 7.5 Hz, 1H), 7.05 (d, J = 8.0 Hz, 1H), 7.00 (d, J = 8.3 Hz, 1H), 6.96 (td, J = 7.3, 0.8 Hz, 1H), 6.92 (tq, J = 7.4, 1.0 Hz, 1H), 6.19 (d, J = 14.2 Hz, 1H), 3.65 (s, 2H), 0.64 (s, 3H), 0.24 (s, 9H).

^{13}C NMR (101 MHz, $CDCl_3$) δ 165.1, 154.0, 145.6, 135.0, 131.9, 130.7, 129.5, 124.7, 124.5, 122.8, 120.7, 120.6, 119.5, 109.4, 61.0, 1.5, -2.7.

HRMS (EI) m/z: [M]+ Calcd. for $C_{19}H_{24}O_2Si_2$, 340.5690; Found, 340.1311.

2-(*sec*-butoxy)-2-phenyl-2*H*-benzo[1,2]oxasiline (2aI)

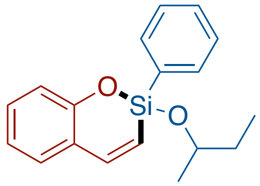

According to the **General Procedure**, a mixture of **1a** (21.0 mg, 0.18 mmol), **I** (52.3 mg, 0.20mmol, 1.1 equiv) and 130 µL of Ir catalyst stock solution with benzene (containing $[IrCl(cod)]_2$ (3.1 mg, 0.0046 mmol, 2 mol%), (*S*)-DTBM-SEGPHOS (9.9 mg, 0.0083 mmol, 4 mol%) and benzene (0.2 mL)) in benzene (270 µL, in total 400 µL, 0.5 M) was heated at 80 °C for 12 h. **2aI** was obtained as a colorless oil in 29% yield (17.6 mg, 0.052 mmol, d.r. = 1 : 1) after purification by column chromatography on silica gel (DIOL-silica; eluent: hexane: Et_2O = 95: 5) and preparative GPC. Low yield because of decomposition during purification, while NMR yield was 60%.

^{1}H NMR (400 MHz, $CDCl_3$) δ 7.69 (dd, J = 7.8, 1.4 Hz, 2H), 7.57 (d, J = 14.2 Hz, 1H), 7.44 (tt, J = 7.2, 2.1 Hz, 1H), 7.38 (td, J = 7.0, 1.5 Hz, 2H), 7.23 (tt, J = 7.6, 1.8 Hz, 2H), 7.03 (dd, J = 8.1, 3.8 Hz, 1H), 6.98 (tt, J = 7.4, 1.3 Hz, 1H), 6.06 (d, J = 14.2 Hz, 1H), 3.97-3.89 (sextet, J = 5.6 Hz 1H), 1.60-1.40 (m, 2H), 1.17 (d, J = 8.9 Hz, 3H), 0.89 (t, J = 7.4 Hz, 3H).

^{13}C NMR (101 MHz, $CDCl_3$) δ 154.1, 154.0, 149.1, 149.0, 134.5, 133.4, 133.4, 130.7, 129.7, 128.0, 124.2, 124.2, 121.1, 121.1, 120.0, 119.9, 119.5, 71.3, 71.3, 32.1, 32.0, 23.4, 23.4, 10.2, 10.1.

HRMS (EI) m/z: [M]+ Calcd. for $C_{18}H_{20}O_2Si$, 296.1233; Found, 296.1233.

2,2-diphenyl-2*H*-benzo[1,2]oxasiline (2aJ)

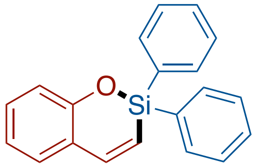

According to the **General Procedure**, a mixture of **1a** (141.4 mg, 1.2 mmol), **J** (318.7 mg, 1.23 mmol, 1.1 equiv), $[IrCl(cod)]_2$ (9.48 mg, 0.014 mmol, 1 mol%) and (*S*)-DTBM-SEGPHOS (35.5 mg, 0.03 mmol, 2 mol%) in benzene (1 mL, 1.2 M) was heated at 80 °C for 12 h. **2aJ** was obtained as a white solid in 78% yield (281.6 mg, 0.94 mmol) after purification by column chromatography on silica gel (SiliaFlash; eluent: hexane: Et_2O = 95: 5). Recrystallization was performed in hexane and the structure of **2aJ** was also checked by scXRD as mentioned below.

^{1}H NMR (400 MHz, $CDCl_3$) *δ*. 7.65 (tt, J = 5.0, 1.8 Hz, 4H), 7.54 (d, J = 14.2 Hz, 1H), 7.46 (tt, J = 7.2, 2.0 Hz, 2H), 7.42-7.38 (m, 4H), 7.23-7.16 (m, 2H), 7.02 (d, J = 7.8 Hz, 1H), 6.95 (td, J = 7.4, 1.2 Hz, 1H), 6.23 (d, J = 14.2 Hz, 1H)

^{13}C NMR (101 MHz, $CDCl_3$) *δ* 153.6, 147.5, 134.7, 134.5, 130.9, 130.6, 129.8, 128.1, 124.3, 121.1, 119.7, 119.7

HRMS (EI) m/z: [M]+ Calcd. for $C_{20}H_{16}OSi$, 300.0970; Found, 300.0969.

spiro[benzo[1,2]oxasiline-2,5'-dibenzo[*b*,*d*]silole] (2aK)

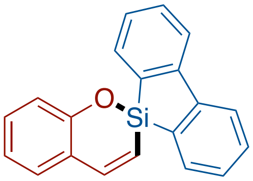

According to the **General Procedure**, a mixture of **1a** (20.5 mg, 0.17 mmol, 1.7 equiv), **K** (26.2 mg, 0.1 mmol), $[IrCl(cod)]_2$ (2.5 mg, 0.0037 mmol, 2 mol%) and (*S*)-DTBM-SEGPHOS (8.8 mg, 0.0074 mmol, 4 mol%) in toluene (0.4 mL) was heated at 80 °C for 12 h gave **2aK** as a white solid in 73% yield (21.9 mg, 0.073 mmol) after purification by column chromatography on silica gel (SiliaFlash; eluent: hexane: Et_2O = 95: 5) and preparative GPC.

^{1}H NMR (400 MHz, $CDCl_3$) *δ* 7.81 (d, J = 7.8 Hz, 2H), 7.72 (d, J = 14.2 Hz, 1H), 7.62 (dt, J = 7.2, 1.0 Hz, 2H), 7.49 (td, J = 7.5, 1.4 Hz, 2H), 7.31-7.22 (m, 5H), 7.03 (td, J = 7.5, 1.2 Hz, 1H), 6.97 (d, J = 8.2 Hz, 1H), 5.95 (d, J = 14.2 Hz, 1H).

^{13}C NMR (101 MHz, $CDCl_3$) *δ* 154.0, 150.5, 147.5, 134.1, 134.0, 132.2, 131.1, 129.9, 128.3, 124.0, 121.4, 120.9, 120.0, 118.1

HRMS (EI) m/z: [M]+ Calcd. for $C_{20}H_{14}OSi$, 298.0814; Found, 298.0811.

spiro[benzo[1,2]oxasiline-2,9'-tribenzo[*b*,*d*,*f*]silepine] (2aL)

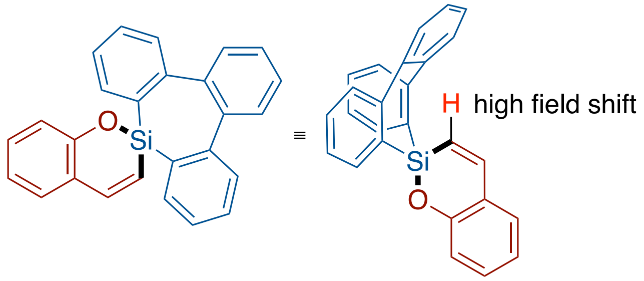

According to the **General Procedure**, a mixture of **1a** (7.9 mg, 0.067 mmol), **L** (26.7 mg, 0.079 mmol, 1.2 equiv),
$[IrCl(cod)]_2$ (1.31 mg, 0.002 mmol, 2 mol%) and (*S*)-DTBM-SEGPHOS (4.97 mg, 0.004 mmol, 4 mol%) in toluene (0.2 mL) was heated at 110 °C for 36 h. **2aL** was obtained as a white solid in 58% yield (20 mg, 0.053 mmol) after purification by column chromatography on silica gel (SiliaFlash; eluent: hexane: Et_2O = 95: 5).
Note that a single diastereomer was obtained even though a mixture of two diastereomeric **L** was used. The stereochemistry of **2aL** is not fully determined but estimated as above, because unusual high field shift of 3-H (5.14 ppm) was observed because of the terphenyl benzene ring.
^{1}H NMR (400 MHz, $CDCl_3$) *δ*. 7.69-7.71 (dt, *J* = 7.3, 0.9 Hz, 2H), 7.49 (s, 4H), 7.42-7.44 (m, 4H), 7.31-7.34 (m, 4H), 7.30-7.31 (d, *J* = 3.7 Hz, 2H), 7.142, 7.107 (d, *J* = 14.2 Hz, 1H), 7.063-7.082 (dt, *J* = 7.3, 1.1 Hz, 1H), 6.951-6.991 (dt, *J* = 8.1, 3.8 Hz, 1H), 5.141 (d, *J* = 14.2 Hz, 1H).
^{13}C NMR (101 MHz, $CDCl_3$) *δ* 153.9, 146.7, 144.7, 140.1, 139.8, 132.5, 131.0, 130.5, 130.2, 129.9, 129.8, 127.8, 126.7, 124.6, 121.4, 119.6, 119.0.
HRMS (EI) m/z: [M]+ Calcd. for $C_{26}H_{18}OSi$, 374.1127; Found, 374.1123.

2-(3,5-di-*tert*-butyl-4-methoxyphenyl)-2-phenyl-2*H*-benzo[1,2]oxasiline (2aM)

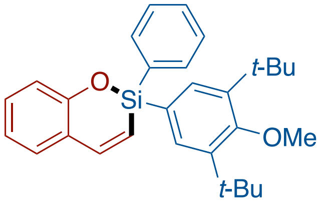

According to the **General Procedure**, a mixture of **1a** (9.8 mg, 0.083 mmol, 1.1 equiv), **M** (29.2 mg, 0.073 mmol), $[IrCl(cod)]_2$ (1.35 mg, 0.002 mmol, 2 mol%) and (*S*)-DTBM-SEGPHOS (4.98 mg, 0.004 mmol, 4 mol%) in benzene (0.2 mL) was heated at 80 °C for 12 h. **2aM** was obtained as a white solid in 67% yield (21.5 mg, 0.049 mmol) after purification by column chromatography on silica gel (SiliaFlash; eluent: hexane: Et_2O = 95: 5).
^{1}H NMR (400 MHz, $CDCl_3$) *δ* 7.65 (dd, J = 7.8, 1.8 Hz, 2H), 7.54-7.50 (m, 3H), 7.47-7.43 (m, 1H), 7.42-7.38 (m, 2H), 7.22-7.16 (m, 2H), 7.02 (d, J = 7.8 Hz, 1H), 6.94 (td, J = 7.4, 1.1 Hz, 1H), 6.22 (d, J = 14.6 Hz, 1H), 3.69 (s, 3H), 1.39 (s, 18H)
^{13}C NMR (101 MHz, $CDCl_3$) *δ* 162.0, 153.7, 147.2, 143.4, 135.0, 134.7, 133.4, 130.8, 130.4, 129.7, 128.0, 127.5, 124.4, 121.0, 120.4, 119.7, 64.3, 35.9, 32.1.
HRMS (EI) m/z: [M]+ Calcd. for $C_{29}H_{34}O_2Si$, 442.2328; Found, 442.2326.

6-(*tert*-butyl)-2,2-dimethyl-2*H*-benzo[1,2]oxasiline (2bA)

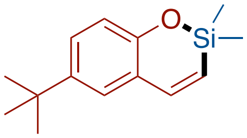

According to the **General Procedure**, a mixture of **1b** (36.53 mg, 0.21 mmol), **A** (28.25 mg, 0.21 mmol, 1.0 equiv), $[IrCl(cod)]_2$ (2.3 mg, 0.003 mmol, 2 mol%) and (*S*)-DTBM-SEGPHOS (6.9 mg, 0.005 mmol, 4 mol%) in benzene (0.2 mL) was heated at 80 °C for 12 h. **2bA** was obtained as a colorless oil in 85% yield (41.4 mg, 0.178 mmol) after purification by kügelrhor distillation.

^{1}H NMR (400 MHz, $CDCl_3$) δ 7.26 (d, J = 14.6 Hz, 1H), 7.19 (dd, J = 8.5, 2.5 Hz, 1H), 7.07 (d, J = 5.9 Hz, 1H), 6.82 (d, J = 8.7 Hz, 1H), 5.90 (d, J = 14.2 Hz, 1H), 1.30 (s, 9H), 0.34 (s, 6H)

^{13}C NMR (101 MHz, $CDCl_3$) δ 151.4, 145.9, 143.3, 127.4, 126.5, 123.6, 122.9, 118.8, 34.1, 31.6, 1.6

HRMS (EI) m/z: [M]+ Calcd. for $C_{14}H_{20}OSi$, 232.1283; Found, 232.1280.

2,2-dimethyl-6-(trimethylsilyl)-2*H*-benzo[1,2]oxasiline (2cA)

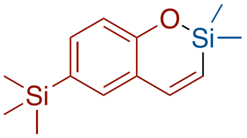

According to the **General Procedure**, a mixture of **1c** (37.9 mg, 0.20 mmol), **A4** (26.5 mg, 0.20 mmol, 1.0 equiv) and 400 µL of Ir catalyst stock solution ($[IrCl(cod)]_2$ (3.0 mg, 0.0044 mmol, 2 mol%), (*S*)-DTBM-SEGPHOS (9.7 mg, 0.0082 mmol, 4 mol%) and benzene (400 µL, 0.5M)) was heated at 80 °C for 12 h. **2cA** was obtained as a colorless oil in 53% yield (26.0 mg, 0.105 mmol) after purification by kügelrhor distillation.

^{1}H NMR (400 MHz, $CDCl_3$) δ 7.33-7.27 (m, 2H), 7.21 (d, J = 1.4 Hz, 1H), 6.89 (d, J = 7.8 Hz, 1H), 5.92 (d, J = 14.6 Hz, 1H), 0.35 (s, 6H), 0.25 (s, 9H).

^{13}C NMR (101 MHz, $CDCl_3$) δ 154.5, 145.6, 136.0, 134.6, 131.5, 124.0, 123.1, 119.0, 1.6, -0.9.

HRMS (EI) m/z: [M]+ Calcd. for $C_{13}H_{20}OSi_2$, 248.1053; Found, 248.1048.

6-bromo-2,2-dimethyl-2*H*-benzo[1,2]oxasiline (2dA)

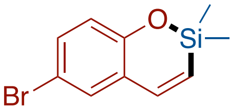

According to the **General Procedure**, a mixture of **1d** (30.6 mg, 0.156 mmol), **A4** (22.1 mg, 0.163 mmol, 1.1 equiv), $[IrCl(cod)]_2$ (3.0 mg, 0.0044 mmol, 2 mol%) and (*S*)-DTBM-SEGPHOS (12.6 mg, 0.01 mmol, 6 mol%) in benzene (0.2 mL) was heated at 80 °C for 12 h. **2dA** was obtained as a colorless oil in 20% yield (8.0 mg, 0.031 mmol) after purification by column chromatography on silica gel (SiliaFlash; eluent: hexane: Et_2O = 95: 5) and preparative GPC.

^{1}H NMR (400 MHz, $CDCl_3$) δ 7.22 (dd, J = 8.5, 2.5 Hz, 1H), 7.18 (d, J = 2.3 Hz, 1H), 7.15 (d, J = 14.2 Hz, 1H), 6.75 (d, J = 8.7 Hz, 1H), 5.97 (d, J = 14.6 Hz, 1H), 0.35 (s, 6H).

^{13}C NMR (101 MHz, $CDCl_3$) δ 152.8, 144.1, 132.8, 131.9, 126.2, 124.9, 121.2, 112.5, 1.4.

HRMS (EI) m/z: [M]+ Calcd. for $C_{10}H_{11}OBrSi$, 253.9763; Found, 253.9762.

2,2-dimethyl-6-(acetyl)-2*H*-benzo[1,2]oxasiline (2eA)

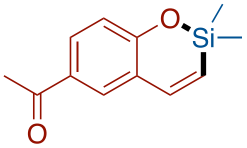

According to the **General Procedure**, a mixture of **1e** (20.6 mg, 0.129 mmol), **A4** (24.7 mg, 0.180 mmol, 1.3 equiv), $[IrCl(cod)]_2$ (1.6 mg, 0.0023 mmol, 2 mol%) and (*S*)-DTBM-SEGPHOS (5.3 mg, 0.0045 mmol, 4 mol%) in benzene (0.2 mL) was heated at 80 °C for 12 h. **2eA** was obtained as a white solid in 44% yield (12.2 mg, 0.0559 mmol) after purification by kügelrhor distillation.

^{1}H NMR (400 MHz, $CDCl_3$) δ 7.78 (dd, J = 8.2, 2.3 Hz, 1H), 7.73 (d, J = 2.3 Hz, 1H), 7.29 (d, J = 14.2 Hz, 1H), 6.90 (d, J = 8.2 Hz, 1H), 5.99 (d, J = 14.6 Hz, 1H), 2.55 (s, 3H), 0.38 (s, 6H).

^{13}C NMR (101 MHz, $CDCl_3$) δ 196.9, 158.1, 144.9, 131.5, 130.4, 130.1, 124.3, 124.2, 119.6, 26.5, 1.5.

HRMS (EI) m/z: [M]+ Calcd. for $C_{12}H_{14}O_2Si$, 218.0763; Found, 218.0756.

6-methoxy-2,2-dimethyl-2*H*-benzo[1,2]oxasiline (2fA)

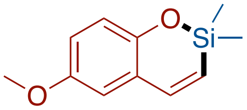

According to the **General Procedure**, a mixture of **1f** (37.4 mg, 0.253 mmol), **A4** (47.12 mg, 0.346 mmol, 1.3 equiv), $[IrCl(cod)]_2$ (4.28 mg, 0.0063 mmol, 2 mol%) and (*S*)-DTBM-SEGPHOS (10.04 mg, 0.0102 mmol, 4 mol%) in benzene (0.4 mL) was heated at 80 °C for 12 h. **2fA** was obtained as a colorless oil in 78% yield (40.7 mg, 0.198 mmol) after purification by kügelrhor distillation and preparative GPC.

^{1}H NMR (400 MHz, $CDCl_3$) δ 7.20 (d, J = 14.2 Hz, 1H), 6.81 (d, J = 8.7 Hz, 1H), 6.73 (dd, J = 8.9, 3.0 Hz, 1H), 6.62 (d, J = 2.7 Hz, 1H), 5.94 (d, J = 14.2 Hz, 1H), 3.77 (s, 3H), 0.33 (s, 6H).

^{13}C NMR (101 MHz, $CDCl_3$) δ 153.5, 147.7, 145.2, 124.7, 124.2, 119.8, 115.1, 115.0, 55.9, 1.4.

HRMS (EI) m/z: [M]+ Calcd. for $C_{11}H_{14}O_2Si$, 206.0763; Found, 206.0759.

2,2-dimethyl-6-(4,4,5,5-tetramethyl-1,3,2-dioxaborolan-2-yl)-2*H*-benzo[1,2]oxasiline (2gA)

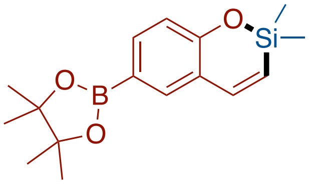

According to the **General Procedure**, a mixture of **1g** (7.87 mg, 0.067 mmol), **A4** (26.70 mg, 0.079 mmol, 1.2 equiv), $[IrCl(cod)]_2$ (1.31 mg, 0.002 mmol, 2 mol%) and (*S*)-DTBM-SEGPHOS (4.97 mg, 0.004 mmol, 4 mol%) in toluene (0.2 mL) was heated at 80 °C for 12 h. **2gA** was obtained as a white solid in 58% yield (11.7 mg) after purification by column chromatography on silica gel (DIOL-silica; eluent: hexane only).

^{1}H NMR (400 MHz, $CDCl_3$) δ 7.60 (dd, J = 8.0, 1.6 Hz, 1H), 7.54 (d, J = 1.1 Hz, 1H), 7.27 (d, J = 14.2 Hz, 1H), 6.86 (d, J = 8.2 Hz, 1H), 5.88 (d, J = 14.6 Hz, 1H), 1.32 (s, 12H), 0.34 (s, 6H).

^{13}C NMR (101 MHz, $CDCl_3$) δ 156.6, 145.5, 137.7, 136.3, 124.0, 122.9, 119.0, 83.7, 24.9, 1.5.

HRMS (EI) m/z: [M]+ Calcd. for $C_{13}H_{20}OSi_2$, 248.1053; Found, 248.1048.

2,2-dimethyl-7-(4,4,5,5-tetramethyl-1,3,2-dioxaborolan-2-yl)-2*H*-benzo[1,2]oxasiline (2hA)

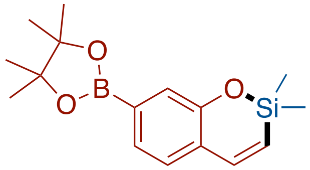

According to the **General Procedure**, a mixture of **1h** (7.87 mg, 0.067 mmol), **A4** (26.70 mg, 0.079 mmol, 1.2 equiv), $[IrCl(cod)]_2$ (1.31 mg, 0.002 mmol, 2 mol%) and (*S*)-DTBM-SEGPHOS (4.97 mg, 0.004 mmol, 4 mol%) in toluene (0.2 mL) was heated at 80 °C for 12 h. **2hA** was obtained as a white solid in 41% yield (14.2 mg) after purification by column chromatography on silica gel (DIOL-silica; eluent: hexane only).

^{1}H NMR (400 MHz, $CDCl_3$) δ 7.33-7.31 (m, 2H), 7.25 (d, J = 14.2 Hz, 1H), 7.07 (d, J = 7.3 Hz, 1H), 5.97 (d, J = 14.2 Hz, 1H), 1.33 (s, 12H), 0.33 (s, 6H).

^{13}C NMR (101 MHz, $CDCl_3$) δ 153.0, 145.3, 130.0, 126.9, 126.9, 125.5, 124.9, 83.9, 24.9, 1.5.

HRMS (EI) m/z: [M]+ Calcd. for $C_{13}H_{20}OSi_2$, 248.1053; Found, 248.1048.

2,2-dimethyl-8-(4,4,5,5-tetramethyl-1,3,2-dioxaborolan-2-yl)-2*H*-benzo[1,2]oxasiline (2iA)

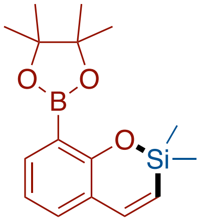

According to the **General Procedure**, a mixture of **1i** (28.1 mg, 0.115 mmol), **A4** (16.4 mg, 0.121 mmol, 1.1 equiv), $[IrCl(cod)]_2$ (2.9 mg, 0.004 mmol, 4 mol%) and (*S*)-DTBM-SEGPHOS (10.3 mg, 0.008 mmol, 8 mol%) in benzene (0.4 mL) was heated at 80 °C for 12 h. **2iA** was obtained as a colorless oil in 51% yield (17.7 mg) after purification by column chromatography on silica gel (DIOL-silica; eluent: hexane only).

^{1}H NMR (400 MHz, $CDCl_3$) δ 7.45 (dd, J = 7.1, 1.6 Hz, 1H), 7.22 (d, J = 14.6 Hz, 1H), 7.12 (dd, J = 7.3, 1.8 Hz, 1H), 6.87 (t, J = 7.3 Hz, 1H), 5.89 (d, J = 14.2 Hz, 1H), 1.34 (s, 12H), 0.33 (s, 6H).

^{13}C NMR (101 MHz, $CDCl_3$) δ 158.2, 145.6, 135.5, 133.2, 124.2, 123.3, 120.2, 83.4, 24.9, 1.4.

HRMS (EI) m/z: [M]+ Calcd. for $C_{13}H_{20}OSi_2$, 248.1053; Found, 248.1048.

4-methyl-2,2-diphenyl-2*H*-benzo[1,2]oxasiline (2jJ)

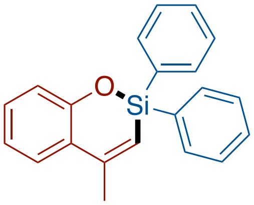

According to the **General Procedure**, a mixture of **1j** (24.9 mg, 0.189 mmol), **J** (59.9 mg, 0.23 mmol, 1.2 equiv) and 130 µL of Ir catalyst stock solution with benzene ($[IrCl(cod)]_2$ (3.1 mg, 0.0046 mmol, 2 mol%), (*S*)-**L1** (9.9 mg, 0.008q3 mmol, 4 mol%) and benzene (0.2 mL)) in benzene (270 µL, in total 400 µL, 0.5 M) was heated at 80 °C for 12 h. **2jJ** was obtained as a white solid in 45% yield (26.7 mg, 0.085 mmol) after purification by column chromatography on silica gel (eluent: hexane: Et_2O = 95: 5) and preparative GPC.

^{1}H NMR (400 MHz, $CDCl_3$) δ 7.65-7.62 (m, 4H), 7.48-7.37 (m, 7H), 7.22 (t, J = 7.5 Hz, 1H), 7.05 (dt, J = 8.1, 1.3 Hz, 1H), 6.99-6.95 (m, 1H), 6.05 (d, J = 0.9 Hz, 1H), 2.40 (s, 3H).

^{13}C NMR (101 MHz, CDCl$_3$) δ 153.8, 152.5, 134.8, 134.7, 130.5, 129.8, 128.1, 126.9, 125.3, 120.9, 120.2, 118.0, 26.0.
HRMS (EI) m/z: [M]+ Calcd. for $C_{21}H_{18}OSi$, 314.1127; Found, 314.1126.

4-benzyl-2,2-diphenyl-2*H*-benzo[1,2]oxasiline (2kJ)

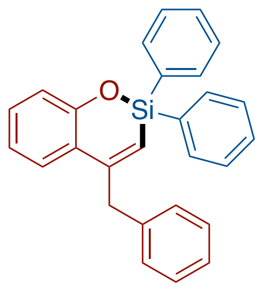

According to the **General Procedure**, a mixture of **1k** (45.2 mg, 0.217 mmol), **J** (56.7 mg, 0.218 mmol, 1.0 equiv) and 400 μL of Ir catalyst stock solution ([IrCl(cod)]$_2$ (3.0 mg, 0.0044 mmol, 2 mol%), (*S*)-DTBM-SEGPHOS (9.7 mg, 0.0082 mmol, 4 mol%) in benzene (400 μL, 0.5M)) was heated at 80 °C for 12 h. **2kJ** was obtained as a white solid in 25% yield (21.3 mg, 0.054 mmol) after purification by column chromatography on silica gel (SiliaFlash; eluent: hexane: Et$_2$O = 95: 5) and preparative GPC.
^{1}H NMR (400 MHz, CDCl$_3$) δ 7.63 (dt, J = 6.3, 1.7 Hz, 4H), 7.50 (dd, J = 8.0, 1.6 Hz, 1H), 7.46 (tt, J = 7.3, 2.1 Hz, 2H), 7.40 (tt, J = 6.9, 1.5 Hz, 4H), 7.35-7.28 (m, 4H), 7.25-7.18 (m, 2H), 7.07 (dd, J = 8.2, 1.4 Hz, 1H), 6.91 (td, J = 7.5, 1.2 Hz, 1H), 5.95 (s, 1H), 4.08 (s, 2H)
^{13}C NMR (101 MHz, CDCl$_3$) δ 154.6, 154.0, 139.0, 134.8, 134.5, 130.6, 129.8, 129.2, 128.7, 128.1, 127.0, 126.4, 124.5, 120.9, 120.4, 119.5, 44.1.
HRMS (EI) m/z: [M]+ Calcd. for $C_{27}H_{22}OSi$, 390.1440; Found, 390.1439.

8-(*tert*-butyl)-2,2,6-trimethyl-2*H*-benzo[1,2]oxasiline (2lA)

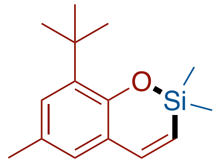

According to the **General Procedure**, a mixture of **1l** (37.7 mg, 0.153 mmol), **A4** (28.3 mg, 0.20 mmol, 1.3 equiv) and 400 μL of Ir catalyst stock solution (containing [IrCl(cod)]$_2$ (3.0 mg, 0.0044 mmol, 2 mol%), (*S*)-DTBM-SEGPHOS (9.7 mg, 0.0082 mmol, 4 mol%) and benzene (400 μL, 0.5M)) was heated at 80 °C for 12 h. **2lA** was obtained as a colorless oil in 79% yield (29.7 mg, 0.121 mmol) after purification by column chromatography on silica gel (SiliaFlash; eluent: hexane: Et$_2$O = 95: 5) and preparative GPC.
^{1}H NMR (400 MHz, CDCl$_3$) δ 7.24 (d, J = 14.6 Hz, 1H), 7.02 (d, J = 1.8 Hz, 1H), 6.78 (d, J = 2.3 Hz, 1H), 5.89 (d, J = 14.6 Hz, 1H), 2.28 (s, 3H), 1.40 (s, 9H), 0.36 (s, 6H)
^{13}C NMR (101 MHz, CDCl$_3$) δ 150.2, 146.4, 138.7, 129.5, 128.8, 127.8, 124.4, 122.4, 34.8, 29.8, 20.8, 1.4
HRMS (EI) m/z: [M]+ Calcd. for $C_{15}H_{22}OSi$, 246.1440; Found, 246.1438.

6,6'-(ethene-1,1-diyl)bis(2,2-dimethyl-2*H*-benzo[1,2]oxasiline) (2mA)

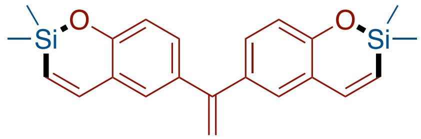

According to the **General Procedure**, a mixture of **1m** (11.63 mg, 0.045 mmol), **A4** (22.2 mg, 0.163 mmol, 3.6 equiv), $[IrCl(cod)]_2$ (1.7 mg, 0.0025 mmol, 6 mol%) and (*S*)-DTBM-SEGPHOS (6.13 mg, 0.005 mmol, 12 mol%) in benzene (0.2 mL) was heated at 80 °C for 12 h. **2mA** was obtained as a white solid in 76% yield (12.7 mg, 0.034 mmol) after purification by column chromatography on silica gel (SiliaFlash; eluent: hexane: Et_2O = 95: 5) and preparative GPC.

^{1}H NMR (400 MHz, $CDCl_3$) δ 7.21 (d, J = 14.2 Hz, 2H), 7.15 (dd, J = 8.2, 2.3 Hz, 2H), 7.06 (d, J = 2.3 Hz, 2H), 6.83 (d, J = 8.2 Hz, 2H), 5.91 (d, J = 14.6 Hz, 2H), 5.28 (s, 2H), 0.36 (s, 12H)

^{13}C NMR (101 MHz, $CDCl_3$) δ 153.5, 148.8, 145.5, 134.3, 130.5, 129.4, 124.0, 123.4, 119.1, 111.8, 1.6.

HRMS (EI) m/z: [M]+ Calcd. for $C_{22}H_{24}O_2Si_2$, 376.1315; Found, 376.1317.

2,2-diphenyl-2*H*-1,2-oxasiline (4aJ)

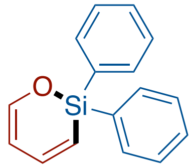

According to the **General Procedure**, a mixture of furan **3a** (31.0 mg, 0.45 mmol, 1.7 equiv), **J** (68.8 mg, 0.26 mmol) and 400 µL of Ir catalyst stock solution (containing $[IrCl(cod)]_2$ (3.0 mg, 0.0044 mmol, 2 mol%), (*S*)-DTBM-SEGPHOS (9.7 mg, 0.0082 mmol, 4 mol%) and benzene (400 µL, 0.5M)) was heated at 80 °C for 12 h. **4aJ** was obtained as a white solid in 62% yield (39.7 mg, 0.16 mmol) after purification by column chromatography on silica gel (DIOL-silica; eluent: hexane only) and preparative GPC.

^{1}H NMR (400 MHz, $CDCl_3$) δ 7.64 (td, J = 3.9, 2.1 Hz, 4H), 7.50-7.41 (m, 6H), 7.02 (ddd, J = 14.3, 6.1, 1.3 Hz, 1H), 6.77 (td, J = 3.4, 1.8 Hz, 1H), 5.98 (d, J = 14.2 Hz, 1H), 5.34-5.31 (t, J = 6.0 Hz, 1H).

^{13}C NMR (101 MHz, $CDCl_3$) δ 146.1, 143.2, 134.9, 134.6, 130.6, 128.1, 116.2, 105.4.

HRMS (EI) m/z: [M]+ Calcd. for $C_{16}H_{14}OSi$, 250.0814; Found, 250.0812.

6-methyl-2,2-diphenyl-2*H*-1,2-oxasiline (4bJ)

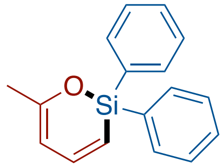

According to the **General Procedure**, a mixture of **3b** (32.9 mg, 0.40 mmol, 1.6 equiv.), **J** (64.5 mg, 0.248 mmol), $[IrCl(cod)]_2$ (2.26 mg, 0.0034 mmol, 1.4 mol%) and (*S*)-DTBM-SEGPHOS (8.74 mg, 0.0074 mmol, 0.3 mol%) in benzene (0.4 mL) was heated at 80 °C for 12 h. **4bJ** was obtained as a white solid in 99% yield (65.2 mg, 0.247 mmol) after purification by kügelrhor distillation.

^{1}H NMR (400 MHz, $CDCl_3$) δ 7.62 (td, J = 4.0, 1.9 Hz, 4H), 7.48-7.40 (m, 6H), 6.98 (dd, J = 14.2, 6.4 Hz, 1H), 5.79 (d, J = 14.2 Hz, 1H), 5.20 (d, J = 6.4 Hz, 1H), 1.98 (s, 3H).

^{13}C NMR (101 MHz, $CDCl_3$) δ 155.3, 144.3, 135.4, 134.6, 130.4, 128.1, 112.1, 101.9, 22.7.

HRMS (EI) m/z: [M]+ Calcd. for $C_{17}H_{16}OSi$,264.0970 Found, 264.0968.

6-carboxymethyl 2,2-diphenyl-2*H*-1,2-oxasiline (4cJ)

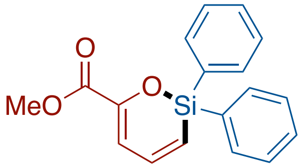

According to the **General Procedure**, a mixture of **3c** (40.58 mg, 0.322 mmol), **J** (89.8 mg, 0.345 mmol, 1.07 equiv.) $[IrCl(cod)]_2$ (4.3 mg, 0.0064 mmol, 2.0 mol%) and (*S*)-DTBM-SEGPHOS (12.45 mg, 0.011 mmol, 0.34 mol%) in benzene (0.6 mL) was heated at 80 °C for 12 h. **4cJ** was obtained as a white solid in 64% yield (63.2 mg, 0.205 mmol) after purification by kügelrhor distillation.
^{1}H NMR (400 MHz, $CDCl_3$) δ 7.62 (dt, J = 6.3, 1.7 Hz, 4H), 7.48 (tt, J = 7.3, 2.1 Hz, 2H), 7.42 (tt, J = 6.9, 1.4 Hz, 4H), 7.12 (q, J = 6.9 Hz, 1H), 6.55 (dd, J = 6.4, 0.9 Hz, 1H), 6.31 (d, J = 14.6 Hz, 1H), 3.81 (s, 3H)
^{13}C NMR (101 MHz, $CDCl_3$) δ 164.3, 143.4, 142.2, 134.8, 133.8, 130.9, 128.2, 123.5, 110.9, 52.5
HRMS (EI) m/z: [M]+ Calcd. for $C_{18}H_{16}O_3Si$, 308.0869; Found, 308.0862.

5-carboxymethyl 6-methyl 2,2-diphenyl-2*H*-1,2-oxasiline (4dJ)

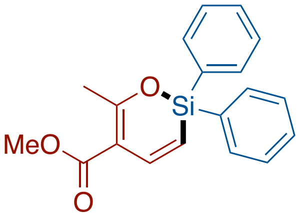

According to the **General Procedure**, a mixture of **3d** (40.2 mg, 0.287 mmol), **J** (83.0 mg, 0.319 mmol, 1.1 equiv), $[IrCl(cod)]_2$ (4.1 mg, 0.006 mmol, 2 mol%) and (*S*)-DTBM-SEGPHOS (12.2 mg, 0.010 mmol, 4 mol%) in benzene (0.4 mL) was heated at 80 °C for 12 h. **4dJ** was obtained as a white solid in 52% yield (47.9 mg, 0.148 mmol) after purification by column chromatography on silica gel (DIOL-silica; eluent: hexane only).
^{1}H NMR (400 MHz, $CDCl_3$) δ 7.66 (d, J = 15.1 Hz, 1H), 7.61 (dt, J = 6.1, 1.6 Hz, 4H), 7.49 (tt, J = 7.2, 2.0 Hz, 2H), 7.45-7.41 (m, 4H), 5.91 (d, J = 14.6 Hz, 1H), 3.78 (s, 3H), 2.47 (s, 3H).
^{13}C NMR (101 MHz, $CDCl_3$) δ 168.4, 167.9, 144.8, 134.7, 133.9, 130.9, 128.2, 112.0, 108.5, 51.6, 23.5.
HRMS (EI) m/z: [M]+ Calcd. for $C_{18}H_{16}O_3Si$, 308.0869; Found, 308.0862.

(2,2-dimethyl-2*H*-1,2-oxasilin-6-yl)di(furan-2-yl)phosphine oxide (4eA)

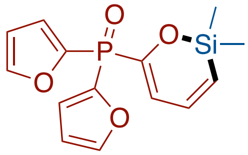

According to the **General Procedure**, a mixture of **3e** (49.8 mg, 0.2 mmol, 1.7 equiv), **A4** (43.3 mg, 0.32 mmol, 1.6 equiv) and 400 µL of Ir catalyst stock solution (containing $[IrCl(cod)]_2$ (3.0 mg, 0.0044 mmol, 2 mol%), (*S*)-DTBM-SEGPHOS (9.7 mg, 0.0082 mmol, 4 mol%) in benzene (0.4 mL) and AcOEt (0.2 mL, to dissolve substrate) was heated at 110 °C for 12 h. **4eA** was obtained as a white solid in 6% yield (3.8 mg, 0.012 mmol) after purification by column chromatography on silica gel (SiliaFlash; eluent: AcOEt = 100%) and preparative GPC.
^{1}H NMR (400 MHz, $CDCl_3$) δ 7.72 (q, J = 1.7 Hz, 2H), 7.16-7.14 (m, 2H), 6.81 (qd, J = 6.9, 3.7 Hz, 1H), 6.54-6.53 (m, 2H), 6.27 (ddd, J = 8.9, 6.2, 0.9 Hz, 1H), 5.93 (dq, J = 14.2, 1.4 Hz, 1H), 0.27 (s, 6H).
^{13}C NMR (101 MHz, $CDCl_3$) δ 148.6, 148.5, 147.0, 146.9, 145.4, 139.7, 139.6, 125.8, 125.8, 123.2, 123.0, 116.2, 115.9, 111.0, 110.9, 1.4.

^{31}P NMR (162 MHz, CDCl$_3$) δ -3.1
HRMS (EI) m/z: [M]+ Calcd. for $C_{20}H_{16}O_2Si$, 316.0920; Found, 316.0919.

6,6'-(ethane-1,1-diyl)bis(2,2-diphenyl-2*H*-1,2-oxasiline) (4fJ)

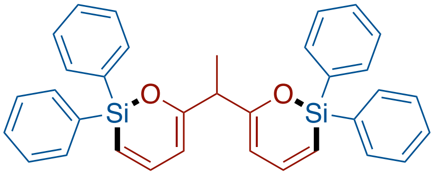

According to the **General Procedure**, a mixture of **3f** (31.3 mg, 0.193 mmol), **J** (59.5 mg, 0.229 mmol, 1.2 equiv), [IrCl(cod)]$_2$ (2.8 mg, 0.004 mmol, 2 mol%) and (*S*)-DTBM-SEGPHOS (7.9 mg, 0.067 mmol, 4 mol%) in benzene (0.4 mL) wass heated at 80 °C for 12 h. **4fJ** was obtained as a white solid in 24% yield (24.6 mg, 0.0468 mmol) after purification by column chromatography on silica gel (DIOL-silica; eluent: hexane only) and preparative GPC.
^{1}H NMR (400 MHz, CDCl$_3$) δ 7.56-7.53 (m, 8H), 7.40 (tt, J = 7.2, 1.8 Hz, 4H), 7.35-7.31 (m, 8H), 6.93 (q, J = 6.9 Hz, 2H), 5.80 (d, J = 14.2 Hz, 2H), 5.28 (d, J = 6.9 Hz, 2H), 3.15 (q, J = 7.0 Hz, 1H), 1.32 (d, J = 7.3 Hz, 3H).
^{13}C NMR (101 MHz, CDCl$_3$) δ 158.2, 144.0, 135.3, 134.6, 134.5, 130.3, 130.2, 128.0, 113.4, 101.5, 46.6, 15.7.
HRMS (EI) m/z: [M]+ Calcd. for $C_{34}H_{30}O_2Si_2$, 526.1784; Found, 526.1780.

2,2-diphenyl-2*H*-benzo[*f*][1,5,2]dioxasilepine (6J)

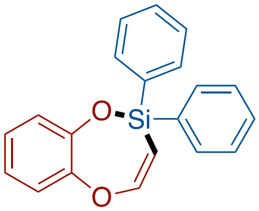

According to the **General Procedure**, a mixture of **5** (24.8 mg, 0.185 mmol) and **J** (53.26 mg, 0.205 mmol, 1.1 equiv.), [IrCl(cod)]$_2$ (2.38 mg, 0.0035 mmol, 2 mol%) and (*S*)-DTBM-SEGPGOS (10.54 mg, 0.0089 mmol, 4 mol%) in benzene (0.4 mL) was heated at 80 °C for 12 h. **6J** was obtained as a white solid in 67% yield (39.0 mg, 0.123 mmol) after purification by column chromatography on silica gel (DIOL-silica; eluent: hexane only).
^{1}H NMR (400 MHz, CDCl$_3$) δ 7.67-7.64 (m, 19H), 7.48-7.37 (m, 34H), 7.10 (dd, J = 8.0, 1.1 Hz, 5H), 7.01-6.89 (m, 14H), 5.14 (dd, J = 8.7, 0.9 Hz, 5H).
^{13}C NMR (101 MHz, CDCl$_3$) δ 161.2, 149.6, 146.7, 134.8, 133.4, 130.7, 128.1, 126.2, 123.5, 123.2, 122.9, 101.7.
^{29}Si NMR (79 MHz, CDCl$_3$) δ -10.6.
HRMS (EI) m/z: [M]+ Calcd. for $C_{20}H_{16}O_2Si$, 316.0920; Found, 316.0919.

2,2-diphenyl-2,5,6,7-tetrahydro-1,2-oxasilepine (8J)

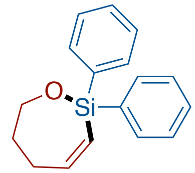

According to the **General Procedure**, a mixture of **7** (26.9 mg, 0.32 mmol) and **J** (94.85 mg, 0.36 mmol, 1.14 equiv.), $[IrCl(cod)]_2$ (7.14 mg, 0.011 mmol, 3 mol%) and (*S*)-DTBM-SEGPGOS (23.11 mg, 0.020 mmol, 6 mol%) in benzene (0.6 mL) was heated at 80 °C for 12 h. **8J** was obtained as a white solid in 42% yield (35.5 mg, 0.133 mmol) after purification by column chromatography on silica gel (DIOL-silica; eluent: hexane only) and preparative GPC.

^{1}H NMR (400 MHz, $CDCl_3$) δ 1H-NMR (400 MHz, CHLOROFORM-D) δ 7.64-7.61 (m, 4H), 7.45-7.36 (m, 6H), 6.36 (dt, J = 6.4, 1.4 Hz, 1H), 4.82 (dt, J = 13.0, 6.4 Hz, 1H), 2.20 (ddd, J = 11.5, 6.5, 1.0 Hz, 2H), 1.90-1.83 (m, 2H), 1.46-1.43 (m, 2H).

^{13}C NMR (101 MHz, $CDCl_3$) δ 141.9, 135.3, 134.3, 130.1, 128.0, 111.0, 27.3, 21.9, 14.8.

HRMS (EI) m/z: [M]+ Calcd. for $C_{17}H_{18}OSi$, 266.1127; Found, 266.1122.

2,2-diphenyl-5,6-dihydro-2*H*-1,2-oxasiline (10J)

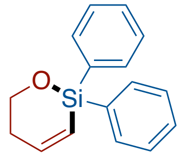

According to the **General Procedure**, a mixture of **9** (36.2 mg, 0.517 mmol, 1.46 equiv.) and **J** (92.14 mg, 0.354 mmol), $[IrCl(cod)]_2$ (5.22 mg, 0.0078 mmol, 2 mol%) and (*S*)-DTBM-SEGPGOS (18.73 mg, 0.0159 mmol, 4 mol%) in benzene (0.6 mL) was heated at 80 °C for 12 h. **10J** was obtained as a white solid in 17% yield (15.5 mg, 0.062 mmol) after purification by column chromatography on silica gel (DIOL-silica; eluent: hexane only).

^{1}H NMR (400 MHz, $CDCl_3$) δ 7.65-7.62 (m, 4H), 7.48-7.44 (m, 2H), 7.43-7.39 (m, 4H), 6.52 (td, J = 4.1, 2.1 Hz, 1H), 4.74 (td, J = 4.4, 6.4 Hz, 1H), 2.35-2.30 (m, 2H), 1.34 (t, J = 7.3 Hz, 2H).

^{13}C NMR (101 MHz, $CDCl_3$) δ 143.0, 134.3, 130.4, 128.1, 106.1, 18.6, 7.6.

HRMS (EI) m/z: [M]+ Calcd. for $C_{16}H_{16}OSi$, 252.0970; Found, 252.0968.

ethoxydiphenyl(vinyl)silane (12J)

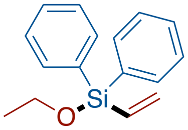

According to the **General Procedure**, a mixture of **11** (32.7 mg, 0.454 mmol, 1.73 equiv.) and **J** (68.12 mg, 0.262 mmol), $[IrCl(cod)]_2$ (4.56 mg, 0.0068 mmol, 2.5 mol%) and (*S*)-DTBM-SEGPGOS (13.74 mg, 0.0116 mmol, 4.4 mol%) in benzene (0.4 mL) was heated at 80 °C for 12 h. **12J** was obtained as a colorless oil in 12% yield (8.2 mg, 0.032 mmol) after purification by column chromatography on silica gel (DIOL-silica; eluent: hexane only).

^{1}H NMR (400 MHz, $CDCl_3$) δ 7.62-7.60 (m, 4H), 7.45-7.36 (m, 6H), 6.49 (dd, J = 20.4, 14.9 Hz, 1H), 6.27 (dd, J = 15.1, 3.7 Hz, 1H), 5.90 (dd, J = 20.1, 3.7 Hz, 1H), 3.84 (q, J = 7.0 Hz, 2H), 1.24 (t, J = 6.9 Hz, 3H).

^{13}C NMR (101 MHz, $CDCl_3$) δ 137.0, 135.1, 134.5, 133.7, 130.0, 127.9, 59.7, 18.5.

HRMS (EI) m/z: [M]+ Calcd. for $C_{16}H_{18}OSi$, 254.1127; Found, 254.1123.

5. Synthetic Applications

5.1. Functionalized Cyclic Silylenes

Reaction of *khellin* (2m) and N

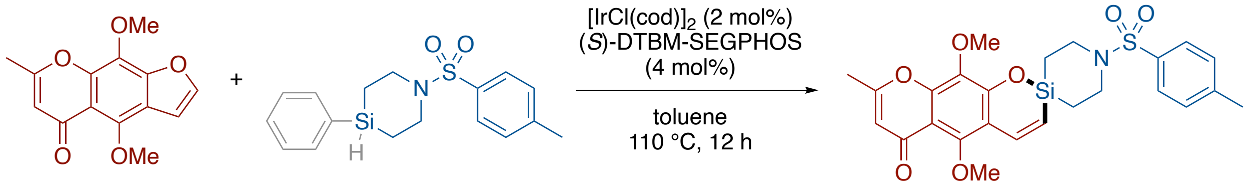

According to the **General Procedure**, a mixture of *khellin* **2m** (30.9 mg, 0.12 mmol), hydrosilane **N** (synthesized according to the literature,[22] 41.7 mg, 0.13 mmol, 1.1 equiv), $[IrCl(cod)]_2$ (2.1 mg, 0.003 mmol, 2 mol%) and (*S*)-DTBM-SEGPHOS (6.9 mg, 0.0058 mmol, 4 mol%) in toluene (400 µL) was heated at 110 °C for 12 h. **2mN** was obtained as white solid in 36% yield (22 mg, 0.042 mmol) after purification by column chromatography on silica gel (SiliaFlashthe ; eluent: hexane : Et_2O = 90 : 10) and preparative GPC. The structure of **2mN** was also determined by scXRD as mentioned below.

^{1}H NMR (400 MHz, $CDCl_3$) *δ*. 7.85 (d, J = 15.1 Hz, 1H), 7.69 (dt, J = 8.4, 1.9 Hz, 2H), 7.33 (d, J = 7.8 Hz, 2H), 6.01 (d, J = 0.9 Hz, 1H), 5.95 (d, J = 15.1 Hz, 1H), 3.84-3.90 (m, 2H), 3.83 (d, J = 2.7 Hz, 6H), 3.30-3.23 (m, 2H), 2.45 (s, 3H), 2.34 (s, 3H), 1.28-1.18 (m, 4H).

^{13}C NMR (101 MHz, $CDCl_3$) *δ* 177.0, 164.2, 153.1, 152.2, 150.8, 143.5, 141.5, 141.3, 141.1, 135.9, 134.5, 130.3, 130.2, 130.2, 130.0, 129.9, 129.9, 129.8, 129.7, 129.5, 129.5, 129.5, 127.2, 119.6, 116.6, 112.3, 111.4, 63.2, 61.3, 45.8, 45.3, 44.8, 21.6, 20.2, 16.0.

^{29}Si NMR (79 MHz, $CDCl_3$) *δ* 0.4.

HRMS (ESI) m/z: [M + Na]+ Calcd. for $C_{25}H_{27}O_7SNSiNa$, 536.1175; Found, 536.1165.

5.2. Formal Silicon Atom Transfer

Reaction of phenylsilane (O) and 13

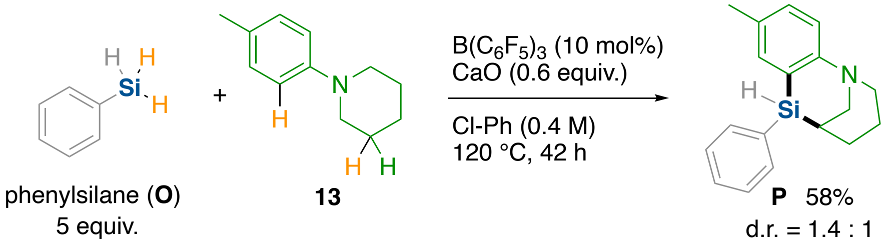

According to the reported procedure,[23] an oven dried 200 mL two-neck flask, equipped with a magnetic stirring bar and rubber septum was charged CaO (Wako, 78 mg, 1.4 mmol, 0.7 equiv.) then brought into grove box filled with nitrogen. $B(C_6F_5)$ (TCI, 46.4mg, 0.09 mmol, 4 mol%) and chlorobenzene (5 mL) were charged to the flask and brought out of the grove box while sealed. 1-(*p*-tolyl)piperidine **13** (synthesized according to literature,[24] 360.1 mg, 2.06 mmol) and phenylsilane (TCI, 1.229 mg, 11.4 mmol, 5.5 equiv.) were added to the flask dropwise, and the mixture was stirred at 120 °C for 18 h. Another portion of $B(C_6F_5)$ (64.9 mg) was added to the flask and further stirred at 120 °C for 24 h. After the reaction finished, solvent was removed under reduced pressure. The resulting crude materials was purified by column chromatography on silica gel (SiliaFlash; eluent: hexane: AcOEt = 90 : 10) and kügelrhor distillation. (332 mg, 1.19 mmol, 58% yield, d.r. = 1.38 : 1) was obtained as a white solid. The spectrum data of the product suits to the reported literature.[23]

^{1}H NMR (400 MHz, $CDCl_3$) δ. 7.65-7.59 (m, 2H), 7.48-7.38 (m, 3H), 7.26 (dd, J = 10.5, 1.8 Hz, 1H), 7.20 (dd, J = 8.2, 1.8 Hz, 1H), 7.07 (t, J = 8.0 Hz, 1H), 5.03 (d, J = 3.2 Hz, 0.41H), 4.77 (d, J = 1.4 Hz, 0.58H), 3.70-3.62 (m, 0.85H), 3.58-3.49 (m, 1.19H), 3.39-3.29 (m, 2H), 2.30 (s, 3H), 2.22 (dd, J = 13.0, 2.1 Hz, 0.59H), 2.05 (tt, J = 13.3, 4.6 Hz, 0.58H), 1.96-1.87 (m, 0.42H), 1.80-1.65 (m, 1.41H), 1.45-1.44 (m, 0.42H), 1.39 (s, 0.59H), 1.27 (d, J = 13.7 Hz, 0.6H), 1.14-1.10 (m, 0.42H)

^{13}C NMR (101 MHz, $CDCl_3$) δ 156.8, 136.1, 135.9, 135.7, 135.2, 134.9, 132.9, 132.2, 132.1, 132.0, 132.0, 129.9, 129.9, 128.2, 128.1, 125.9, 125.8, 125.5, 124.9, 56.7, 56.7, 53.0, 52.2, 28.4, 26.2, 20.9, 19.0, 18.9, 17.8, 14.9.

Iridium-Catalyzed Silylene Insertion of P and 1a

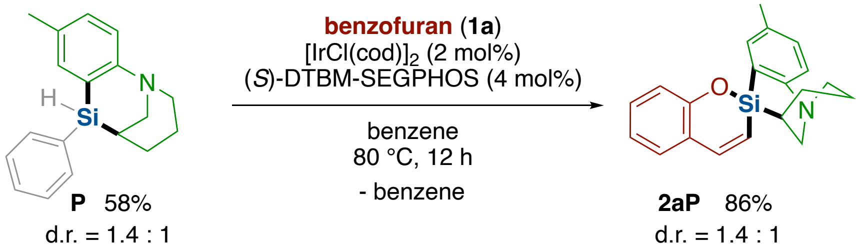

According to the **General Procedure**, a mixture of **1a** (26.4 mg, 0.22 mmol), **P** (70.1 mg, 0.26 mmol, 1.2 equiv) and 400 µL of Ir catalyst stock solution (containing $[IrCl(cod)]_2$ (3.0 mg, 0.0044 mmol, 2 mol%), (*S*)-DTBM-SEGPHOS (9.7 mg, 0.0082 mmol, 4 mol%) and benzene (400 µL, 0.5M)) was heated at 80 °C for 12 h. **2aP** was obtained as a white solid in 86% yield (60.2 mg, d.r. = 1.39 : 1, 50% yield over 2 steps) after purification by column chromatography on silica gel (SiliaFlash; eluent: hexane: Et_2O = 95: 5) and preparative GPC. Diastereomeric ratio didn't change during the Silylene Insertion.

^{1}H NMR (400 MHz, $CDCl_3$) δ 7.62 (d, J = 14.2 Hz, 0.44H), 7.51 (d, J = 14.2 Hz, 0.6H), 7.29-7.19 (m, 5H), 7.01-6.97 (m, 1H), 6.96-6.91 (m, 1H), 6.00 (d, J = 14.2 Hz, 0.58H), 5.95 (d, J = 14.2 Hz, 0.42H), 3.79 (ddd, J = 21.8, 13.8, 1.5 Hz, 1H), 3.68-3.64 (m, 1H), 3.53-3.39 (m, 1H), 3.36-3.28 (m, 1H), 2.26 (s, 1.38H), 2.25 (s, 1.57H),

2.09 (qt, J = 13.6, 3.8 Hz, 1H), 1.98-1.83 (m, 1H), 1.63 (qt, J = 13.6, 4.1 Hz, 0.46H), 1.50 (d, J = 14.2 Hz, 0.6H), 1.40-1.26 (m, 2H)

^{13}C NMR (101 MHz, CDCl$_3$) δ 153.7, 153.2, 152.2, 151.9, 149.9, 147.8, 135.4, 135.2, 135.1, 134.3, 133.4, 133.4, 131.0, 130.1, 129.9, 128.9, 125.0, 124.6, 124.0, 124.0, 121.4, 121.3, 119.8, 119.7, 119.0, 117.7, 56.4, 56.1, 53.3, 52.6, 24.5, 24.2, 20.9, 20.8, 20.5, 19.1, 18.9, 18.7.

HRMS (EI) m/z: [M]+ Calcd. for $C_{20}H_{21}ONSi$, 319.1392; Found, 319.1390.

5.3. Iridum-Catalyzed Germylene Insertion

Preperation of Triphenylgelmane (Q)

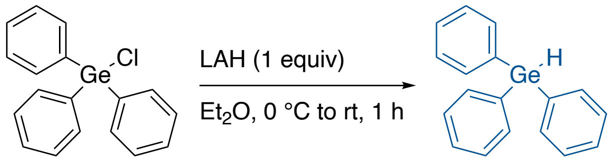

An oven dried 200 mL three-neck flask, equipped with a magnetic stirring bar and rubber septum was evacuated and backfilled with nitrogen. Triphenylchlorogerman (TCI, 503.7 mg, 1.48 mmol) and Et_2O (7.5 mL) were charged to the flask and cooled to 0 °C. LAH (Aldrich, 87.5 mg, 2.3 mmol, 1.5 equiv) was slowly added to the flask under 0 °C, then the mixture was stirred at room temperature overnight. H_2SO_4 was added to the flask. The organic products were extracted by Et_2O, washed with Brine, and dried over Na_2SO_4. Solvent was removed under reduced pressure. The resulting crude materials were purified by kügelrhor distillation. Triphenylgelmane (**Q**) (414.8 mg, 1.36 mmol, 92% yield) was obtained as a white solid. The spectrum data of the product suits to the reported literature.[25]

^{1}H NMR (400 MHz, CDCl$_3$) δ 7.61-7.58 (m, 6H), 7.45-7.40 (m, 10H), 5.78 (s, 1H).

^{13}C NMR (101 MHz, CDCl$_3$) δ 135.6, 135.3, 129.3, 128.5.

Iridum-Catalyzed Germylene Insertion Reaction

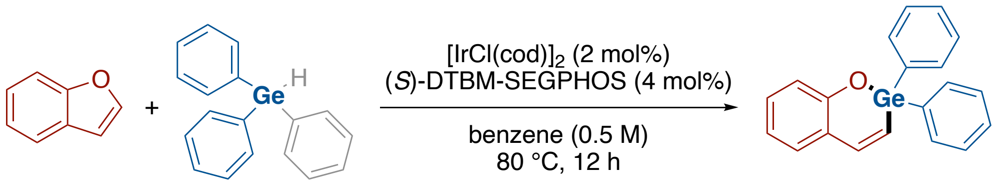

According to the **General Procedure**, a mixture of benzofuran **1a** (12.3 mg, 0.1 mmol), triphenylgelmane **Q** (36.7 mg, 0.12 mmol, 1.2 equiv), [IrCl(cod)]$_2$ (1.18 mg, 0.0017 mmol, 2 mol%) and (*S*)-DTBM-SEGPHOS (3.79 mg, 0.0032 mmol, 4 mol%) in benzene (0.2 mL) was heated at 80 °C for 12 h. **2aQ** was obtained as a colorless crystal in 89% yield (32 mg, 0.09 mmol) after purification by column chromatography on silica gel (SiliaFlash; eluent: hexane : Et_2O = 95 : 5). The structure of **2aQ** was also checked by scXRD as mentioned below.

^{1}H NMR (400 MHz, CDCl$_3$) δ 7.64 (td, J = 3.8, 2.0 Hz, 4H), 7.50-7.41 (m, 7H), 7.17 (td, J = 7.5, 1.7 Hz, 1H), 7.12 (dd, J = 7.5, 1.6 Hz, 1H), 6.98 (d, J = 7.3 Hz, 1H), 6.85 (td, J = 7.4, 1.1 Hz, 1H), 6.24 (d, J = 13.3 Hz, 1H).

^{13}C NMR (101 MHz, CDCl$_3$) δ 157.6, 146.6, 135.4, 134.1, 132.0, 130.6, 130.1, 128.7, 123.0, 120.3, 119.4, 116.8.

HRMS (EI) m/z: [M]+ Calcd. for $C_{20}H_{16}OGe$, 346.0413; Found, 346.0405.

5.4. Silylene-Inserting Polymerization

Synthesis of Monomer

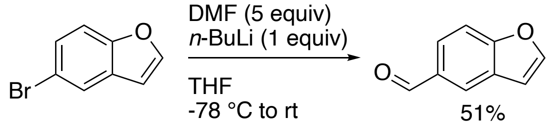

To An oven dried 300 mL three-neck flask, equipped with a magnetic stirring bar and rubber septum was evacuated and backfilled with Ar. 5-Bromobenzofuran (BLD, 2.63 g, 13.4 mmol) and THF (30 mL) were charged to the flask and cooled to -78 °C. *n*-BuLi (nacalai, 1.6 M in hexane, 8.5 mL, 1 equiv) was slowly added to the flask under -78 °C, then the mixture was stirred at -78 °C for 0.5 h. DMF (Wako, Super dehydrated, stabilizer free, 5.01 g, 68.6mmol, 5 equiv) was added to the flask dropwise, and the mixture was sttired at room temperature, overnight. Water was added to the flask. The organic products were extracted by Et_2O, washed with Brine, and dried over Na_2SO_4. Solvent was removed under reduced pressure. The resulting crude materials were purified by column chromatography on silica gel (SiliaFlash; eluent: hexane: Et_2O = 90 : 10). benzofuran-5-carbaldehyde (1.01 g, 6.9 mmol, 51% yield) was obtained as a white solid.

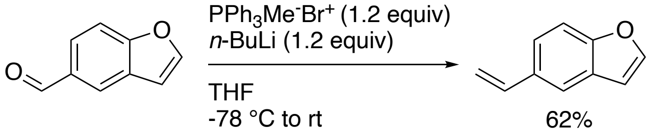

To An oven dried 200 mL three-neck flask, equipped with a magnetic stirring bar and rubber septum was evacuated and backfilled with Ar. Ph_3PMeBr (TCI, 2.93 g, 8.2 mmol, 1.2 equiv) and THF (20 mL) were charged to the flask and cooled to 0 °C. *n*-BuLi (nacalai, 1.6 M in hexane, 5 mL, 1.2 equiv) was slowly added to the flask under 0 °C, then the mixture was stirred at 0 °C for 1 h, generating yellow dispersion. benzofuran-5-carbaldehyde (1.01 g, 6.9 mmol) was added to the flask dropwise, and the mixture was sttired at room temperature for 3 h. Water was added to the flask. The organic products were extracted by Et_2O, washed with Brine, and dried over Na_2SO_4. Solvent was removed under reduced pressure. The resulting crude materials were purified by kügelrhor distillation. 5-vinylbenzofuran (618.7 mg, 4.3 mmol, 62% yield) was obtained as a colorless oil.

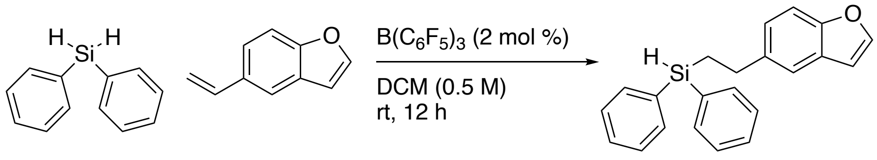

14 55%

According to reported method,[26] to An oven dried 200 mL three-neck flask, equipped with a magnetic stirring bar and rubber septum was charged tris(pentafluorophenyl)boran (TCI, 35.5 mg, 0.07 mmol, 2 mol%), then backfilled with Ar. DCM (Wako, 10 mL) were charged to the flask, following diphenylsilane (TCI, 1.01 g, 5.5 mmol, 1.3 equiv) and 5-vinylbenzofuran (600 mg, 4.2 mmol) was added to the flask. The mixture was stirred at room temperature, overnight. Water was added to the flask. The organic products were extracted by Et_2O, washed with Brine, and dried over Na_2SO_4. Solvent was removed under reduced pressure. The resulting crude materials were purified by column chromatography on silica gel (SiliaFlash; eluent: hexane: Et_2O = 90 : 10) and kügelrhor distillation. Monomer (2-(benzofuran-5-yl)ethyl)diphenylsilane (**14**) (757.8 mg, 2.3 mmol, 55% yield) was obtained as a colorless sticky oil.

^{1}H NMR (400 MHz, $CDCl_3$) δ 7.64-7.61 (m, 5H), 7.46-7.38 (m, 8H), 7.14 (dd, J = 8.7, 1.8 Hz, 1H), 6.72 (dd, J = 2.3, 0.9 Hz, 1H), 4.94 (t, J = 3.7 Hz, 1H), 2.92-2.88 (m, 2H), 1.59 (ddd, J = 11.9, 5.0, 3.7 Hz, 2H).

^{13}C NMR (101 MHz, $CDCl_3$) δ 153.6, 145.2, 139.0, 135.3, 134.3, 129.8, 128.2, 127.6, 124.6, 120.0, 111.1, 106.5, 30.5, 15.2.

HRMS (EI) m/z: [M]+ Calcd. for $C_{22}H_{20}OSi$, 328.1283; Found, 328.1281.

Iridum-Catalyzed Silylene-Inserting Polymerization

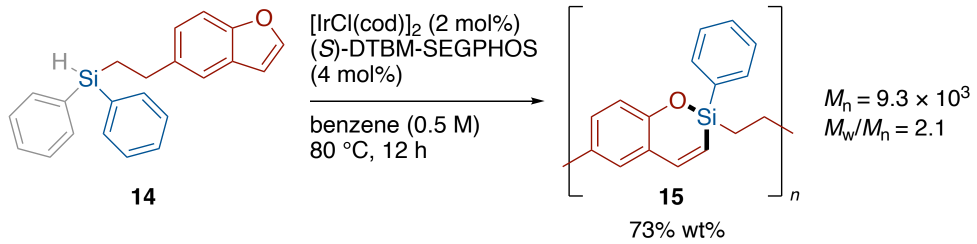

According to the **General Procedure**, a mixture of monomer **14** (112.2 mg, 0.34 mmol), $[IrCl(cod)]_2$ (1.18 mg, 0.0017 mmol, 2 mol%) and (*S*)-DTBM-SEGPHOS (3.79 mg, 0.0032 mmol, 4 mol%) in benzene (0.8 mL) was heated at 80 °C for 12 h. The solvent was removed under reduced pressure. The polymer **15** (62.7 mg, 73 wt% yield, $M_n = 9.3\times10^3$, $M_w/M_n = 2.1$) was obtained as white solid after purification by reprecipitation from hexane and $CHCl_3$.

^{1}H NMR (400 MHz, $CDCl_3$) δ 7.58-7.56 (m, 2H), 7.38-7.36 (m, 4H), 6.97-6.79 (m, 3H), 5.91 (d, J = 14.2 Hz, 1H), 2.72 (t, J = 8.0 Hz, 2H), 1.56-1.48 (m, 1H), 1.41-1.33 (m, 1H).

^{13}C NMR (101 MHz, $CDCl_3$) δ 151.8, 147.5, 136.4, 135.9, 133.8, 130.4, 130.1, 129.2, 128.1, 123.9, 119.9, 119.3, 27.8, 17.8.

^{29}Si NMR (79 MHz, $CDCl_3$) δ -4.6.

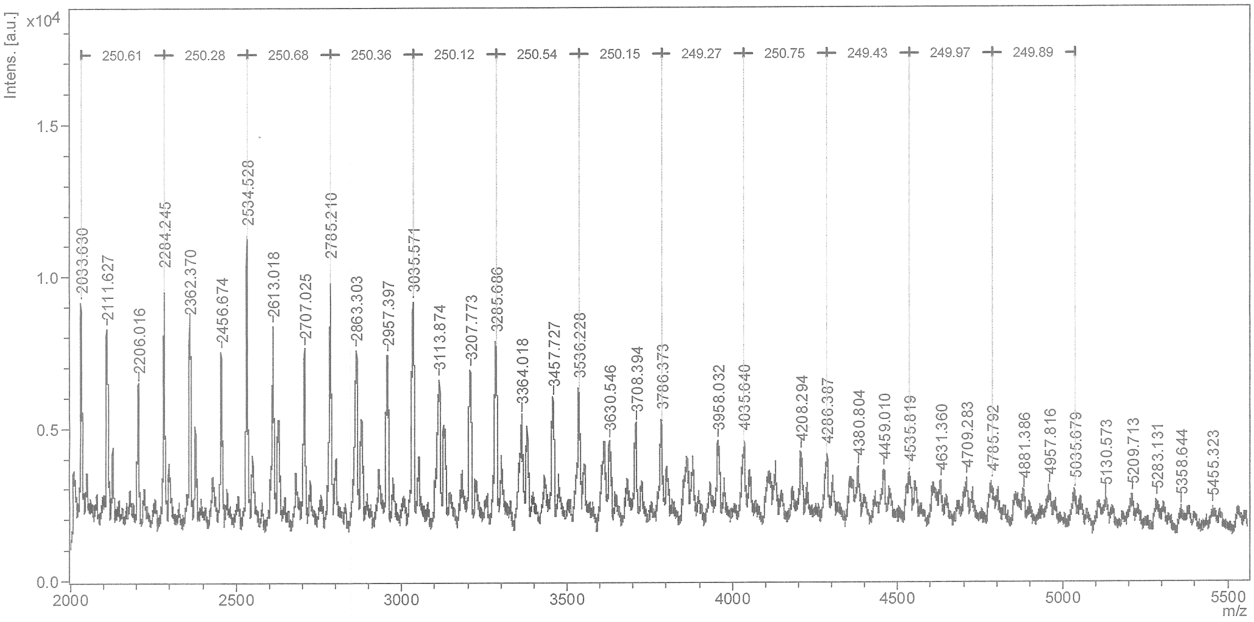

Figure S7. MALDI spectrum of polymer **15**. Monomer unit weight 250 was repeatedly observed as Ag+ ion.

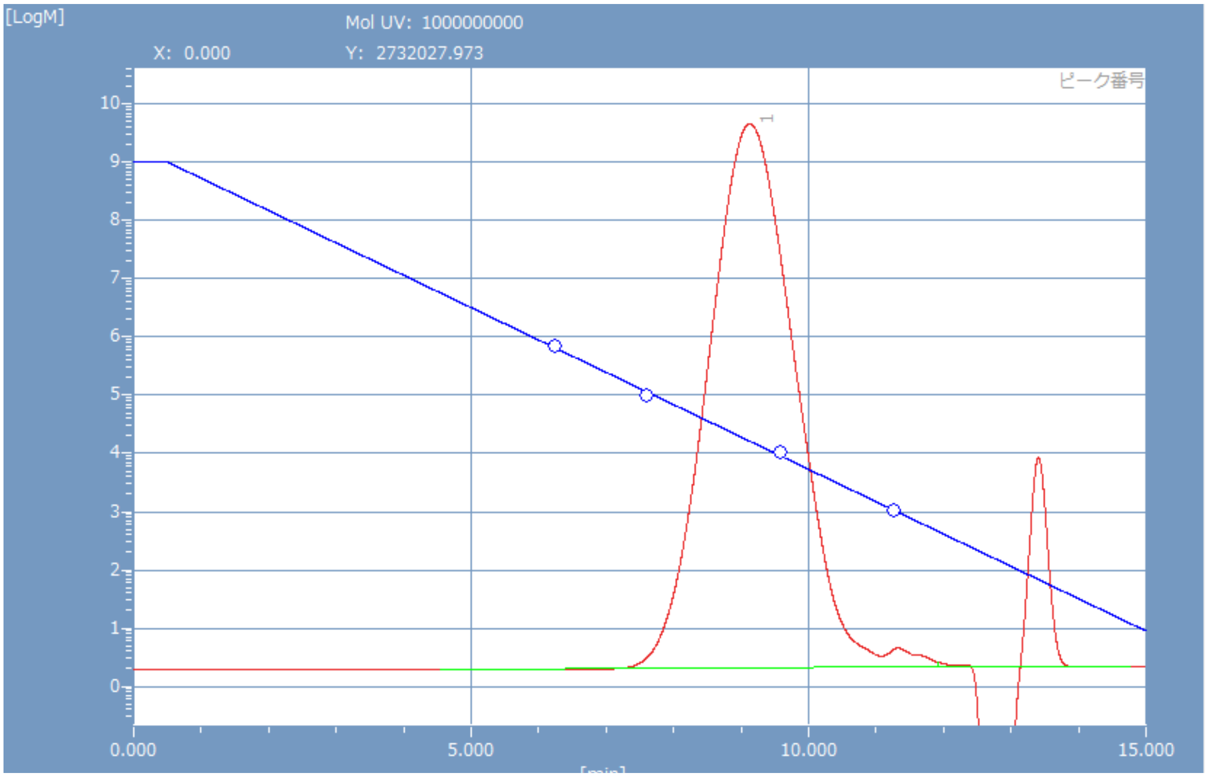

Figure S8. Analytical GPC chart of purified polymer **15**. Blue line: polystyrene standard.

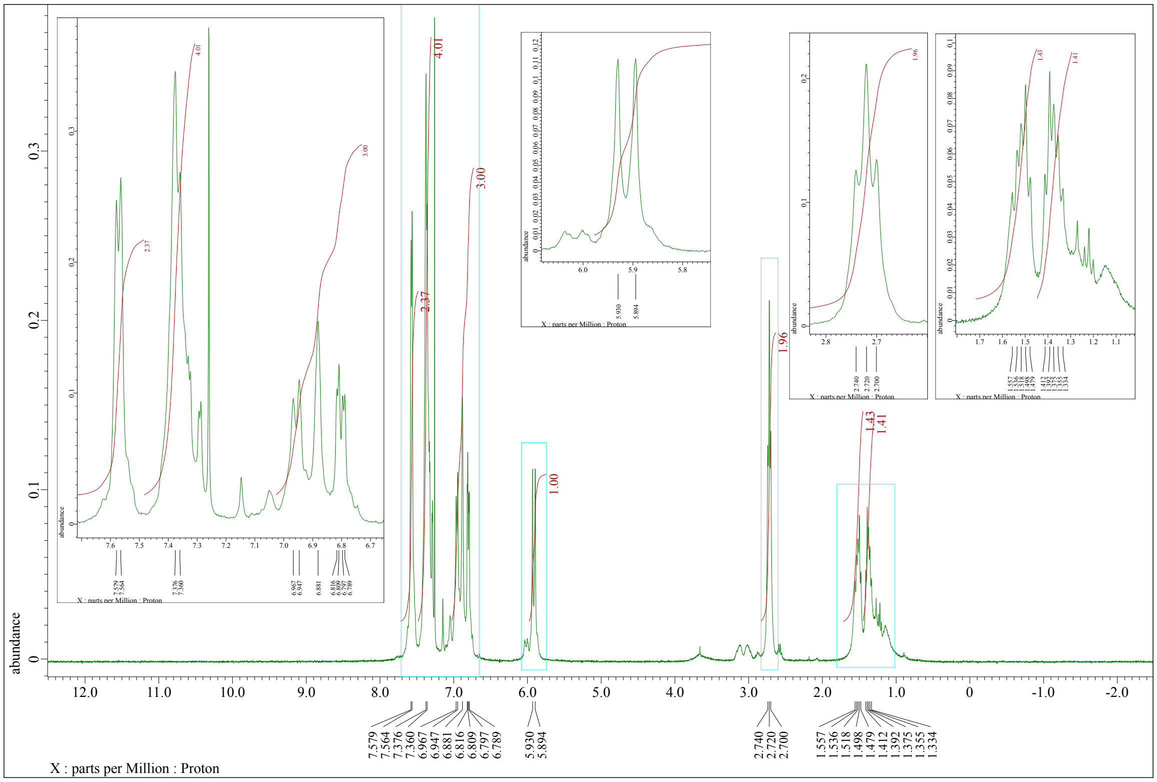

Figure S9. ^{1}H NMR chart of **15.**

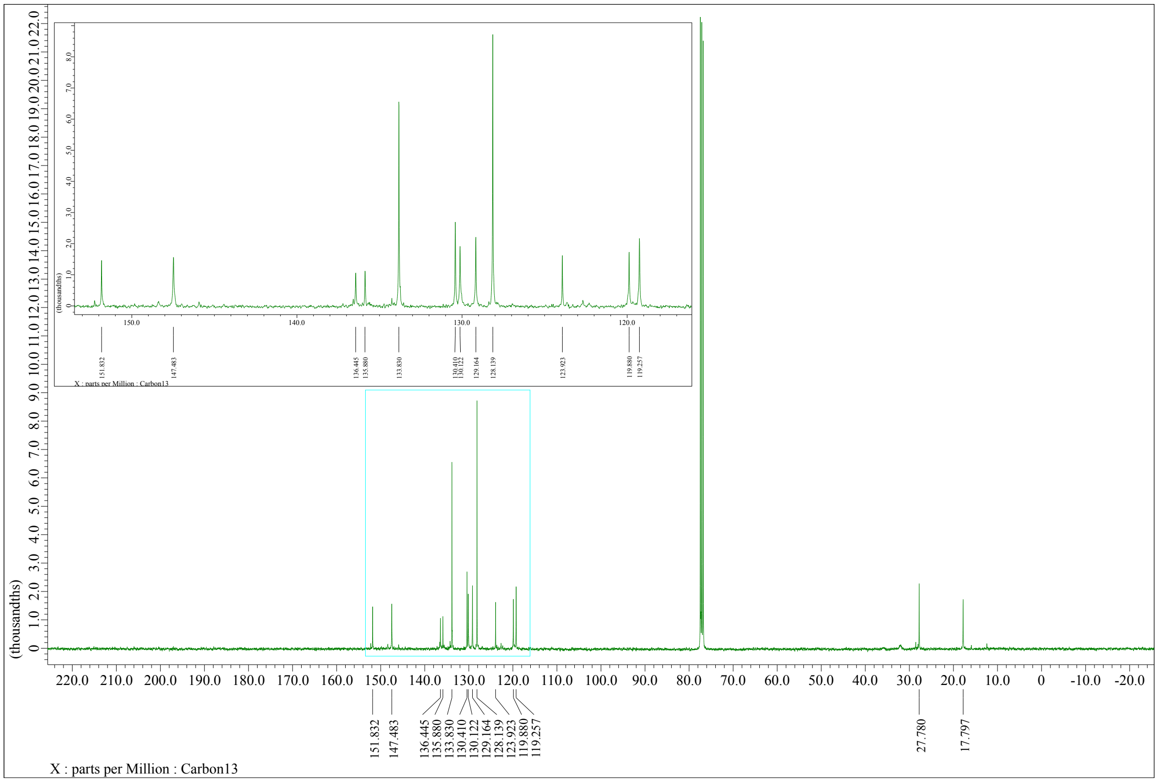

Figure S10. ^{13}C NMR chart of **15.**

Time Course of the Reaction

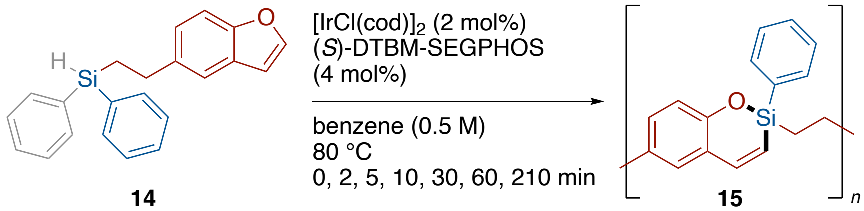

According to the **General Procedure**, a mixture of monomer **14** (0.03 mmol), $[IrCl(cod)]_2$ (0.002 mmol, 2 mol%) and (*S*)-DTBM-SEGPHOS (0.004 mmol, 4 mol%) in benzene (0.2 M) was heated at 80 °C. GPC measurement was carried out in 0, 2, 5, 10, 30, 60 and 210 minutes. In each sampling, 10 μL of the reacting solution was taken out in a grove box. Mp (peak top weight) was plotted below (Table **S5**, Figure **S11**). The polymerization occurred even in 2 minutes. Elongation of the polymer chain was observed as the reaction time increased.

Table S5. Reaction Time

Reacting time	Mp /10^3	Degree of polymerization
0 min.	1.8	7.2
2 min.	7.4	30
5 min.	10.0	40
10 min.	10.6	42
30 min.	11.0	44
60 min.	11.7	47
210 min.	12.2	49

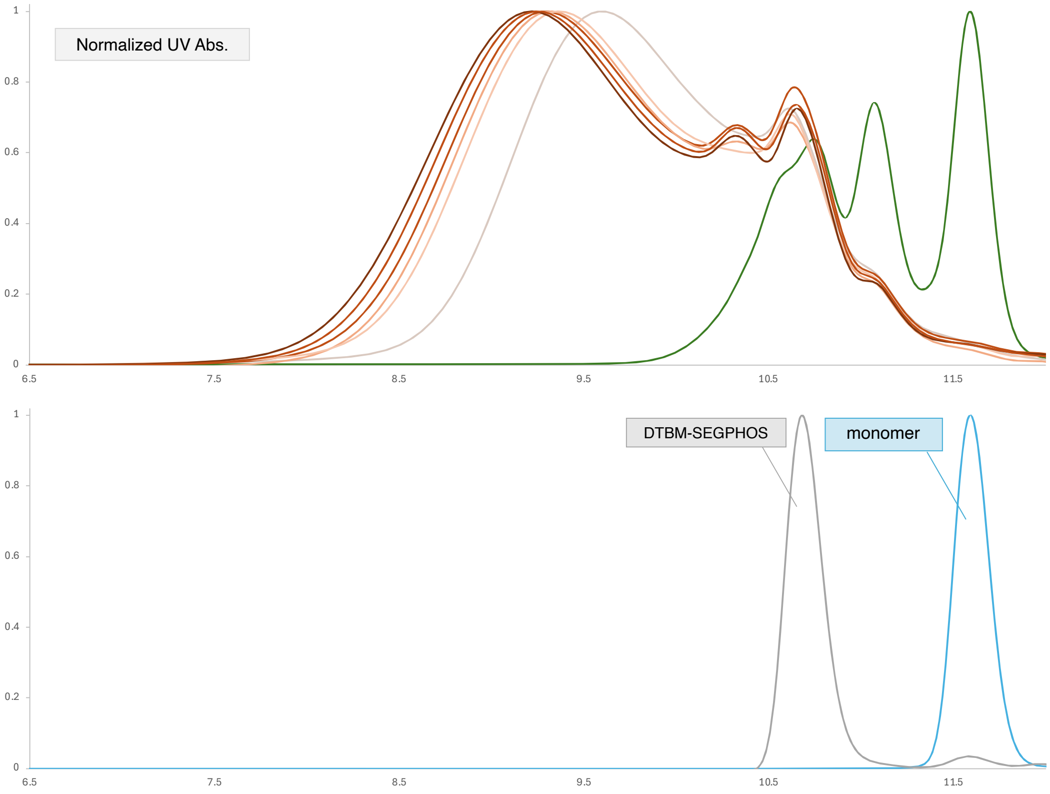

Figure S11. GPC chart of elongation of the polymer.

6. Mechanistic Studies

6.1. Detection of Silyliridiumhydride Complex *in situ*

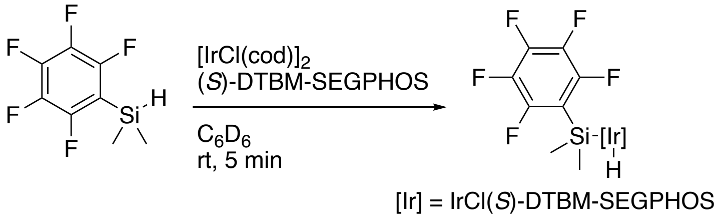

In a glovebox, a well-oven dried NMR tube equipped with a PTFE stopcock (Wilmad, outside diameter : 5 mm), was charged with [IrCl(cod)]$_2$ (16.95 mg, 0.025 mmol, 7 mol%), (*S*)-DTBM-SEGPHOS (59.18 mg, 0.050 mmol, 14 mol%) and C_6D_6 (0.6 mL). The tube was sealed by the stopcock, taken out from the glovebox and heated at aluminum block (diameter : 6.5 mm) at 80 ℃ for 1 hour to form Ir/DTBM-SEGPHOS complex (green). Glass tube was brough into grove box, to which hydrosilane **A6** (80.76 mg, 0.357 mmol) was added. The mixture was stirred at room temperature for 5 minutes (blue). Oxidatively added 2-benzofuryliridium complex was detected at ^{31}P NMR δ = 20.6, 20.5.

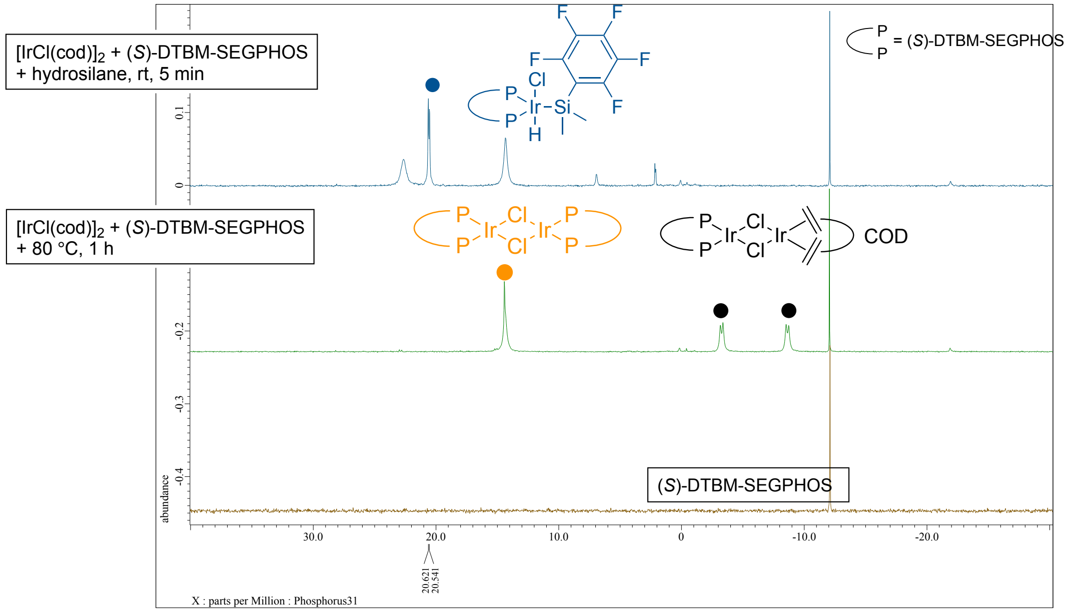

Figure S12 ^{31}P NMR of the mixture.

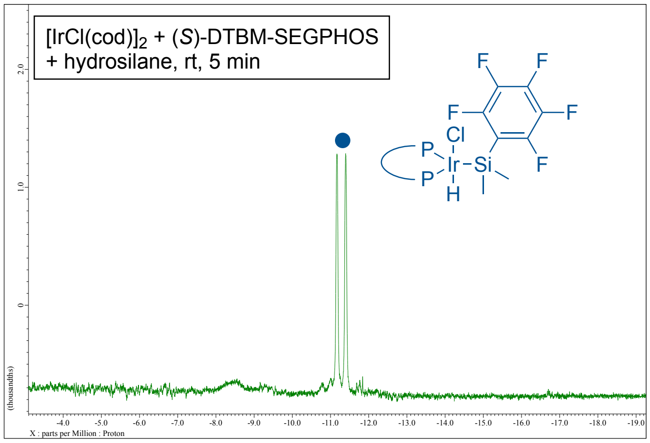

Figure S13 ^{1}H NMR of the mixture.

6.2. Detection of Benzofuryliridiumhydride Complex *in situ*

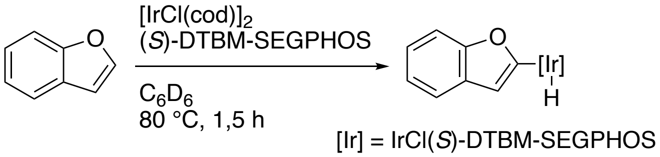

In a glovebox, a well-oven dried NMR tube equipped with a PTFE stopcock (Wilmad, outside diameter : 5 mm), was charged with $[IrCl(cod)]_2$ (10.30 mg, 0.015 mmol, 6 mol%), (*S*)-DTBM-SEGPHOS (41.24 mg, 0.035 mmol, 13 mol%) and C_6D_6 (0.6 mL). The tube was sealed by the stopcock, taken out from the glovebox and heated at aluminum block (diameter : 6.5 mm) at 80 ℃ for 1 hour to form Ir/DTBM-SEGPHOS complex (green). Glass tube was brough into grove box, to which benzofuran **1a** (31.38 mg, 0.266 mmol) was added. The mixture was stirred at room temperature for 5 minutes (brown), at 80 ℃ for 0.5 h (blue) and at 80 ℃ for 1.5 h (purple). Oxidatively added 2-benzofuryliridium complex was detected at ^{31}P NMR δ = 16.2, 15.5.

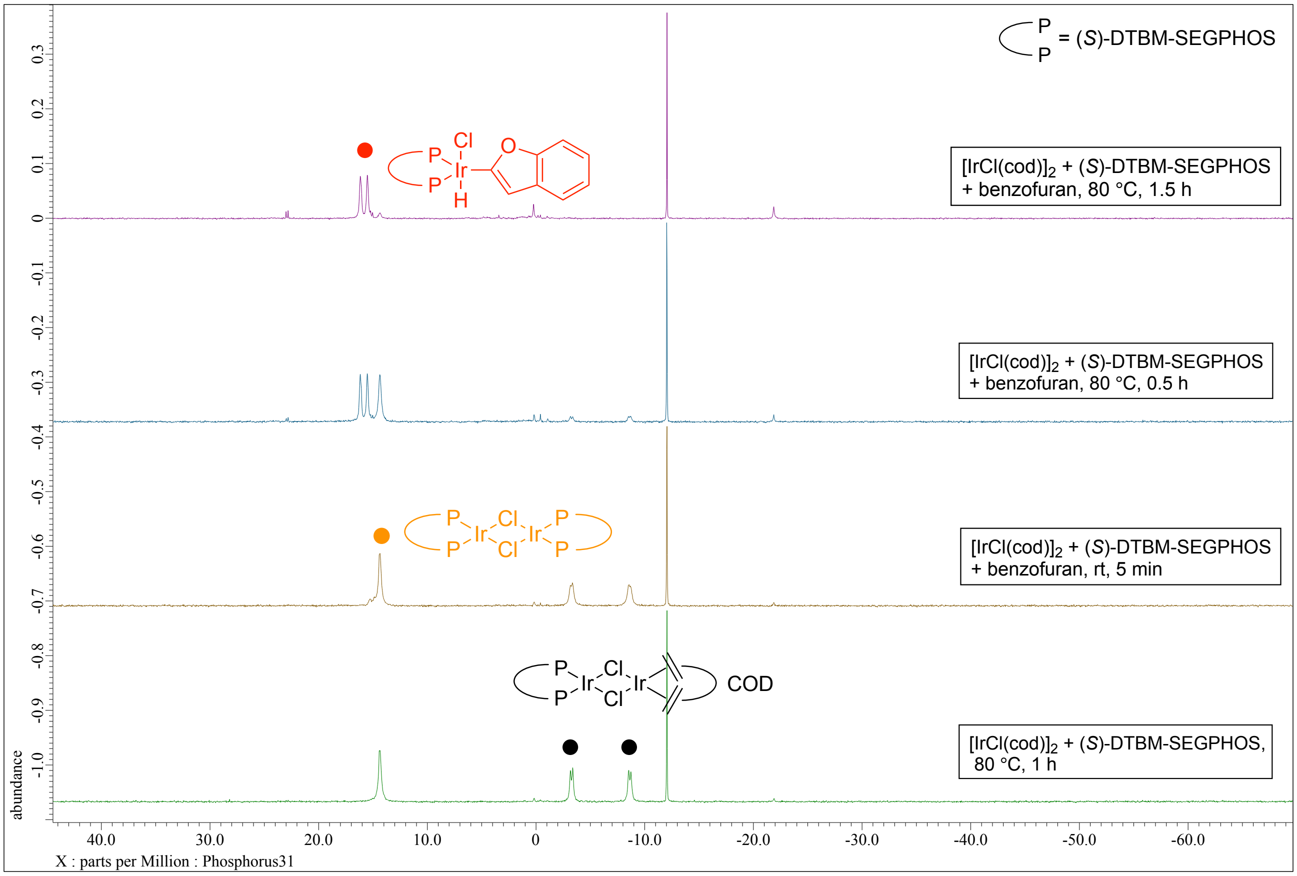

Figure S14 ^{31}P NMR of the mixture.

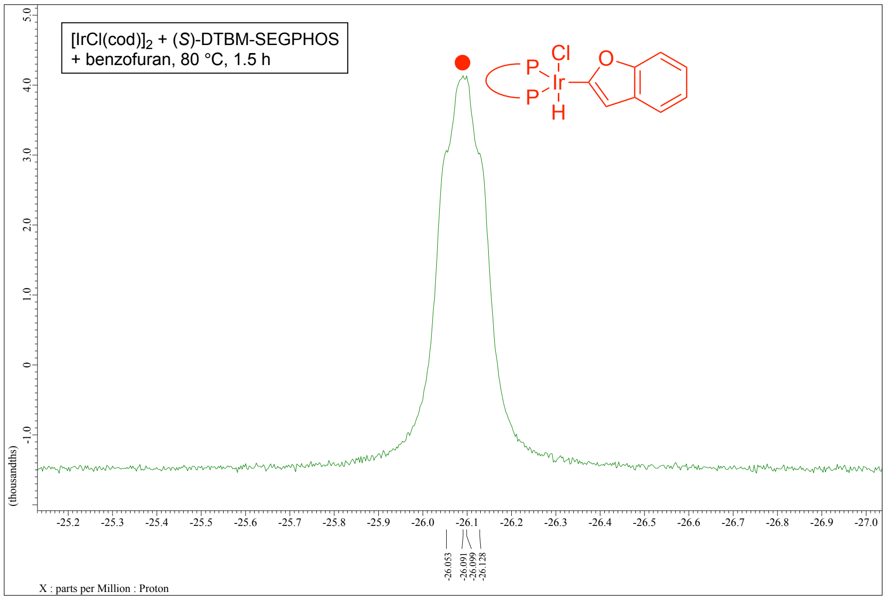

Figure S15 ^{1}H NMR of the mixture.

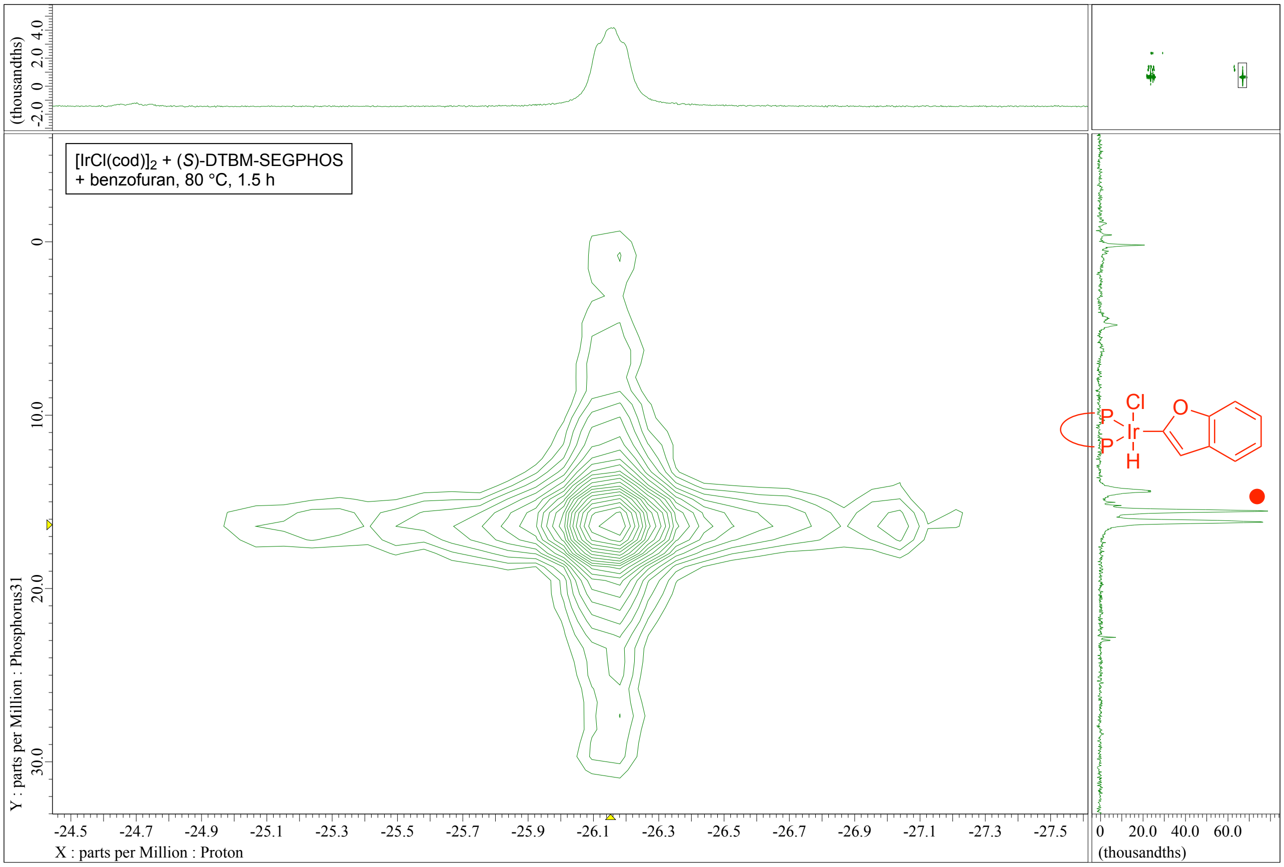

Figure S16 ^{31}P, ^{1}H HMQC of the reaction (J = 100 Hz, relaxation delay 1.5 s,)

6.3. Observation of the Behavior of the Substrates and Iridium Complex Mixture

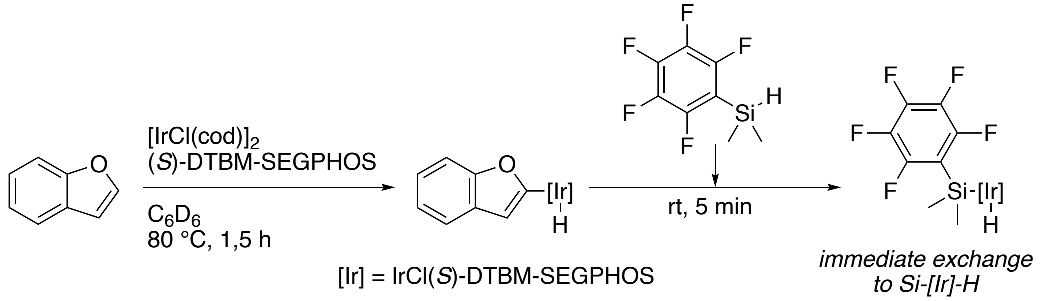

In a glovebox, a well-oven dried NMR tube equipped with a PTFE stopcock (Wilmad, outside diameter : 5 mm), was charged with $[IrCl(cod)]_2$ (10.27 mg, 0.015 mmol, 10 mol%), (*S*)-DTBM-SEGPHOS (36.11 mg, 0.030 mmol, 20 mol%) and C_6D_6 (0.6 mL). The tube was sealed by the stopcock, taken out from the glovebox and heated at aluminum block (diameter : 6.5 mm) at 80 ℃ for 1 hour to form Ir/DTBM-SEGPHOS complex (blue). Glass tube was brough into grove box, to which benzofuran **1a** (18.81 mg, 0.159 mmol) was added. The mixture was stirred at 80 ℃ for 1.5 h (brown). Then hydrosilane **A6** (26.80 mg, 0.119 mmol, 0.75 equiv.) was added and resulting mixture was stirred by shaking at room temperature for 5 minutes (green).

Note:2-H of benzofuran oxidatively added to the iridium complex initially after heating at 80 ℃. After adding hydrosilane to the solution, silyliridiumhydride was immediately formed.

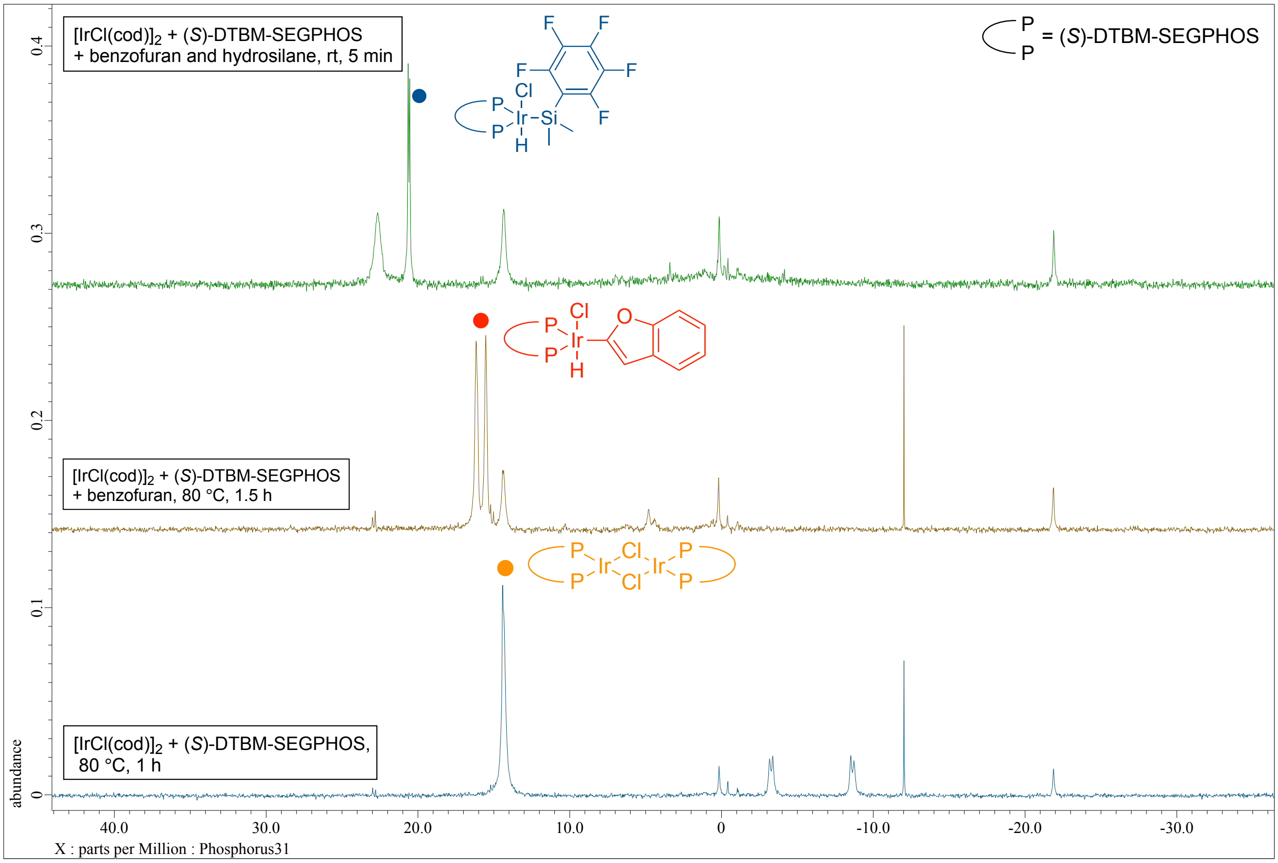

Figure S17. ^{31}P NMR of the mixture.

6.4. Detection of Silylene Exchange

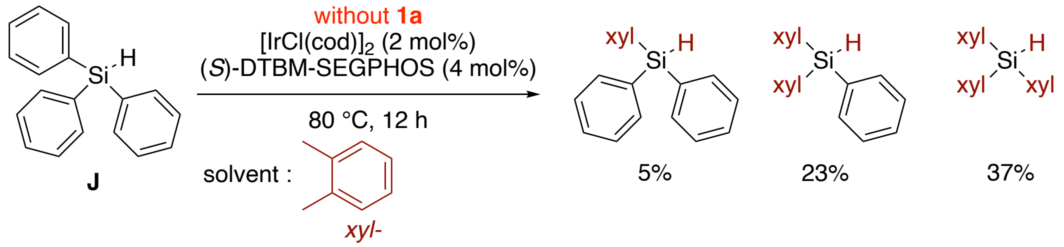

According to the **General Procedure**, a mixture of triphenylsilane **J** (47.6 mg, 0.183 mmol), $[IrCl(cod)]_2$ (1.7 mg, 0.002 mmol, 2 mol%) and (*S*)-DTBM-SEGPHOS (5.8 mg, 0.004 mmol, 4 mol%) in *o*-xylene (Nacalai, 400 μL) was heated in 80 °C for 12 h. The crude mixture was purified by column chromatography on silica gel (SiliaFlash; eluent: hexane: Et_2O = 99 : 1) to remove catalyst and solvent following preparative GPC to separate each product. **J** was fully converted to **(3,4-dimethylphenyl)diphenylsilane** (2.4 mg, 0.0083 mmol, 5% yield), **bis(3,4-dimethylphenyl)(phenyl)silane** (13.1 mg, 0.042 mmol, 23% yield) and **tris(3,4-dimethylphenyl)silane** (23.6 mg, 0.0686 mmol, 37% yield).

(3,4-dimethylphenyl)diphenylsilane

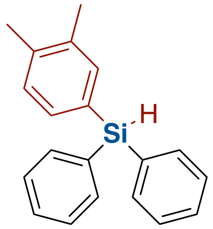

^{1}H NMR (400 MHz, $CDCl_3$) δ 7.56 (td, J = 3.9, 2.1 Hz, 4H), 7.43-7.38 (m, 2H), 7.37-7.33 (m, 5H), 7.30 (d, J = 7.3 Hz, 1H), 7.14 (d, J = 7.8 Hz, 1H), 5.42 (s, 1H), 2.27 (s, 3H), 2.24 (s, 3H).
^{13}C NMR (101 MHz, $CDCl_3$) δ 138.7, 137.0, 136.4, 135.9, 133.8, 133.6, 130.2, 129.8, 129.5, 128.1, 20.0, 19.8.
HRMS (EI) m/z: [M]+ Calcd. for $C_{13}H_{20}Si$, 204.1334; Found, 204.1332.

bis(3,4-dimethylphenyl)(phenyl)silane

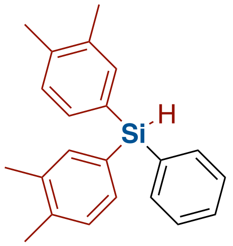

^{1}H NMR (400 MHz, $CDCl_3$) δ 7.57 (td, J = 4.0, 1.8 Hz, 2H), 7.42-7.33 (m, 5H), 7.30 (d, J = 7.3 Hz, 2H), 7.14 (d, J = 7.3 Hz, 2H), 5.39 (s, 1H), 2.27 (s, 6H), 2.24 (s, 6H).
^{13}C NMR (101 MHz, $CDCl_3$) δ 138.5, 137.0, 136.3, 135.9, 134.2, 133.6, 130.6, 129.6, 129.5, 128.0, 20.0, 19.8.
HRMS (EI) m/z: [M]+ Calcd. for $C_{13}H_{20}Si$, 204.1334; Found, 204.1332.

tris(3,4-dimethylphenyl)silane

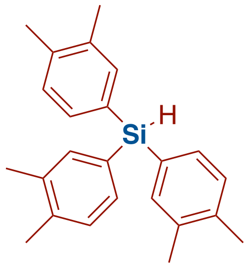

^{1}H NMR (400 MHz, $CDCl_3$) δ 7.36 (s, 2H), 7.32 (d, J = 7.3 Hz, 2H), 7.15 (d, J = 7.3 Hz, 2H), 5.38 (s, 1H), 2.28 (s, 6H), 2.25 (s, 6H).
^{13}C NMR (101 MHz, $CDCl_3$) δ 138.4, 137.0, 136.3, 133.6, 131.0, 129.5, 20.0, 19.8.
HRMS (EI) m/z: [M]+ Calcd. for $C_{13}H_{20}Si$, 204.1334; Found, 204.1332.

6.5. Reaction from Intermediates

Preperation of Estimated Intermediate (16)

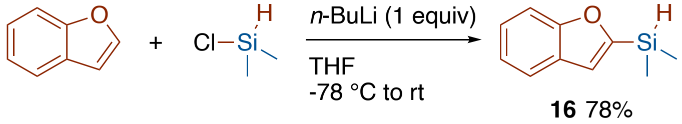

An oven dried 200 mL three-neck flask, equipped with a magnetic stirring bar and rubber septum was evacuated and backfilled with nitrogen. benzofuran (TCI, 1.33 g, 11.3 mmol) and THF (20 mL) were charged to the flask and cooled to -78 °C. *n*-BuLi (nacalai, 1.6 M in hexane, 7 mL, 1 equiv) was slowly added to the flask under -78 °C, then the mixture was stirred at -78 °C for 0.5 h. Chlorodimethylsilane (TCI, 1.89 g, 20.1 mmol, 1.8 equiv) was added to the flask dropwise, and the mixture was sttired at room temperature, overnight. Water was added to the flask. The organic products were extracted by Et_2O, washed with Brine, and dried over Na_2SO_4. Solvent was removed under reduced pressure. The resulting crude materials were purified by column chromatography on silica gel (SiliaFlash; eluent: hexane: Et_2O = 99: 1) and kügelrhor distillation. benzofuran-2-yldimethylsilane (**16**) (1.54 g, 8.8 mmol, 78% yield) was obtained as a colorless oil.

^{1}H NMR (400 MHz, $CDCl_3$) δ 7.59 (dt, J = 7.9, 1.1 Hz, 1H), 7.52 (dq, J = 8.2, 0.9 Hz, 1H), 7.32-7.27 (m, 1H), 7.22 (td, J = 7.4, 1.1 Hz, 1H), 7.05 (d, J = 0.9 Hz, 1H), 4.56-4.50 (m, 1H), 0.44 (d, J = 3.7 Hz, 6H).

^{13}C NMR (101 MHz, $CDCl_3$) δ 160.5, 158.3, 127.9, 124.7, 122.5, 121.1, 117.5, 111.4, -4.6

Iridium-Catalyzed Reaction from Estimated Intermediate (Figure 5C(b))

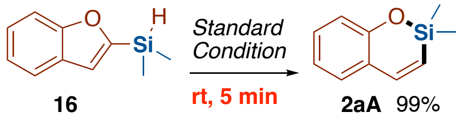

According to the the **General Procedure**, a mixture of **16** (104.3 mg, 0.3 mmol), $[IrCl(cod)]_2$ (8.3 mg, 0.006 mmol, 2 mol%) and (*S*)-DTBM-SEGPHOS (27.4 mg 0.012 mmol, 4 mol%) in toluene (1.4 mL) was stirred at room temperature (around 20 °C) for 5 minutes. **2aA** was detected in 99% NMR yield (diphenylmethane (TCI, 37.4 mg) as an internal standard).

Crossover Experiments

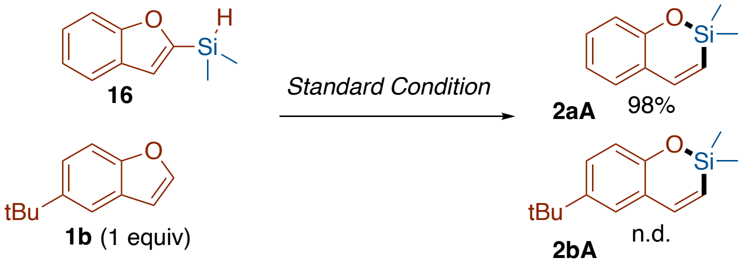

According to the the **General Procedure**, a mixture of **16** (21.3 mg, 0.12 mmol), benzofuran **1b** (20.3 mg, 0.12 mmol), $[IrCl(cod)]_2$ (1.7 mg, 0.0025 mmol, 2 mol%) and (*S*)-DTBM-SEGPHOS (8.1 mg, 0.0068 mmol, 4 mol%) in toluene (600 μL) was heated in 80 °C for 12 h then NMR measurement took place with diphenylmethane as an internal standard. **2aA** was obtained in 98% NMR yield and **2bA** was not detected while **2b** was completely recovered. This result indicates that **16** reacts perfectly in intramolecular manner without any intermolecular silylene exchange.

6.6. Deuterium Labeling Experiments

Synthesis of Benzofuran-*d* (1a-*d*)

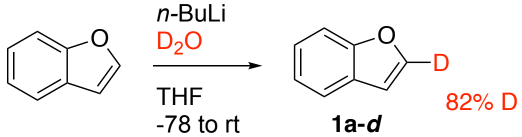

An oven dried 200 mL three-neck flask, equipped with a magnetic stirring bar and rubber septum was evacuated and backfilled with nitrogen. Benzofuran (TCI, 1.46 g, 12.4 mmol) and THF (20 mL) were charged to the flask and cooled to -78 °C. *n*-BuLi (nacalai, 1.6 M in hexane, 8 mL, 1 equiv) was slowly added to the flask under -78 °C, then the mixture was stirred at -78 °C for 0.5 h. D_2O (Aldrich, 460 mg, 23 mmol, 2 equiv) was added to the flask dropwise, and the mixture was sttired at room temperature, overnight. Water was added to the flask. The organic products were extracted by Et_2O, washed with Brine, and dried over Na_2SO_4. Solvent was removed under reduced pressure. The resulting crude materials were purified by kügelrhor distillation. benzofuran-2-*d* (**1a-*d***, 82% D) (967.8 mg, 8.13 mmol, 66% yield) was obtained as a colorless oil.

^{1}H NMR (400 MHz, CDCl$_3$) δ 7.64-7.62 (m, 1H), 7.54 (dd, J = 8.2, 0.9 Hz, 1H), 7.32 (td, J = 7.8, 1.4 Hz, 1H), 7.26 (td, J = 7.4, 1.1 Hz, 1H), 6.79 (dd, J = 2.7, 0.9 Hz, 1H)

^{13}C NMR (101 MHz, CDCl$_3$) δ 155.0, 145.1, 145.0, 144.8, 144.5, 127.5, 124.3, 122.8, 121.3, 111.5, 106.7, 106.5.

HRMS (EI) m/z: [M]+ Calcd. for C_8H_5DO, 119.0481; Found, 119.0477.

Synthesis of Triphenylsilane-*d* (J-*d*)

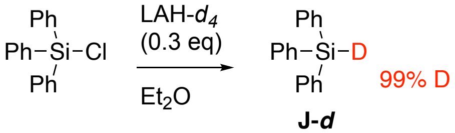

An oven dried 200 mL three-neck flask, equipped with a magnetic stirring bar and rubber septum was evacuated and backfilled with nitrogen. Triphenylchlorosilane (TCI, 1.81 g, 6.2 mmol) and Et_2O (15 mL) were charged to the flask and cooled to 0 °C. LAH-d_4 (Strem, 82 mg, 2.0 mmol, 0.3 equiv) was slowly added to the flask under 0 °C, then the mixture was stirred at room temperature overnight. Water was added to the flask. The organic products were extracted by Et_2O, washed with Brine, and dried over Na_2SO_4. Solvent was removed under reduced pressure. The resulting crude materials were purified by kügelrhor distillation. triphenylsilane-*d* (**J-*d***, > 99% D) (836.7 mg, 3.2 mmol, 52% yield) was obtained as a white solid.

^{1}H NMR (400 MHz, CDCl$_3$) δ 7.61-7.58 (m, 4H), 7.46-7.41 (m, 2H), 7.41-7.36 (m, 4H), 5.48 (s, 0.009H).

^{13}C NMR (101 MHz, CDCl$_3$) δ 135.9, 133.4, 129.9, 128.1.

HRMS (EI) m/z: [M]+ Calcd. for $C_{18}H_{15}DSi$, 261.1084; Found, 261.1076.

Synthesis of Intermediate-*d* (17aJ-*d*)

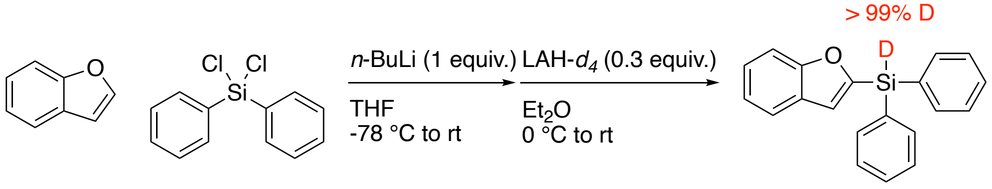

An oven dried 200 mL three-neck flask, equipped with a magnetic stirring bar and rubber septum was evacuated and backfilled with nitrogen. Benzofuran (TCI, 540.6 mg, 4.6 mmol) and THF (20 mL) were charged to the flask and cooled to -78 °C. *n*-BuLi (nacalai, 1.6 M in hexane, 3 mL, 1 equiv) was slowly added to the flask

under -78 °C, then the mixture was stirred at -78 °C for 0.5 h. dichlorodiphenylsilane (TCI, 2.6 g, 10.3 mmol, 2.2 equiv) was added to the flask dropwise, and the mixture was sttired at room temperature. Solvent was removed under reduced pressure after removing the insolvable solids by celite. Resulting crude mixture was used in the next step without further purification.

An oven dried 200 mL three-neck flask, equipped with a magnetic stirring bar and rubber septum was evacuated and backfilled with nitrogen. benzofuran-2-ylchlorodiphenylsilane and Et_2O (15 mL) were charged to the flask and cooled to 0 °C. LAH-d_4 (Strem, 70 mg, 1.7 mmol, 0.4 equiv) was slowly added to the flask under 0 °C, then the mixture was stirred at room temperature overnight. Water was added to the flask. The organic products were extracted by Et_2O, washed with Brine, and dried over Na_2SO_4. Solvent was removed under reduced pressure. The resulting crude materials were purified by kügelrhor distillation. benzofuran-2-yldiphenylsilane-*d* (**17aJ-*d***, > 99% D) (720.1 mg, 2.39 mmol, 52% yield over 2 steps) was obtained as a white solid.

^{1}H NMR (400 MHz, CDCl$_3$) δ 1H-NMR (400 MHz, CHLOROFORM-D) δ 7.71-7.68 (m, 4H), 7.59 (dd, J = 7.8, 1.4 Hz, 1H), 7.54 (dq, J = 8.2, 0.9 Hz, 1H), 7.50-7.45 (m, 2H), 7.44-7.40 (m, 4H), 7.34-7.30 (m, 1H), 7.25-7.21 (m, 1H), 7.14 (d, J = 0.9 Hz, 1H), 5.57 (s, 0.015H).

^{13}C NMR (101 MHz, CDCl$_3$) δ 158.9, 156.9, 135.8, 131.3, 130.4, 128.3, 127.7, 125.1, 122.7, 121.4, 120.9, 111.7.

HRMS (EI) m/z: [M]+ Calcd. for $C_{20}H_{15}DOSi$, 301.1033; Found, 301.1030.

Reaction of 17aJ-*d* (Figure 5C(c))

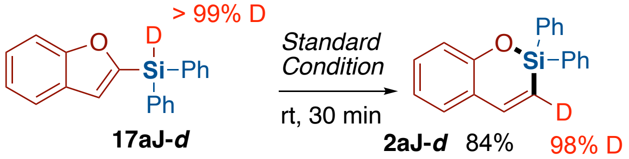

According to the **GP**, a mixture of hydrosilane **17aJ-*d*** (33.4 mg, 0.11 mmol), $[IrCl(cod)]_2$ (1.54 mg, 0.002 mmol, 2 mol%) and (*S*)-DTBM-SEGPHOS (6.5 mg, 0.004 mmol, 4 mol%) in toluene (0.2 mL) was stirred for 30 minutes at room temperature. **2aJ-*d*** was obtained as white solid in 84% yield (29.1 mg) after purification by column chromatography on silica gel (SiliaFlash; eluent: hexane : Et_2O = 95 : 5). The H/D ratio was determined based on the 7-hydrogen on oxasiline ring. Perfect deuterium incorporation in 2aJ-*d* 3-position carbon was detected, indicating no H/D exchange is occurring during second step, intramolecular tautomerization and after the reaction.

^{1}H NMR (400 MHz, CDCl$_3$) δ 7.67 (dt, J = 6.1, 1.6 Hz, 4H), 7.55 (s, 1H), 7.49-7.40 (m, 6H), 7.24-7.17 (m, 2H), 7.04 (d, J = 8.2 Hz, 1H), 6.97 (td, J = 7.4, 1.1 Hz, 1H), 6.25 (d, J = 14.2 Hz, 0H).

^{13}C NMR (101 MHz, CDCl$_3$) δ 153.6, 147.5, 134.7, 134.5, 130.9, 130.7, 129.9, 128.2, 124.3, 121.1, 119.7, 119.6, 119.4 (t, J = 22 Hz, sp^2*C*D).

HRMS (EI) m/z: [M]+ Calcd. for $C_{20}H_{21}ONSi$, 319.1392; Found, 319.1390.

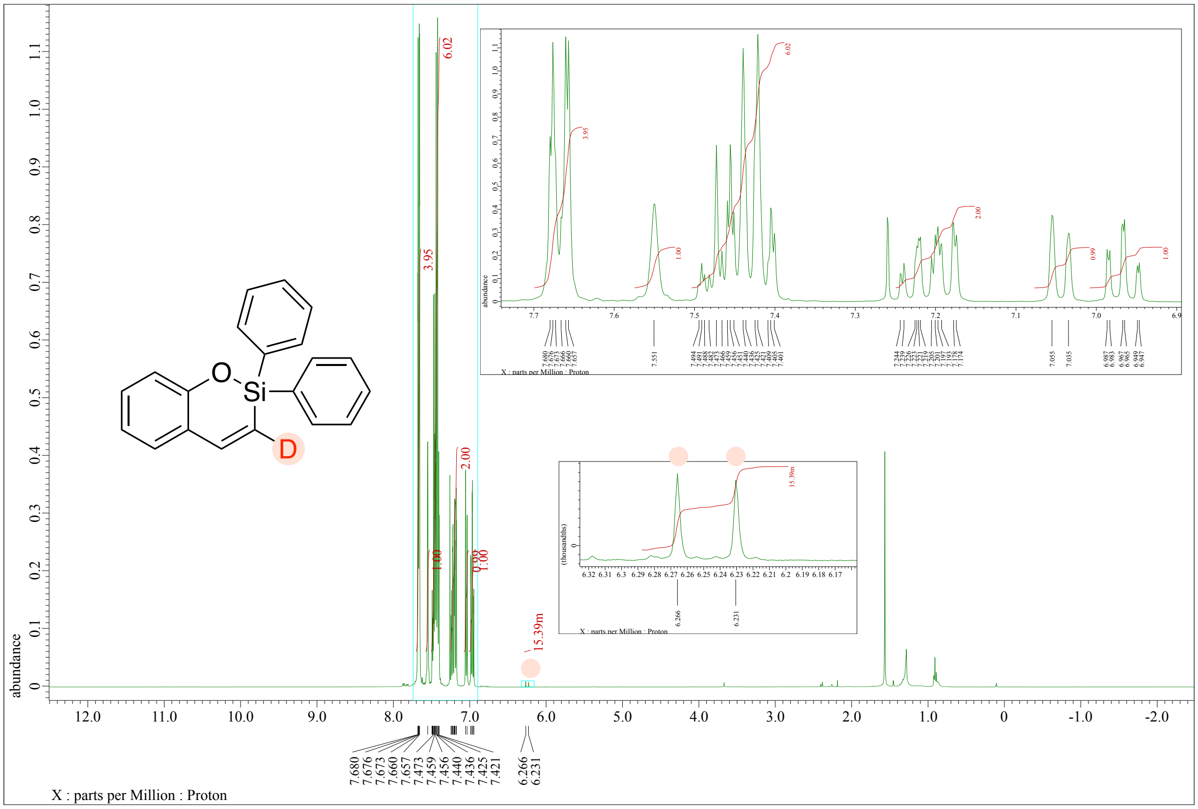

Figure S18. H NMR of **2aK-*d***.

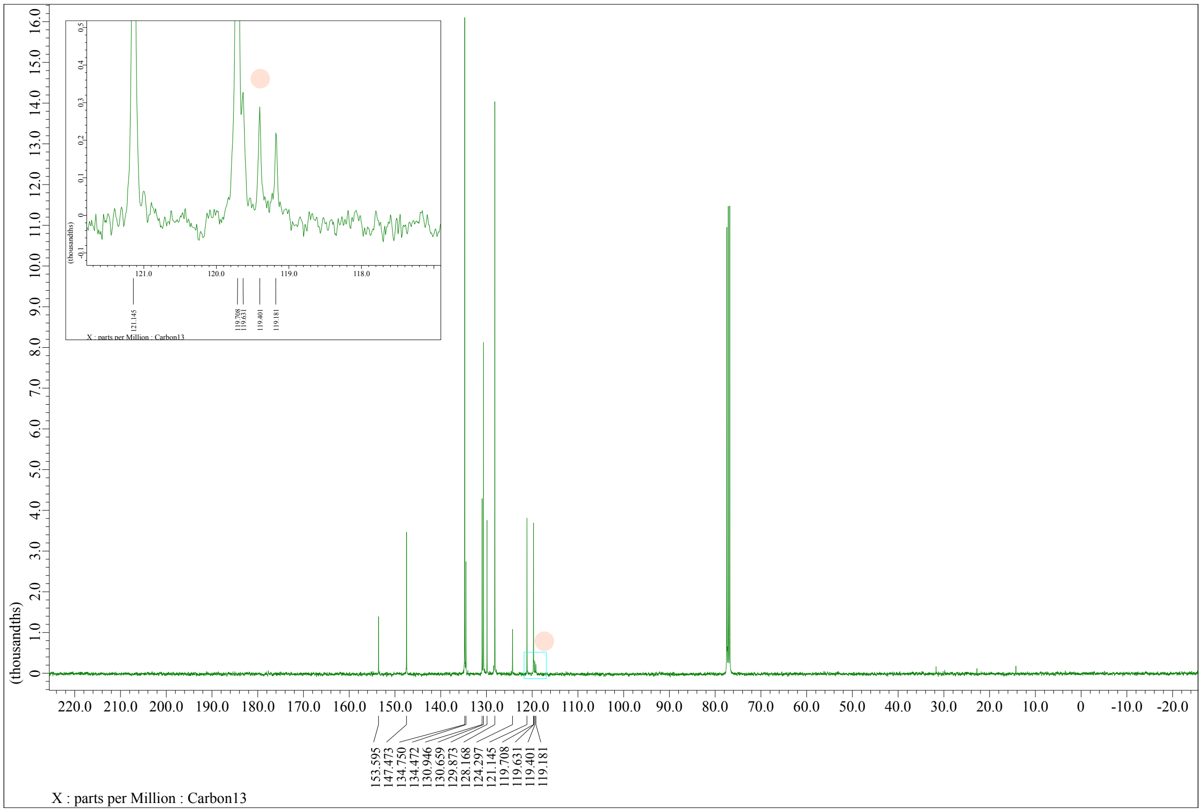

Figure S19. C NMR of **2aK-*d***.

Reaction of 2a-*d* and J (Figure 5C(d))

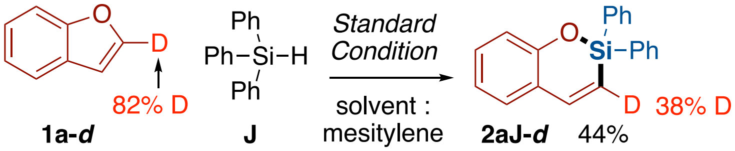

According to the **GP**, a mixture of deuterated benzofuran **1a-*d*** (16.65 mg, 0.14 mmol), hydrosilane **J** (36.91 mg, 0.14 mmol, 1 equiv.), [IrCl(cod)]$_2$ (1.53 mg, 0.0023 mmol, 1.6 mol%) and (*S*)-DTBM-SEGPHOS (5.4 mg, 0.0046 mmol, 3.2 mol%) in mesitylene (0.2 mL) was heated at 80 °C for 12 h. **2aJ-*d*** was obtained as white solid in 44% yield (18.6 mg, 0.062 mmol) after purification by column chromatography on silica gel (SiliaFlash; eluent: hexane : Et$_2$O = 90 : 10).

^{1}H NMR (400 MHz, CDCl$_3$) *δ* 7.64 (td, J = 3.9, 2.1 Hz, 4H), 7.53 (d, J = 14.0 Hz, and s, 1H), 7.47-7.38 (m, 6H), 7.22-7.15 (m, 2H), 7.02 (d, J = 8.2 Hz, 1H), 6.94 (td, J = 7.3, 1.4 Hz, 1H), 6.22 (d, J = 14.2 Hz, 0.62H).

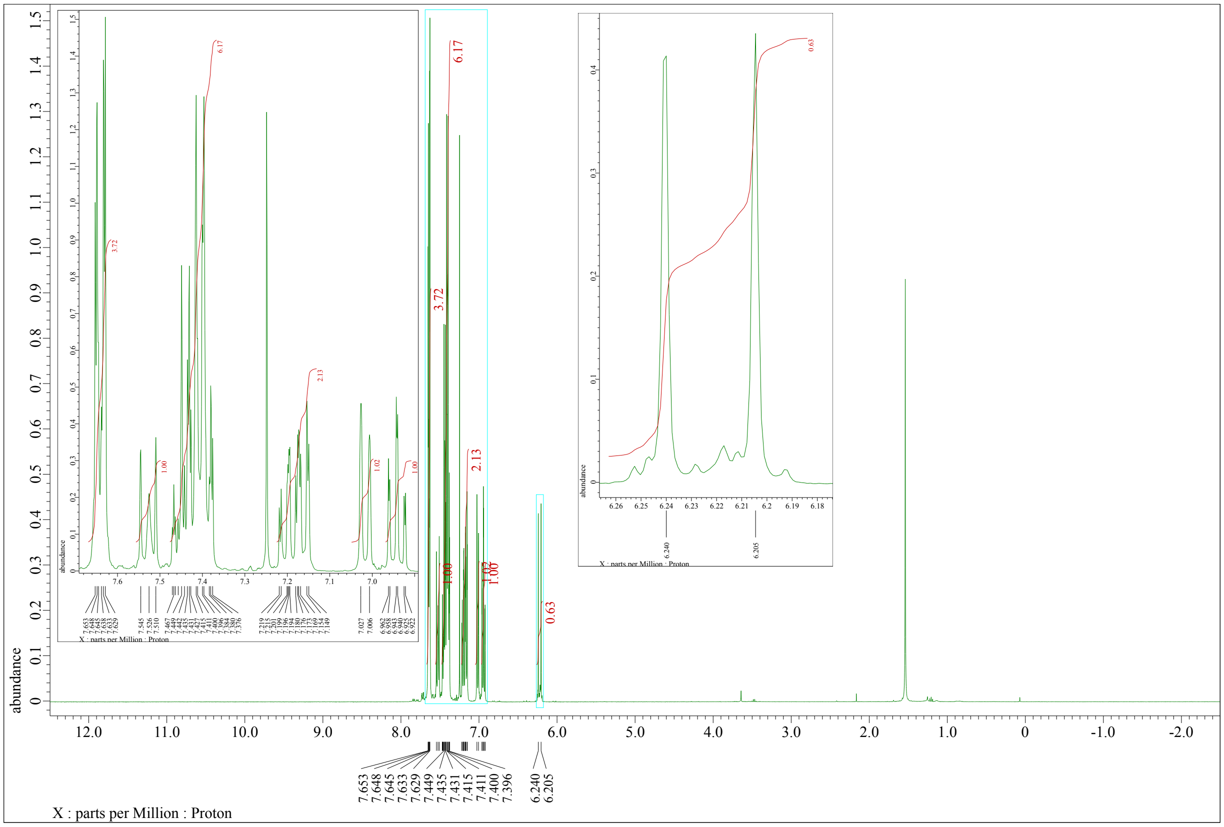

Figure S20. H NMR of **2aK-*d***.

Reaction of 2a and J-*d*

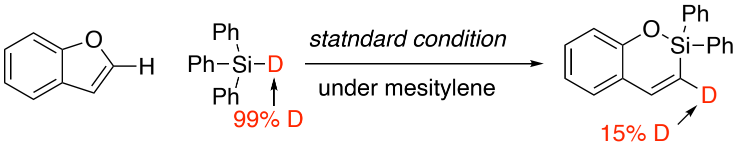

According to the **GP**, a mixture of benzofuran **1a** (18.43 mg, 0.156 mmol, 1.2 equiv.), deuterated hydrosilane **J-*d*** (32.41 mg, 0.125 mmol), $[IrCl(cod)]_2$ (1.61 mg, 0.0024 mmol, 2 mol%) and (*S*)-DTBM-SEGPHOS (6.55 mg, 0.0056 mmol, 4 mol%) in mesitylene (0.2 mL) was heated at 80 °C for 12 h. **2aJ-*d*** was obtained as white solid in 78% yield (25.2 mg, 0.097 mmol) after purification by column chromatography on silica gel (SiliaFlash; eluent: hexane : Et_2O = 90 : 10).

^{1}H NMR (400 MHz, $CDCl_3$) *δ* 7.65 (dt, J = 6.3, 1.7 Hz, 4H), 7.54 (d, J = 14 Hz, and s, 1H), 7.48-7.39 (m, 6H), 7.23-7.16 (m, 2H), 7.03 (d, J = 7.8 Hz, 1H), 6.95 (td, J = 7.4, 1.1 Hz, 1H), 6.23 (d, J = 14.2 Hz, 0.85H).

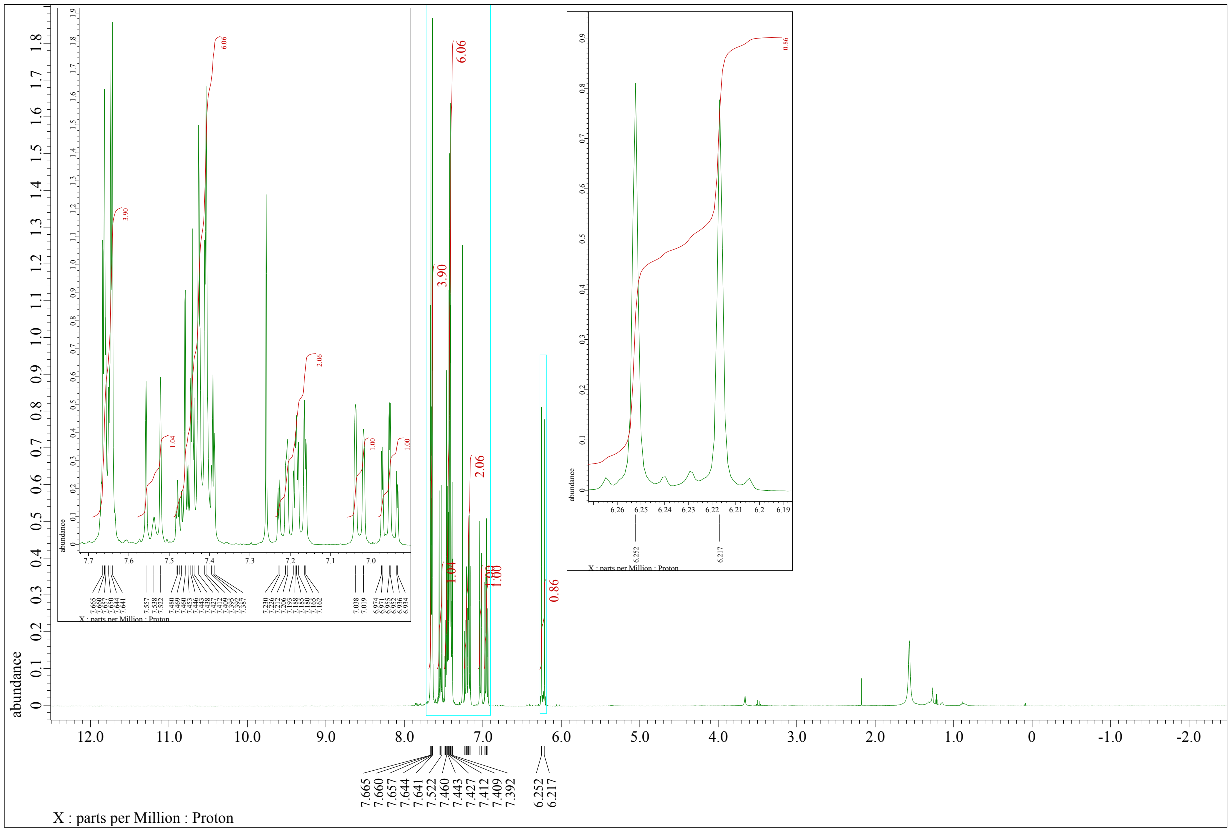

Figure S21. H NMR of **2aK-*d***.

7. DFT Calculation

Geometrical optimizations were conducted by the DFT method with the B3LYP function in the gas phase with 6-31G(d) basis sets for H, C, O, Cl, Si and P atoms, and LANL2DZ basis sets for Ir atom with corresponding effective core potentials (ECPs). The potential energy was re-evaluated using the B3LYP-D3 functional with 6-311++G(d,p) basis sets for H, C, O, Cl, Si and P atoms, and SDD basis sets for Ir atom. The solvation effect of toluene was considered using the SMD model. Vibrational frequency calculations were performed for all the stationary points to confirm if each optimized structure is a local minimum or a transition state structure. All these calculations were carried out with the Gaussian 16 program.

For the simplification, (S)-SEGPHOS ligand is used in the calculation, since tert-butyl group of DTBM group on DTBM-SEGPHOS rotates freely. We have already checked the reaction from **16** proceeds well even with Ir/(*S*)-SEGPHOS catalyst condition.[27]

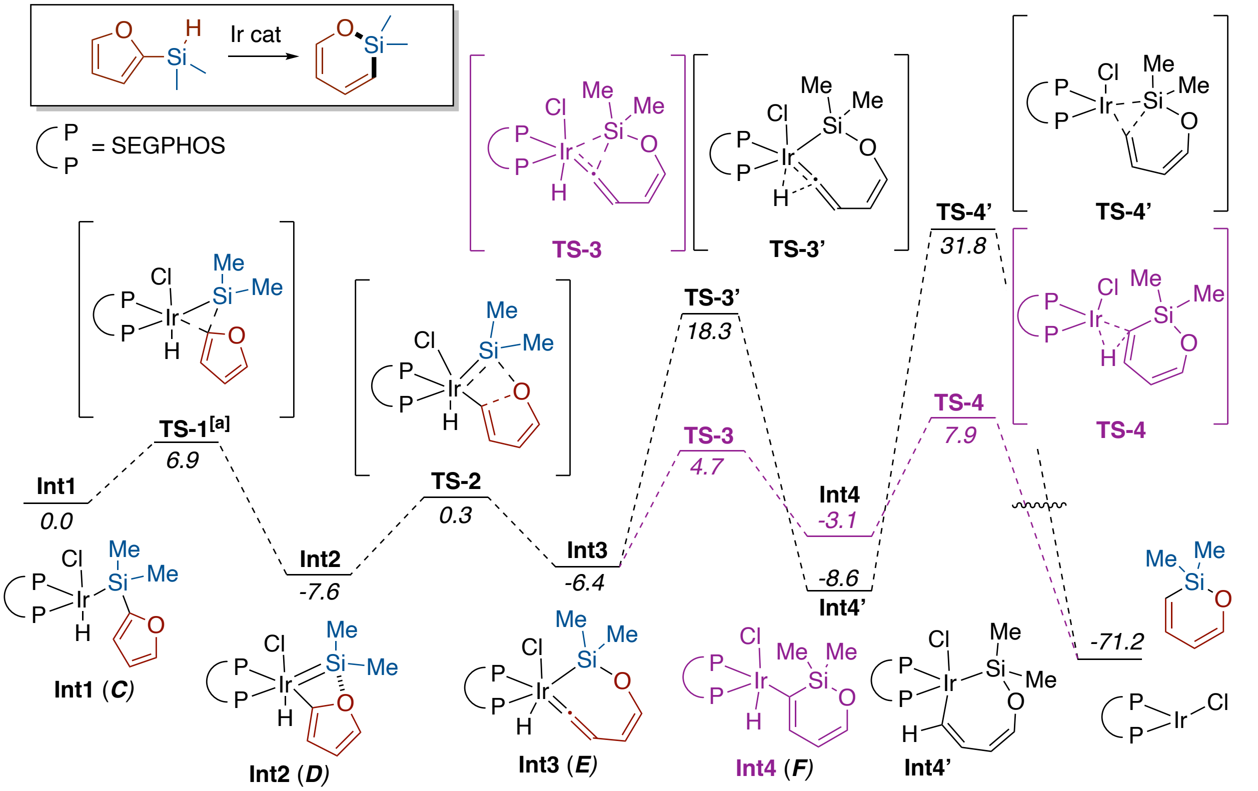

Figure S22. (Figure 5B) DFT Calculation of second step. The highest energy barrier is 15.5 kcal/mol (**Int2-TS-4**), which is reasonable for the reaction proceeds under room temperature.

▪Structures in DFT Calculation

Int 1

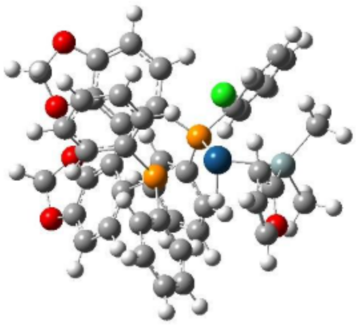

P	-0.22094700	1.76248300	0.03635600
P	0.21669000	-1.80199100	-0.00505800
O	-4.61409000	-0.72810800	-3.34858600
O	-4.73101400	-0.24362900	3.42103200
O	-4.33546400	-1.29286600	1.40587800
O	-4.46492600	0.36992200	-1.32525200
C	0.43145700	3.24723000	0.94315900
C	0.17287500	4.55880300	0.52782500
H	-0.42402100	4.74104800	-0.35580400
C	0.67525900	5.64392200	1.24541900
C	1.70349200	4.13588300	2.81195200
C	1.22344900	3.05105200	2.08381800
H	1.46496300	2.04404900	2.40029900
C	-1.46073300	-2.24732600	-2.20924200
H	-0.70428600	-2.95892700	-2.50338700
C	-1.13722800	2.53801800	-1.37315400
C	-1.60872200	1.19246200	1.13122600
C	-0.36677400	-2.29301300	1.68024300
C	-0.51450100	2.61702600	-2.62418800
H	0.45130300	2.15172500	-2.77433700
C	-1.15498400	3.25107900	-3.68806200
C	-2.41448200	3.09234400	-1.20940600
H	-2.91503800	3.03012200	-0.25100600
C	1.43071900	5.43784000	2.39583600
C	-2.52984400	-2.03327200	-3.09026600
H	-2.60476100	-2.56378000	-4.03006300
C	-2.32542300	-0.62488800	-0.59684900
C	-3.36718400	-0.44140200	-1.48760000
C	-3.46801900	-1.10855800	-2.70393700
C	0.79566200	-4.60130800	0.06864600
H	0.26046300	-4.62992800	1.00741200
C	0.43547900	-2.06644400	2.80274600
H	1.36506200	-1.52438800	2.69257300
C	-1.15466500	-3.22166100	4.20669900
C	-1.56846800	-2.99539100	1.83787800
H	-2.20716600	-3.17892900	0.98283300
C	-5.11212000	0.38908900	-2.60120700
H	-6.18764700	0.28044000	-2.46307100
H	-4.85790300	1.31859800	-3.12401800
C	-1.33401900	-1.57545800	-0.99157200
C	-1.86726100	1.81727300	2.35370500
H	-1.26054900	2.65803200	2.65707500
C	-2.90140200	1.41248700	3.21197400
H	-3.08143500	1.90739800	4.15732800
C	-3.67757100	0.36250500	2.78614300
C	-3.43768800	-0.26424000	1.56941100
C	-2.41343300	0.09401300	0.71138300
C	-5.30831200	-1.11021000	2.43754400
H	-5.54054400	-2.07213900	2.89324400
H	-6.20307700	-0.63631700	2.01429300
C	0.93992800	-3.39728400	-0.63322800
C	1.65804400	-3.40035400	-1.83811900
H	1.79677200	-2.47361000	-2.38317700
C	2.19457300	-4.58519700	-2.33520000
C	1.34817800	-5.78120600	-0.42758400
C	2.04342100	-5.77918900	-1.63285100
C	-2.41937200	3.80864100	-3.51682200
C	-3.04935800	3.72547200	-2.27490500
C	0.04408500	-2.52874200	4.05726600
C	-1.96019100	-3.45250800	3.09364600
C	4.66654700	-1.70897500	-1.32656500
H	4.35132000	-1.32582200	-2.29848200
H	5.73672900	-1.51609700	-1.19847000
H	4.51759400	-2.79180900	-1.31959800
C	4.32459300	-1.56349200	1.71695000
H	3.97486100	-2.58962200	1.86749500
H	5.41874500	-1.58117200	1.74305000
H	3.97363100	-0.95769100	2.55511800
H	0.46517100	6.65189200	0.90483400
H	1.80985000	6.28293900	2.95973700
H	2.29961800	3.96122000	3.70104300
H	-0.66435700	3.29733800	-4.65377000
H	-2.91421400	4.30307300	-4.34584900
H	-4.03398600	4.15812100	-2.13249800
H	-2.89521200	-3.99136600	3.19913300
H	-1.46055100	-3.57757000	5.18418900
H	0.67711300	-2.34351600	4.91780400
H	1.23174000	-6.70141000	0.13421700
H	2.46965800	-6.69854600	-2.01895800
H	2.74090500	-4.56778100	-3.27166100

Ir	1.52902600	0.01907300	-0.16317600
H	1.55286600	0.09805900	1.40782600
Cl	1.65392200	-0.05249200	-2.72915700
Si	3.69481700	-0.88692600	0.06689300
C	3.94524000	0.99600600	0.02445400
C	3.74481600	1.94585900	-0.96508900
O	4.33683900	1.67027500	1.17784000
C	4.05790000	3.22247000	-0.41085900
H	3.45413800	1.72025100	-1.97851500
C	4.40945300	2.98851200	0.88027700
H	4.01507200	4.18116100	-0.90192200
H	4.72066100	3.63634700	1.68313500

Int 2

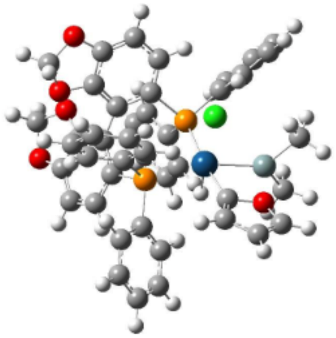

P	-0.16248600	-1.74536100	-0.16928600
P	0.08774400	1.78597300	-0.02067400
O	-4.41797200	0.52196200	3.69472800
O	-5.04484200	0.19054300	-3.04475100
O	-4.54045100	1.15924000	-1.01348300
O	-4.37916100	-0.55872200	1.65677200
C	0.39072800	-3.20519400	-1.18226000
C	0.02783400	-4.51841400	-0.86072600
H	-0.58538800	-4.71591100	0.00835500
C	0.45563000	-5.58739300	-1.64766700
C	1.61000100	-4.05704300	-3.10173200
C	1.19713000	-2.99105500	-2.30845100
H	1.51265800	-1.98520700	-2.55197000
C	-1.41931500	2.17149300	2.33199200
H	-0.67189200	2.91178700	2.57396800
C	-0.86096300	-2.57436800	1.32833000
C	-1.67906200	-1.18401400	-1.08398500
C	-0.68962900	2.28944300	-1.62492900
C	-0.02085800	-2.78998100	2.42722400
H	0.98095700	-2.38140000	2.42921000
C	-0.48693100	-3.48839700	3.53939000
C	-2.17477800	-3.05979600	1.36662300
H	-2.84120500	-2.89450700	0.52947700
C	1.24287100	-5.36060900	-2.77262300
C	-2.40882600	1.90826100	3.29040400
H	-2.43297200	2.42965800	4.23801600
C	-2.34258600	0.52958700	0.77630500
C	-3.30366500	0.29464100	1.74275200
C	-3.33632800	0.94913100	2.96885900
C	0.66170700	4.59462600	-0.16700900
H	0.08500100	4.61168400	-1.08128000
C	-0.04579400	2.00204700	-2.83190800
H	0.86762100	1.42126900	-2.81936600
C	-1.75893400	3.18035100	-4.06052700
C	-1.87991200	3.02837800	-1.65288000
H	-2.39952000	3.25239600	-0.72900700
C	-4.92530000	-0.60968800	2.97891800
H	-6.01191100	-0.54553700	2.92380400
H	-4.59462800	-1.53097700	3.47319900
C	-1.36166900	1.51389800	1.10288200
C	-2.00184600	-1.75559400	-2.31821500
H	-1.37689500	-2.54141900	-2.71636000
C	-3.12075100	-1.36736800	-3.07023500
H	-3.34739300	-1.82072100	-4.02628100
C	-3.91554200	-0.38812700	-2.52688300
C	-3.61085500	0.18775100	-1.29968600
C	-2.50428000	-0.15519200	-0.54281400
C	-5.58064500	0.98167300	-1.97693800
H	-5.88767400	1.95268100	-2.36413900
H	-6.41962800	0.44698500	-1.51376800
C	0.82116500	3.40169000	0.55017700
C	1.59415800	3.42092900	1.72195400
H	1.74838800	2.50173200	2.27739400
C	2.16467700	4.60908000	2.17266400
C	1.24947300	5.77735200	0.28062400
C	1.99609100	5.79144300	1.45474900
C	-1.79135300	-3.97485000	3.56963100
C	-2.63551800	-3.75669700	2.48146600
C	-0.57621900	2.44603500	-4.04204300
C	-2.41061400	3.46843400	-2.86225900
Si	3.66132200	1.07852600	-0.20839400
C	4.69554000	1.80792900	1.18201400
H	4.41152000	1.36692500	2.13878400
H	5.76305000	1.64088700	1.00448700
H	4.53495700	2.88835000	1.23917900
C	4.31677700	1.77325700	-1.83614200
H	3.91320800	2.78165700	-1.98077500
H	5.40815900	1.85246300	-1.84835100
H	3.99508600	1.16494200	-2.68404400

H	0.16943600	-6.59778300	-1.37697800
H	1.57405900	-6.19240900	-3.38447900
H	2.23298200	-3.86916400	-3.96919800
H	0.17170400	-3.63859000	4.38723300
H	-2.15080900	-4.51780700	4.43711700
H	-3.65213800	-4.13527000	2.49453000
H	-3.33350300	4.03793300	-2.86720600
H	-2.17374800	3.52242400	-5.00219300
H	-0.06621000	2.21211800	-4.96993600
H	1.11778900	6.68806500	-0.29334500
H	2.44661200	6.71361600	1.80501900
H	2.74865900	4.60360600	3.08649300
Ir	1.61348000	-0.05626500	-0.01590400
H	1.63295000	-0.09513900	-1.59230100
Cl	1.75430500	0.05731100	2.56392100
C	3.12574300	-1.45047700	0.02595500
C	3.51506500	-2.73011600	0.26574100
C	4.95093700	-2.83264300	0.18140200
H	2.84628100	-3.54372700	0.49253300
C	5.44942100	-1.61657100	-0.11421800
H	5.53915800	-3.72626500	0.32469200
H	6.42863600	-1.20400800	-0.27871400
O	4.36761100	-0.74537400	-0.23632900

Int 3

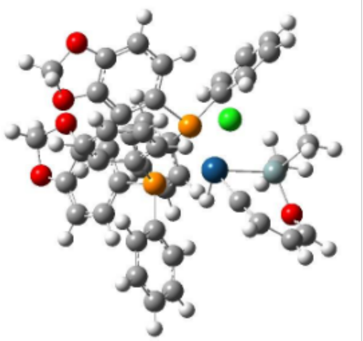

P	-0.46869000	1.76735300	0.05599900
P	0.33943000	-1.69558900	-0.03464100
O	-4.64068200	-1.18208300	-3.31024400
O	-4.66466900	-0.75148600	3.48441600
O	-4.23391200	-1.71051500	1.43118400
O	-4.60633700	-0.12037300	-1.25987600
C	-0.05330300	3.36564600	0.91251800
C	-0.63540900	4.58123600	0.53204200
H	-1.33850200	4.61578300	-0.28889200
C	-0.31152900	5.76293200	1.19722200
C	1.17745100	4.54551400	2.64200700
C	0.86722600	3.36664600	1.97025000
H	1.35228600	2.44460900	2.26016300
C	-1.26866800	-2.23919400	-2.27655600
H	-0.42420100	-2.82533400	-2.60515200
C	-1.43189000	2.35518800	-1.40569500
C	-1.76276400	1.02514300	1.15219400
C	-0.23959700	-2.26367600	1.62800500
C	-0.76101700	2.56870900	-2.61511000
H	0.27915200	2.28562800	-2.71344400
C	-1.44253000	3.10132200	-3.70820600
C	-2.79322200	2.67533100	-1.30981100
H	-3.32858600	2.50906200	-0.38336100
C	0.59013800	5.74893300	2.25710800
C	-2.37490300	-2.15645100	-3.13365800
H	-2.38795900	-2.65979000	-4.09112800
C	-2.32974900	-0.81442900	-0.59907500
C	-3.40867900	-0.76309100	-1.46415100
C	-3.43585800	-1.40244400	-2.69772600
C	1.13958400	-4.44659700	-0.03465000
H	0.59993200	-4.55035200	0.89603600
C	0.43196200	-1.84941800	2.78184700
H	1.24917000	-1.14525600	2.69940900
C	-1.00648800	-3.22590300	4.14844300
C	-1.30712500	-3.16355500	1.75286900
H	-1.85154200	-3.48448200	0.87302400
C	-5.29688200	-0.18978700	-2.51262700
H	-6.33043800	-0.49029800	-2.33922100
H	-5.23345800	0.78135500	-3.01669300
C	-1.22067200	-1.60011100	-1.03634700
C	-2.03549600	1.59005100	2.40066300
H	-1.49850200	2.47497100	2.71037300
C	-3.00067400	1.06779500	3.27471200
H	-3.19503700	1.51657400	4.23989700
C	-3.69178900	-0.03624100	2.83820600
C	-3.43281300	-0.60764800	1.59764300
C	-2.47138400	-0.13450400	0.72308900
C	-5.18175700	-1.65550100	2.50008000
H	-5.29179000	-2.64607000	2.94015200
H	-6.13724600	-1.27254000	2.12053700
C	1.19090000	-3.21254100	-0.69532700
C	1.92782500	-3.11488300	-1.88450500
H	1.99005900	-2.16369800	-2.40121800
C	2.57937400	-4.23103300	-2.40422400
C	1.80147100	-5.55712900	-0.55518600
C	2.52003900	-5.45524500	-1.74283000
C	-2.79266400	3.42557100	-3.60653000

C	-3.46738500	3.20966400	-2.40497900
C	0.05124500	-2.32825700	4.03412700
C	-1.68645200	-3.64034500	3.00418400
Si	3.87180300	-0.59193200	0.18550700
C	4.83706300	-0.84525600	-1.40847300
H	4.89237900	0.08406400	-1.97850500
H	5.85279000	-1.18278400	-1.17555600
H	4.36162000	-1.58825500	-2.04978500
C	3.96324600	-2.13467000	1.26365200
H	3.44453400	-2.98552800	0.82117100
H	5.01767900	-2.40079200	1.38852700
H	3.54634500	-1.95400400	2.25687900
H	-0.76807700	6.69445100	0.88152900
H	0.84032600	6.66870300	2.77396900
H	1.89223100	4.52260700	3.45682300
H	-0.91327400	3.25203800	-4.64213300
H	-3.31890300	3.84215800	-4.45851300
H	-4.51792600	3.46497700	-2.31526400
H	-2.51365500	-4.33698600	3.08364300
H	-1.30332300	-3.59721800	5.12294300
H	0.58163800	-1.99525800	4.91906500
H	1.75557600	-6.50154400	-0.02428500
H	3.03449900	-6.32051200	-2.14589300
H	3.14091600	-4.13508300	-3.32684400
Ir	1.62723000	0.42632600	-0.13366000
H	1.62494800	0.50686000	1.43934600
Cl	1.69783000	0.22120100	-2.70279400
C	2.82268400	1.84751400	-0.20885000
C	3.74282300	2.78243700	-0.22951100
C	5.09334900	2.66269900	0.31096800
H	3.43822800	3.72274000	-0.68674300
C	5.56289400	1.56876500	0.93535100
H	5.74506600	3.52208300	0.22201900
H	6.56403000	1.56755800	1.36023500
O	4.89202200	0.42489500	1.14949600

Int 4

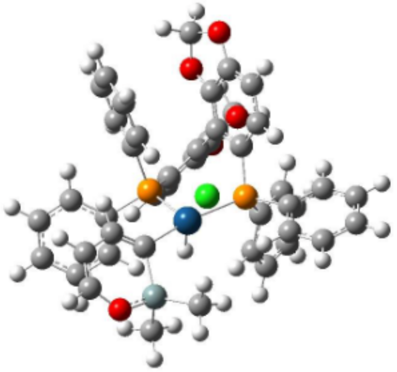

P	0.48454700	-1.43333900	-0.23882900
P	-0.80118800	1.78660100	0.01935200
O	-4.38220200	-1.36167600	3.58475700
O	-4.68765600	-1.75117100	-3.22434400
O	-4.72431200	-0.75332400	-1.14617700
O	-3.93096900	-2.23126600	1.49314900
C	1.62162600	-2.55510600	-1.17734200
C	1.88103300	-3.86708400	-0.77052700
H	1.43120400	-4.26103300	0.13025400
C	2.74144600	-4.67942900	-1.50867300
C	3.09988700	-2.88567500	-3.07154600
C	2.25386100	-2.06938900	-2.32939600
H	2.09721000	-1.04353300	-2.63653800
C	-2.29449300	1.41474600	2.35740300
H	-1.87184100	2.36511400	2.65164100
C	0.06465100	-2.36226700	1.30398400
C	-1.10499000	-1.51008100	-1.19144600
C	-1.60203700	1.97077500	-1.64278100
C	0.54510700	-1.94125800	2.54761100
H	1.12506200	-1.03007000	2.62753900
C	0.25563900	-2.67860800	3.69456400
C	-0.72492900	-3.51916800	1.22837800
H	-1.13016600	-3.84295900	0.27718000
C	3.34716000	-4.19553100	-2.66286500
C	-3.08030100	0.72069700	3.28884200
H	-3.28230500	1.12320100	4.27256100
C	-2.51371900	-0.37056400	0.69698600
C	-3.29940000	-1.01987500	1.63264500
C	-3.57264400	-0.49937800	2.89265800
C	-2.05722000	4.25747000	0.67439100
H	-2.99887400	3.74869300	0.50975400
C	-0.77507000	2.13540300	-2.75871600
H	0.29964900	2.06190900	-2.64176900
C	-2.70082000	2.47936600	-4.17292900

C	-2.98891000	2.06019900	-1.81088200
H	-3.65121600	1.91548700	-0.96581900
C	-4.43027900	-2.55588600	2.79600700
H	-5.46352900	-2.89385700	2.71314700
H	-3.78703000	-3.31818500	3.25031600
C	-2.02127600	0.91120000	1.08379000
C	-1.10579900	-2.10022100	-2.45921800
H	-0.18304500	-2.48964500	-2.86281600
C	-2.26433700	-2.23848000	-3.23658900
H	-2.23867000	-2.69306600	-4.21796500
C	-3.43173800	-1.77056600	-2.68465200
C	-3.44918600	-1.18146800	-1.42539200
C	-2.32458400	-1.00931700	-0.63949400
C	-5.54988300	-1.34058600	-2.15427500
H	-6.25752500	-0.59936500	-2.52337000
H	-6.06056400	-2.21977500	-1.74268500
C	-0.84666100	3.55812200	0.56427300
C	0.35100500	4.24685200	0.77402700
H	1.29380600	3.72118100	0.71942400
C	0.34047400	5.60546300	1.08795000
C	-2.06628900	5.60995100	0.99748500
C	-0.86530000	6.28868200	1.20290600
C	-0.51002500	-3.83850100	3.61209500
C	-1.00330500	-4.25602500	2.37604900
C	-1.32032700	2.39087500	-4.01511200
C	-3.53303100	2.30974400	-3.06797600
Si	4.50758000	1.05118800	-0.45945400
C	4.55043700	0.98047300	-2.34394500
H	4.67856600	-0.04606900	-2.69595000
H	3.63195100	1.38286000	-2.78194300
H	5.38858000	1.57409500	-2.72284500
C	4.41015600	2.85353000	0.06016500
H	5.32284400	3.38124400	-0.23466200
H	3.56553100	3.35926100	-0.41973900
H	4.28728200	2.94226000	1.14169700
H	2.94028500	-5.69032200	-1.17109200
H	4.01771100	-4.82743700	-3.23409700
H	3.57923900	-2.49220600	-3.96073300
H	0.63085800	-2.33746300	4.65239000
H	-0.72602200	-4.41323500	4.50609800
H	-1.60653900	-5.15432600	2.30272100
H	-4.60949800	2.37432800	-3.18329200
H	-3.12643500	2.67666600	-5.15040800
H	-0.66492600	2.51834400	-4.86945100
H	-3.01106100	6.13504800	1.08437800
H	-0.87286100	7.34397400	1.45207000
H	1.27946900	6.12155100	1.25201900
Ir	1.31798600	0.59338200	0.19057100
C	3.26279000	-0.08778700	0.35399500
C	3.78383300	-1.13619600	1.03503000
C	5.20047300	-1.43983000	1.21873000
H	3.11941500	-1.84390200	1.52915700
C	6.20389900	-0.66836000	0.76812900
H	5.46612400	-2.33012700	1.77710600
H	7.24968200	-0.90061100	0.94147100
O	6.02938100	0.47546000	0.06542200
Cl	1.62123600	1.54571700	2.45410000
H	1.43972000	0.57313500	-1.37336200

Int 4’

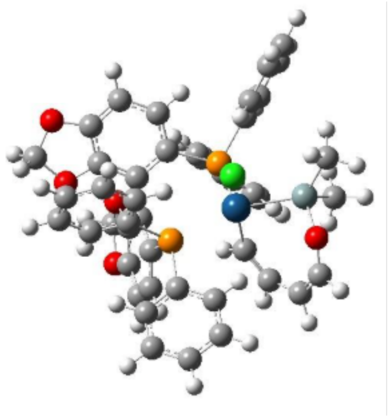

P	-1.13434400	1.62394300	-0.11285400
P	1.00725200	-1.36448900	-0.25662200
O	-4.31203300	-2.84605000	-2.56206700
O	-3.32181700	-1.92332900	4.14350500
O	-2.83100800	-2.86234900	2.09103900
O	-4.31367100	-1.74756000	-0.52670200
C	-1.23619200	3.38205700	0.45423500
C	-2.45798000	4.05175900	0.61422500
H	-3.39120800	3.53562500	0.42629500
C	-2.48672000	5.38811100	0.99966500
C	-0.07821600	5.42836500	1.05083000
C	-0.04849000	4.08775000	0.67114500
H	0.91041400	3.60016500	0.55047700
C	-0.67378100	-2.44877300	-2.22326100
H	0.25497200	-2.68560300	-2.71906700
C	-2.39977900	1.61472800	-1.46750200
C	-1.91686700	0.61297500	1.21457000
C	1.02439300	-2.38634000	1.29099900
C	-1.99358400	1.89737900	-2.77761000
H	-0.94184200	2.03615100	-2.99591100

C	-2.93699800	1.98833400	-3.79938300
C	-3.76203300	1.41981100	-1.20251600
H	-4.09355000	1.18027600	-0.19949500
C	-1.29576600	6.07993800	1.21883700
C	-1.86211600	-2.84566400	-2.85200600
H	-1.85570600	-3.35813500	-3.80467500
C	-1.87402700	-1.47103300	-0.32791200
C	-3.02412000	-1.89500400	-0.96922000
C	-3.03089100	-2.55523000	-2.19330100
C	2.82136400	-3.46912000	-0.91276500
H	2.59175700	-3.89283600	0.05500500
C	1.88472300	-2.08015500	2.35066300
H	2.47095300	-1.17472700	2.32133400
C	1.22176900	-4.09356600	3.50989100
C	0.26240500	-3.56106700	1.35711900
H	-0.40925600	-3.82221700	0.55030600
C	-5.15615500	-2.22763000	-1.57990100
H	-5.84858100	-2.97023800	-1.17958200
H	-5.68381600	-1.38548200	-2.03784300
C	-0.64561800	-1.78362300	-0.99373800
C	-2.22426000	1.16443100	2.45815900
H	-2.08398100	2.22523000	2.61395500
C	-2.69880600	0.38923300	3.52873800
H	-2.93867900	0.82954700	4.48765600
C	-2.84419000	-0.95705400	3.29768000
C	-2.54115700	-1.52123800	2.06176600
C	-2.05789400	-0.78958300	0.99258100
C	-3.11653400	-3.16607300	3.46122000
H	-2.25955500	-3.68725200	3.90344200
H	-4.02646200	-3.76412000	3.51613000
C	2.22740500	-2.26861600	-1.32398500
C	2.55838200	-1.74221700	-2.58108300
H	2.13989800	-0.79426900	-2.89898900
C	3.44267500	-2.42007400	-3.41527500
C	3.71961000	-4.13302300	-1.74656200
C	4.02695700	-3.61573800	-3.00164000
C	-4.29053200	1.80425700	-3.52895800
C	-4.70046600	1.51871600	-2.22756100
C	1.97993000	-2.92663400	3.45272800
C	0.36329100	-4.40809000	2.45858200
Si	3.69829800	0.86111400	-0.06924800
C	4.75772200	0.76689000	-1.62169100
H	4.29547200	1.29226900	-2.45722800
H	5.72589600	1.22971400	-1.40359000
H	4.93726800	-0.26922600	-1.91785600
C	4.64707900	-0.07888000	1.27175600
H	4.62989300	-1.15748600	1.09137500
H	5.69412800	0.24123100	1.24725900
H	4.26036600	0.11701000	2.27303200
H	-3.43940200	5.89217700	1.11901700
H	-1.31944200	7.12376700	1.51166100
H	0.85463400	5.95582800	1.21185100
H	-2.60775600	2.20547900	-4.80934200
H	-5.02218700	1.88347500	-4.32556000
H	-5.75375600	1.38223800	-2.00535200
H	-0.22761800	-5.31691500	2.48796900
H	1.30093300	-4.75417800	4.36625100
H	2.64890900	-2.66948400	4.26602800
H	4.17645700	-5.05690200	-1.40989800
H	4.72376800	-4.13557800	-3.64967400
H	3.68547500	-1.99922300	-4.38433100
Ir	1.30909500	0.84907400	-0.22086800
Cl	1.48758700	1.58838700	-2.59856800
O	3.82622200	2.52919800	0.33913100
C	1.24771300	0.94318700	1.81426200
H	0.52624100	0.29086400	2.30089400
C	3.65250400	3.17498900	1.50844000
C	2.83634700	2.85189600	2.53055200
H	2.91216100	3.52655900	3.37869200
C	1.85759300	1.78504800	2.68640300
H	1.49419600	1.70819600	3.71243500
H	4.25620600	4.07751300	1.56715800

product

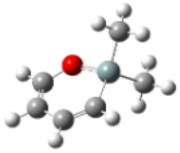

C	1.57824800	-1.25740900	-0.00003300
C	2.32040300	-0.13282100	0.00004500
C	1.74496100	1.20142500	-0.00010400
C	0.41862000	1.47386700	-0.00020500
H	2.02407100	-2.24702600	-0.00002800
H	3.39772300	-0.24045600	0.00015800
H	2.45857100	2.02371100	-0.00019400
H	0.11050300	2.51637800	-0.00037000
O	0.23189500	-1.29380700	-0.00025400
Si	-0.79744000	0.07406700	-0.00001100
C	-1.87157000	-0.01956200	1.53518800
H	-2.55184300	0.83720200	1.58460400
H	-2.48140200	-0.92799200	1.53665000

H	-1.26178400	-0.01621000	2.44207400
C	-1.87223000	-0.01963200	-1.53473400
H	-2.48198000	-0.92811700	-1.53589300
H	-2.55260400	0.83706600	-1.58386400
H	-1.26285100	-0.01625800	-2.44189200

Iridium / (S)-DTBM-SEGPHOS Monomer

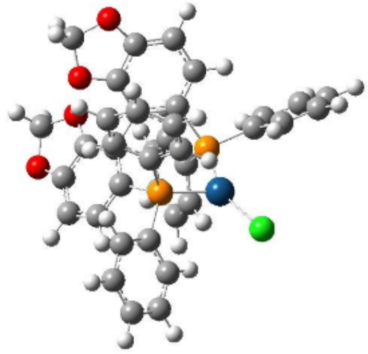

P	-0.82829200	1.50936300	-0.05584600
P	1.52669900	-0.66624400	-0.07602000
O	-2.76385600	-3.51163200	-3.10745200
O	-3.21483000	-2.53219200	3.62564000
O	-2.25857600	-3.29428600	1.66713800
O	-3.36352900	-2.51352600	-1.11432100
C	-1.35315200	3.18216000	0.54715000
C	-2.44518100	3.88271000	0.02098300
H	-3.04435100	3.44560700	-0.76732500
C	-2.76227800	5.15307600	0.49654700
C	-0.89922800	5.05665300	2.02221100
C	-0.57950900	3.78625600	1.54997800
H	0.27846500	3.25713200	1.95260400
C	0.56040200	-2.21603500	-2.20190900
H	1.58262100	-2.16567900	-2.54903800
C	-1.72525700	1.33478100	-1.66357800
C	-1.68248400	0.30930300	1.06140000
C	1.61320800	-1.46472500	1.58892100
C	-1.02705600	1.62280400	-2.84083000
H	0.02417500	1.88222600	-2.77419800
C	-1.67078600	1.56086700	-4.07452700
C	-3.07549400	0.97260600	-1.74057800
H	-3.62481200	0.72778000	-0.83868000
C	-1.99236700	5.74209000	1.49733000
C	-0.37215800	-2.89549700	-2.99868000
H	-0.09244200	-3.34889000	-3.94036500
C	-1.12230800	-1.66876900	-0.51302900
C	-2.01403300	-2.35512200	-1.31840700
C	-1.66036400	-2.94959800	-2.52434200
C	3.73362300	-2.40790700	-0.55006200
H	3.31614200	-3.04760500	0.21722700
C	2.19670300	-0.74173500	2.63680800
H	2.52260600	0.27683800	2.45957400
C	1.90947900	-2.62856300	4.11755100
C	1.17711700	-2.77431300	1.82043400
H	0.71092900	-3.34396600	1.02560900
C	-3.79817700	-3.43378100	-2.11924000
H	-3.94564700	-4.42349300	-1.66957400
H	-4.71303600	-3.06210200	-2.58046400
C	0.22305800	-1.61436300	-0.98630100
C	-2.26614200	0.75152600	2.24955600
H	-2.29444100	1.81142100	2.46214200
C	-2.82643500	-0.12839400	3.18850200
H	-3.27125700	0.22823200	4.10814200
C	-2.77557400	-1.46714700	2.88333500
C	-2.20385800	-1.92185700	1.70029600
C	-1.63961700	-1.08240600	0.75698600
C	-3.09496500	-3.67066400	2.76582000
H	-2.62885000	-4.49055200	3.31211900
H	-4.08733500	-3.94876100	2.38961100
C	3.10546700	-1.20177800	-0.88459500
C	3.68122400	-0.38030900	-1.86020900
H	3.21786700	0.57029900	-2.09434600
C	4.85638200	-0.76606300	-2.50037400
C	4.90959900	-2.78896000	-1.19095500
C	5.47171300	-1.97066900	-2.16887800
C	-3.01620300	1.20535600	-4.14405800
C	-3.71670600	0.90896400	-2.97578300
C	2.34568600	-1.32399500	3.89236900
C	1.32388200	-3.35120900	3.08078400
H	-3.60888200	5.68564100	0.07761200
H	-2.23874600	6.73362100	1.86047300
H	-0.28749500	5.51109200	2.79315700
H	-1.11970200	1.78501200	-4.98100300
H	-3.51629000	1.15423200	-5.10492300
H	-4.76277400	0.62679200	-3.02496300
H	0.97994300	-4.36582300	3.24932400
H	2.02294800	-3.07862100	5.09763500
H	2.80008400	-0.75527100	4.69580300
H	5.39075900	-3.72171800	-0.91841700
H	6.39138100	-2.26602400	-2.66172900
H	5.29684800	-0.11529700	-3.24708700
Ir	1.39580300	1.52141600	-0.01224900
Cl	3.48384800	2.58019900	0.24002500

TS-1

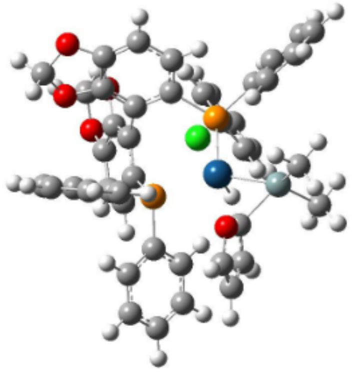

P	-0.20782300	-1.67723300	0.21645300
P	0.30257600	1.84215600	0.08554700
O	4.20496400	-0.32956700	-3.86391700
O	5.18421100	-1.04177700	2.74957200
O	4.75040700	0.14259800	0.82027100
O	4.00761600	-1.44401200	-1.85569500
C	-1.09252800	-2.86936000	1.33675200
C	-1.38710100	-4.18201900	0.95538600
H	-1.07930900	-4.54605500	-0.01592800
C	-2.07862400	-5.03555300	1.81626500
C	-2.18134600	-3.28985600	3.46846800
C	-1.50970200	-2.43250300	2.60323400
H	-1.30232200	-1.41463300	2.91061700
C	1.80451100	1.96672400	-2.27483200
H	1.24426200	2.87211600	-2.45444100
C	0.15818800	-2.73355700	-1.25620000
C	1.45546300	-1.52780000	1.03426200
C	1.13844800	2.11495500	1.71361500
C	-0.75967100	-2.74735700	-2.31352000
H	-1.61472500	-2.08488300	-2.29176600
C	-0.54987000	-3.57700600	-3.41336700
C	1.29325900	-3.55160500	-1.32593500
H	2.01822400	-3.54573100	-0.52172800
C	-2.47333300	-4.59624800	3.07553300
C	2.64720300	1.50756300	-3.29662200
H	2.75258400	2.04504800	-4.22949300
C	2.36422900	0.08409600	-0.82020700
C	3.17780600	-0.34578400	-1.85234100
C	3.31054000	0.32944300	-3.06097300
C	0.52821800	4.69538300	0.18567100
H	1.22339600	4.55564100	1.00239900
C	0.38269100	2.10791400	2.89050400
H	-0.67177800	1.86785700	2.84139800
C	2.33518000	2.70090200	4.18227600
C	2.50214700	2.42375100	1.78822000
H	3.10700800	2.43134200	0.88973000
C	4.42712800	-1.58733600	-3.21594700
H	5.48999600	-1.82562500	-3.24383200
H	3.82122800	-2.36064900	-3.70379200
C	1.64326800	1.29212300	-1.06353700
C	1.74357300	-2.24170700	2.20190600
H	0.99712400	-2.90417200	2.61405100
C	2.97523200	-2.15617400	2.86799000
H	3.17150100	-2.71574200	3.77320000
C	3.91906600	-1.33108100	2.30738500
C	3.65367900	-0.62435300	1.14140700
C	2.44592100	-0.67388900	0.46720400
C	5.79869100	-0.33097400	1.66764600
H	6.35930800	0.51577000	2.06176200
H	6.44351700	-1.01601100	1.10243200
C	-0.06421200	3.59167400	-0.43937500
C	-0.96959300	3.81389800	-1.48685000
H	-1.42512100	2.96625300	-1.98594400
C	-1.26309500	5.11051000	-1.90132000
C	0.22083000	5.99223800	-0.22510500
C	-0.67305600	6.20434400	-1.27002100
C	0.57753000	-4.39243700	-3.47472000
C	1.50056200	-4.37579400	-2.42975700
C	0.97645100	2.40104400	4.11587200
C	3.09613300	2.70997500	3.01552000
Si	-3.45542600	1.59422500	0.10176800
C	-4.39528700	2.35427100	-1.34236900
H	-4.04257500	1.93812200	-2.28663200
H	-5.47123900	2.17614900	-1.24685200
H	-4.24021300	3.43804800	-1.35761900
C	-4.21662100	2.26238000	1.70098500
H	-3.92613000	3.31215100	1.82089300
H	-5.31015800	2.22549700	1.68297800
H	-3.85897200	1.72102500	2.57916700
H	-2.30092800	-6.04827200	1.49823200
H	-3.00298900	-5.26313200	3.74682600
H	-2.48232500	-2.93625900	4.44885100
H	-1.26309700	-3.56977800	-4.22983600
H	0.73996100	-5.03403700	-4.33412800
H	2.38110700	-5.00821100	-2.46929100
H	4.15475500	2.94129200	3.05721700
H	2.79848400	2.92373100	5.13700900
H	0.37666900	2.39155300	5.01929000
H	0.68266600	6.83483500	0.27777700

H	-0.91005200	7.21328000	-1.58946100
H	-1.95773900	5.26246800	-2.72016000
Ir	-1.48633300	0.41110100	0.01972600
H	-1.54677600	0.39457500	1.59698500
Cl	-1.47529600	0.51306100	-2.54571600
C	-3.63740500	-0.40411000	0.00852500
C	-4.21338400	-1.15365700	1.02485600
C	-5.03265400	-2.14934400	0.44850000
H	-4.04741000	-0.98390400	2.07603500
C	-4.92488400	-1.96192500	-0.90382000
H	-5.61386600	-2.90608500	0.95028100
H	-5.36609000	-2.47521500	-1.74444900
O	-4.11808700	-0.94082500	-1.19588800

TS-2

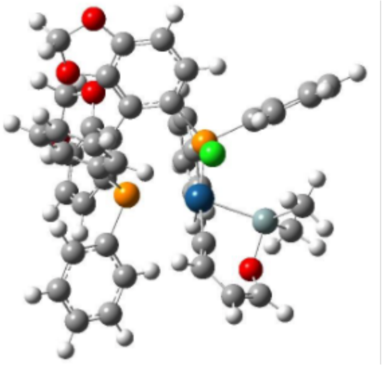

P	-0.37188400	1.77373600	0.13036500
P	0.25838200	-1.73838500	-0.03837400
O	-4.53004300	-0.90239300	-3.51278000
O	-4.83186900	-0.67499100	3.27519300
O	-4.34299100	-1.58595100	1.21315400
O	-4.51490000	0.13818900	-1.45290100
C	0.08584100	3.30463600	1.08230600
C	-0.43090100	4.56658000	0.76339000
H	-1.11610700	4.68056400	-0.06577800
C	-0.06562100	5.69167900	1.50124900
C	1.33733400	4.32381600	2.89716500
C	0.98504800	3.20145800	2.15318000
H	1.42040000	2.24099300	2.39387300
C	-1.29341400	-2.20862300	-2.33836400
H	-0.47715500	-2.85093500	-2.63283500
C	-1.25020400	2.48789800	-1.33199400
C	-1.75675800	1.05656900	1.13370800
C	-0.40353800	-2.33872600	1.58286500
C	-0.52172300	2.73154700	-2.50213800
H	0.50665400	2.40212800	-2.57570000
C	-1.13178100	3.35027500	-3.59192000
C	-2.59916400	2.86405900	-1.27579400
H	-3.18033600	2.67461300	-0.38200700
C	0.81432200	5.57395100	2.57314500
C	-2.35279100	-2.04397300	-3.24230000
H	-2.36200600	-2.54337200	-4.20191600
C	-2.32373600	-0.71702300	-0.69784600
C	-3.35428800	-0.58179900	-1.61071300
C	-3.37145400	-1.21186700	-2.85002000
C	1.07399700	-4.48662300	-0.00494900
H	0.51686100	-4.58616400	0.91605600
C	0.24572000	-2.00238700	2.77414900
H	1.09448600	-1.33185200	2.74562000
C	-1.29615200	-3.36874000	4.03431200
C	-1.51003500	-3.19724600	1.63462600
H	-2.03446200	-3.46026100	0.72395500
C	-5.13789800	0.14041600	-2.74177800
H	-6.20214900	-0.06643700	-2.62901100
H	-4.95845600	1.10534700	-3.23022500
C	-1.25435300	-1.57600800	-1.09543300
C	-2.06727700	1.59263500	2.38614900
H	-1.51030900	2.44453000	2.74892400
C	-3.09139100	1.08352800	3.19899600
H	-3.31278500	1.51047900	4.16833100
C	-3.80287600	0.02141600	2.69692700
C	-3.50786000	-0.52113300	1.45180600
C	-2.48977800	-0.06065400	0.63588300
C	-5.33711600	-1.52487900	2.23836800
H	-5.50789000	-2.52345700	2.63945300
H	-6.25764800	-1.09092700	1.82797900
C	1.12187600	-3.25903300	-0.67835100
C	1.87652200	-3.16734100	-1.85801900
H	1.94510300	-2.21879500	-2.37981300
C	2.54030700	-4.28478200	-2.35927300
C	1.75286200	-5.59728600	-0.50449000
C	2.48232000	-5.50302500	-1.68580600
C	-2.46975300	3.73041100	-3.52765400
C	-3.20322600	3.48339300	-2.36748500
C	-0.19747600	-2.51477500	3.99212700
C	-1.95256300	-3.70704700	2.85192900
Si	3.76734300	-0.71875300	0.22623800
C	4.85006000	-1.13148800	-1.25512400
H	4.66025100	-0.43899900	-2.07684300
H	5.91061200	-1.09230200	-0.98327000
H	4.63496800	-2.14166600	-1.61223900
C	4.14480000	-1.94692200	1.60775700
H	3.70868800	-2.92373600	1.37748200

H	5.22494500	-2.08284700	1.72596900
H	3.73428700	-1.61098600	2.56266900
H	-0.47252700	6.66060000	1.23354700
H	1.09720000	6.44978600	3.14655800
H	2.03432100	4.22109000	3.72138400
H	-0.55800700	3.52286800	-4.49539500
H	-2.94091700	4.21326900	-4.37696900
H	-4.24515100	3.77958300	-2.30689800
H	-2.81048600	-4.36993300	2.87509300
H	-1.64243500	-3.76517300	4.98229900
H	0.31537000	-2.24148500	4.90747900
H	1.70774000	-6.53650300	0.03575400
H	3.00600600	-6.36912400	-2.07516000
H	3.11038900	-4.19477400	-3.27738900
Ir	1.61076700	0.26486200	-0.04248000
H	1.60309700	0.28387000	1.53319400
Cl	1.72537700	0.19218900	-2.62412700
C	2.92656600	1.69981800	-0.07166500
C	3.66177300	2.75138000	-0.42009000
C	5.07740700	2.78639800	-0.10045700
H	3.16771600	3.53659000	-0.98206700
C	5.47528000	1.68489900	0.55879300
H	5.72778200	3.60579500	-0.36901400
H	6.46565100	1.44321400	0.92412800
O	4.46006300	0.83549600	0.84904200

TS-3

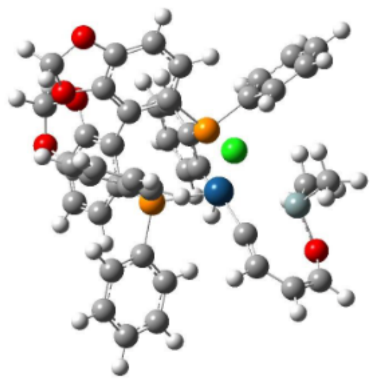

P	0.26010200	-1.73893700	0.10468300
P	-0.09465400	1.72152600	0.00007200
O	4.70010400	0.82039400	-3.45351900
O	4.89727100	0.25932700	3.30086900
O	4.51246200	1.24980400	1.25360700
O	4.59997700	-0.31554500	-1.44545400
C	-0.26641200	-3.28282700	1.00462300
C	0.34103100	-4.51580400	0.72959500
H	1.09924800	-4.59095400	-0.03826300
C	-0.02731600	-5.66116300	1.43155400
C	-1.61508800	-4.37484600	2.70139900
C	-1.25445000	-3.22934700	1.99533200
H	-1.75412900	-2.29362800	2.20167600
C	1.50301300	2.21599500	-2.27173700
H	0.72228600	2.90829100	-2.54865700
C	1.00131100	-2.45496600	-1.42539100
C	1.70649900	-1.15222300	1.09894700
C	0.58837500	2.24375900	1.64076200
C	0.15286600	-2.78943300	-2.48719200
H	-0.89214600	-2.51467600	-2.44997500
C	0.65911500	-3.43868900	-3.61009800
C	2.36060400	-2.78153600	-1.50622600
H	3.03343200	-2.53318300	-0.69577800
C	-1.00369100	-5.59429600	2.42225500
C	2.56921300	2.04251500	-3.16598400
H	2.61673900	2.58149500	-4.10280100
C	2.43913100	0.61190500	-0.68408400
C	3.47895400	0.46905800	-1.58559200
C	3.54497100	1.15144000	-2.79435300
C	-0.75695700	4.50842800	0.10875100
H	-0.19903700	4.55068600	1.03363300
C	-0.09526500	1.91064200	2.81379900
H	-0.98218400	1.29280900	2.75552600
C	1.50718200	3.14392700	4.13379700
C	1.74307900	3.03266900	1.73235700
H	2.29302700	3.29323200	0.83606700
C	5.25902800	-0.27181100	-2.71685200
H	6.32487200	-0.09926300	-2.56536100
H	5.07178400	-1.20768200	-3.25612400
C	1.41585200	1.53382600	-1.05781500
C	1.95066000	-1.73079300	2.34807600
H	1.31098400	-2.52904900	2.69521400
C	3.00765300	-1.33134000	3.17833300
H	3.17512000	-1.78841300	4.14458400
C	3.82011300	-0.33280000	2.69916300
C	3.58927000	0.25430600	1.46050000
C	2.54365600	-0.09871100	0.62563700
C	5.48363700	1.08941100	2.28977000
H	5.72549600	2.06266300	2.71558400
H	6.37332100	0.59282900	1.88306800
C	-0.86823400	3.30568800	-0.60112500
C	-1.62202200	3.29173800	-1.78452100
H	-1.73266300	2.36579700	-2.33747400
C	-2.23015200	4.45528900	-2.24926500
C	-1.37633000	5.66752800	-0.35637800

C	-2.11150700	5.64711200	-1.53773600
C	2.01157400	-3.76214600	-3.68665100
C	2.86111000	-3.43160000	-2.63278800
C	0.36025800	2.35906500	4.05239100
C	2.19875600	3.47767400	2.97014900
Si	-4.08713500	0.74755300	0.13571200
C	-4.82382300	1.15569300	-1.53543400
H	-5.69917700	1.79977400	-1.39984400
H	-4.10350400	1.65769000	-2.18047000
H	-5.13439700	0.24188100	-2.04648300
C	-3.83275800	2.25656900	1.22249200
H	-3.15296900	2.98081100	0.77588300
H	-4.80965900	2.73107000	1.36503200
H	-3.44906300	1.97109300	2.20347800
H	0.44984600	-6.60655500	1.19851600
H	-1.29133400	-6.48698300	2.96648600
H	-2.38564700	-4.31155900	3.46144600
H	-0.00760600	-3.68076100	-4.42966400
H	2.40222300	-4.26762800	-4.56304100
H	3.91407900	-3.68819000	-2.67894500
H	3.09364400	4.08787800	3.02415900
H	1.86302000	3.49087400	5.09750300
H	-0.18101400	2.09032500	4.95258400
H	-1.28246000	6.58619000	0.21219100
H	-2.59209200	6.54994700	-1.89803500
H	-2.80440400	4.42298400	-3.16856300
Ir	-1.54589800	-0.27043500	-0.06005700
H	-1.53009200	-0.32680500	1.51393500
Cl	-1.65527900	-0.04521400	-2.62215400
C	-3.09582300	-1.33535400	-0.07980800
C	-4.01413300	-2.29235400	-0.10522400
C	-5.42987000	-2.23028800	0.22026800
H	-3.61010000	-3.26715700	-0.38222500
C	-5.99617400	-1.15066500	0.77744500
H	-6.03483500	-3.11269100	0.05718900
H	-7.03425100	-1.12907700	1.09328500
O	-5.32927700	-0.00447300	1.04268700

TS-3’

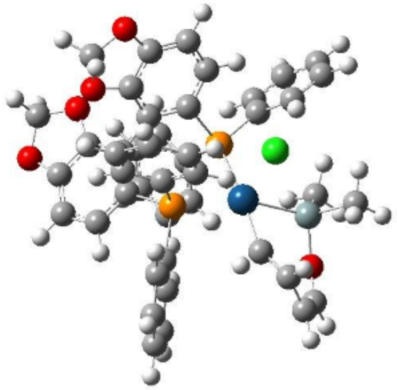

P	-0.58962900	1.77096000	0.02488500
P	0.41411400	-1.71103900	0.01284900
O	-4.58236300	-1.39898300	-3.28772800
O	-4.76663000	-0.81787900	3.45300600
O	-4.15829300	-1.87843400	1.48894900
O	-4.56632100	-0.24870900	-1.28354700
C	-0.16364300	3.35212200	0.89688700
C	-0.41618900	4.60989400	0.33211700
H	-0.91590400	4.68444400	-0.62709400
C	-0.02553600	5.77867400	0.99145400
C	0.86966100	4.46415100	2.80369500
C	0.49252500	3.29781000	2.14041700
H	0.70063200	2.33656700	2.60324600
C	-1.23533400	-2.44823200	-2.14442000
H	-0.39262200	-3.06089900	-2.43357900
C	-1.53180600	2.36787300	-1.44702500
C	-1.90609400	1.03893600	1.10956500
C	-0.04902300	-2.10636500	1.75649700
C	-0.84402000	2.57410800	-2.65294000
H	0.20686800	2.31764100	-2.72537100
C	-1.51482600	3.08484200	-3.76670000
C	-2.89974000	2.67850600	-1.37657700
H	-3.44561000	2.52344500	-0.45167300
C	0.61457800	5.71129400	2.22803700
C	-2.33071900	-2.40347200	-3.02309300
H	-2.33644200	-2.96467800	-3.95081000
C	-2.30401000	-0.93222600	-0.55304600
C	-3.36844300	-0.90940800	-1.44244200
C	-3.38760300	-1.60709100	-2.64672000
C	1.21594300	-4.46272900	0.20941600
H	0.70150000	-4.49271300	1.16257200
C	0.70952600	-1.54231700	2.79198200
H	1.51666100	-0.85564300	2.54793000
C	-0.60122400	-2.72946100	4.44119300

C	-1.09391900	-2.98523500	2.08428000
H	-1.70394900	-3.41849400	1.29729200
C	-5.23010800	-0.36054500	-2.54527500
H	-6.27680300	-0.62977100	-2.38259300
H	-5.13993500	0.58961100	-3.08982800
C	-1.18745500	-1.74045300	-0.93594900
C	-2.30227100	1.67188100	2.29623700
H	-1.86547300	2.62565700	2.56111700
C	-3.26480600	1.13264000	3.16733100
H	-3.55422300	1.64188100	4.07994800
C	-3.82501300	-0.06889600	2.79569900
C	-3.45599800	-0.70189700	1.61268800
C	-2.50275300	-0.20402000	0.73810900
C	-5.11395400	-1.87520700	2.55155600
H	-5.07421300	-2.83131400	3.08079200
H	-6.11774400	-1.69282900	2.14323300
C	1.24238300	-3.28173100	-0.54684300
C	1.94367500	-3.28298100	-1.76662400
H	1.99742800	-2.37354900	-2.35639200
C	2.57867000	-4.43923600	-2.22187800
C	1.86399100	-5.61470600	-0.24306300
C	2.54353200	-5.60965800	-1.46086200
C	-2.87415500	3.39157300	-3.69051200
C	-3.56532800	3.18735700	-2.49288400
C	0.43747400	-1.85219100	4.12628600
C	-1.36722900	-3.29340200	3.41756500
Si	3.91405800	-0.41212100	-0.09006800
C	4.93662400	-0.28455700	-1.67460400
H	5.99063600	-0.50404400	-1.46192300
H	4.57542700	-1.00524900	-2.41626000
H	4.86949200	0.71027200	-2.12342500
C	4.19318600	-2.13431200	0.66123000
H	5.24532400	-2.19524400	0.96555800
H	3.57884600	-2.31023800	1.55088800
H	3.99264500	-2.93753800	-0.05144900
H	-0.22695000	6.74317600	0.53280900
H	0.91632800	6.62128000	2.73955600
H	1.37117600	4.39732600	3.76540900
H	-0.97158800	3.23480900	-4.69568900
H	-3.39381100	3.78874500	-4.55875200
H	-4.62205600	3.43384600	-2.42270600
H	-2.17871200	-3.97590900	3.65718200
H	-0.81591100	-2.97083900	5.47882400
H	1.03656600	-1.40771900	4.91643300
H	1.83660800	-6.51615400	0.36364000
H	3.04623800	-6.50712700	-1.81160100
H	3.11103600	-4.41828000	-3.16918500
Ir	1.59534500	0.40159300	-0.27311600
H	2.46061700	1.83971100	1.08964700
Cl	1.59318400	0.08442100	-2.65113500
C	2.68639600	1.99272600	-0.03616300
C	3.75601300	2.75888700	-0.30247700
C	4.82435700	2.83750000	0.69869500
H	3.91883300	3.13855500	-1.31318500
C	5.24201000	1.70846000	1.31544800
H	5.35598500	3.76302500	0.90860100
H	6.05491600	1.74868100	2.04119100
O	4.78890100	0.44764800	1.15082900

TS-4

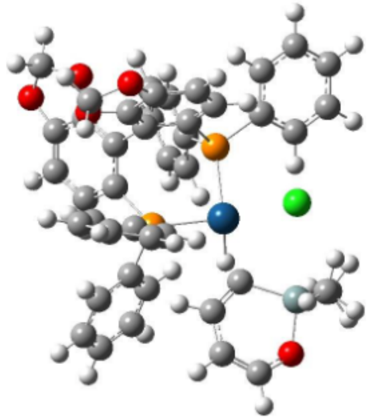

P	0.25487900	-1.40091900	-0.43951400
P	-0.69744900	1.78359200	-0.04024300
O	-3.52648500	-0.98224200	4.40608200
O	-5.48907900	-1.51842300	-2.17594600
O	-5.00783000	-0.50629500	-0.16105800
O	-3.69625800	-1.92064700	2.30268400
C	1.05320500	-2.47833700	-1.72808900
C	1.12799400	-3.87205700	-1.62579900
H	0.73369600	-4.37733200	-0.75384500
C	1.72830200	-4.62430700	-2.63364400
C	2.18494100	-2.61018900	-3.87107100
C	1.59478000	-1.85690700	-2.86005000
H	1.56339800	-0.77736700	-2.94118000
C	-1.62299500	1.59272100	2.58299000
H	-1.05800300	2.50391000	2.72705200
C	0.11111900	-2.48559500	1.06432300
C	-1.52225600	-1.40640200	-1.02185500
C	-1.65228100	1.77843100	-1.62839400
C	0.93104400	-2.24173200	2.17279000
H	1.62981500	-1.41518900	2.14445500

C	0.84782600	-3.05039100	3.30475600
C	-0.80726600	-3.54375200	1.12169400
H	-1.47628000	-3.72916700	0.29023300
C	2.25464100	-3.99711600	-3.76001700
C	-2.19312100	0.98242000	3.70883900
H	-2.09944200	1.41416100	4.69649200
C	-2.40252400	-0.19154200	1.10168000
C	-2.96694700	-0.76024700	2.23006400
C	-2.86551300	-0.19832600	3.49731300
C	-1.82920000	4.26290600	0.82507400
H	-2.63903900	3.68805500	1.25599600
C	-0.93625100	1.63868700	-2.81946700
H	0.12286200	1.41106200	-2.77247900
C	-2.93292900	2.09241500	-4.09749900
C	-3.01714700	2.08223400	-1.68318300
H	-3.58793900	2.19860600	-0.76915600
C	-3.85320500	-2.18622200	3.70150400
H	-4.89014400	-2.45413200	3.90450900
H	-3.16014500	-2.98176600	3.99904800
C	-1.73447800	1.05036000	1.29783900
C	-1.83736500	-1.99349300	-2.25068300
H	-1.04688500	-2.40255300	-2.86164900
C	-3.14930200	-2.09621300	-2.73737500
H	-3.36355000	-2.54518200	-3.69816400
C	-4.14368800	-1.59246100	-1.93555900
C	-3.84986600	-0.99897800	-0.71382300
C	-2.56836000	-0.86762000	-0.21412100
C	-6.06414400	-1.07118300	-0.94048500
H	-6.81390300	-0.30803100	-1.14482300
H	-6.49302000	-1.92959500	-0.40849900
C	-0.73761500	3.61707300	0.23178300
C	0.27988000	4.39148200	-0.33635000
H	1.13195100	3.90770600	-0.79317600
C	0.21267700	5.78138300	-0.29773200
C	-1.88795600	5.65389300	0.87076700
C	-0.86660200	6.41680700	0.31064800
C	-0.05256000	-4.11186900	3.34481700
C	-0.88220500	-4.35535600	2.25077000
C	-1.57365500	1.79773200	-4.04893600
C	-3.65375100	2.23051700	-2.91142500
Si	4.84673600	0.81870900	0.41652500
C	5.10860400	2.30254400	-0.70059200
H	6.17438700	2.37714700	-0.93725700
H	4.56098600	2.19415700	-1.64027000
H	4.78354800	3.22795400	-0.22540800
C	5.31992600	1.12084100	2.21120100
H	5.29945000	0.17855700	2.76748900
H	6.34178000	1.51190100	2.25811200
H	4.64737800	1.82398600	2.70049900
H	1.78784600	-5.70233300	-2.53317800
H	2.72423200	-4.58377700	-4.54147900
H	2.60164200	-2.11111800	-4.73861000
H	1.48915500	-2.84627700	4.15457900
H	-0.11080400	-4.74437700	4.22392800
H	-1.59172300	-5.17530400	2.27530900
H	-4.71309500	2.46029700	-2.94334200
H	-3.43024500	2.21631400	-5.05298100
H	-1.00611400	1.69104800	-4.96674200
H	-2.73591600	6.13864000	1.34196500
H	-0.91273400	7.49956300	0.34679600
H	1.01380800	6.36611400	-0.73532700
Ir	1.34825300	0.54621500	0.05213800
C	3.33832800	-0.28716000	0.13816700
C	3.63092500	-1.62540200	0.10541600
C	4.93746600	-2.23995700	0.19888800
H	2.83443900	-2.34753000	-0.03339800
C	6.08729500	-1.54705300	0.03537600
H	4.99811500	-3.31839800	0.28251900
H	7.05366100	-2.04069800	-0.03586000
O	6.14404800	-0.22403900	-0.11246700
Cl	2.34619900	2.45668400	1.20809200
H	2.45039000	0.15441500	-1.04696200

TS-4'

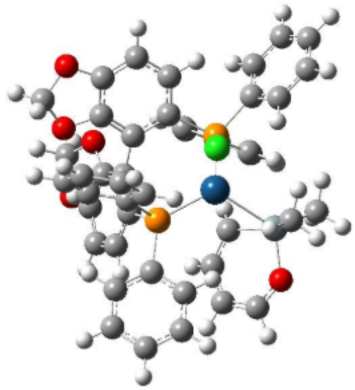

P	-1.98760800	0.30707100	-0.22597100
P	2.06044900	-0.05159700	-0.29568500
O	-0.19067500	-5.40628700	-1.78834700
O	-0.57675200	-3.12145100	4.48577000
O	0.53456400	-3.53641000	2.51070600
O	-1.24393000	-4.29184800	-0.06507600
C	-3.52101600	1.26344700	0.22669400

C	-4.71507100	0.65017900	0.63583600
H	-4.75328200	-0.42236900	0.78324300
C	-5.86610800	1.40433400	0.83707500
C	-4.68055600	3.39537000	0.17971400
C	-3.52261400	2.64010000	-0.00697200
H	-2.61467500	3.11882300	-0.35155400
C	2.00113600	-2.45679900	-1.70711800
H	2.86316300	-2.02528200	-2.19323100
C	-2.78339000	-0.80821600	-1.47476700
C	-1.60821900	-0.80669700	1.21855500
C	2.90027400	-0.20837600	1.35261200
C	-2.97243900	-0.32022300	-2.77360800
H	-2.54820700	0.63367300	-3.05670900
C	-3.68597800	-1.06480500	-3.70838100
C	-3.31859000	-2.05412900	-1.13207700
H	-3.17615200	-2.45486400	-0.13670600
C	-5.85179900	2.78098800	0.60921900
C	1.54714900	-3.69528200	-2.17920800
H	2.04806900	-4.21535700	-2.98482900
C	0.20087400	-2.28732200	-0.05438000
C	-0.20221200	-3.52173700	-0.53716300
C	0.43210900	-4.20705600	-1.56739800
C	4.76568700	-0.56304800	-1.07711200
H	4.80839200	-1.25370700	-0.24327200
C	3.59645000	0.89945300	1.85806100
H	3.61954900	1.82507500	1.29662800
C	4.28247900	-0.36991700	3.79324500
C	2.91756000	-1.39983200	2.08222600
H	2.41114300	-2.27818200	1.70842000
C	-1.38891000	-5.35960400	-1.00650800
H	-1.50787500	-6.30082700	-0.46929300
H	-2.24158400	-5.15427700	-1.66359600
C	1.38445900	-1.75094900	-0.67062900
C	-2.26537500	-0.57675000	2.42997600
H	-3.02454300	0.19069300	2.47689800
C	-1.99282100	-1.29330600	3.60628500
H	-2.51818400	-1.09338200	4.53104900
C	-1.03529900	-2.27073300	3.51339500
C	-0.36532300	-2.51015300	2.31736700
C	-0.57206000	-1.79810200	1.14740500
C	0.21031100	-4.09147200	3.78722200
H	1.12621300	-4.28470600	4.34452900
H	-0.37846700	-5.00679000	3.64651900
C	3.57784500	0.12741600	-1.35959500
C	3.55629500	1.02714200	-2.42976900
H	2.63806400	1.55057200	-2.66065600
C	4.69622800	1.22630500	-3.20746700
C	5.90195000	-0.36321900	-1.85576800
C	5.86958900	0.53315600	-2.92335600
C	-4.22143900	-2.30304800	-3.36054600
C	-4.03547200	-2.79539000	-2.07102800
C	4.28015500	0.82033600	3.06677400
C	3.60325000	-1.47711500	3.29529800
Si	0.78233000	3.53861000	0.12388800
C	0.38510300	4.02622200	-1.68115200
H	-0.60021100	3.71892200	-2.02972600
H	0.43009800	5.12255700	-1.70344500
H	1.12348700	3.62378700	-2.37381400
C	2.51119200	4.22772200	0.44269100
H	3.25807800	3.74755000	-0.19578900
H	2.51429700	5.29596700	0.20194600
H	2.81817900	4.11916100	1.48545000
H	-6.77986500	0.91546300	1.15673900
H	-6.75349900	3.36554100	0.75456900
H	-4.66339400	4.46192200	-0.01529000
H	-3.81800700	-0.67691800	-4.71213100
H	-4.78020000	-2.87978800	-4.08943400
H	-4.46064900	-3.75260500	-1.78754400
H	3.61166700	-2.41244600	3.84433800
H	4.81597600	-0.43298900	4.73509800
H	4.81449600	1.68743700	3.43884500
H	6.81376700	-0.90306400	-1.62476600
H	6.75636200	0.69133200	-3.52731900
H	4.66243500	1.92435600	-4.03649600
Ir	0.16877900	1.18180800	-0.56422600
Cl	0.19126700	0.58978100	-2.91485500
O	-0.32010400	4.78839500	0.80470200
C	0.38832400	2.18554300	1.47280200
H	1.17593500	1.55802700	1.86979900
C	-1.22326500	4.66835300	1.73662400
C	-1.45723300	3.54792500	2.48990100
H	-2.20727400	3.60903800	3.26769300
C	-0.56827400	2.44957300	2.44298500
H	-0.55814500	1.82047700	3.33416400
H	-1.77249800	5.58815300	1.93821000

8. X-ray Crystallographic Analyses

Single crystal of **2aJ**, **2mN** and **2aQ** obtained from hexane or $CHCl_3$ solution was mounted in the loop. The X-ray structural determination was performed on a Rigaku Saturn724+ CCD diffractometer using graphitemonochromated Mo Kα radiation (λ = 0.71070 Å) at 143 K and processed using CrystalClear (Rigaku). The structures were solved using a direct method (SIR2002 or SHELX97) and refined by a full-matrix leastsquares method on F^2 for all reflections using the programs of SHELXL-2018. All non-hydrogen atoms were refined anisotropic displacement parameters. All hydrogen atoms were located at the calculated positions. Crystallographic data for the structure of **2aJ**, **2mN** and **2aQ** have been deposited with the Cambridge Crystallographic Data Centre as supplementary publication No. CCDC-2497867, CCDC-2497868 and CCDC-2497869. This data can be obtained free from the Cambridge Crystallographic Data Centre via www.ccdc.cam.ac.uk/data_request/cif.

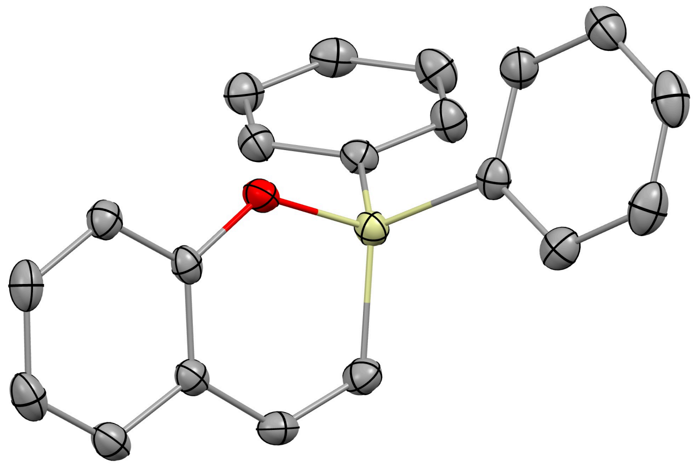

Figure S23. Cristal Structure of **2aJ** (ORTEP 50% probability) H atom was omitted for the Clarity.

Empirical formula	$C_{20}H_{16}SiO$	
Formula weight	300.42	
Temperature	143(2) K	
Wavelength	0.71075 Å	
Crystal system	Monoclinic	
Space group	$P2_1/c$	
Unit cell dimensions	a = 10.5752(17) Å	α = 90°
	b = 14.526(2) Å	β = 104.290(2)°
	c = 10.5590(17) Å	γ = 90°
Volume	1571.8(4) $Å^3$	
Z	4	
Density (calculated)	1.270 Mg/m^3	
Absorption coefficient	0.148 mm^{-1}	
F(000)	632	
Crystal size	0.210 x 0.080 x 0.050 mm^3	
Theta range for data collection	3.438 to 27.515°	
Index ranges	-13<=h<=13, -18<=k<=16, -13<=l<=10	
Reflections collected	12466	
Independent reflections	3595 [*R*(int) = 0.0275]	
Completeness to theta = 25.242°	99.3%	
Weighting scheme	$w=1/[\sigma^2(Fo^2)+(0.0217P)^2+0.0969P]$	
Data / restraints / parameters	3595 / 0 / 199	
Goodness-of-fit on F^2	1.093	
Final *R* indices [I>2sigma(I)]	*R*1 = 0.0322, *wR*2 = 0.0889	
R indices (all data)	*R*1 = 0.0400, *wR*2 = 0.0934	
Largest diff. peak and hole	0.338 and -0.216 e.$Å^{-3}$	

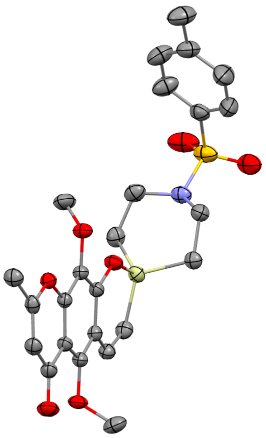

Figure S24. Cristal Structure of **2mN** (ORTEP 50% probability) H atom and solvent $CHCl_3$ was omitted for the Clarity.

Empirical formula	$C_{25}H_{27}SiO_7NS$	
Formula weight	632.99	
Temperature	143(2) K	
Wavelength	0.71075 Å	
Crystal system	Monoclinic	
Space group	C2/c	
Unit cell dimensions	a = 34.354(9) Å	α = 90°
	b = 11.451(3) Å	β = 113.313(4)°
	c = 16.021(4) Å	γ = 90°
Volume	5788(3) $Å^3$	
Z	8	
Density (calculated)	1.453 Mg/m^3	
Absorption coefficient	0.475 mm^{-1}	
F(000)	2624	
Crystal size	0.170 x 0.100 x 0.070 mm^3	
Theta range for data collection	3.136 to 27.491°	
Index ranges	-44<=h<=44, -14<=k<=13, -20<=l<=20	
Reflections collected	22805	
Independent reflections	6550 [*R*(int) = 0.0338]	
Completeness to theta = 25.242°	98.4%	
Weighting scheme	w=1/[σ^2 (Fo ^{2})+(0.0217P)^2+0.0969P]	
Data / restraints / parameters	6550 / 0 / 420	
Goodness-of-fit on F^2	1.036	
Final *R* indices [I>2sigma(I)]	*R*1 = 0.0443, *wR*2 = 0.1058	
R indices (all data)	*R*1 = 0.0617, *wR*2 = 0.1166	
Largest diff. peak and hole	0.289 and -0.274 e.$Å^{-3}$	

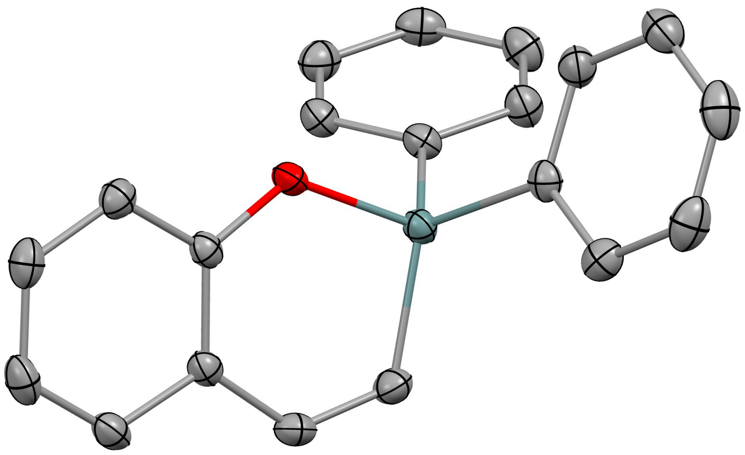

Figure S25. Cristal Structure of **2aQ** (ORTEP 50% probability) H atom was omitted for the Clarity.

Empirical formula	$C_{20}H_{16}GeO$	
Formula weight	344.92	
Temperature	143(2) K	
Wavelength	0.71075 Å	
Crystal system	Monoclinic	
Space group	$P2_1/c$	
Unit cell dimensions	a = 10.627(5) Å	α = 90°
	b = 14.639(6) Å	β = 104.578(6)°
	c = 10.508(5) Å	γ = 90°
Volume	1582.1(12) $Å^3$	
Z	4	
Density (calculated)	1.448 Mg/m^3	
Absorption coefficient	1.935 mm^{-1}	
F(000)	704	
Crystal size	0.200 x 0.200 x 0.200 mm^3	
Theta range for data collection	3.416 to 27.630°	
Index ranges	-13<=h<=13, -16<=k<=18, -13<=l<=13	
Reflections collected	12528	
Independent reflections	3582 [*R*(int) = 0.0275]	
Completeness to theta = 25.242º	97.2%	
Weighting scheme	$w=1/[\sigma^2(Fo^2)+(0.0217P)^2+0.0969P]$	
Data / restraints / parameters	3582 / 0 / 199	
Goodness-of-fit on F^2	1.052	
Final *R* indices [I>2sigma(I)]	*R*1 = 0.0228, *wR*2 = 0.0613	
R indices (all data)	*R*1 = 0.0265, *wR*2 = 0.0624	
Largest diff. peak and hole	0.651 and -0.273 e.$Å^{-3}$	

9. References and Notes

(1) Crabtree, R. H.; Quirk, J. M.; Felkin, H.; Fillebeen-khan, T. An Efficient Synthesis of [Ir(Cod)Cl]$_2$and Its Reaction with PMe_2Ph to Give*FAC*-[IrH($PMe_2C_6H_4$)(PMe_2Ph)$_3$]. *Synth. React. Inorg. Met.-Org. Nano-Met. Chem.* **1982**, *12* (4), 407–413.

(2) Giordano, G.; Crabtree, R. H.; Heintz, R. M.; Forster, D.; Morris, D. E. Di-μ-Chloro-Bis(H4 -1,5-Cyclooctadiene)-Dirhodium(I). In *Inorganic Syntheses*; Inorganic Syntheses; John Wiley & Sons, Inc.: Hoboken, NJ, USA, 2007; pp 88–90.

(3) Giordano, G.; Crabtree, R. H.; Heintz, R. M.; Forster, D.; Morris, D. E. Di-μ-Chloro-Bis(H4-1,5-Cyclooctadlene) Dirhodium(I). In *Inorganic Syntheses*; Inorganic Syntheses; John Wiley & Sons, Inc.: Hoboken, NJ, USA, 2007; pp 218–220.

(4) Sevov, C. S.; Hartwig, J. F. Iridium-Catalyzed Oxidative Olefination of Furans with Unactivated Alkenes. *J. Am. Chem. Soc.* **2014**, *136* (30), 10625–10631.

(5) Suto, Y.; Tsuji, R.; Kanai, M.; Shibasaki, M. Cu(I)-Catalyzed Direct Enantioselective Cross Aldol-Type Reaction of Acetonitrile. *Org. Lett.* **2005**, *7* (17), 3757–3760.

(6) Ma, M.-L.; Peng, Z.-H.; Chen, L.; Guo, Y.; Chen, H.; Li, X.-J. Synthesis of New MeO-BIPHEP-type Chiral Diphosphines by an Improved Way. *Chin. J. Chem.* **2006**, *24* (10), 1391−1396.

(7) Takeuchi, T.; Shishido, R.; Kubota, K.; Ito, H. Synthesis of Hydrosilylboronates via the Monoborylation of a Dihydrosilane Si-H Bond and Their Application for the Generation of Dialkylhydrosilyl Anions. *Chem. Sci.* **2021**, *12* (35), 11799–11804.

(8) Fan, X.; Zhang, M.; Gao, Y.; Zhou, Q.; Zhang, Y.; Yu, J.; Xu, W.; Yan, J.; Liu, H.; Lei, Z.; Ter, Y. C.; Chanmungkalakul, S.; Lum, Y.; Liu, X.; Cui, G.; Wu, J. Stepwise On-Demand Functionalization of Multihydrosilanes Enabled by a Hydrogen-Atom-Transfer Photocatalyst Based on Eosin Y. *Nat. Chem.* **2023**, *15* (5), 666–676.

(9) Šimarek, A.; Lamač, M.; Horáček, M.; Pinkas, J. $B(C_6F_5)_3$ Catalysis Accelerates the Hydrosilane Chlorination by Ph_3CCl. *Appl. Organomet. Chem.* **2018**, *32* (8), e4442.

(10) Sattler, W.; Ruccolo, S.; Rostami Chaijan, M.; Nasr Allah, T.; Parkin, G. Hydrosilylation of Aldehydes and Ketones Catalyzed by a Terminal Zinc Hydride Complex, [K3-Tptm]ZnH. *Organometallics* **2015**, *34* (19), 4717–4731.

(11) Wei, B.; Li, H.; Yin, J.; Zhang, W.-X.; Xi, Z. Reaction of Dilithio Reagents with PhSiH3: Formation of Siloles and 3-Silacyclopentenes. *J. Org. Chem.* **2015**, *80* (17), 8758–8762.

(12) Corey, J. Y.; Corey, E. R. 9,9-Dimethyl-9H-Tribenzo [b,d,f]Silepin: A Stereochemically Rigid Silicon Heterocycle. *Tetrahedron Lett.* **1972**, *13* (46), 4669–4672.

(13) Berzins, T.; DeLahay, P. Theory of Irreversible Polarographic Waves—Case of Two Consecutive Electrochemical Reactions. *J. Am. Chem. Soc.* **1953**, *75* (22), 5716–5720.

(14) Savela, R.; Zawartka, W.; Leino, R. Iron-Catalyzed Chlorination of Silanes. *Organometallics* **2012**, *31* (8), 3199–3206.

(15) Torigoe, T.; Ohmura, T.; Suginome, M. Iridium-Catalyzed Intramolecular Methoxy C-H Addition to Carbon-Carbon Triple Bonds: Direct Synthesis of 3-Substituted Benzofurans from o-Methoxyphenylalkynes. *Chemistry* **2016**, *22* (30), 10415–10419.

(16) Laye, C.; Lusseau, J.; Robert, F.; Landais, Y. The Trityl-cation Mediated Phosphine Oxides Reduction. *Adv. Synth. Catal.* **2021**, *363* (12), 3035−3043.

(17) Tanaka, S.; Tomokuni, H. Syntheses of Tetraoxaquaterene Derivatives. *J. Heterocycl. Chem.* **1991**, *28* (4), 991–994.

(18) Barker, P.; Finke, P.; Thompson, K. Preparation and Cyclization of Aryloxyacetaldehyde Acetals; A General Synthesis of 2,3-Unsubstituted Benzofurans. *Synth. Commun.* **1989**, *19* (1–2), 257–265.

(19) Ishiyama, T.; Murata, M.; Miyaura, N. Palladium(0)-Catalyzed Cross-Coupling Reaction of Alkoxydiboron with Haloarenes: A Direct Procedure for Arylboronic Esters. *J. Org. Chem.* **1995**, *60* (23), 7508–7510.

(20) Kashima, C.; Tomotake, A.; Omote, Y. Photolysis of the Ozonide Derived from 1,4-Benzodioxins. Synthesis of Labile o-Benzoquinones. *J. Org. Chem.* **1987**, *52* (25), 5616–5621.

(21) Coudert, G.; Guillaumet, G.; Loubinoux, B. Synthese de benzodioxines-1,4. *Tetrahedron Lett.* **1978**, *19* (12), 1059–1062.

(22) Guo, J.; Liu, S.; Jing, J.; Fan, Y.; Fu, Y.; Liu, S.; Wang, W.; Gao, L.; Song, Z. Controllable Si-C Bond Formation from Trihydrosilanes En Route to Synthesis of 1,4-Azasilinanes with Diverse Silyl Functionalities. *Org. Lett.* **2023**, *25* (40), 7428–7433.
(23) Zhang, J.; Park, S.; Chang, S. Catalytic Access to Bridged Sila- N-Heterocycles from Piperidines via Cascade Sp3 and Sp2 C-Si Bond Formation. *J. Am. Chem. Soc.* **2018**, *140* (41), 13209–13213.
(24) Zhou, L.; Shen, Y.-B.; An, X.-D.; Li, X.-J.; Li, S.-S.; Liu, Q.; Xiao, J. Redox-Neutral β-C(Sp3)-H Functionalization of Cyclic Amines via Intermolecular Hydride Transfer. *Org. Lett.* **2019**, *21* (21), 8543–8547.
(25) Batchelor, R. J.; Birchall, T. Carbon-13 NMR of Arylgermanes and Arylgermyl Anions. Main-Group Elements as Anionic .Pi.-Donor Substituents. 2. *J. Am. Chem. Soc.* **1983**, *105* (12), 3848–3852.
(26) Rubin, M.; Schwier, T.; Gevorgyan, V. Highly Efficient B(C(6)F(5))(3)-Catalyzed Hydrosilylation of Olefins. *J. Org. Chem.* **2002**, *67* (6), 1936–1940.
(27) Gaussian 16, Revision C.01, M. J. Frisch, G. W. Trucks, H. B. Schlegel, G. E. Scuseria, M. A. Robb, J. R. Cheeseman, G. Scalmani, V. Barone, G. A. Petersson, H. Nakatsuji, X. Li, M. Caricato, A. V. Marenich, J. Bloino, B. G. Janesko, R. Gomperts, B. Mennucci, H. P. Hratchian, J. V. Ortiz, A. F. Izmaylov, J. L. Sonnenberg, D. Williams-Young, F. Ding, F. Lipparini, F. Egidi, J. Goings, B. Peng, A. Petrone, T. Henderson, D. Ranasinghe, V. G. Zakrzewski, J. Gao, N. Rega, G. Zheng, W. Liang, M. Hada, M. Ehara, K. Toyota, R. Fukuda, J. Hasegawa, M. Ishida, T. Nakajima, Y. Honda, O. Kitao, H. Nakai, T. Vreven, K. Throssell, J. A. Montgomery, Jr., J. E. Peralta, F. Ogliaro, M. J. Bearpark, J. J. Heyd, E. N. Brothers, K. N. Kudin, V. N. Staroverov, T. A. Keith, R. Kobayashi, J. Normand, K. Raghavachari, A. P. Rendell, J. C. Burant, S. S. Iyengar, J. Tomasi, M. Cossi, J. M. Millam, M. Klene, C. Adamo, R. Cammi, J. W. Ochterski, R. L. Martin, K. Morokuma, O. Farkas, J. B. Foresman, and D. J. Fox, Gaussian, Inc., Wallingford CT, 2016.

10. ^{1}H and [13] NMR Spectra of New Compounds

Figure S26: 2aB
Figure S27: 2aC
Figure S28: 2aD
Figure S29: 2aE
Figure S30: 2aF
Figure S31: 2aG
Figure S32: 2aH
Figure S33: 2aI
Figure S34: 2aJ
Figure S35: 2aK
Figure S36: 2aL
Figure S37: 2aM
Figure S38: 2aA
Figure S39: 2bA
Figure S40: 2cA
Figure S41: 2dA
Figure S42: 2eA
Figure S43: 2fA
Figure S44: 2gA
Figure S45: 2hA
Figure S46: 2iA
Figure S47: 2jJ
Figure S48: 2kJ
Figure S49: 2lA
Figure S50: 2mA
Figure S51: 4aJ
Figure S52: 4bJ
Figure S53: 4cJ
Figure S54: 4dJ
Figure S55: 4eA
Figure S56: 4fJ
Figure S57: 6J
Figure S58: 8J
Figure S59: 10J
Figure S60: 12J
Figure S61: 2mN
Figure S62: 2aP
Figure S63: 2aQ
Figure S64: 15
Figure S65: tris(3,4-dimethylphenyl)silane
Figure S66: bis(3,4-dimethylphenyl)(phenyl)silane
Figure S67: (3,4-dimethylphenyl)diphenylsilane
Figure S68: 2aJ-*d*
Figure S69: A11
Figure S70: A15
Figure S71: A16
Figure S72: A19
Figure S73: B
Figure S74: C
Figure S75: D
Figure S76: F
Figure S77: H
Figure S78: I
Figure S79: L
Figure S80: M
Figure S81: 1l
Figure S82: P
Figure S83: Q
Figure S84: 14
Figure S85: 16
Figure S86: 1a-*d*
Figure S87: J-*d*
Figure S88: 17aJ-*d*

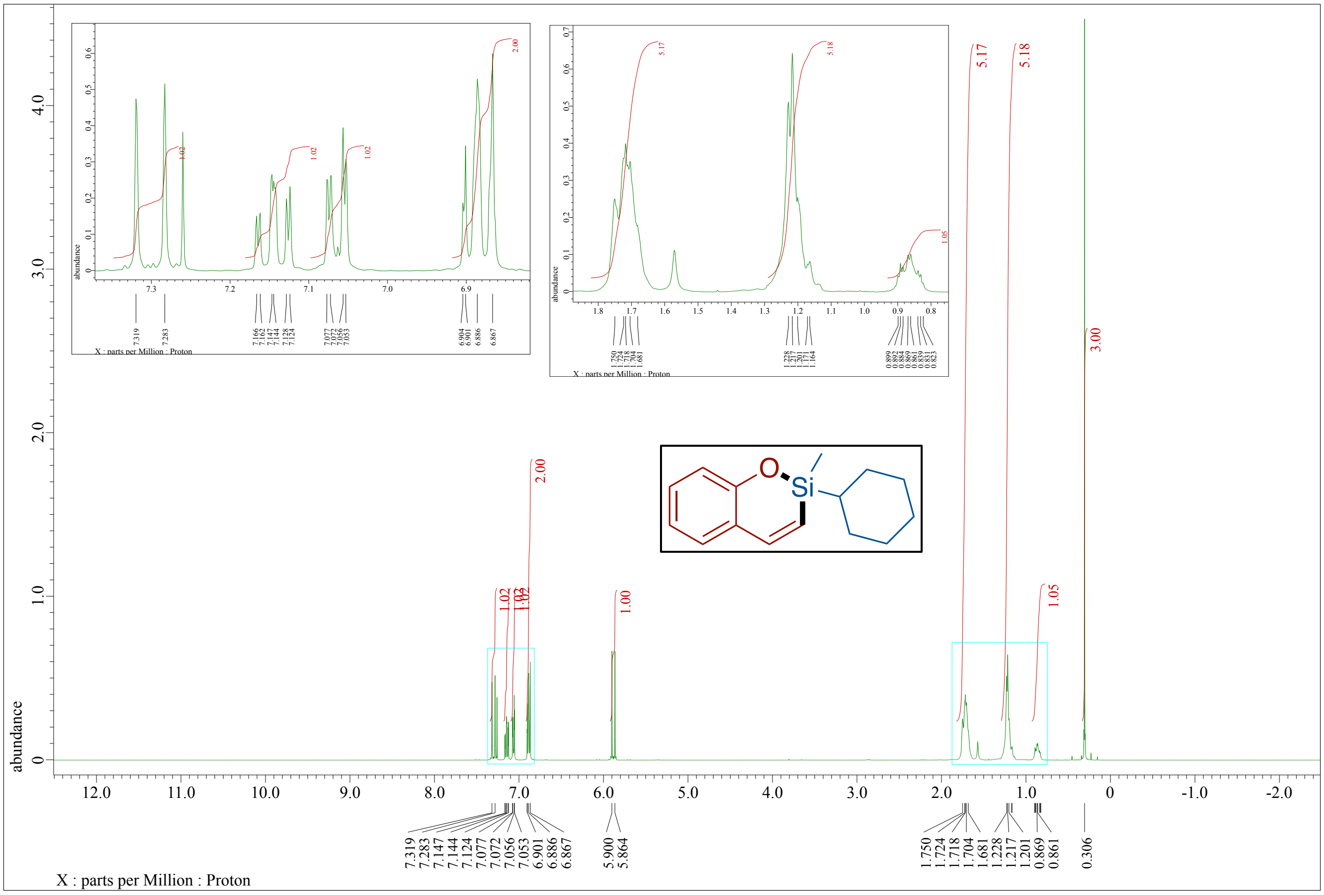

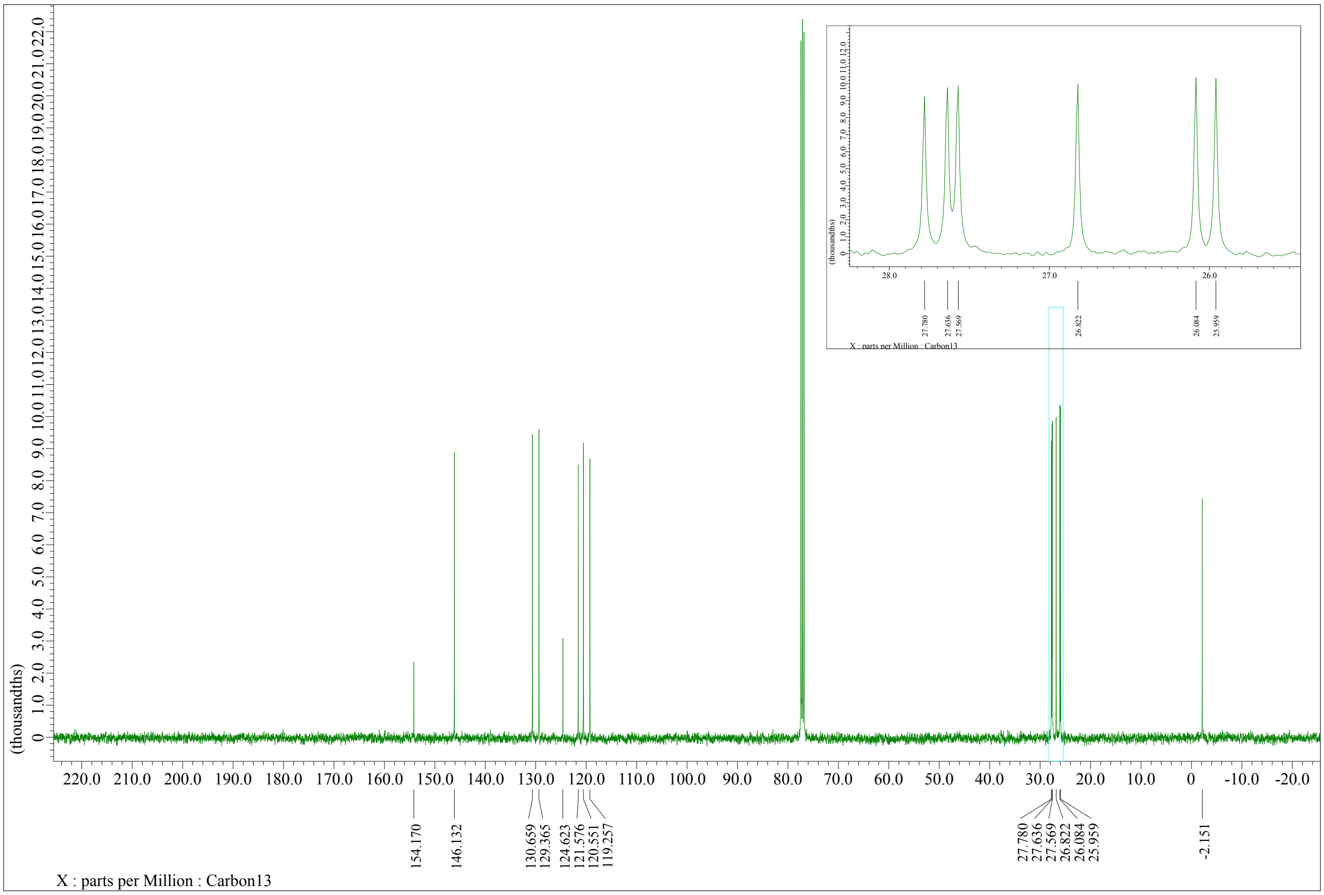

Figure S26. ^{1}H and ^{13}C NMR of **2aB**.

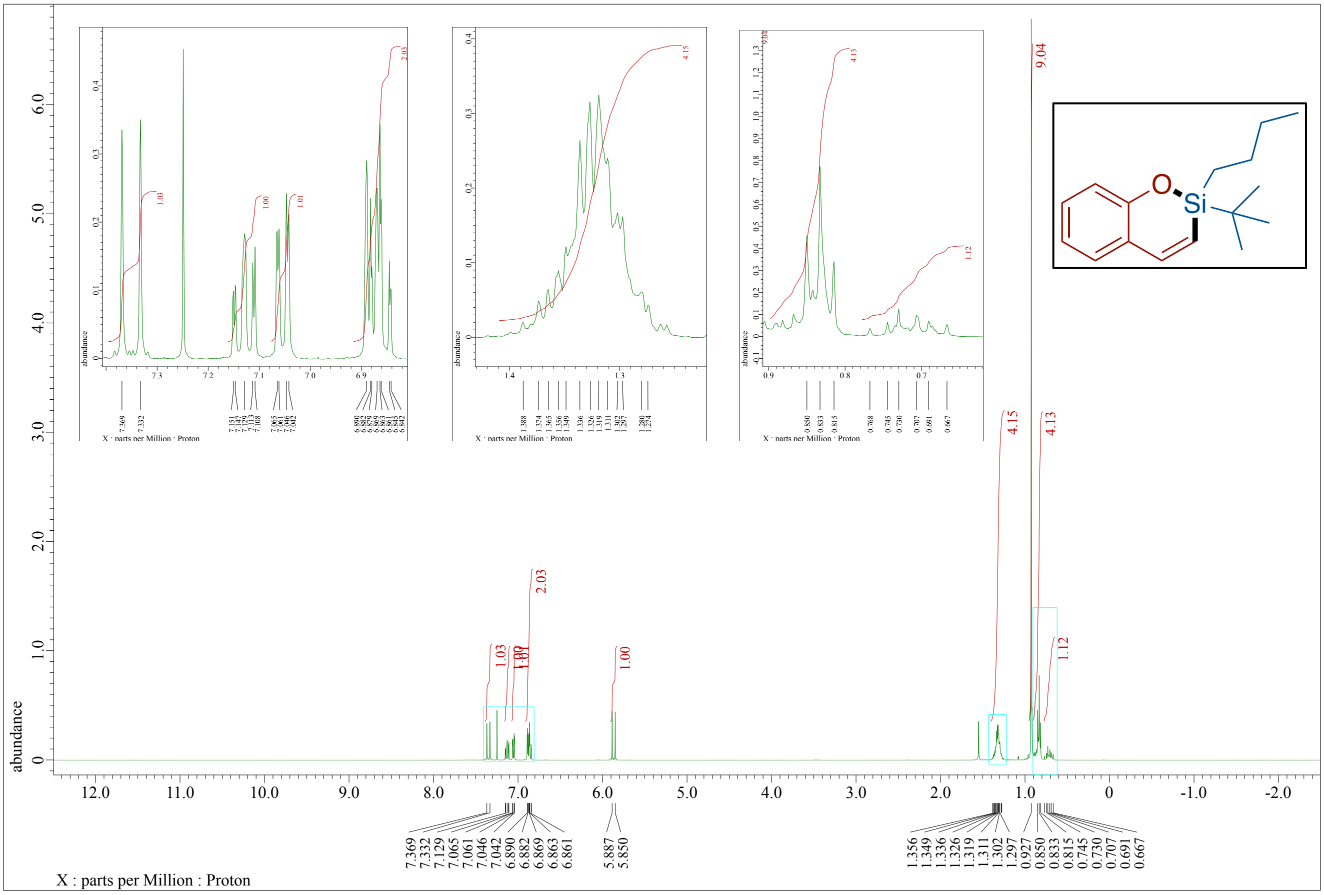

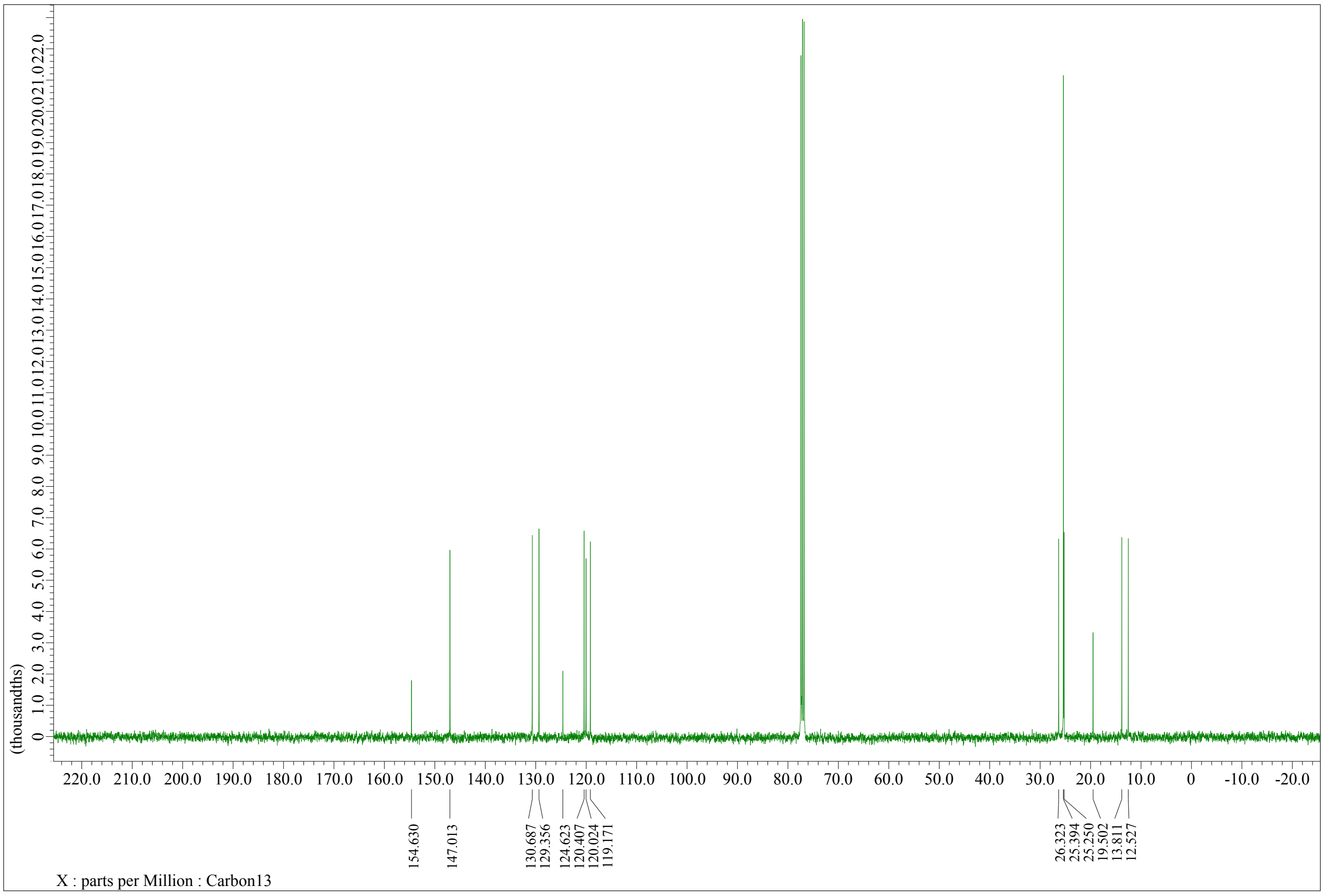

Figure S27. ^{1}H and ^{13}C NMR of **2aC**.

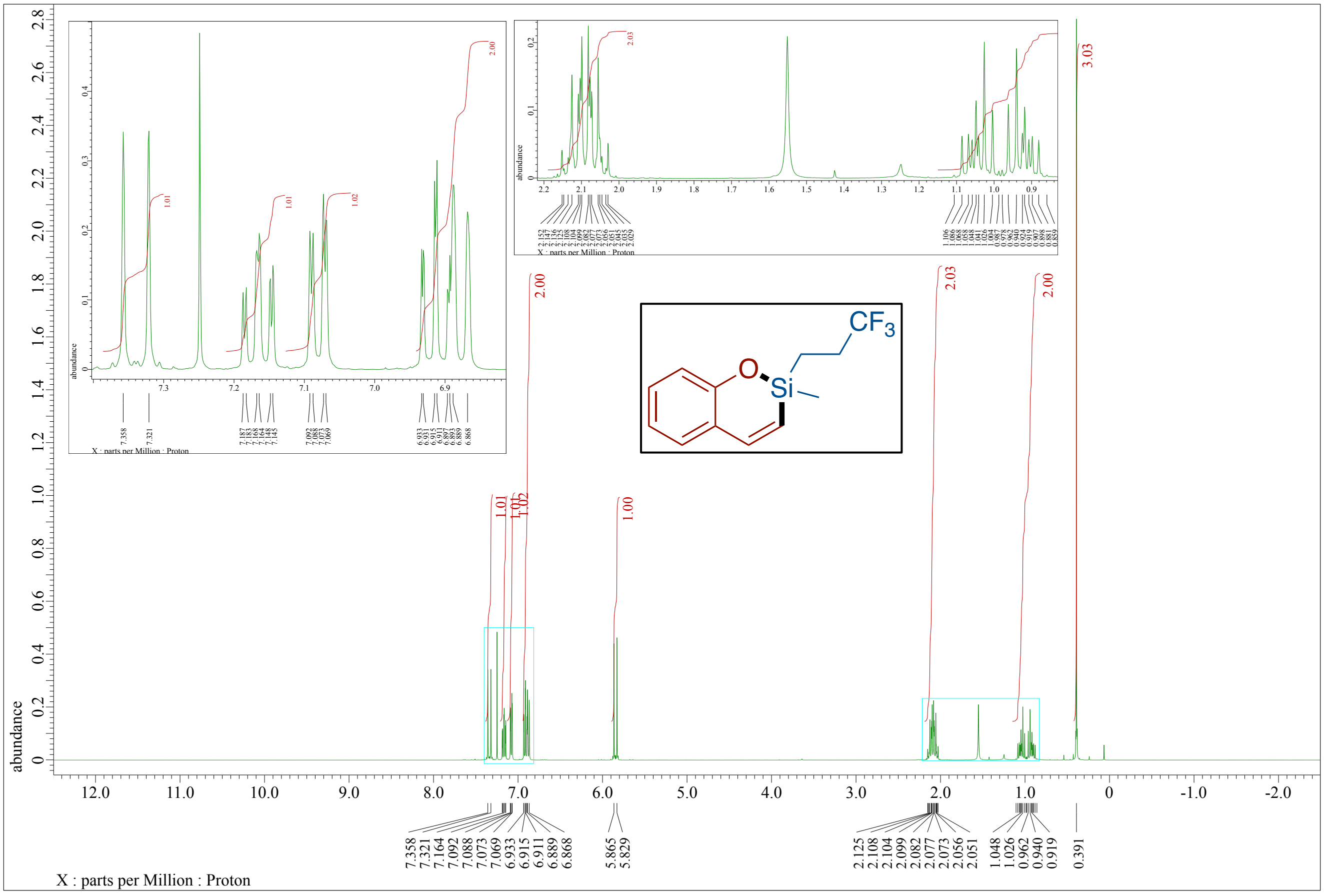

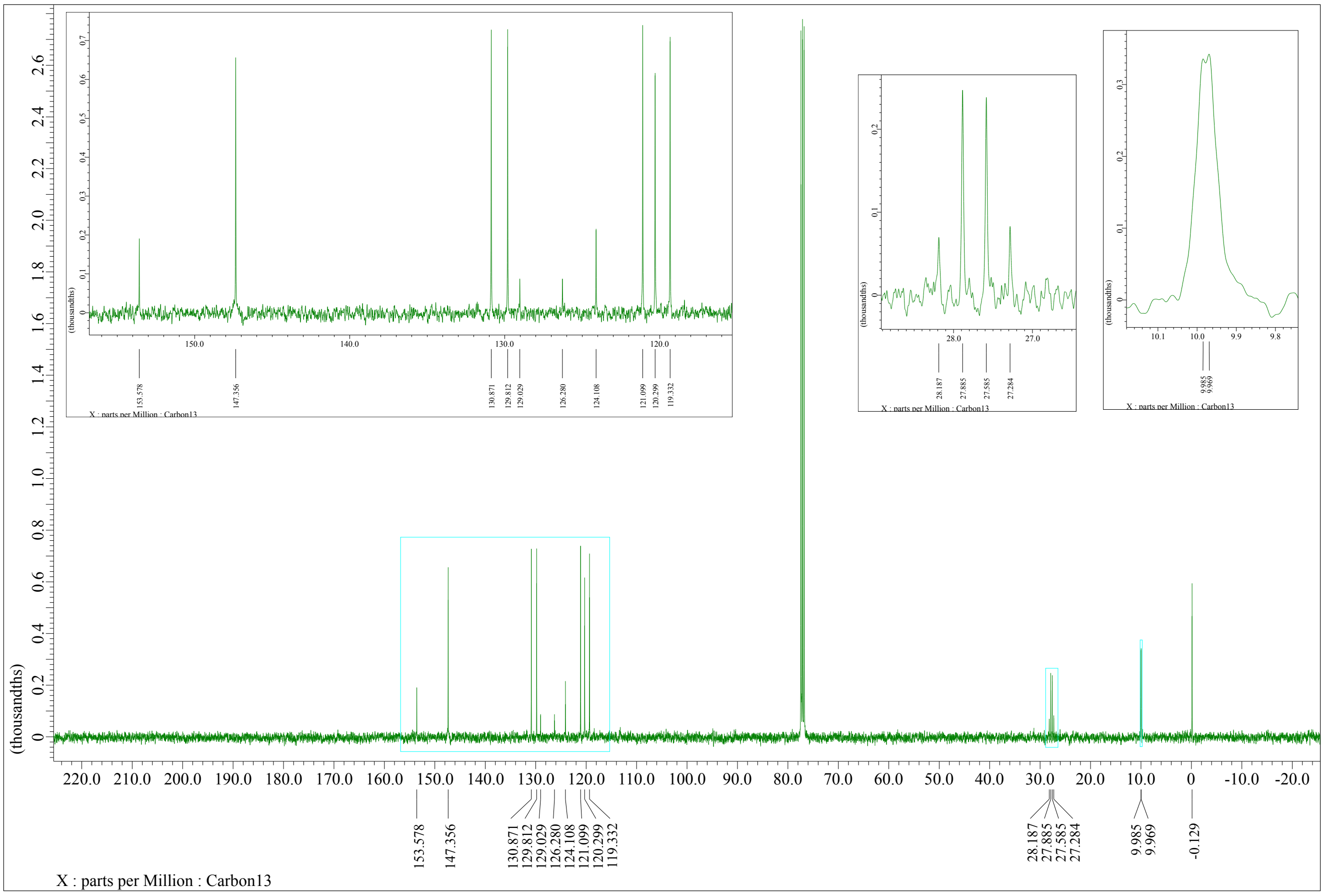

Figure S28. ^{1}H and ^{13}C NMR of **2aD**.

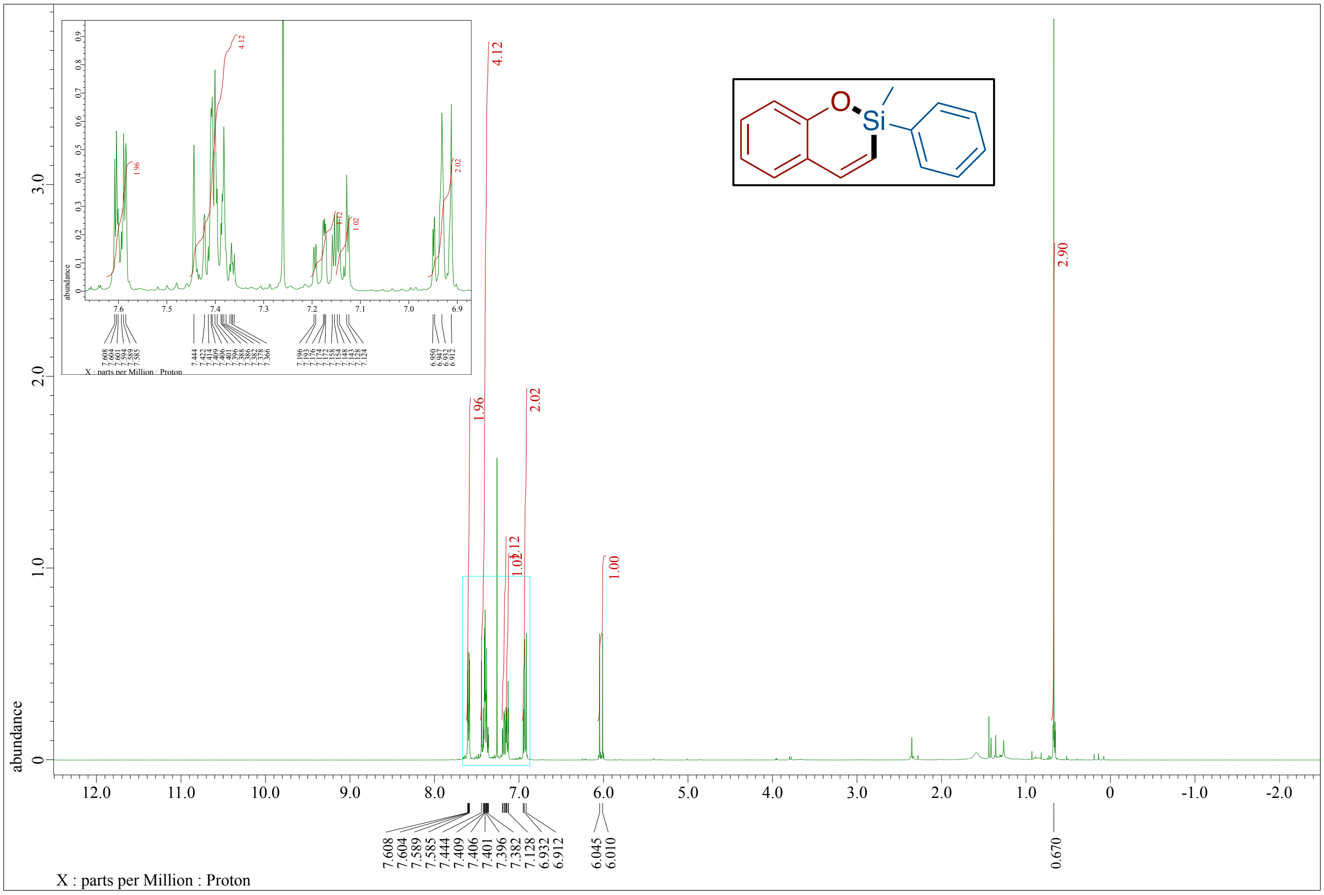

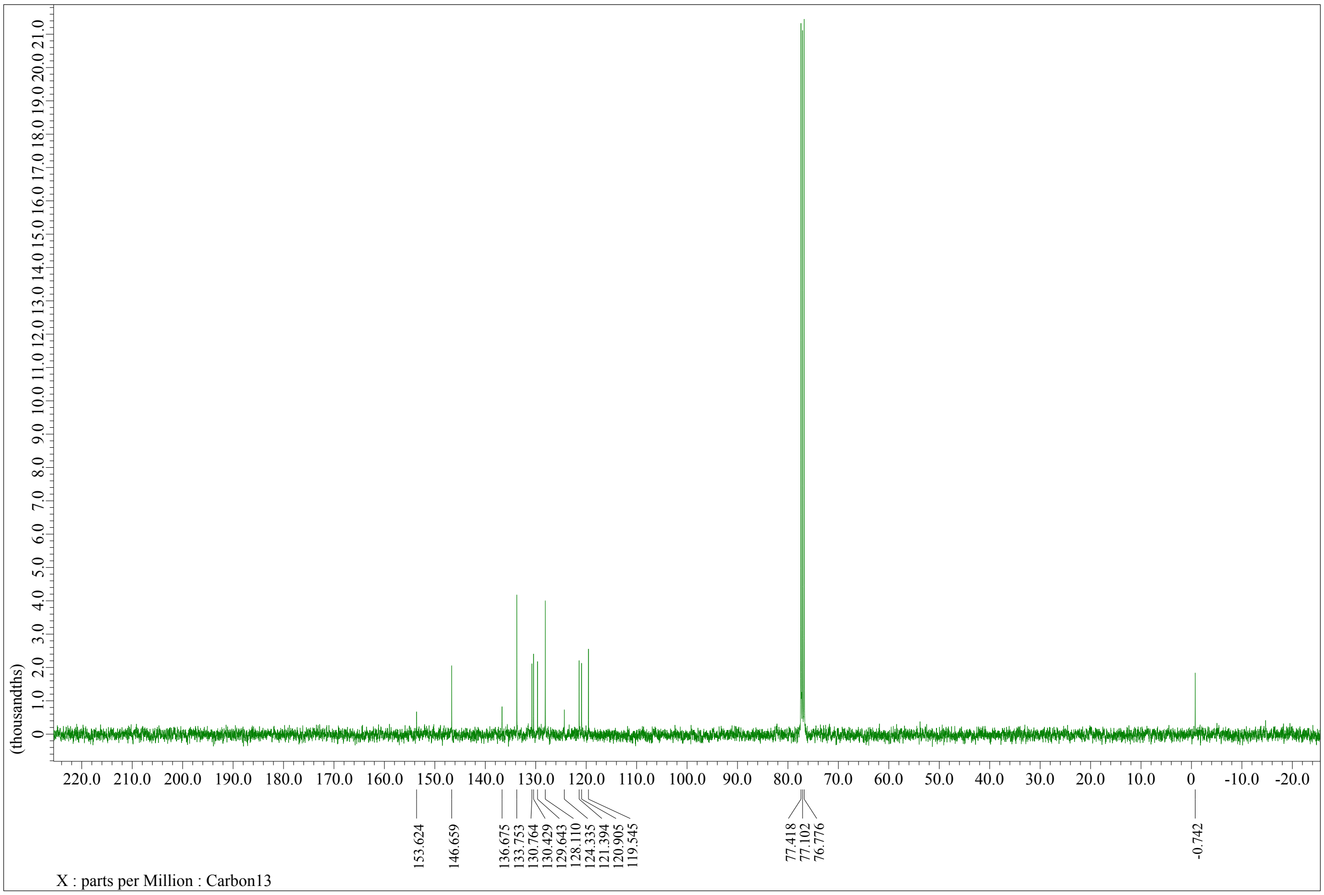

Figure S29. ^{1}H and ^{13}C NMR of **2aE**.

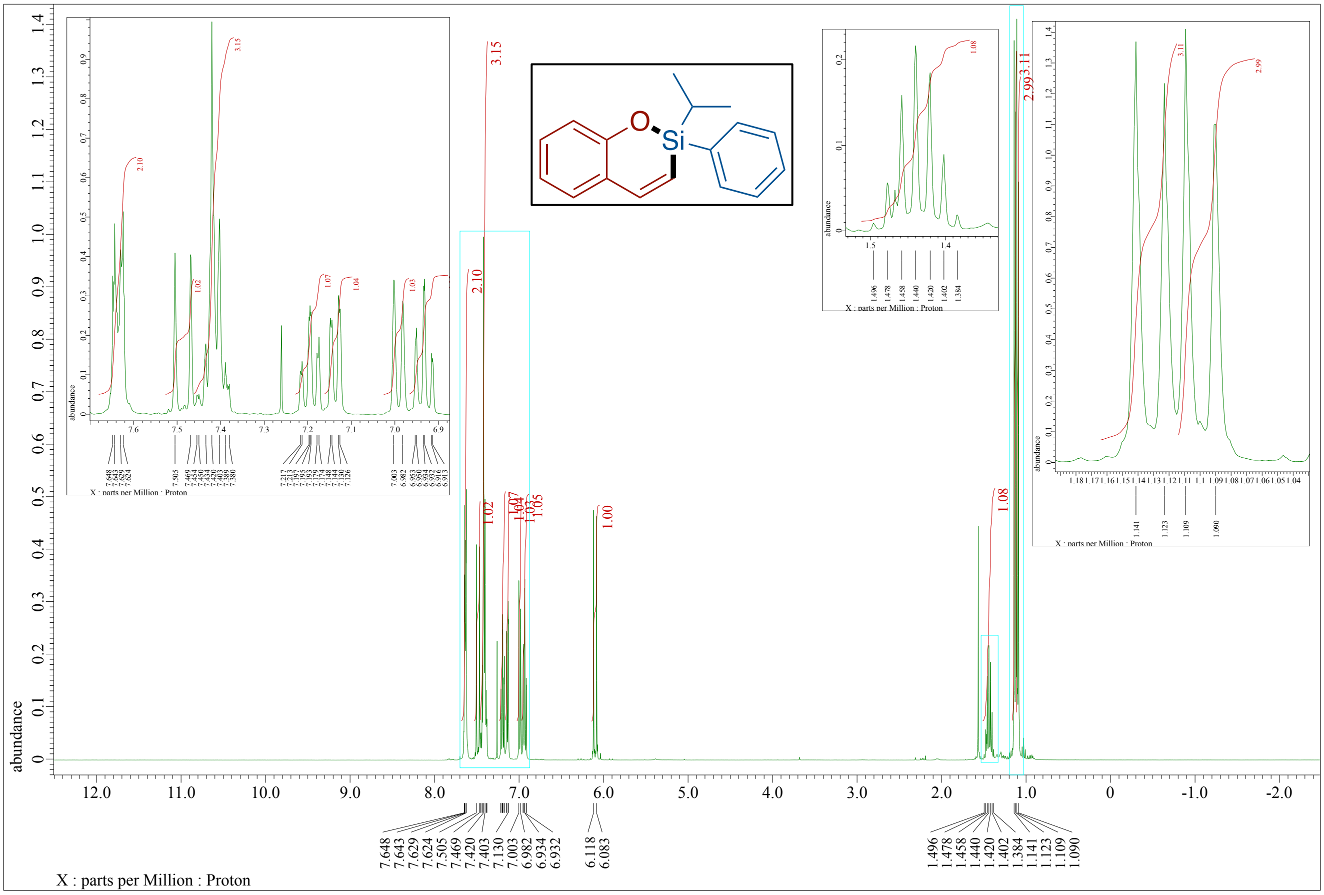

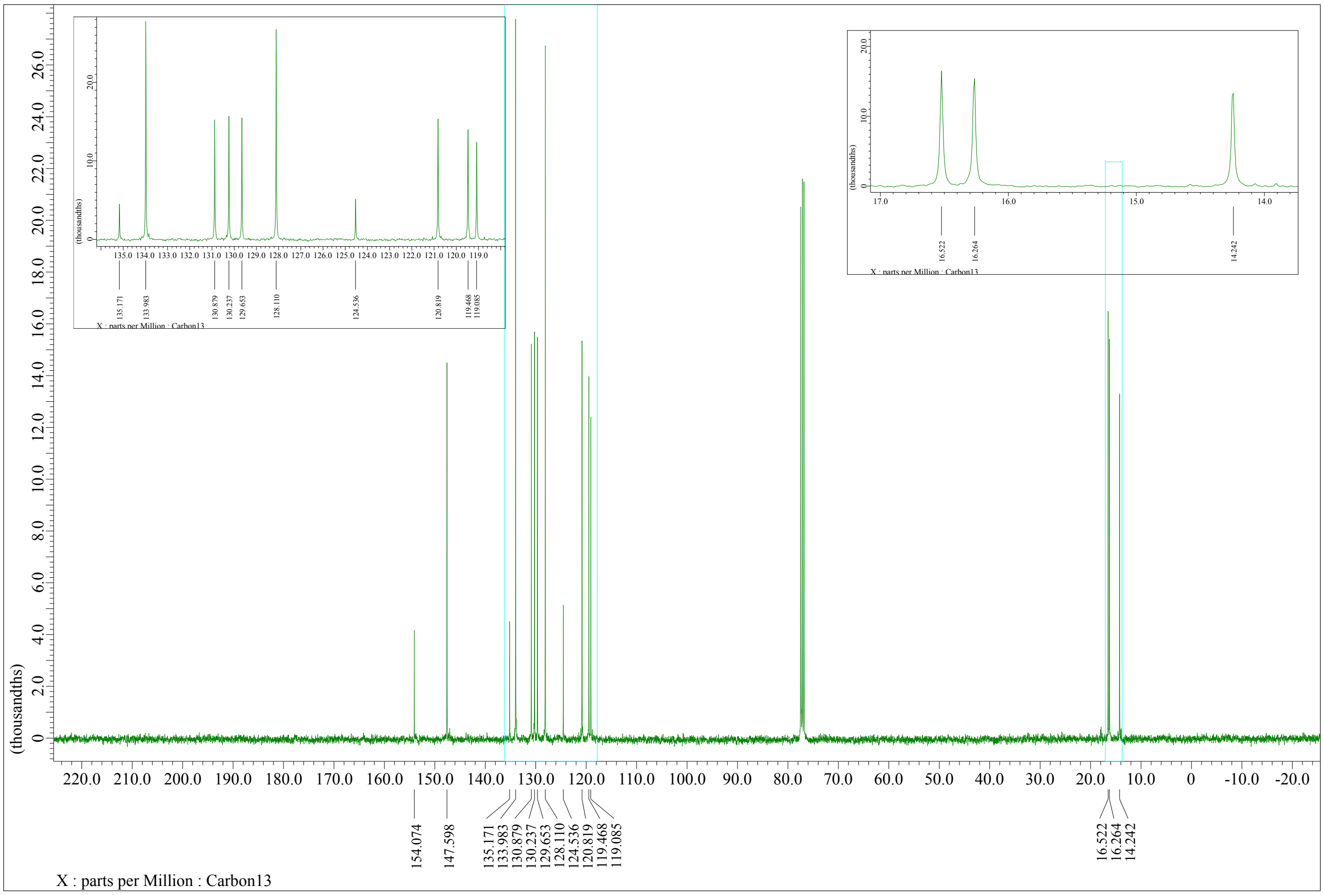

Figure S30. ^{1}H and ^{13}C NMR of **2aF**.

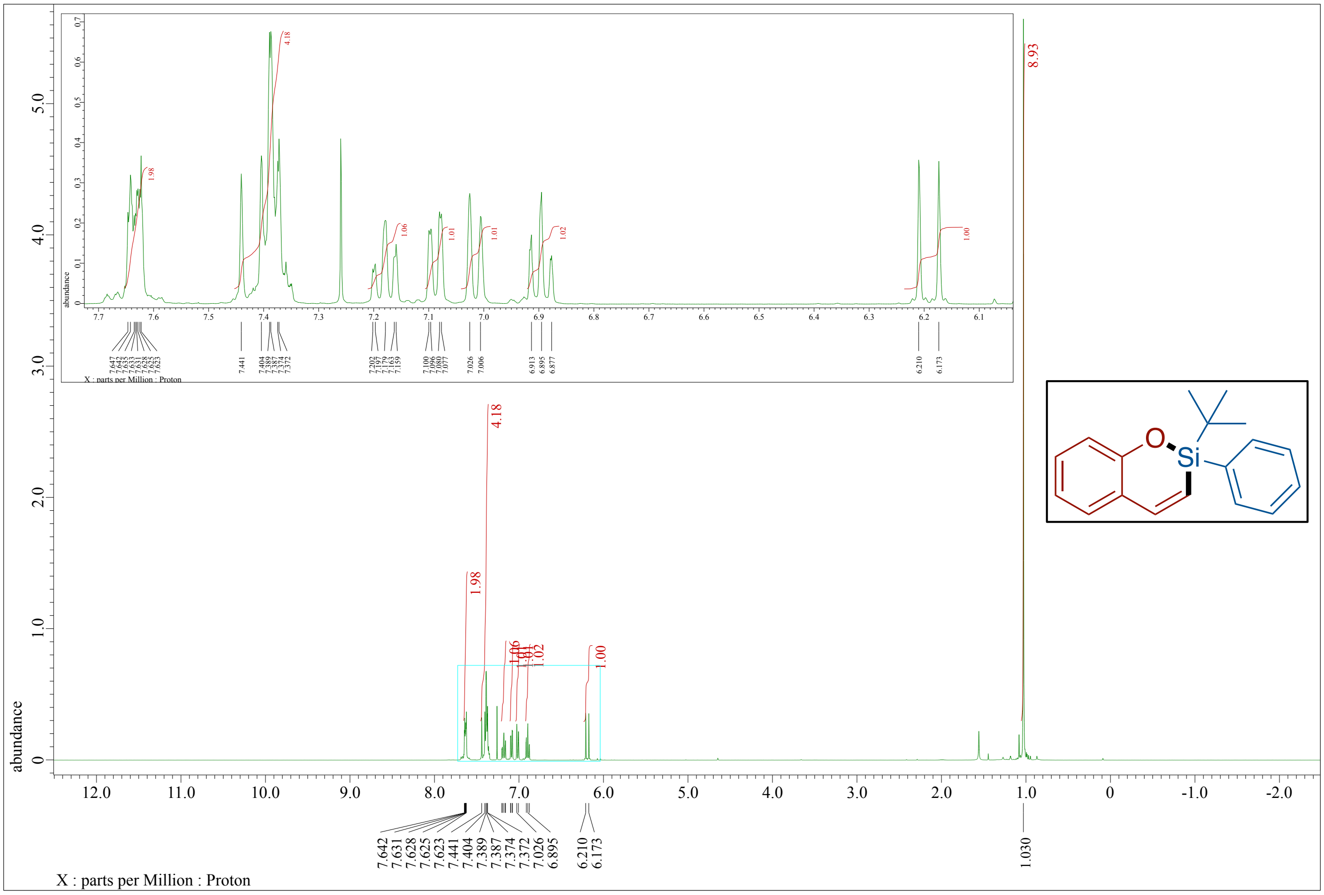

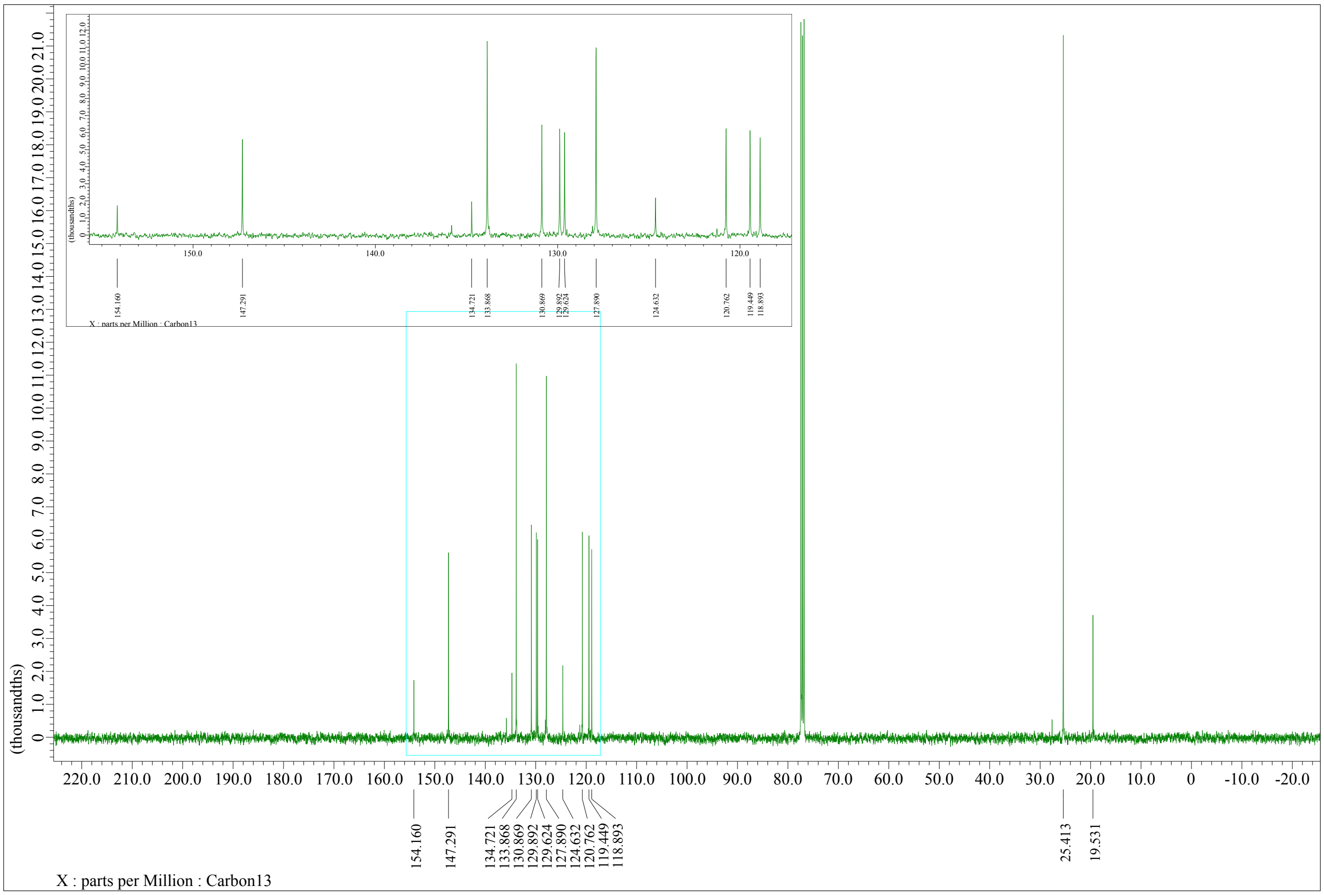

Figure S31. ^{1}H and ^{13}C NMR of 2**aG**.

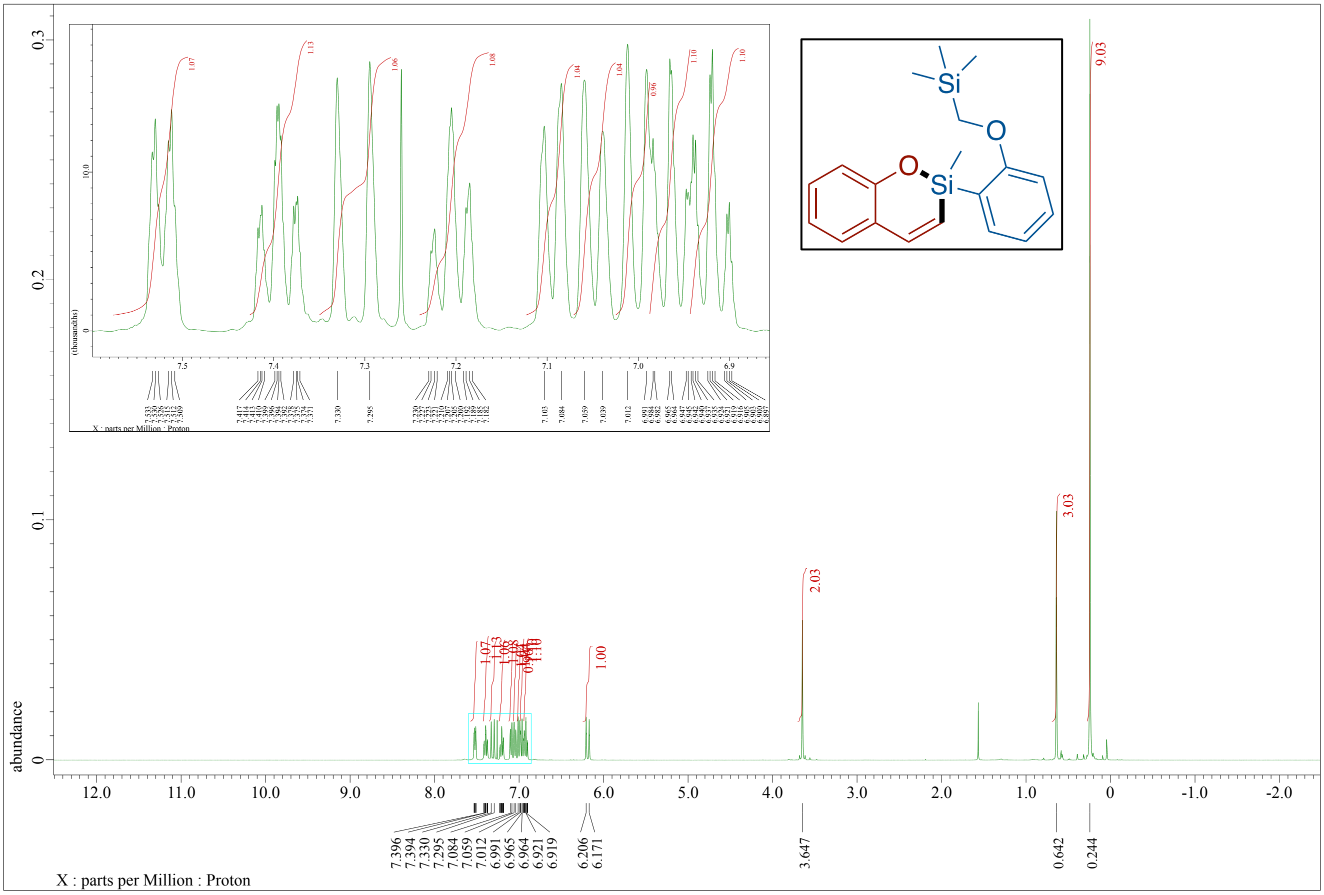

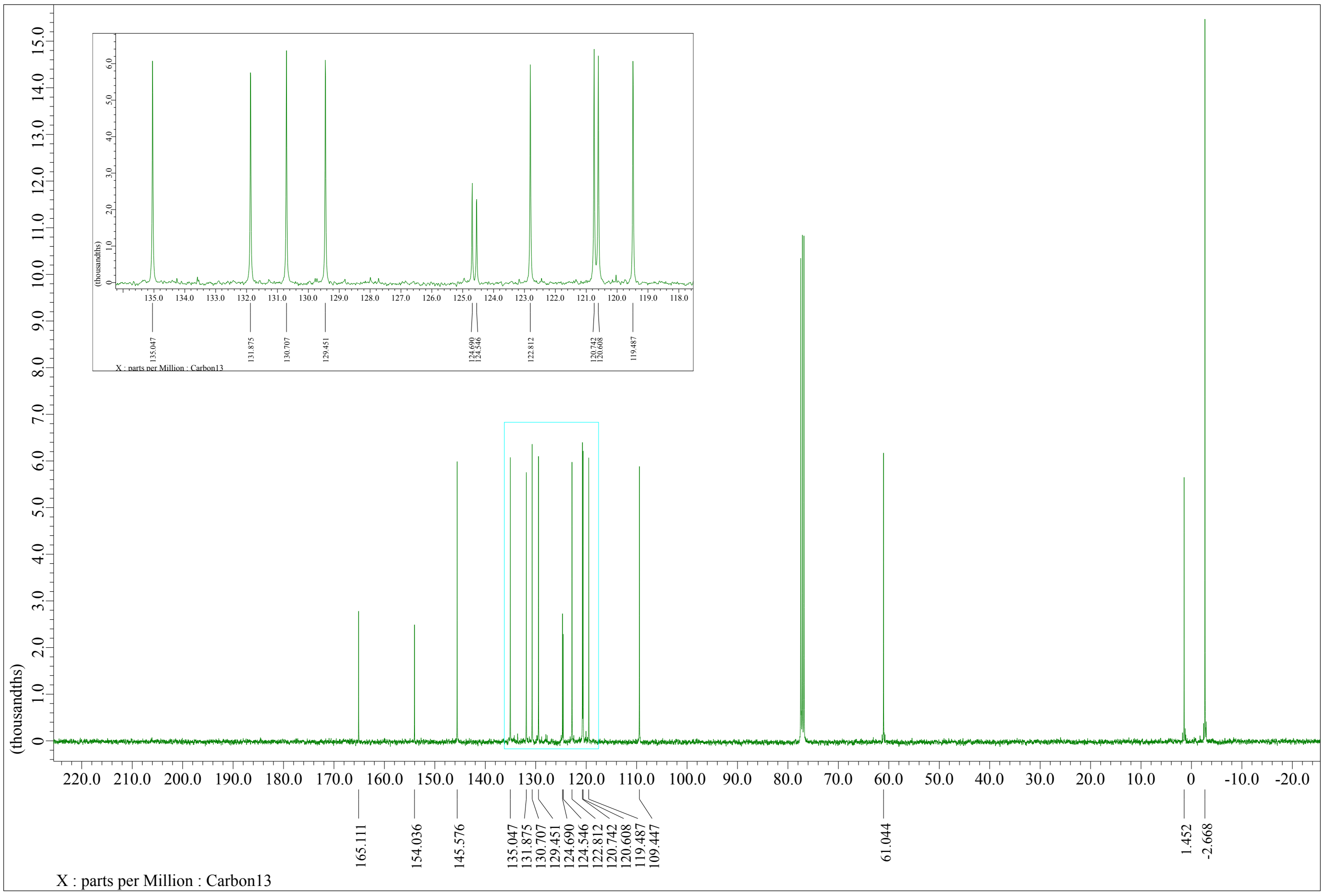

Figure S32. ^{1}H and ^{13}C NMR of **2aH**.

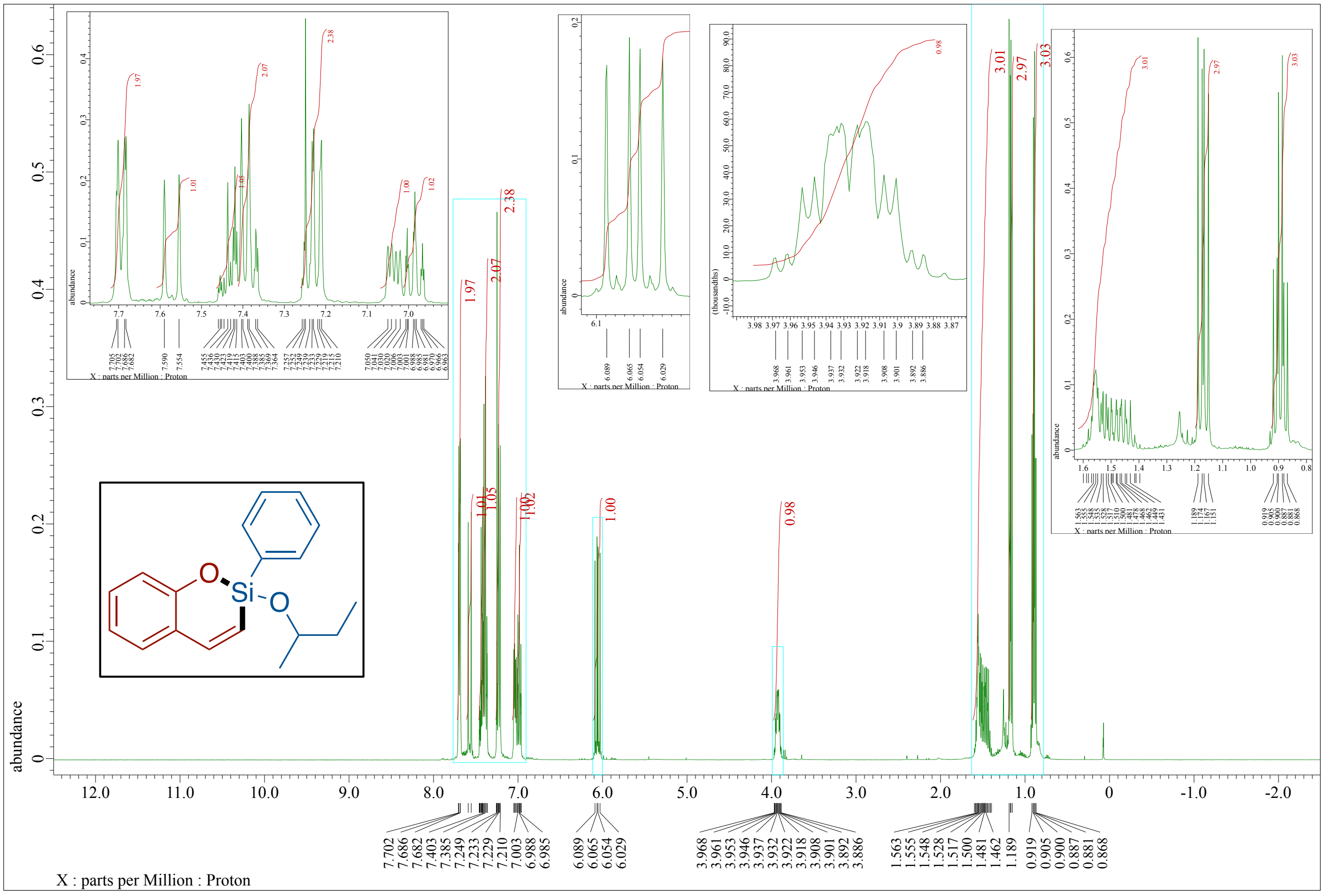

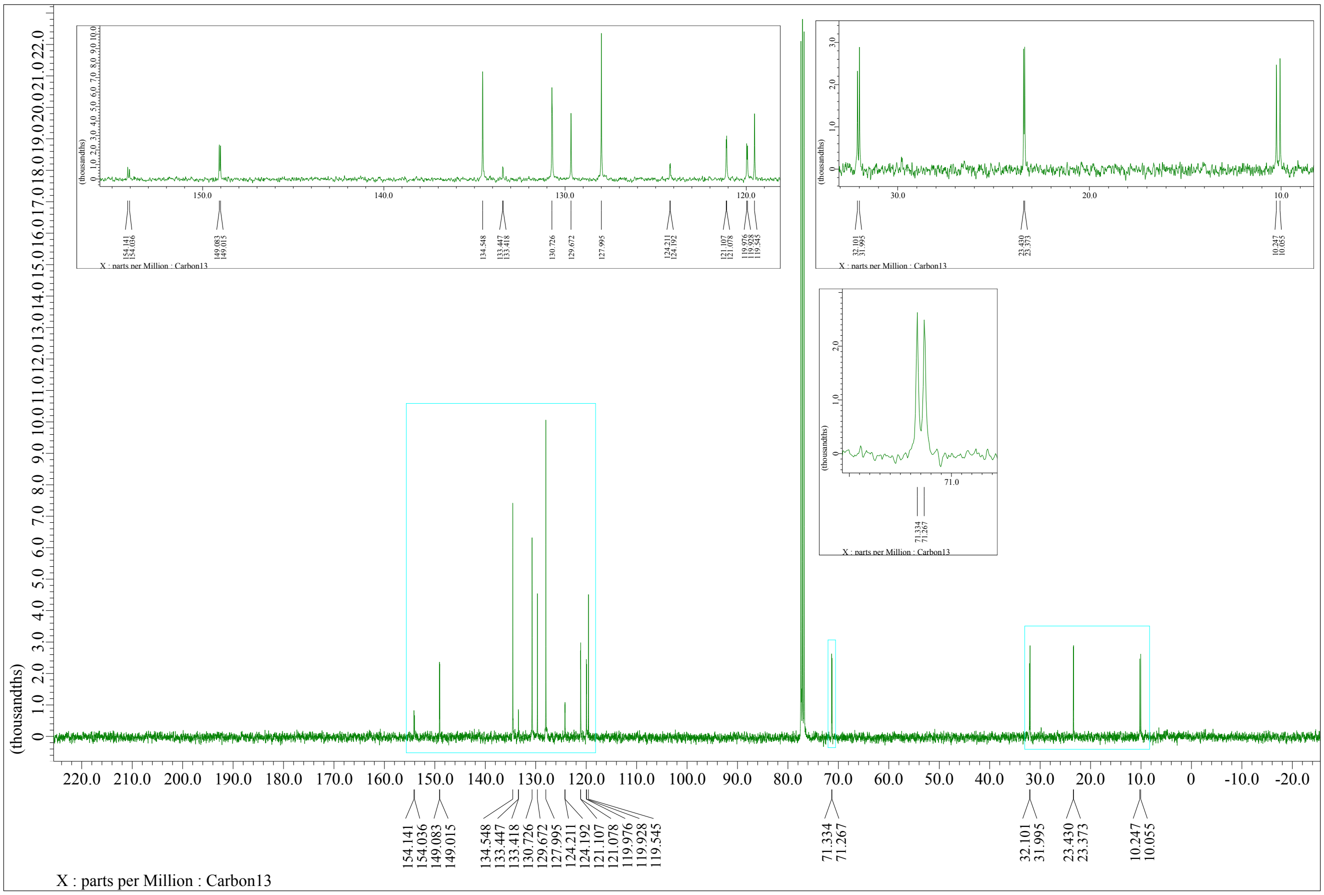

Figure S33. ^{1}H and ^{13}C NMR of **2aI**.

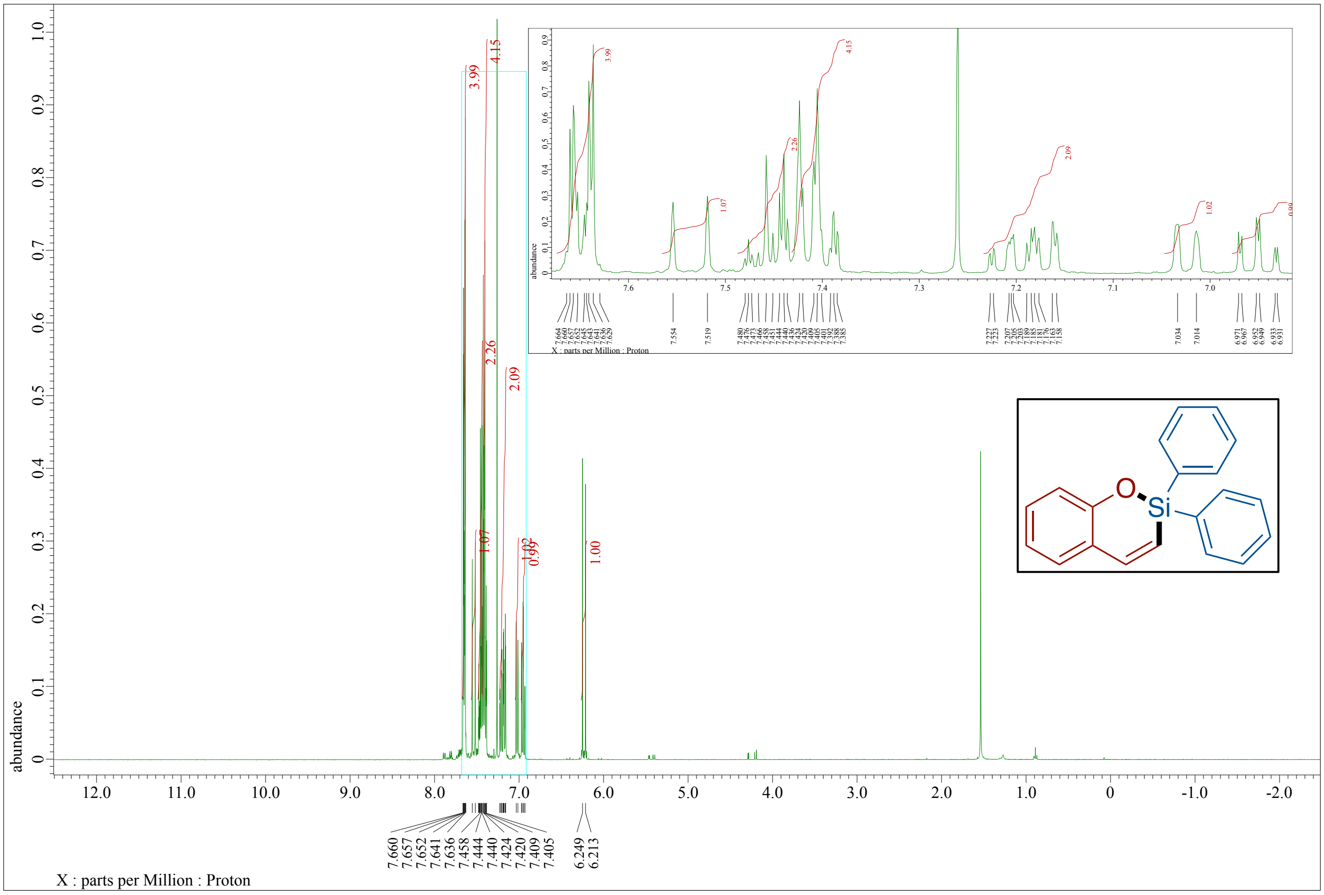

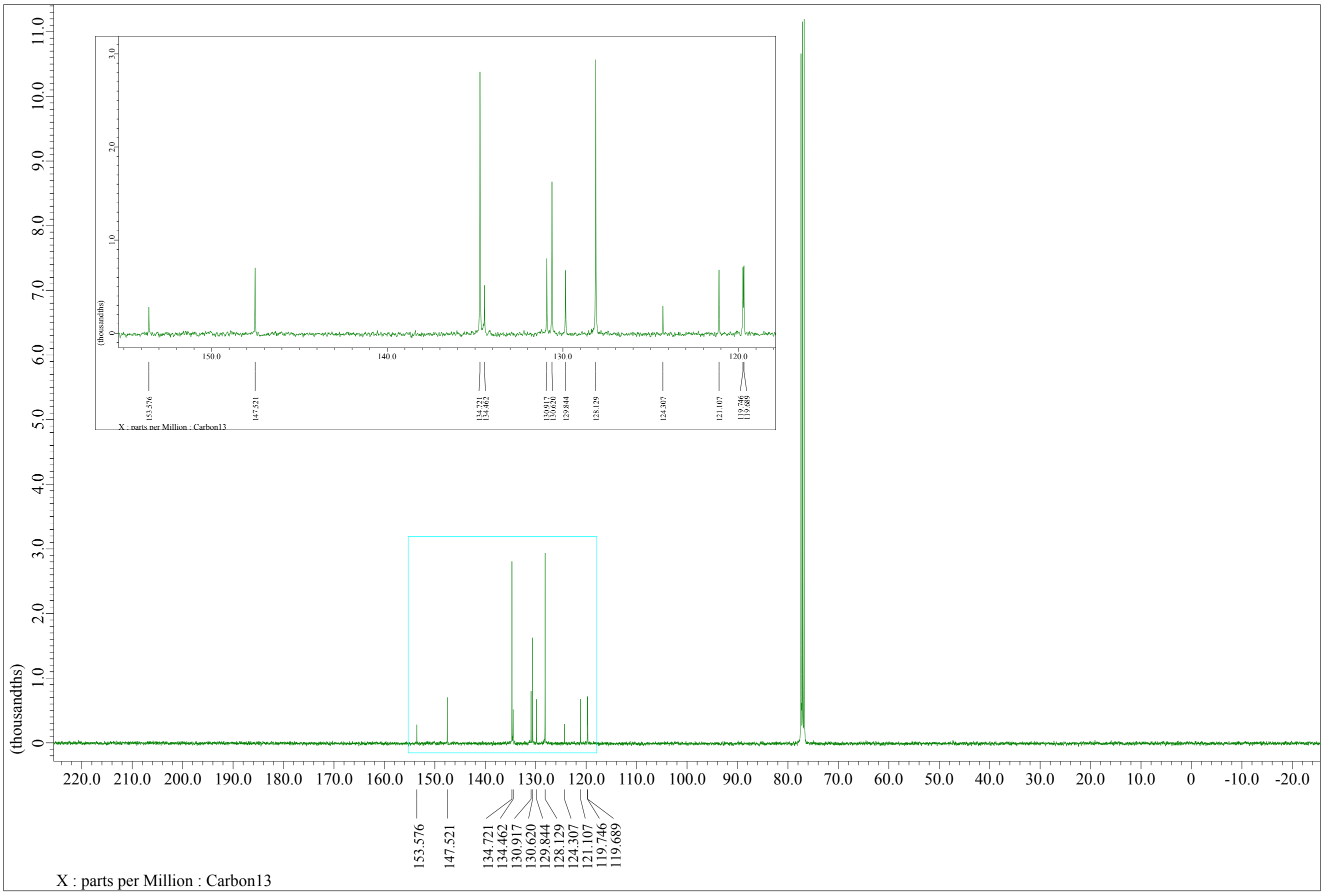

Figure S34. ^{1}H and ^{13}C NMR of **2aJ**.

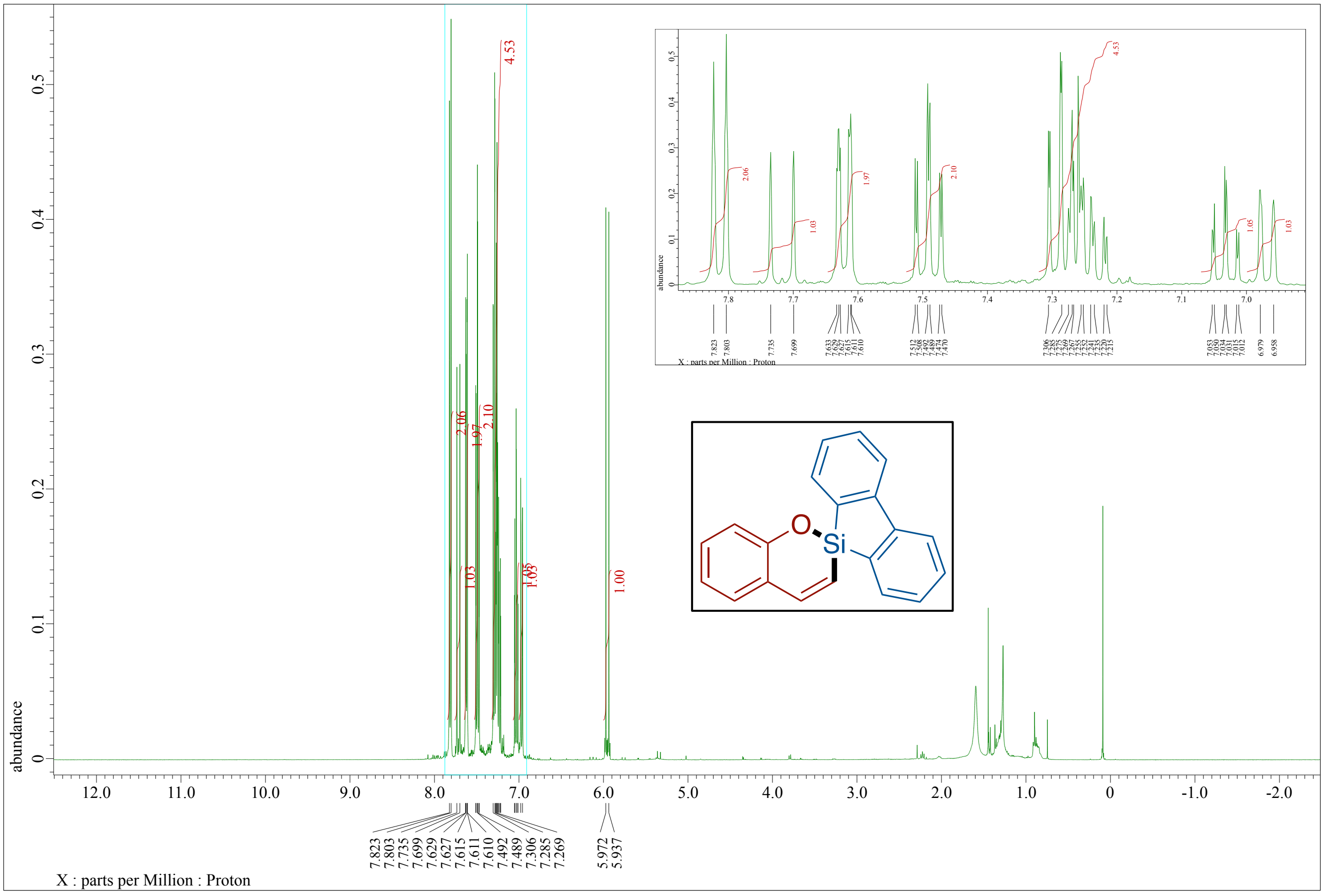

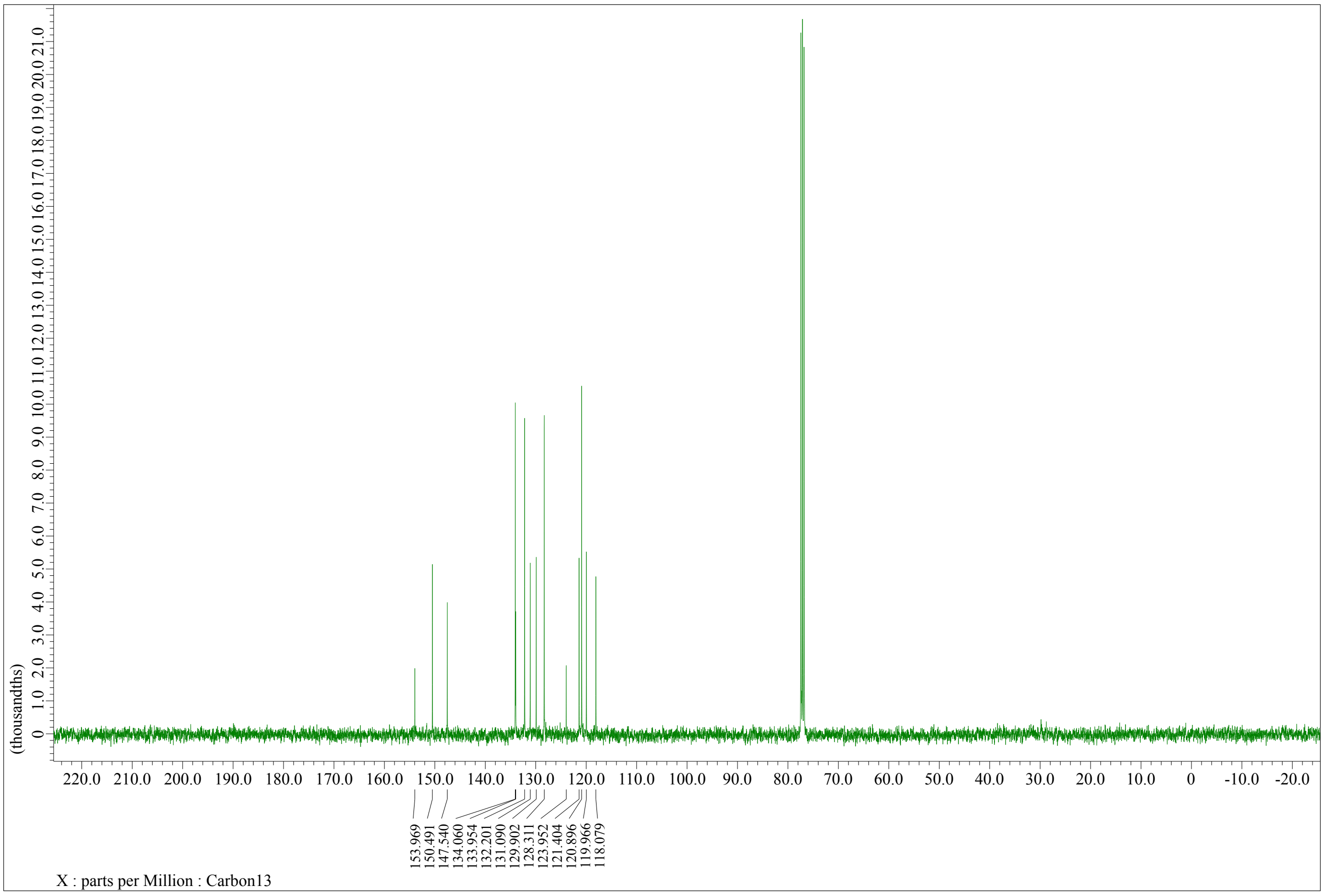

Figure S35. ^{1}H and ^{13}C NMR of **2aK**.

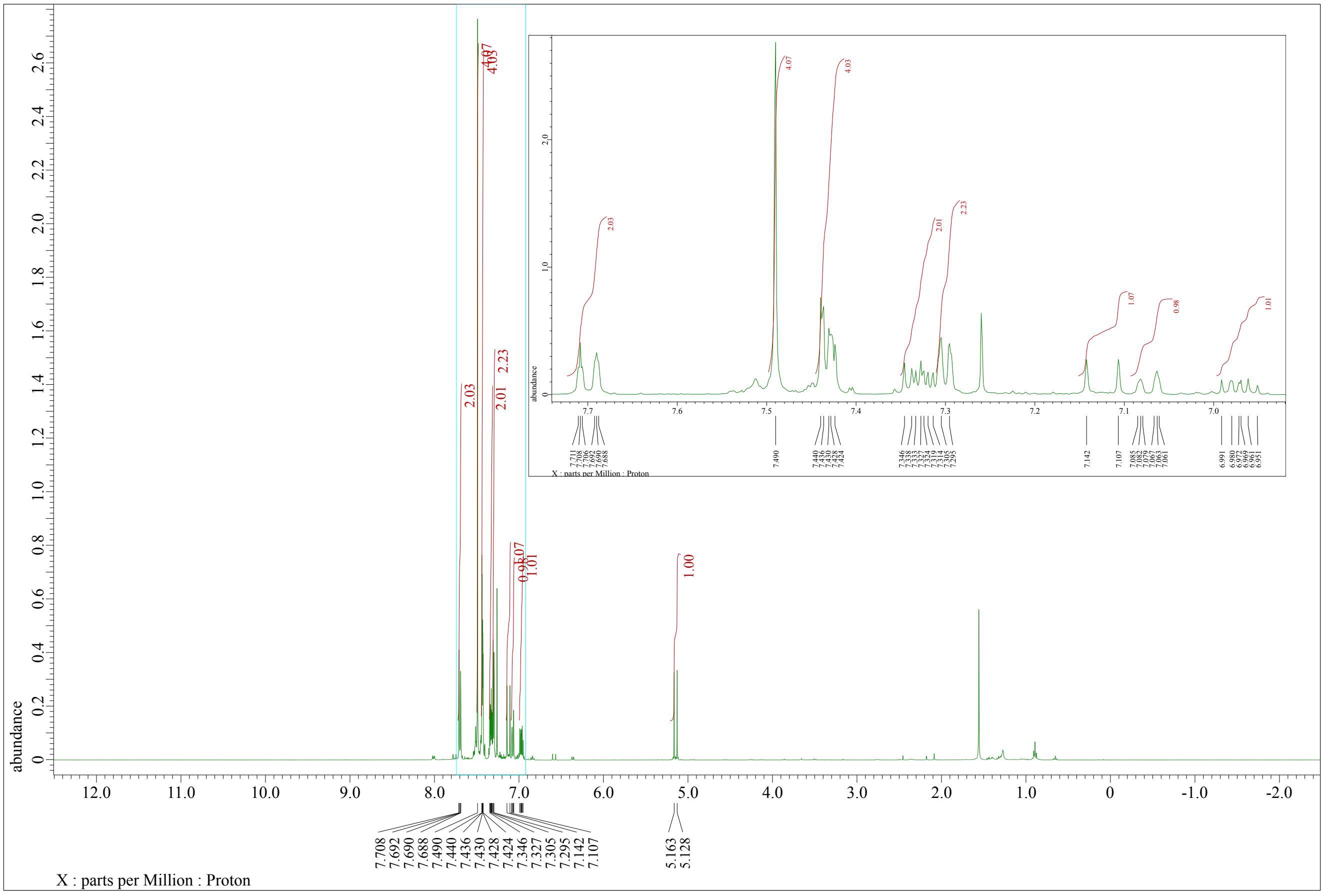

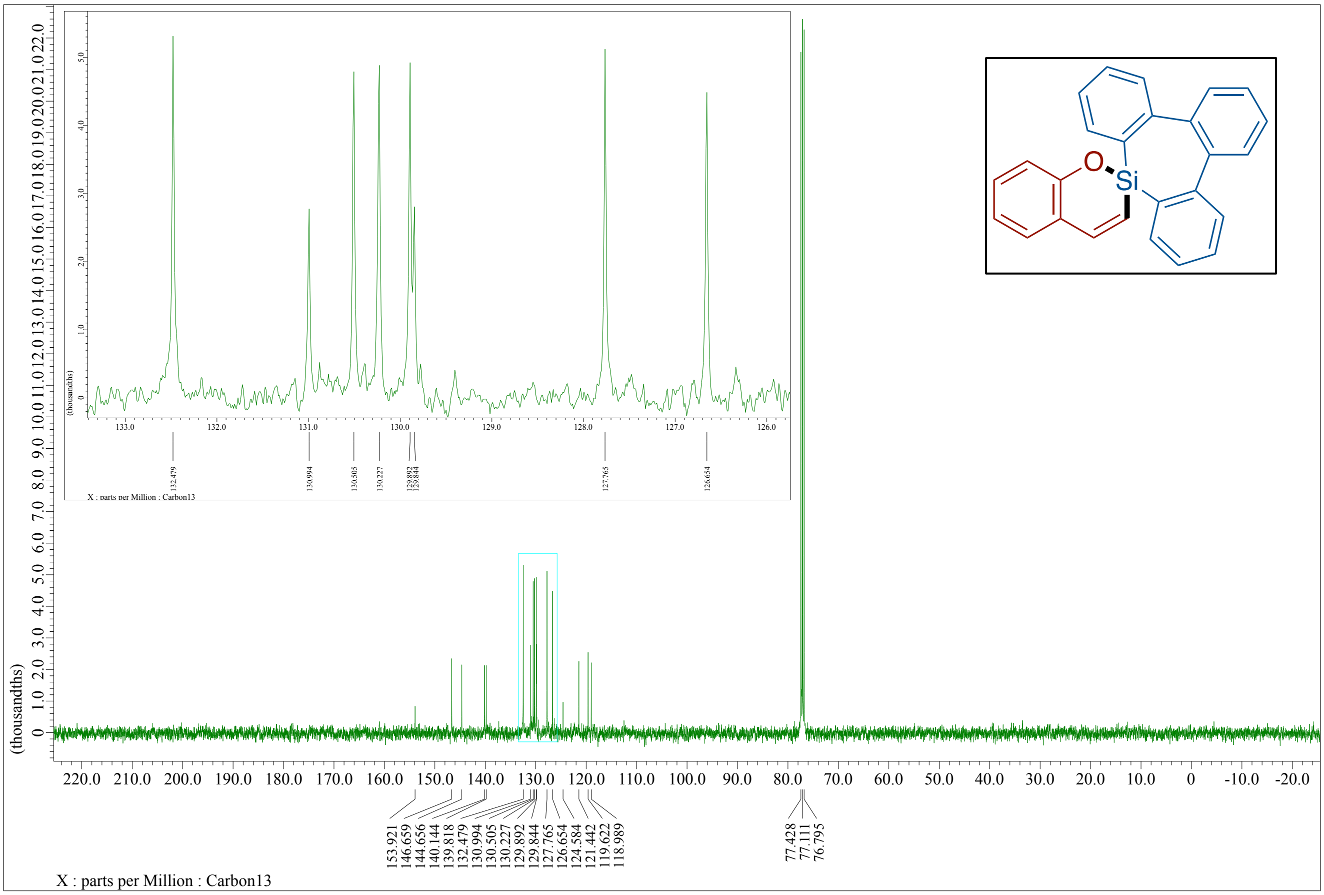

Figure S36. ^{1}H and ^{13}C NMR of **2aL**.

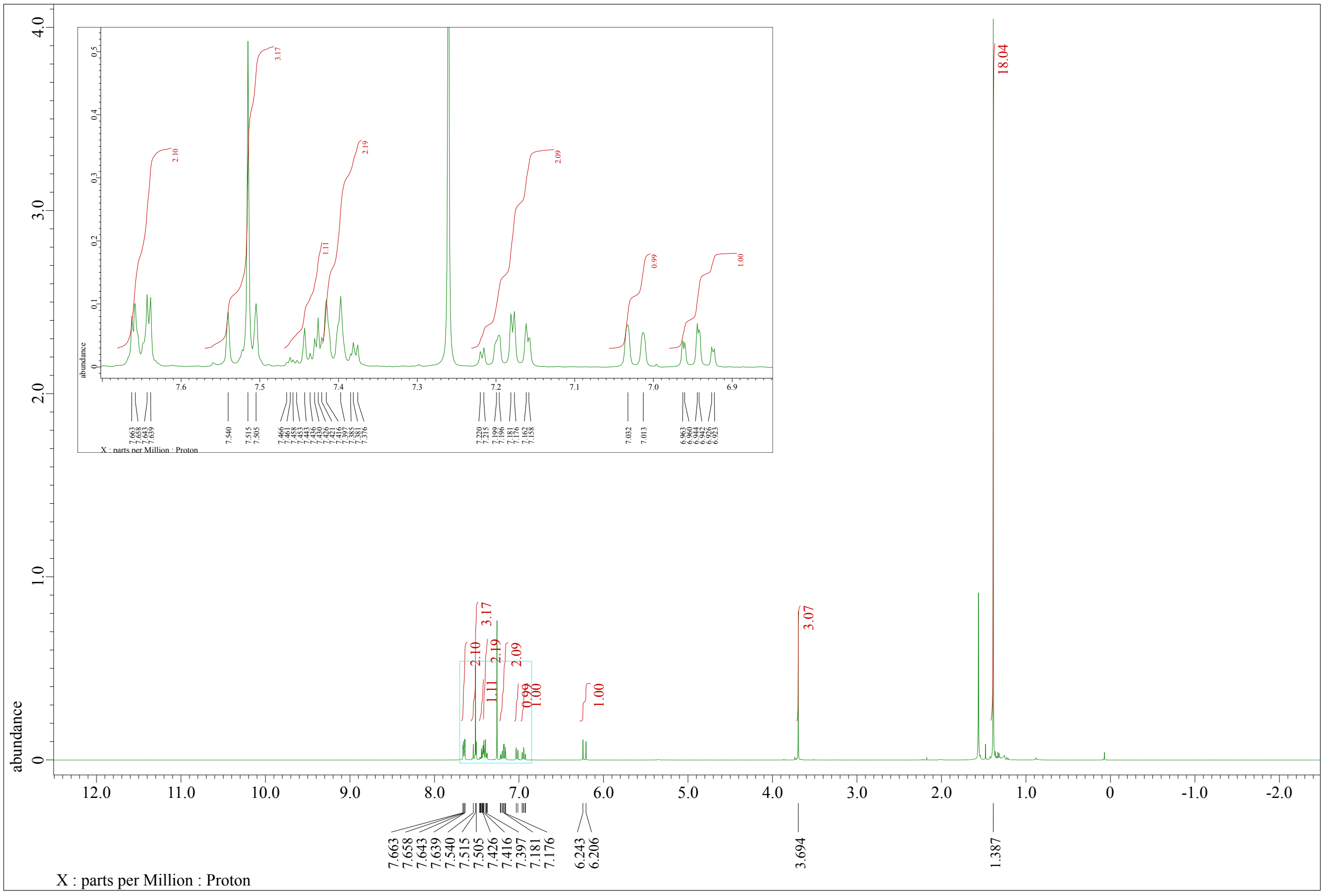

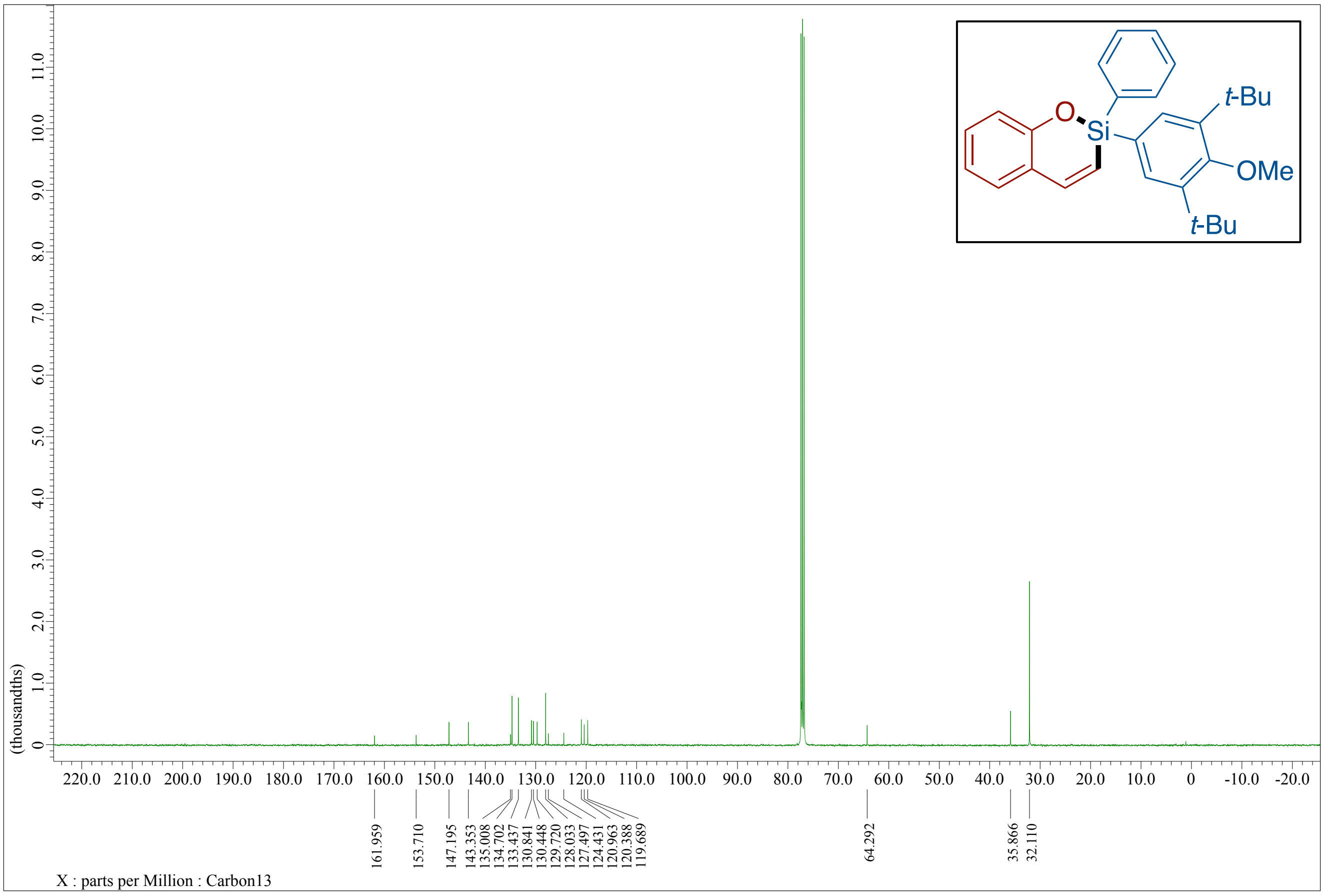

Figure S37. ^{1}H and ^{13}C NMR of **2aM**.

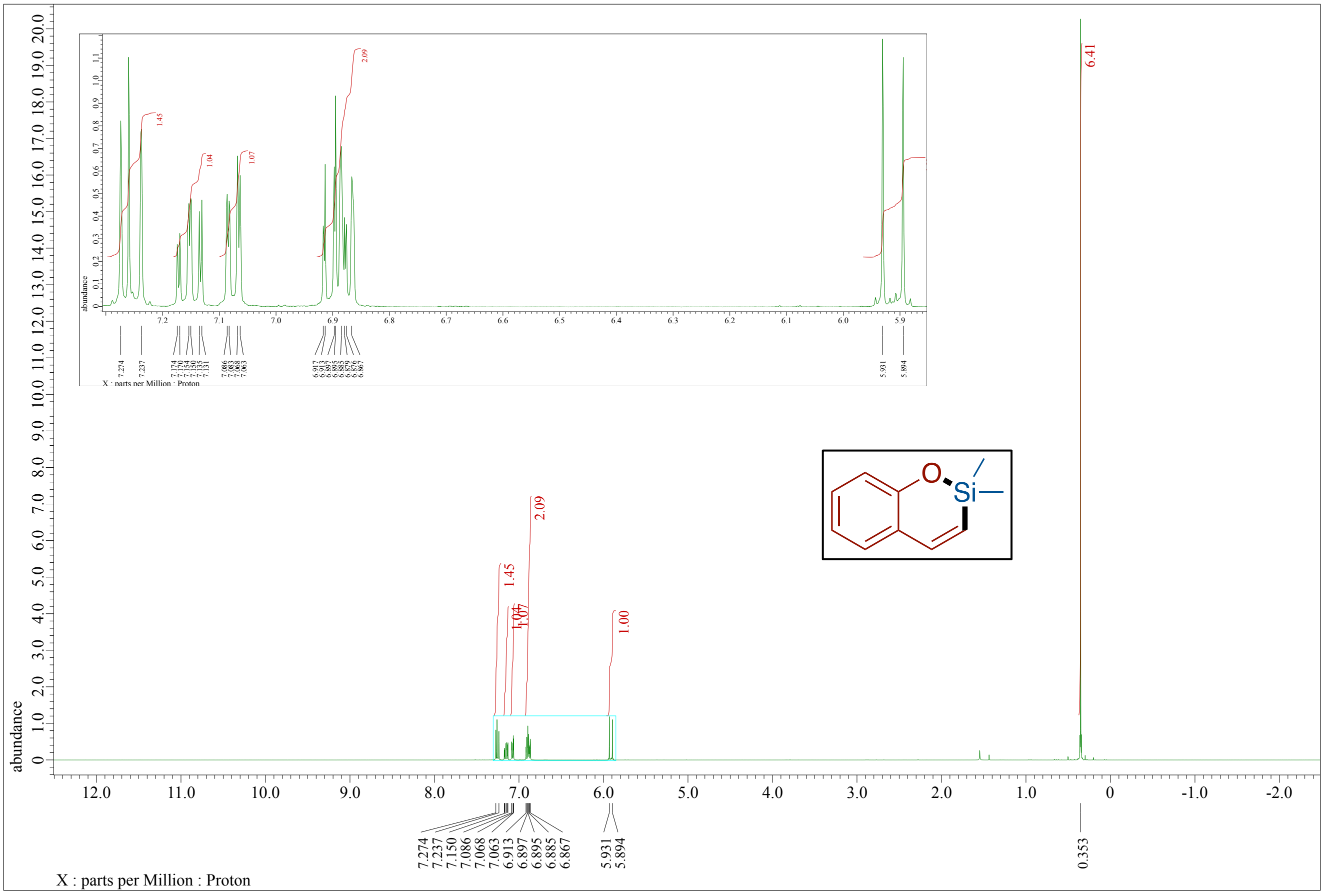

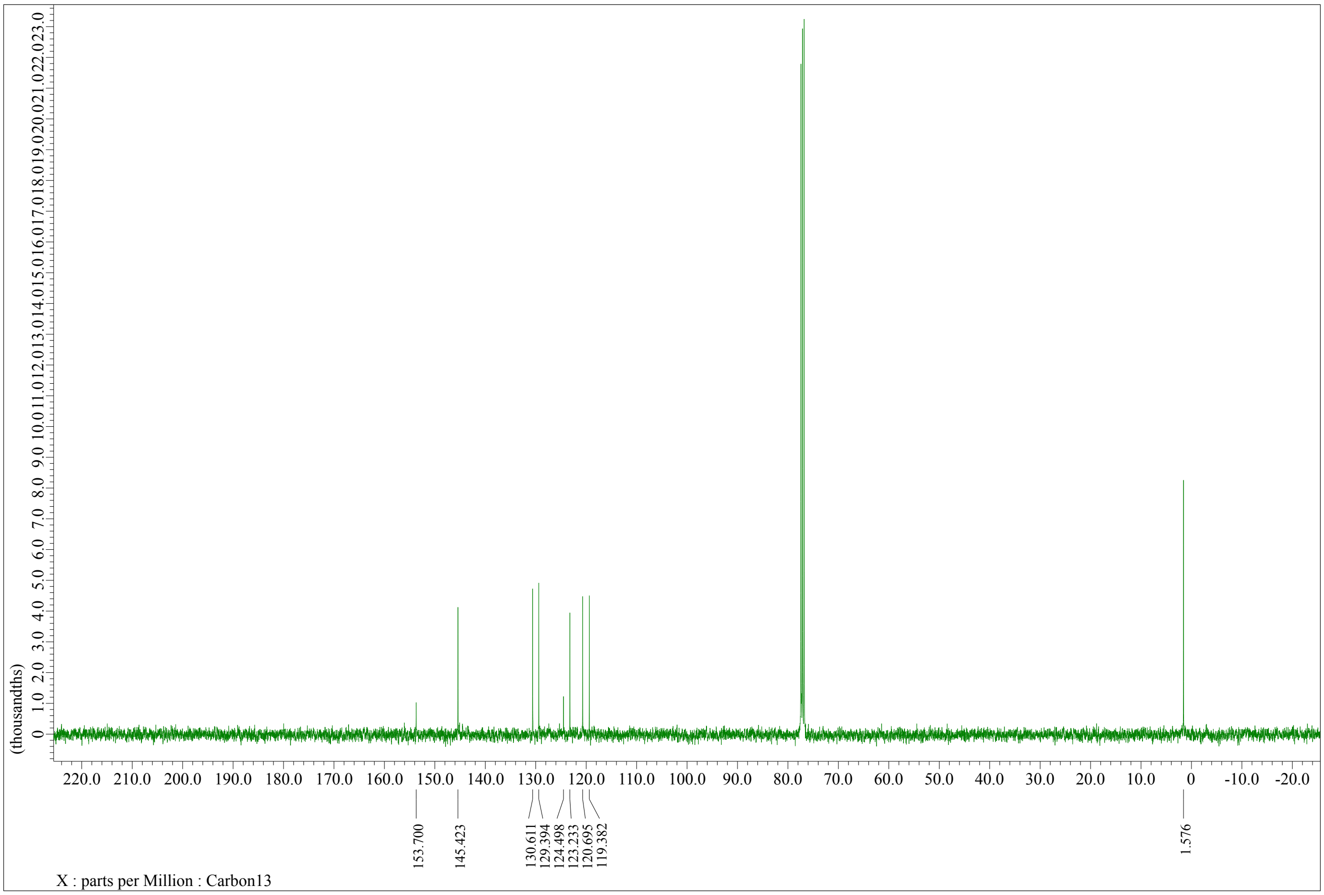

Figure S38. ^{1}H and ^{13}C NMR of **2aA**.

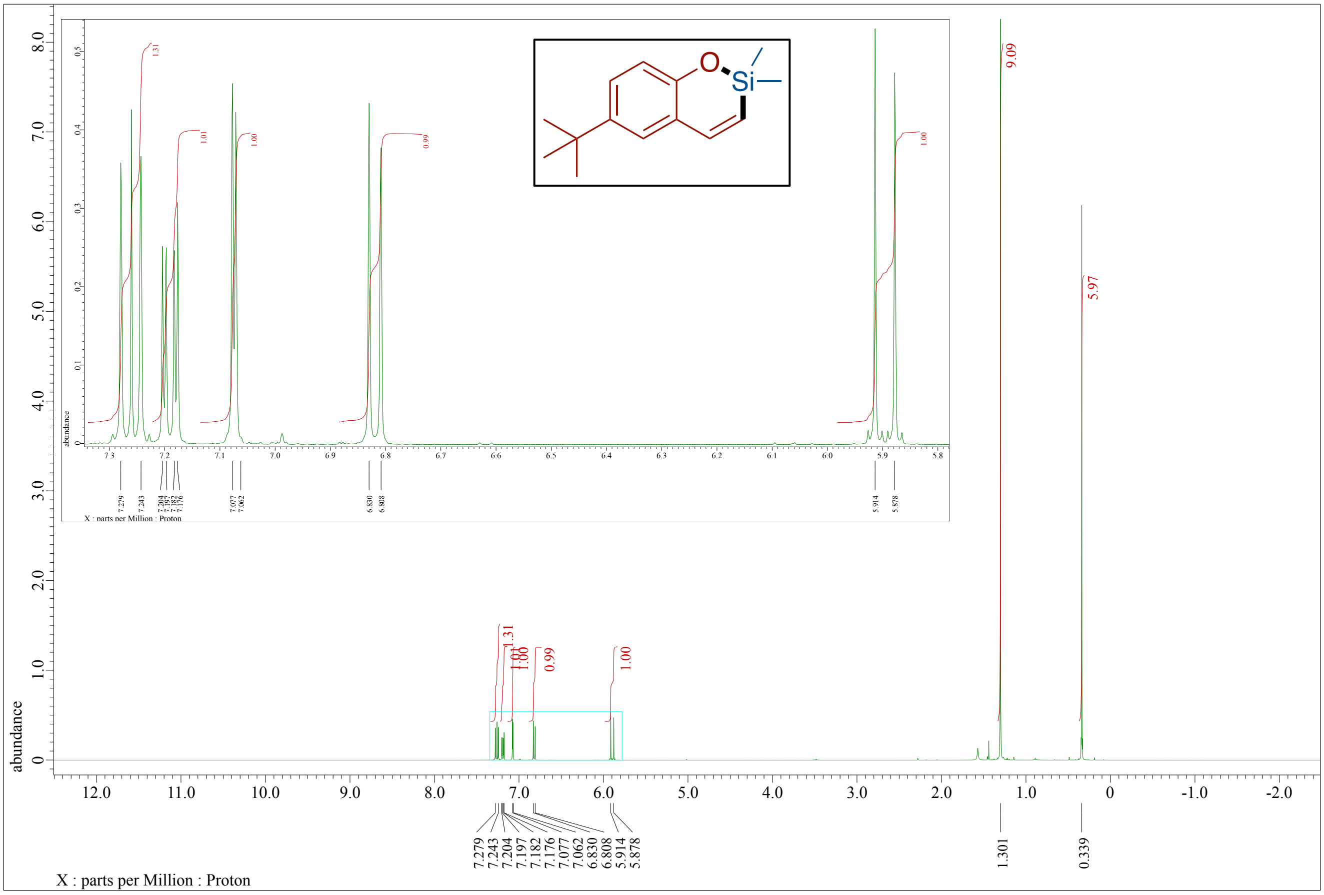

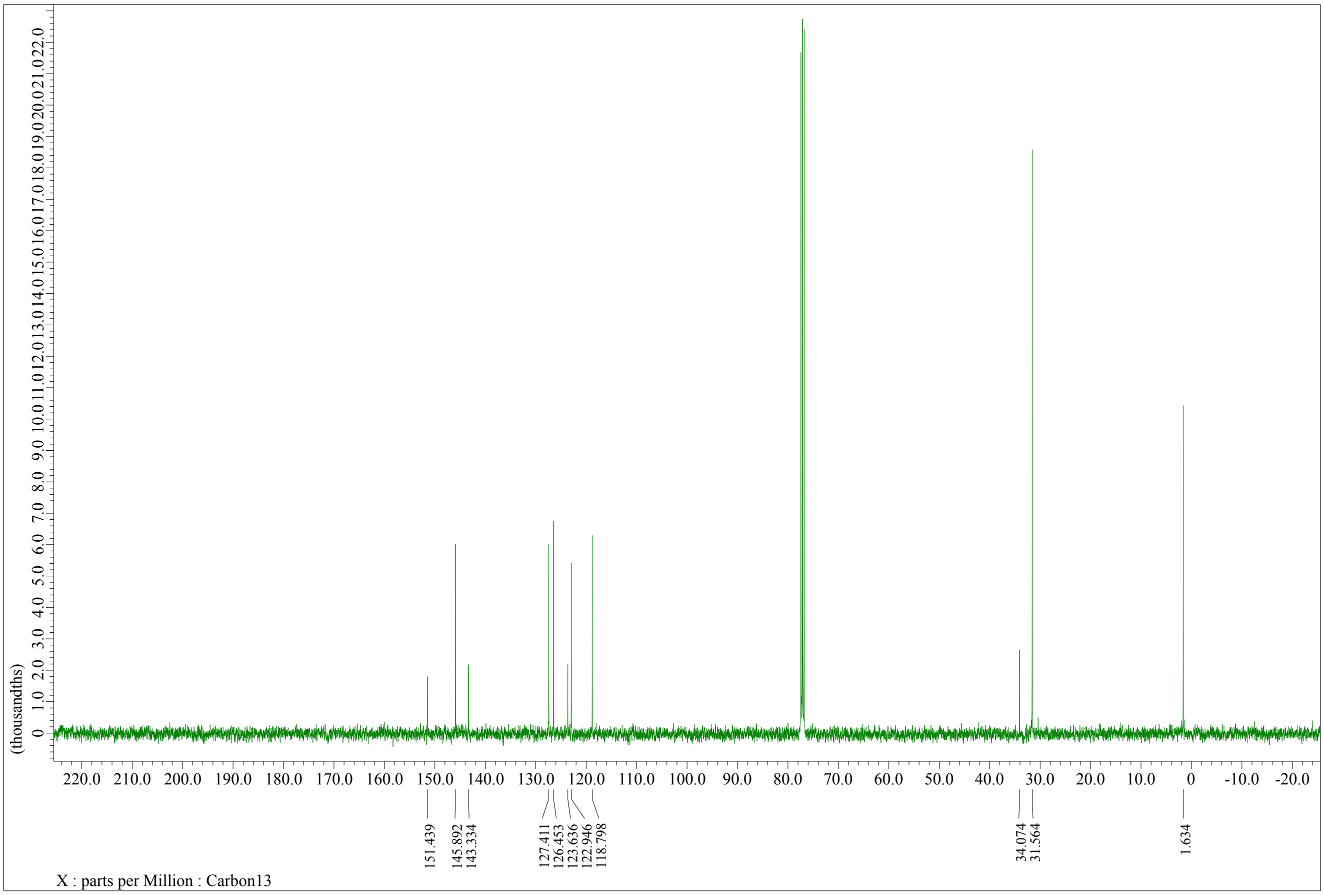

Figure S39. ^{1}H and ^{13}C NMR of **2bA**.

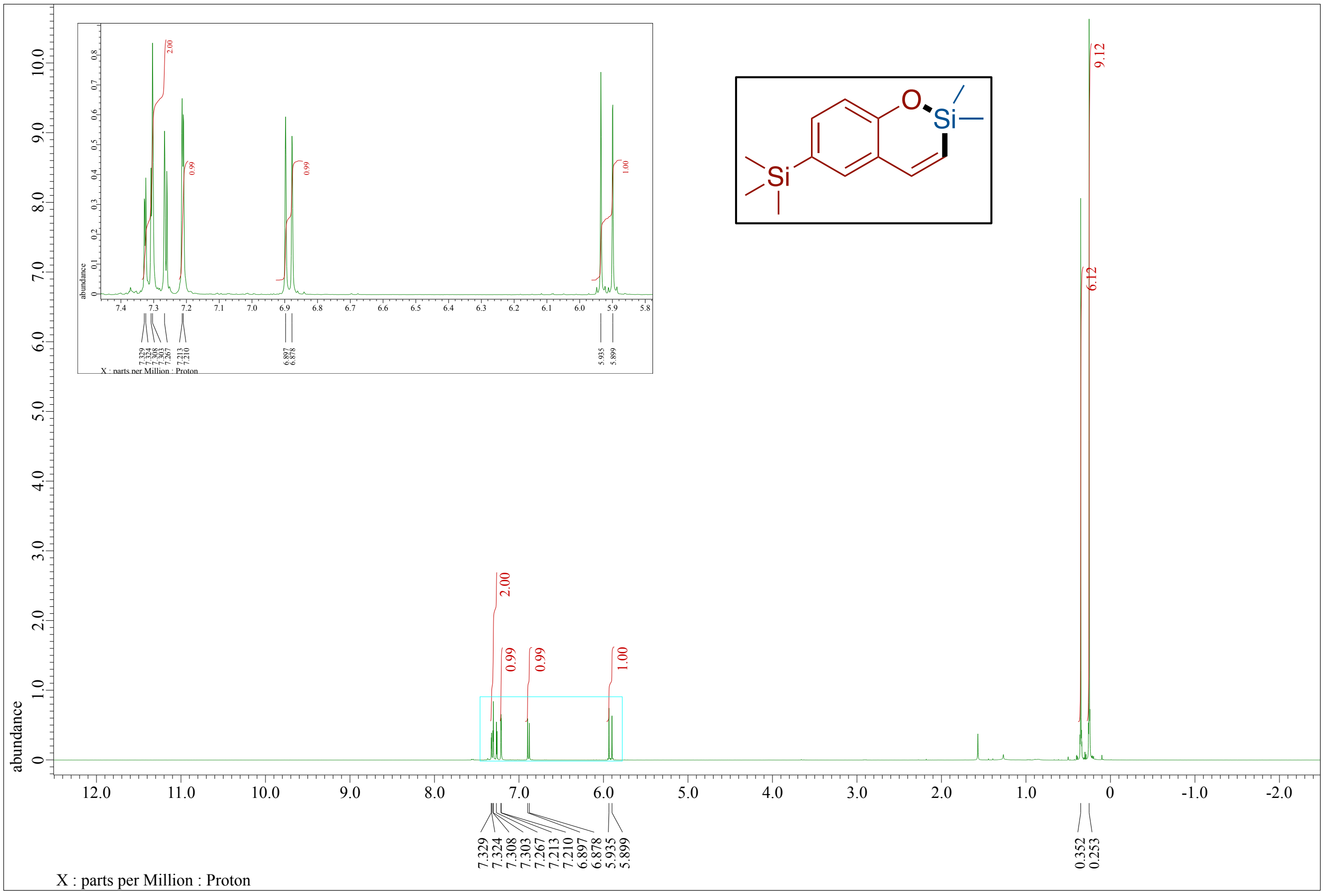

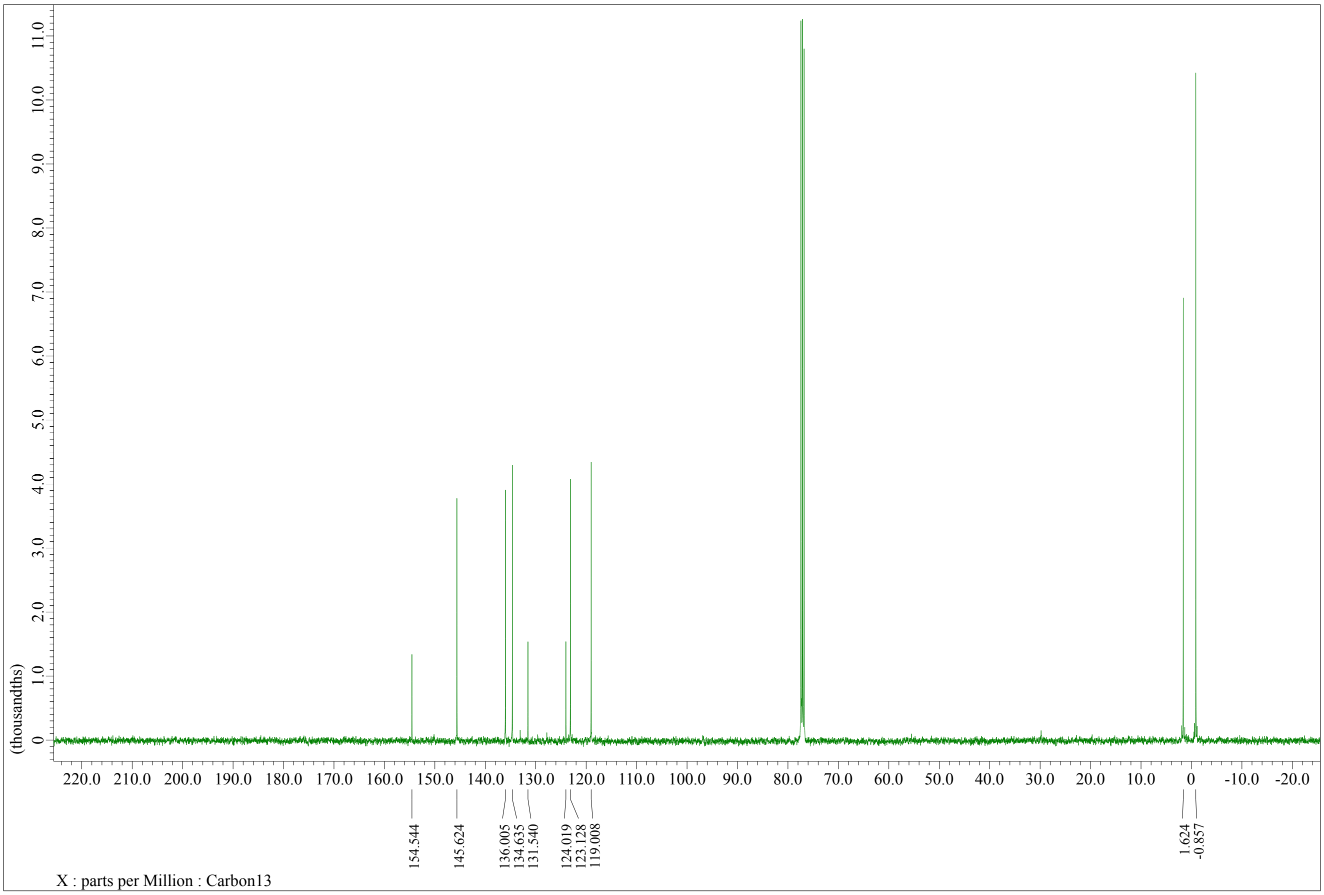

Figure S40. ^{1}H and ^{13}C NMR of **2cA**.

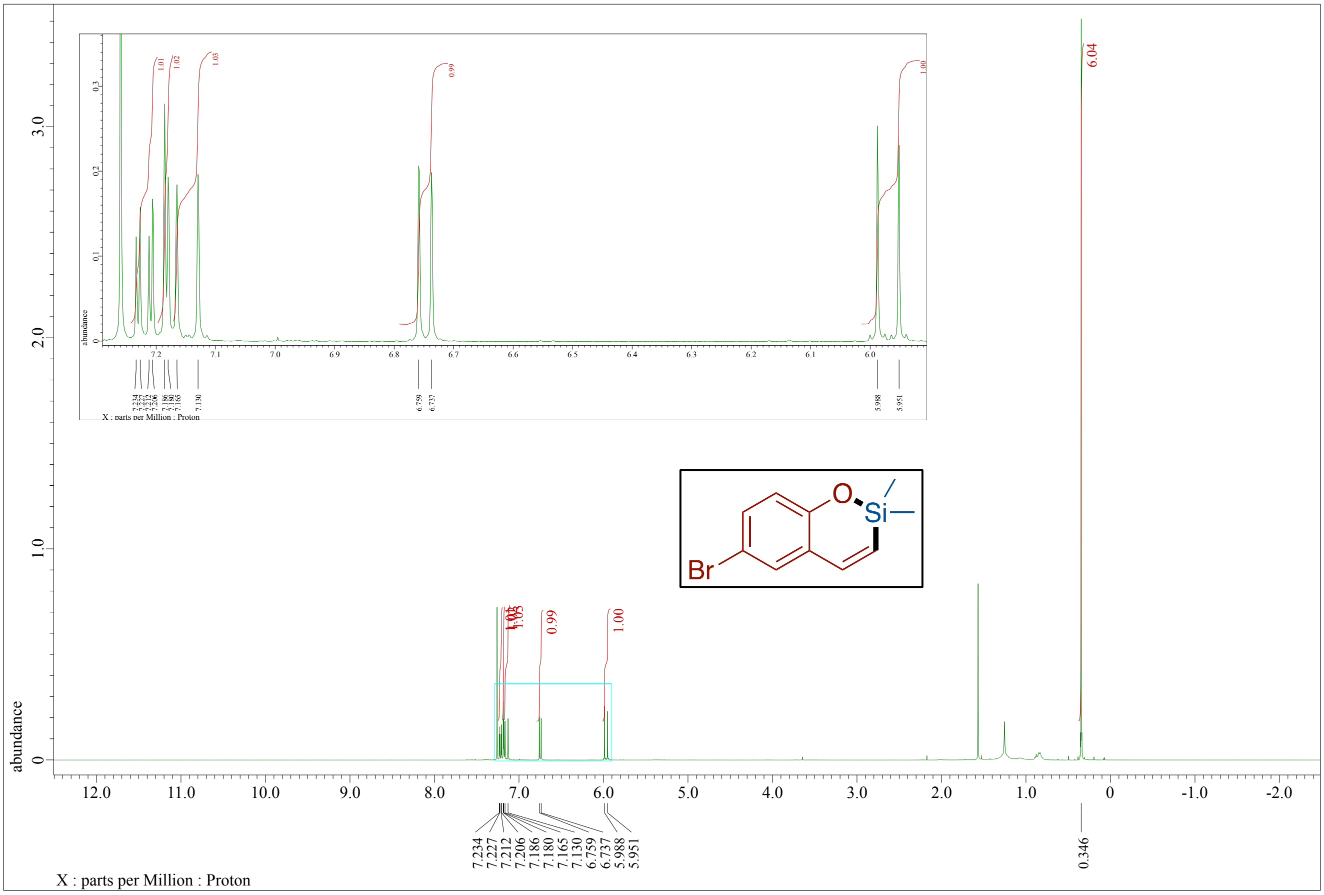

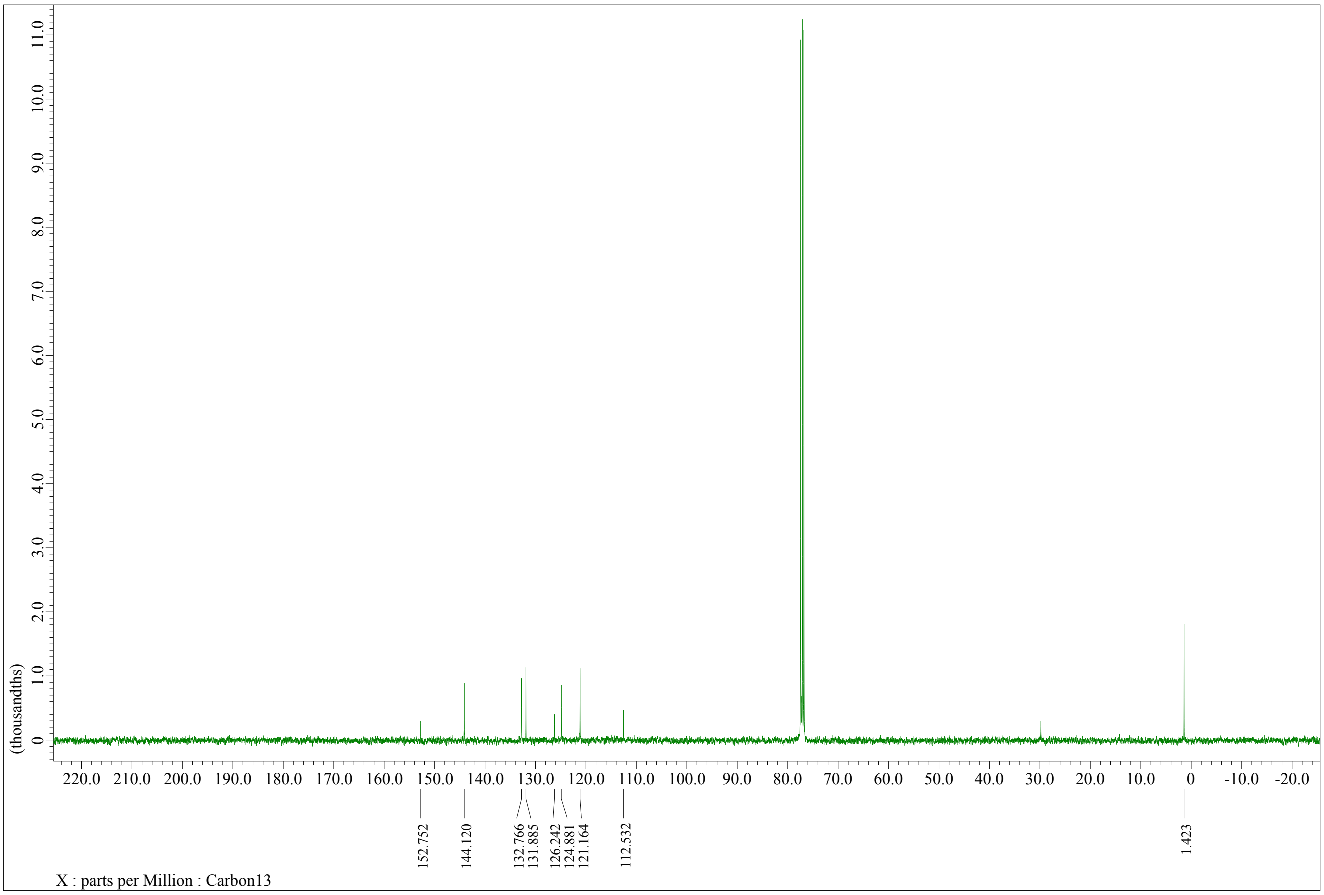

Figure S41. ^{1}H and ^{13}C NMR of **2dA**.

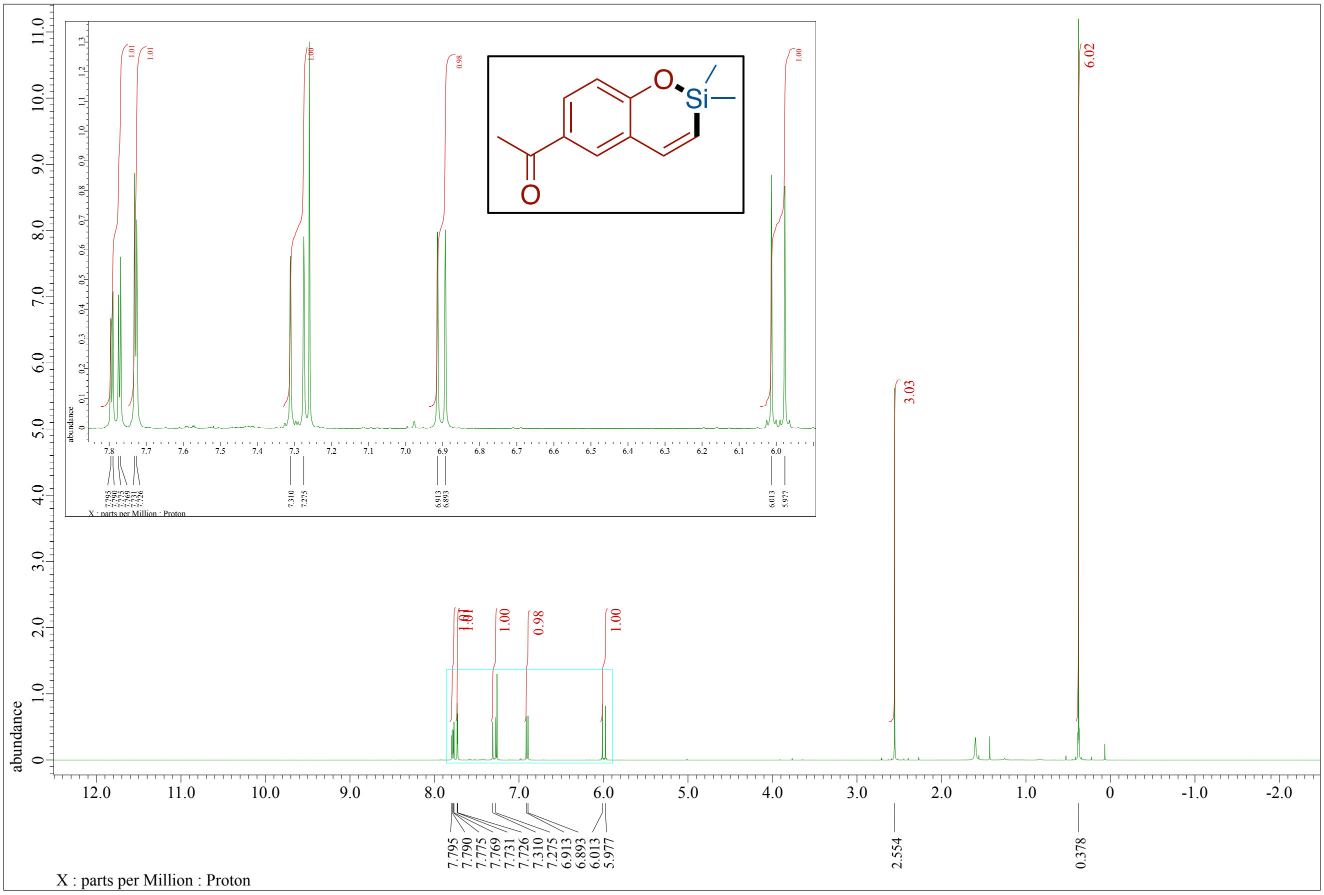

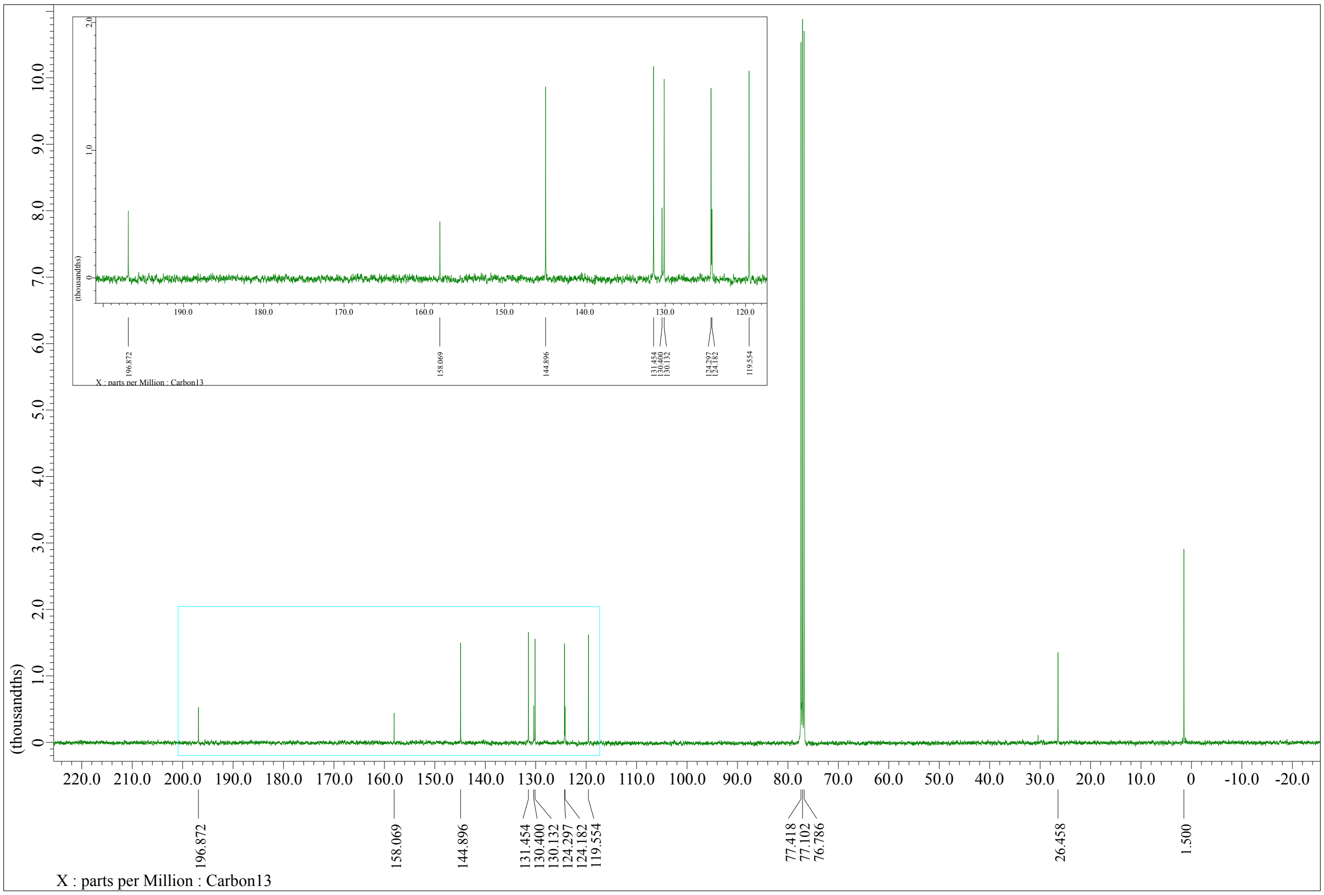

Figure S42. ^{1}H and ^{13}C NMR of **2eA**.

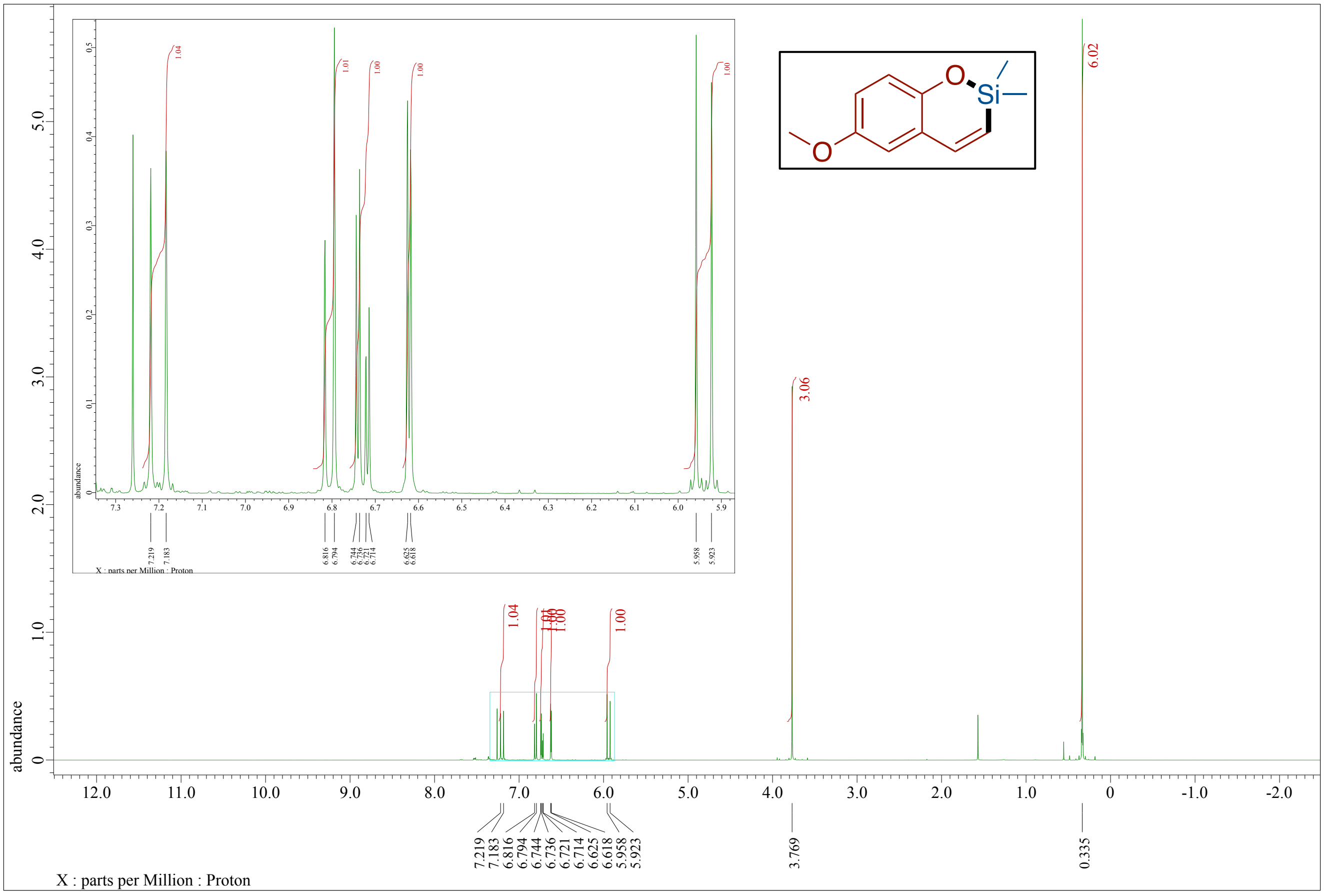

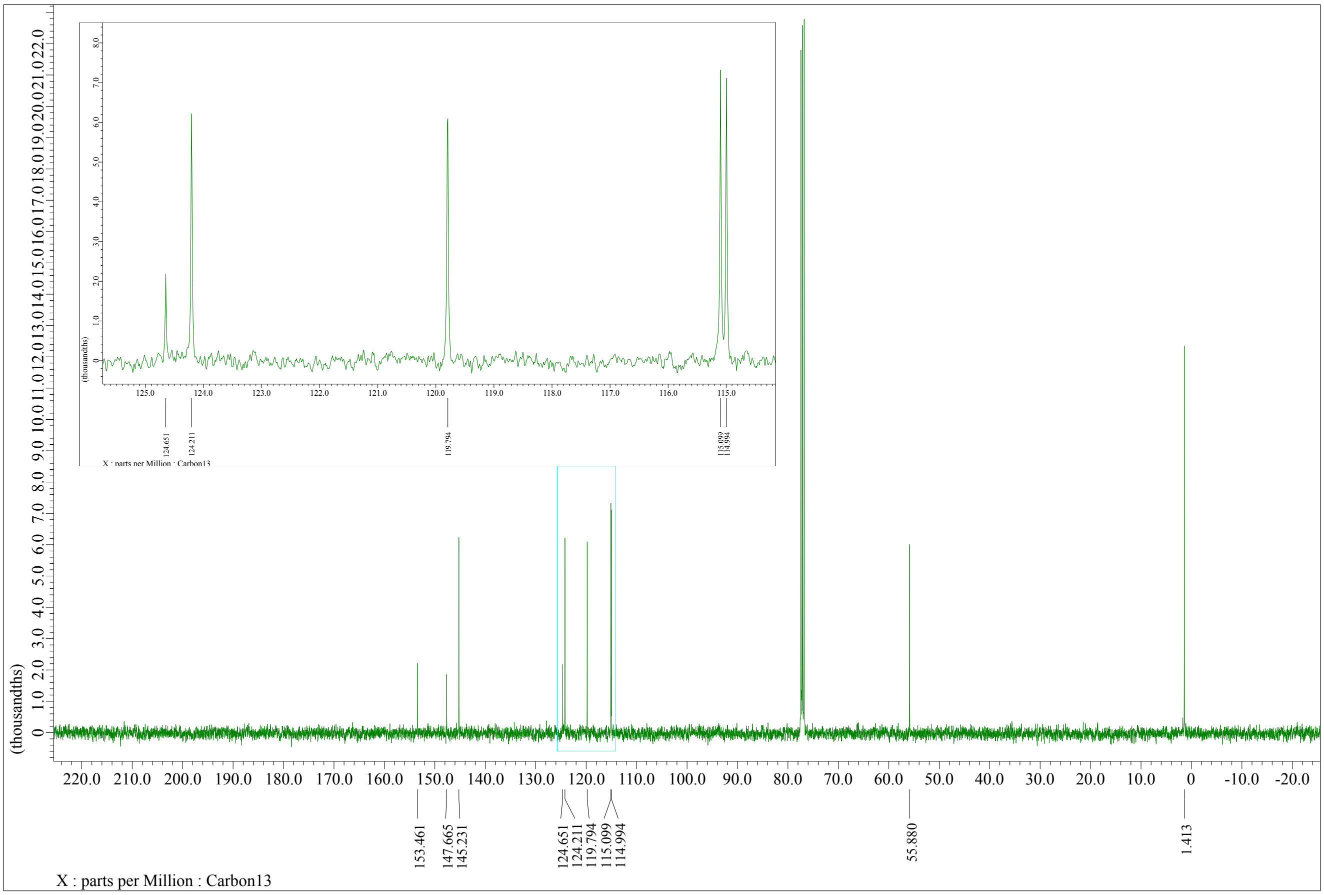

Figure S43. ^{1}H and ^{13}C NMR of **2fA**.

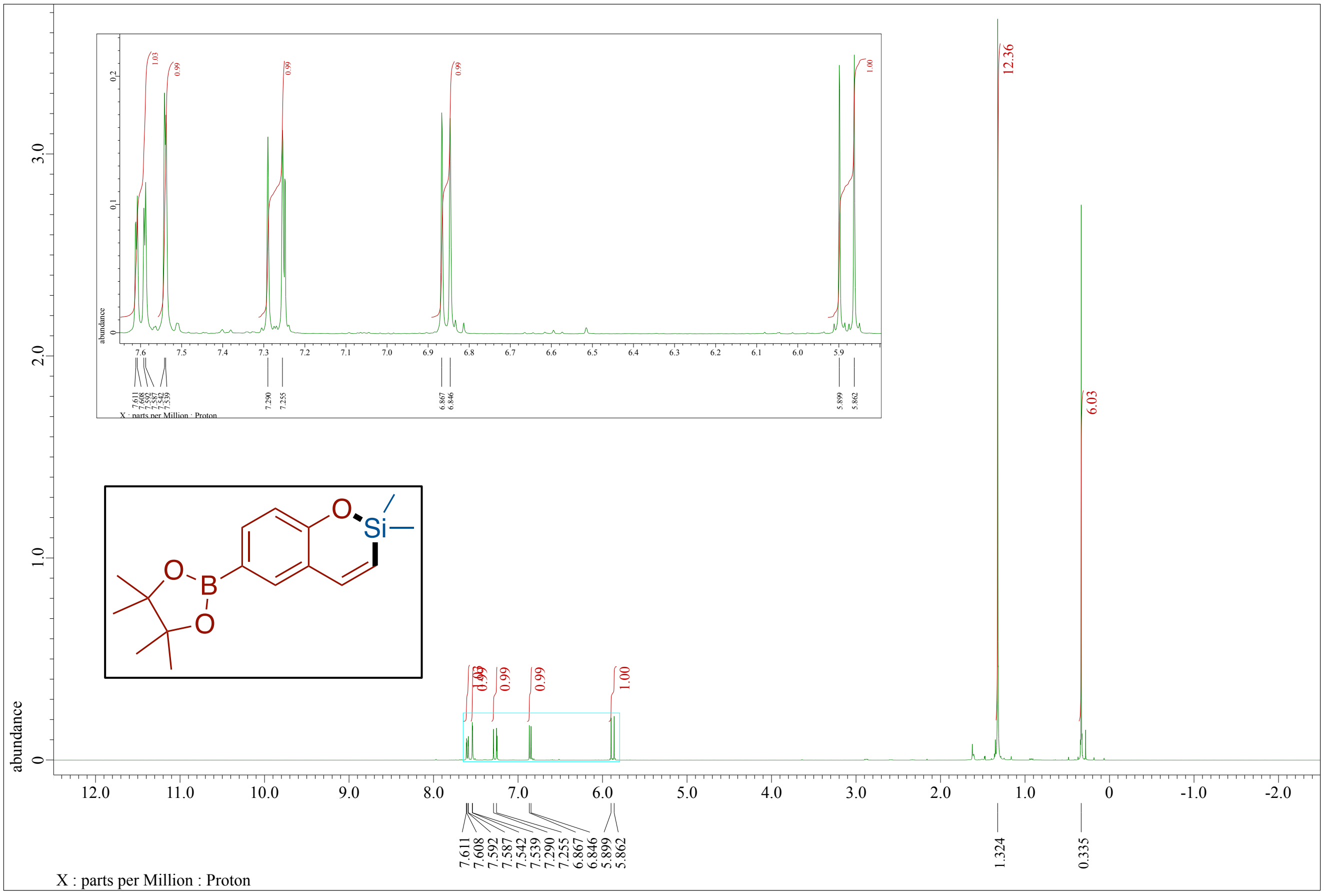

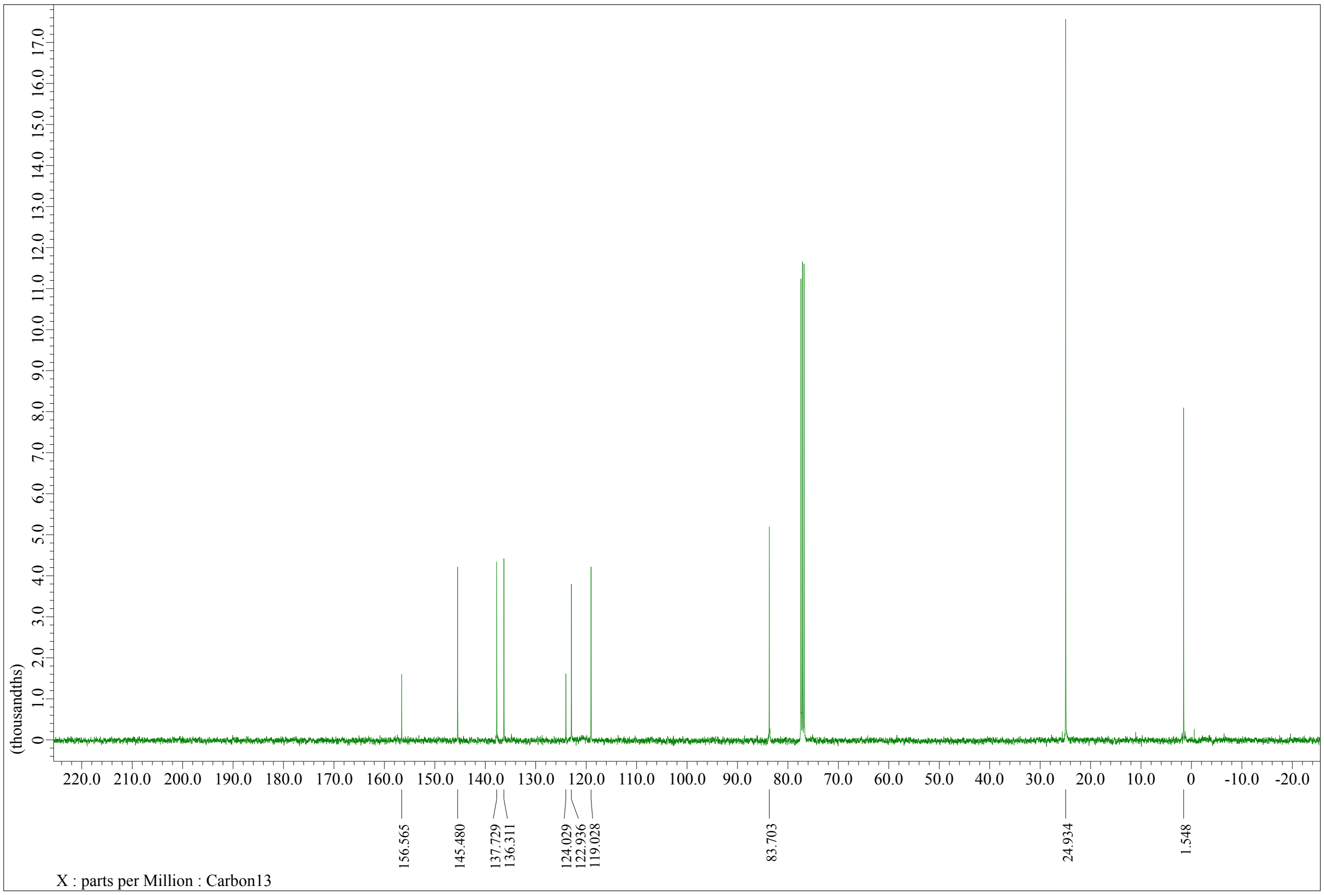

Figure S44. ^{1}H and ^{13}C NMR of **2gA**.

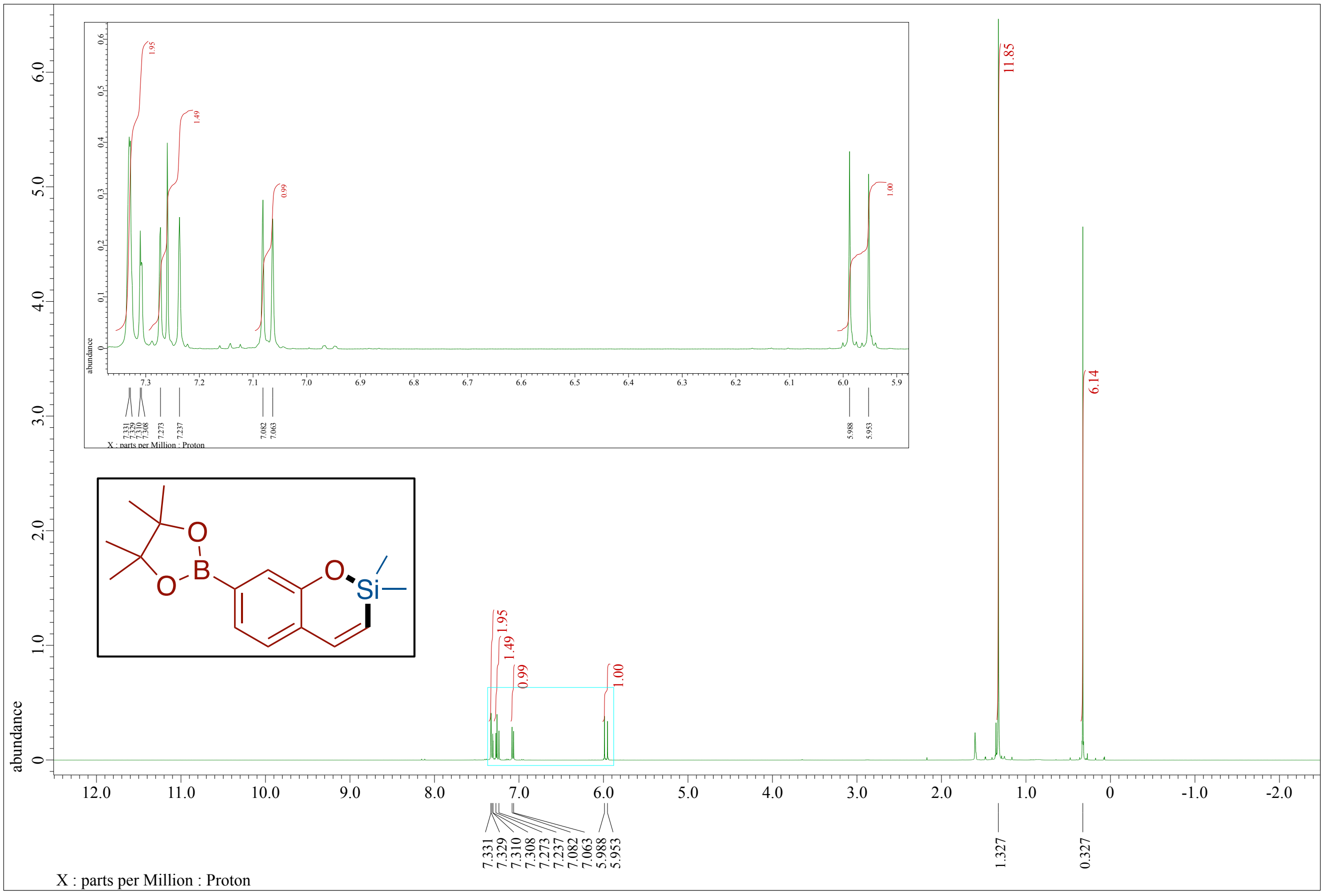

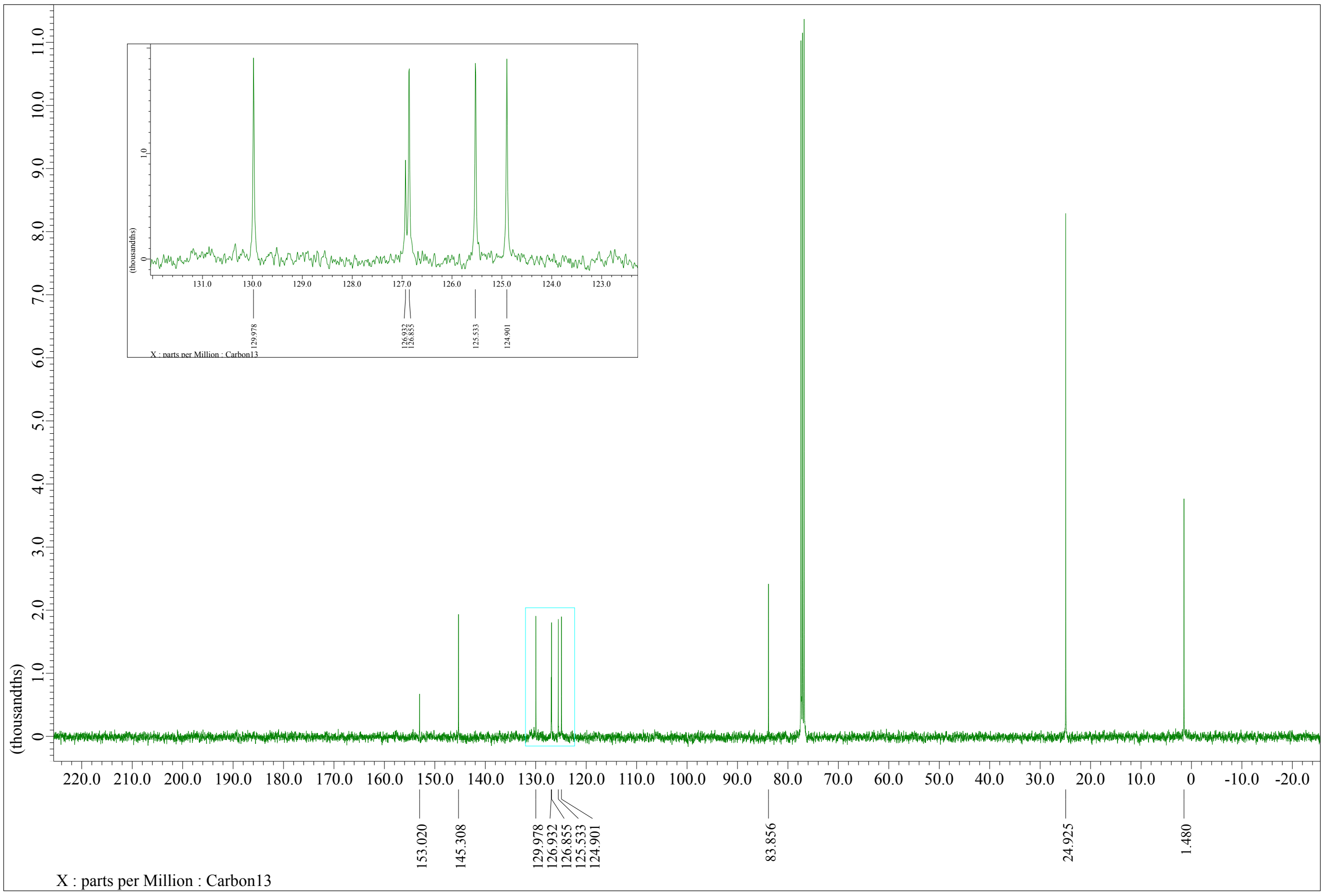

Figure S45. ^{1}H and ^{13}C NMR of **2hA**.

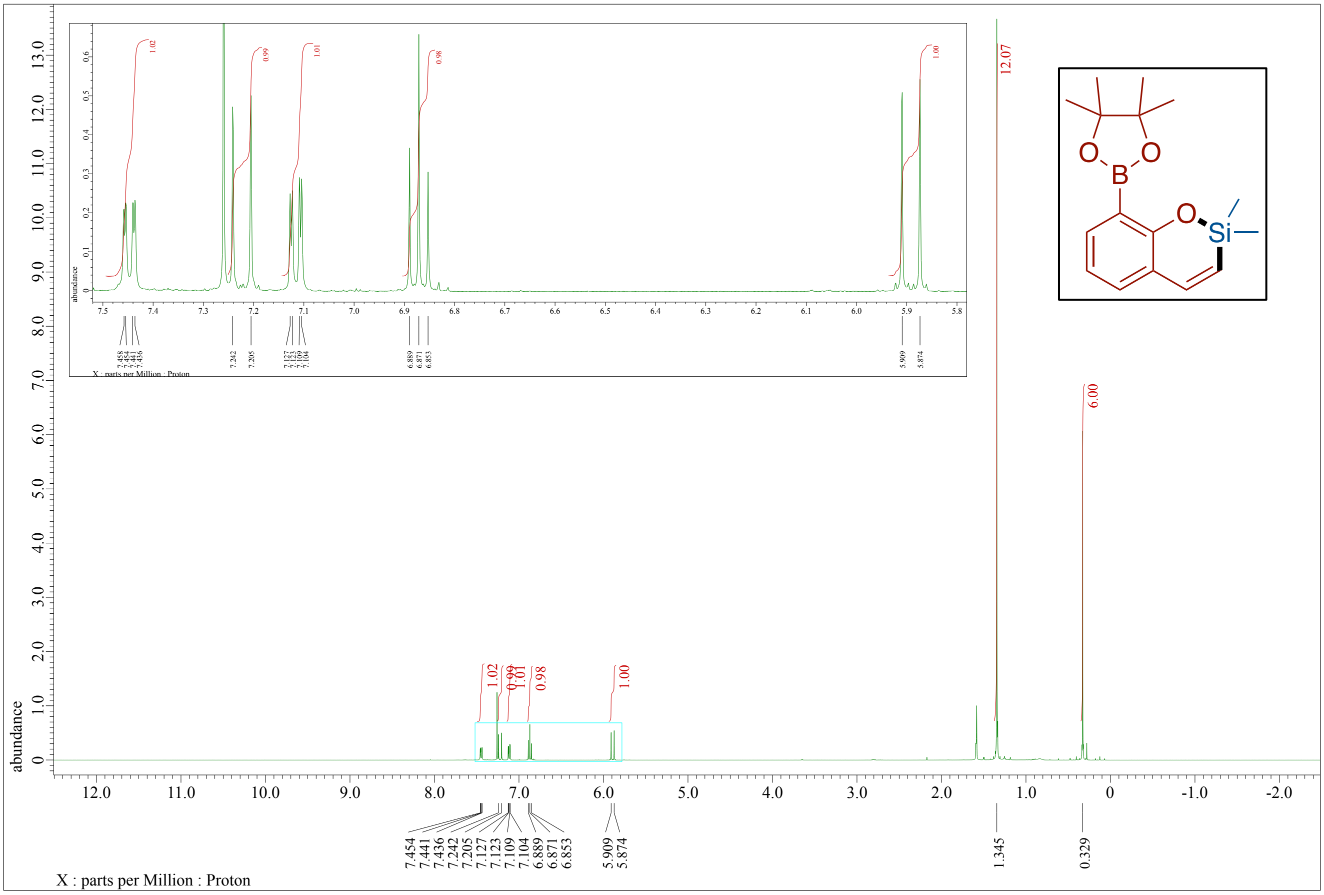

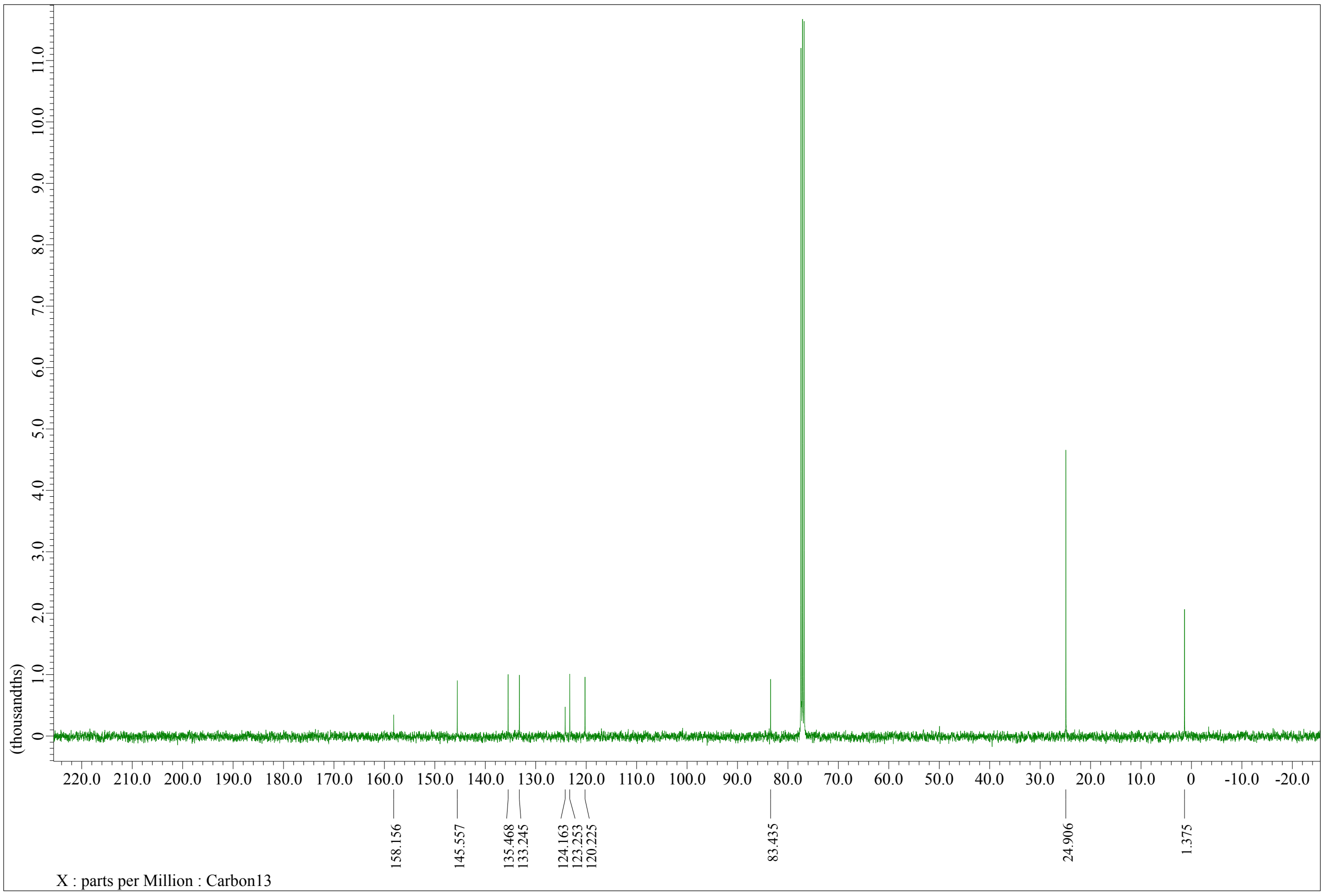

Figure S46. ^{1}H and ^{13}C NMR of **2iA**.

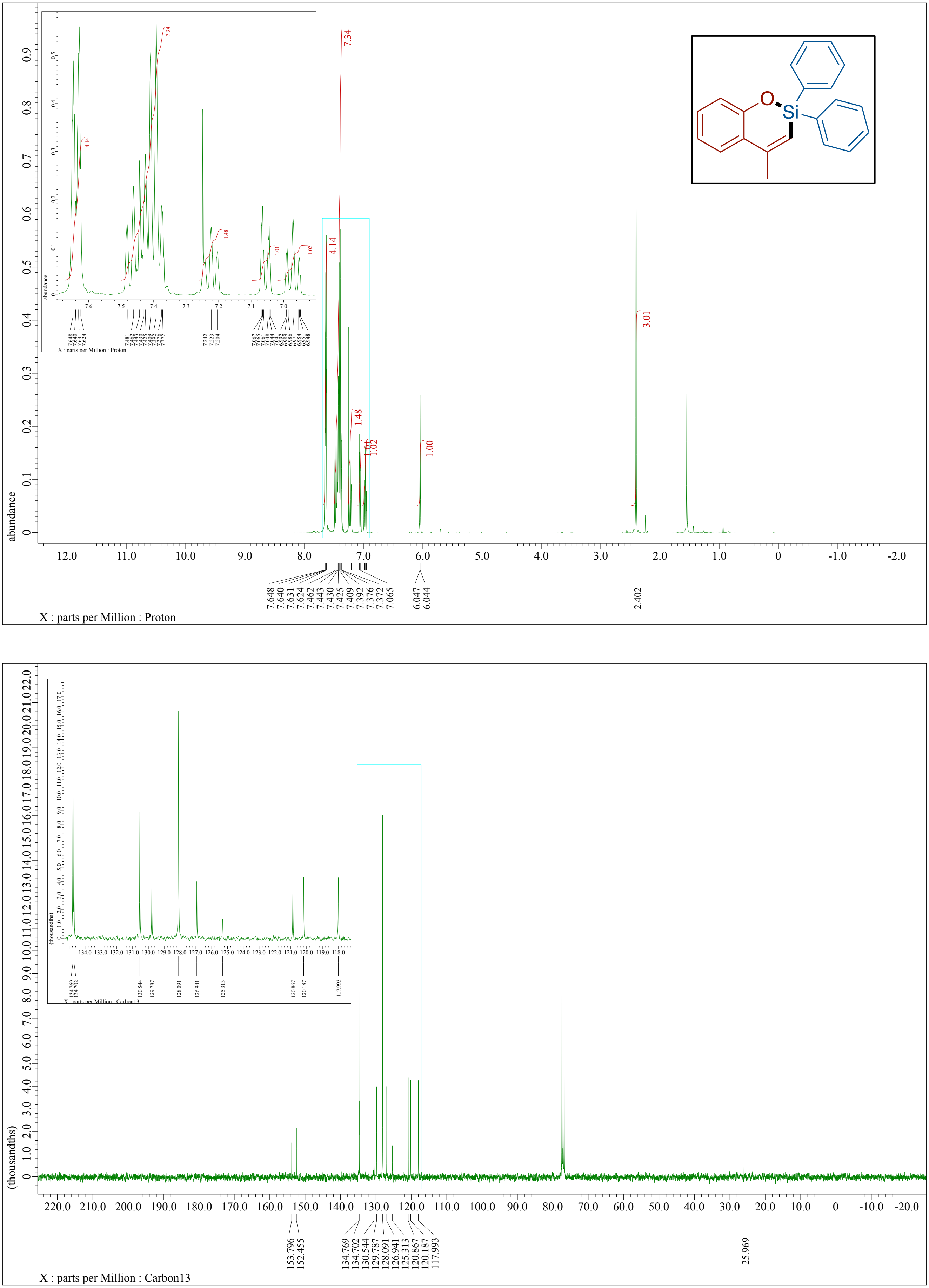

Figure S47. ^{1}H and ^{13}C NMR of **2jJ**.

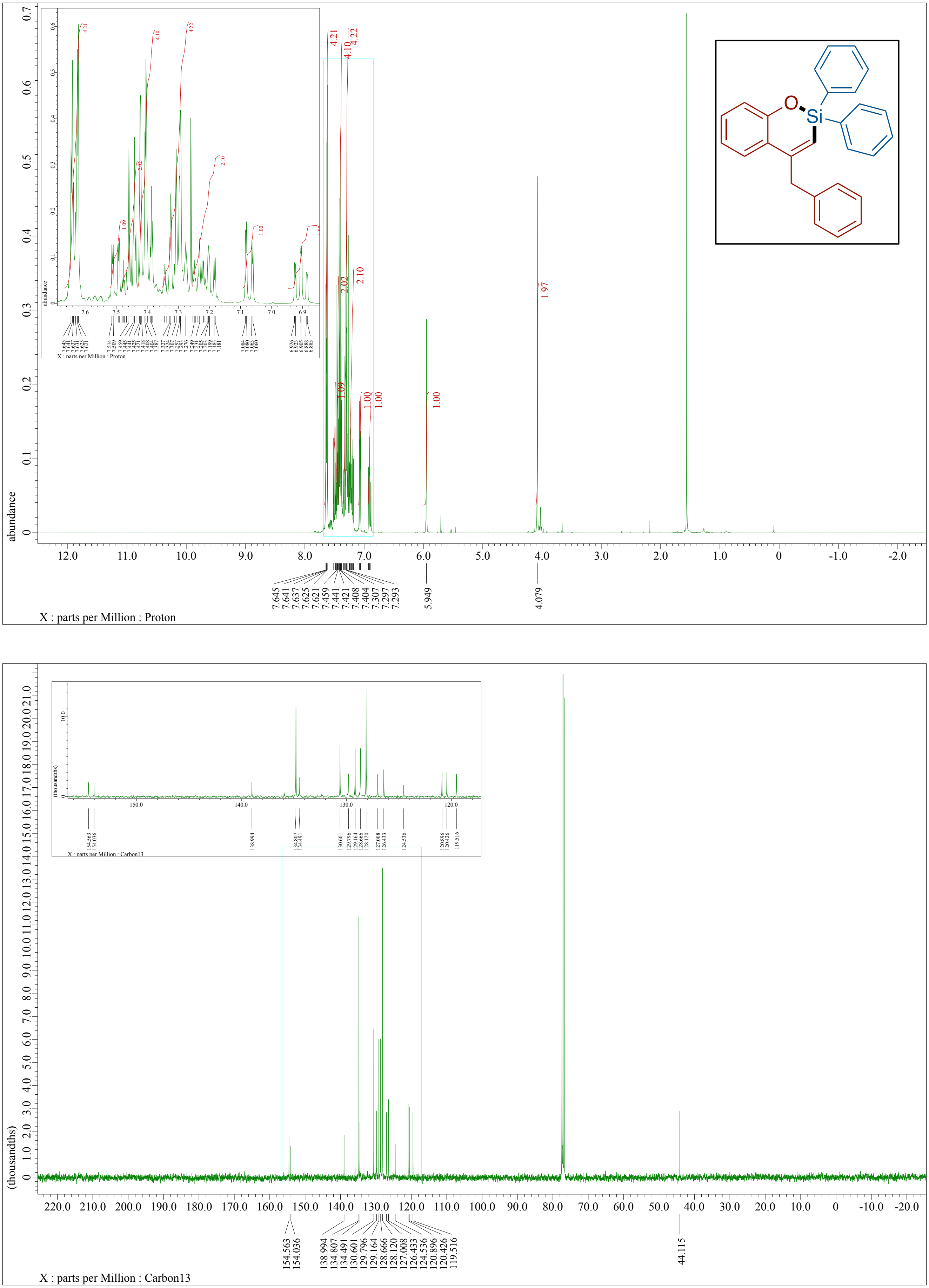

Figure S48. ^{1}H and ^{13}C NMR of **2kJ**.

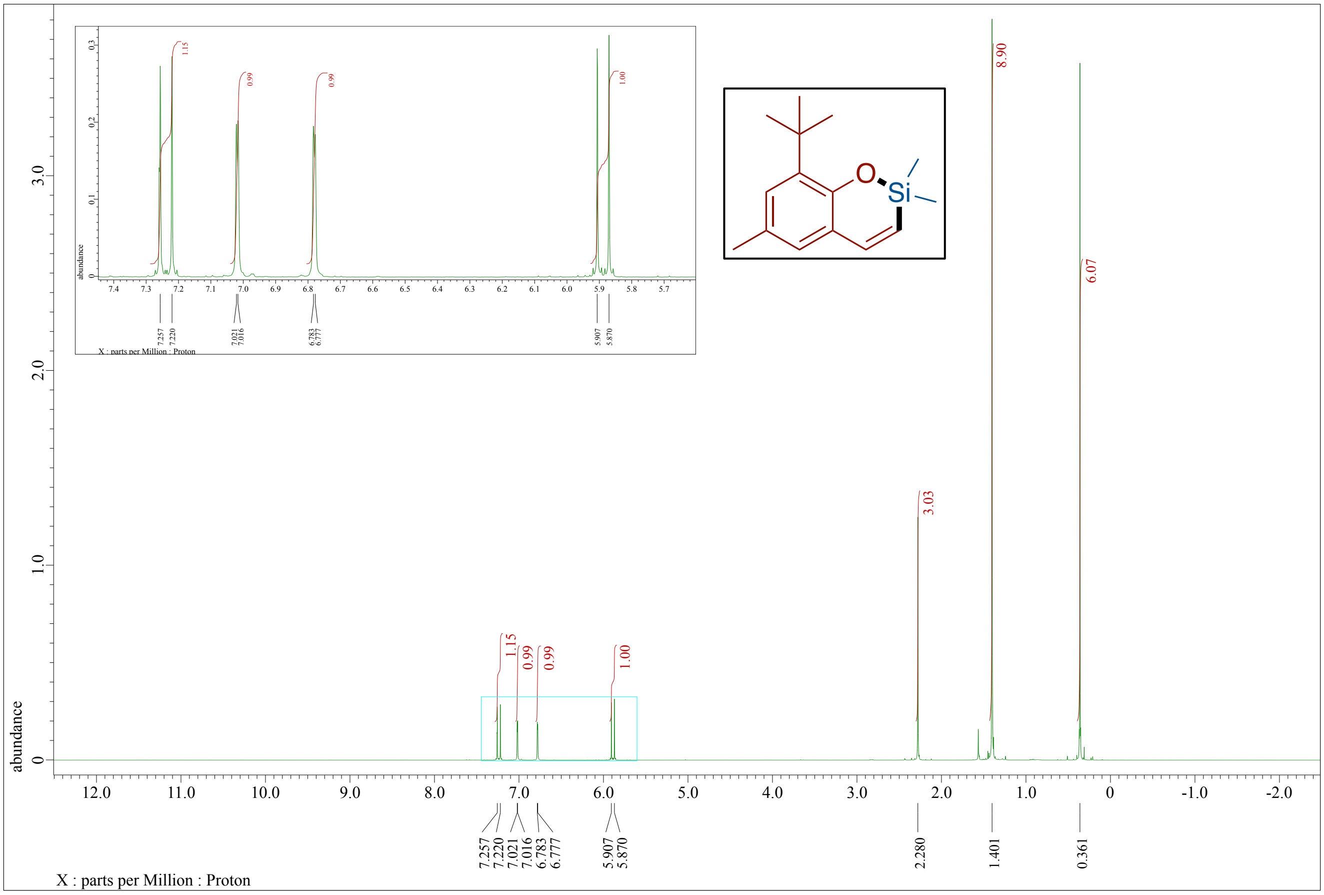

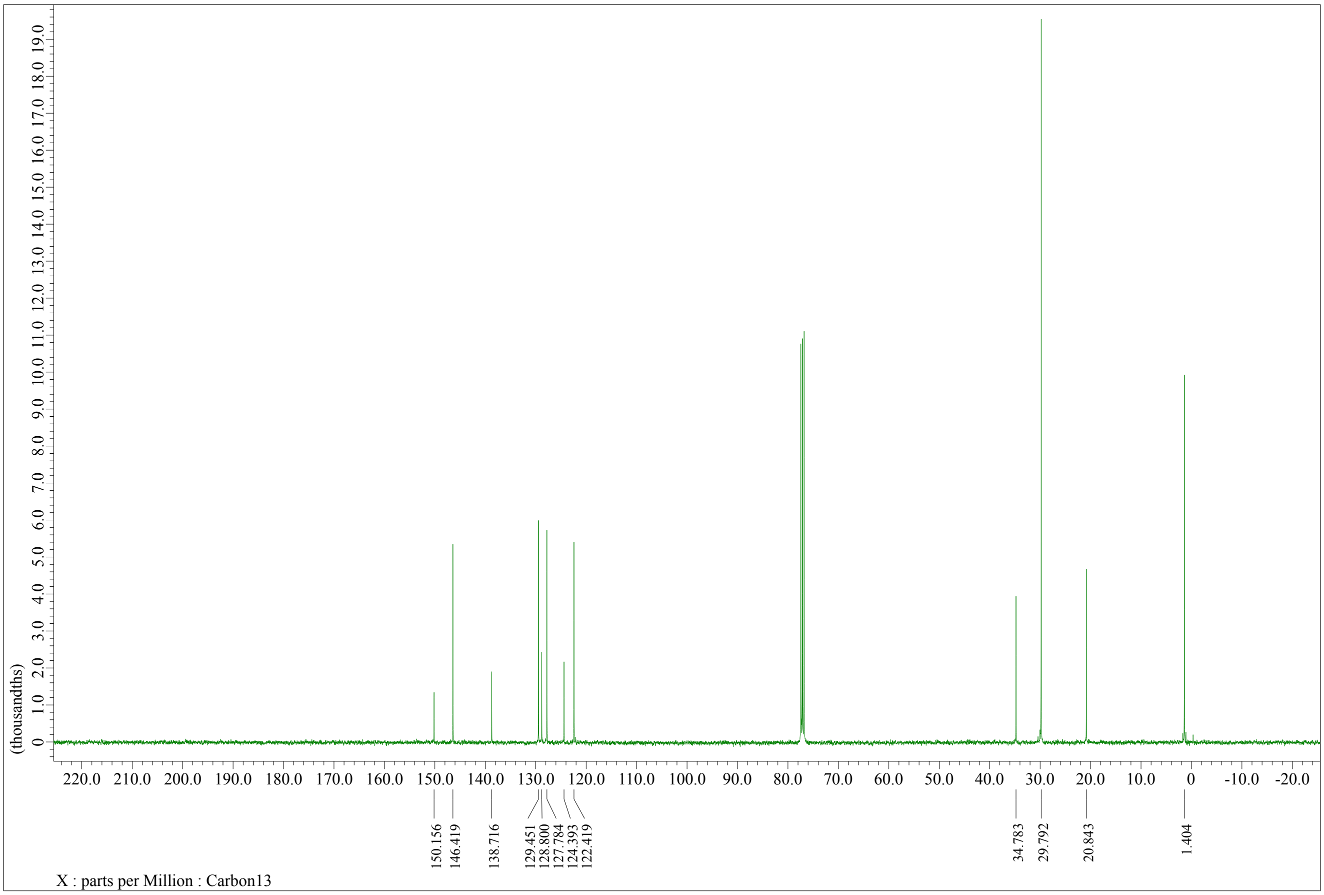

Figure S49. ^{1}H and ^{13}C NMR of **2lA**.

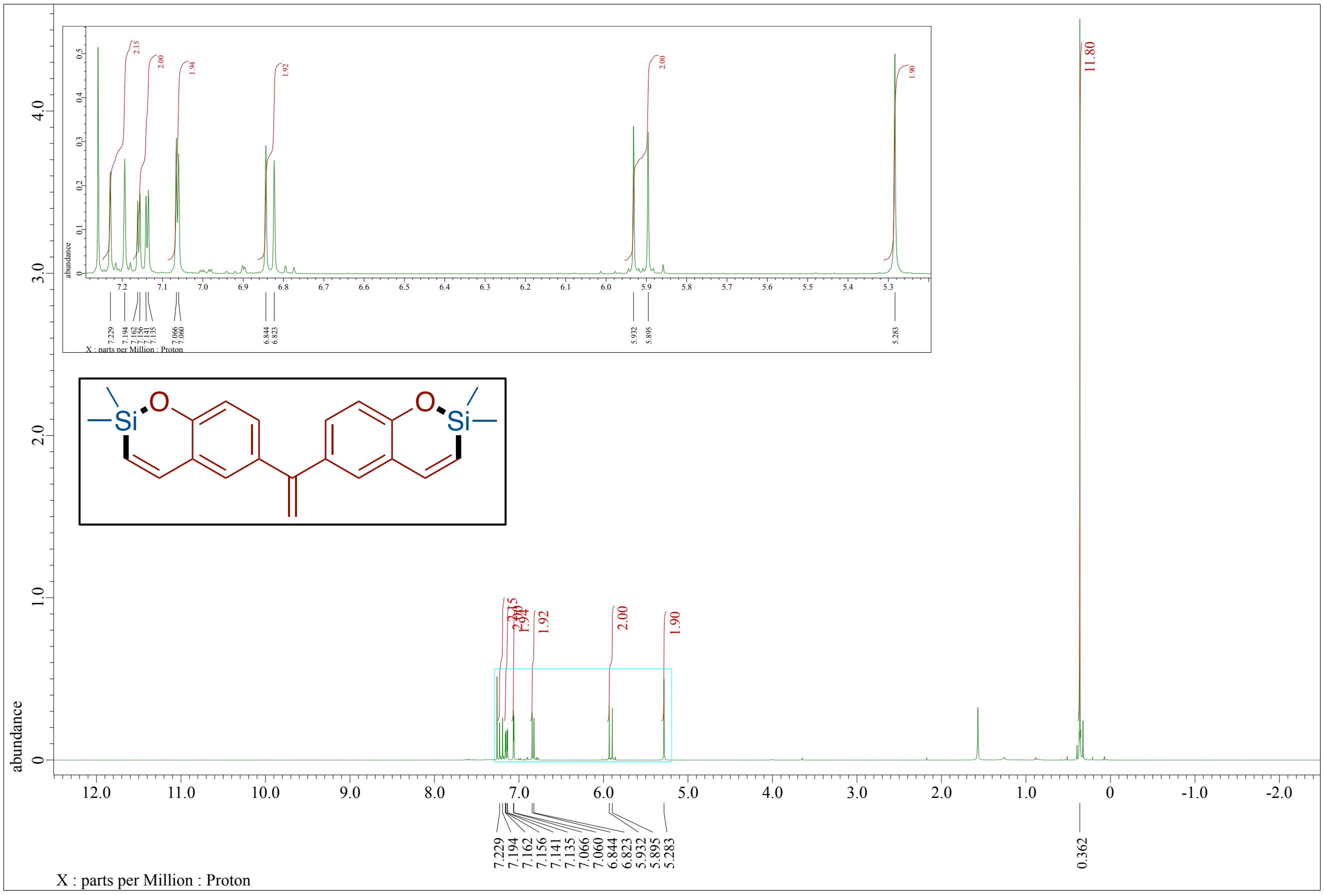

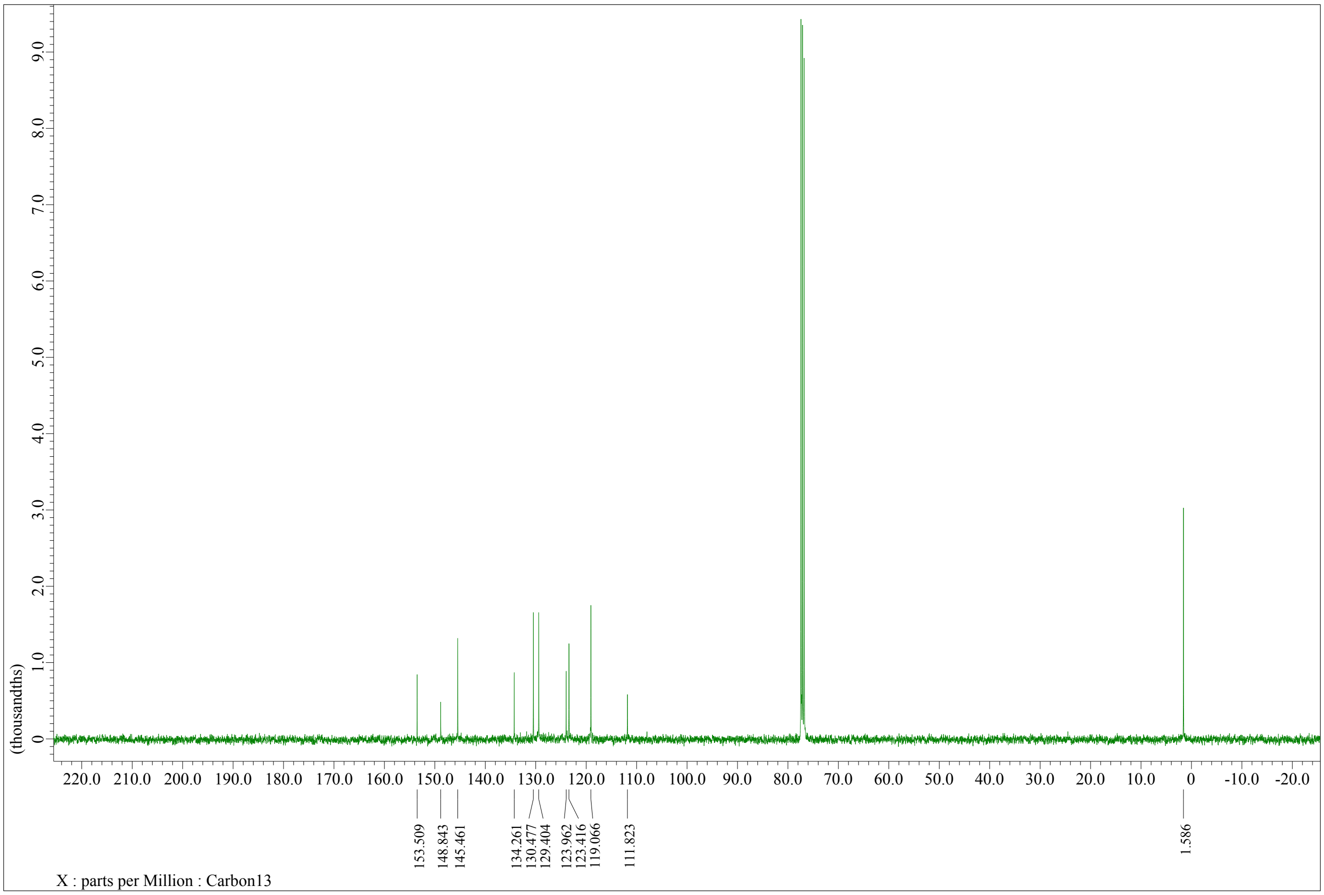

Figure S50. ^{1}H and ^{13}C NMR of **2mA**.

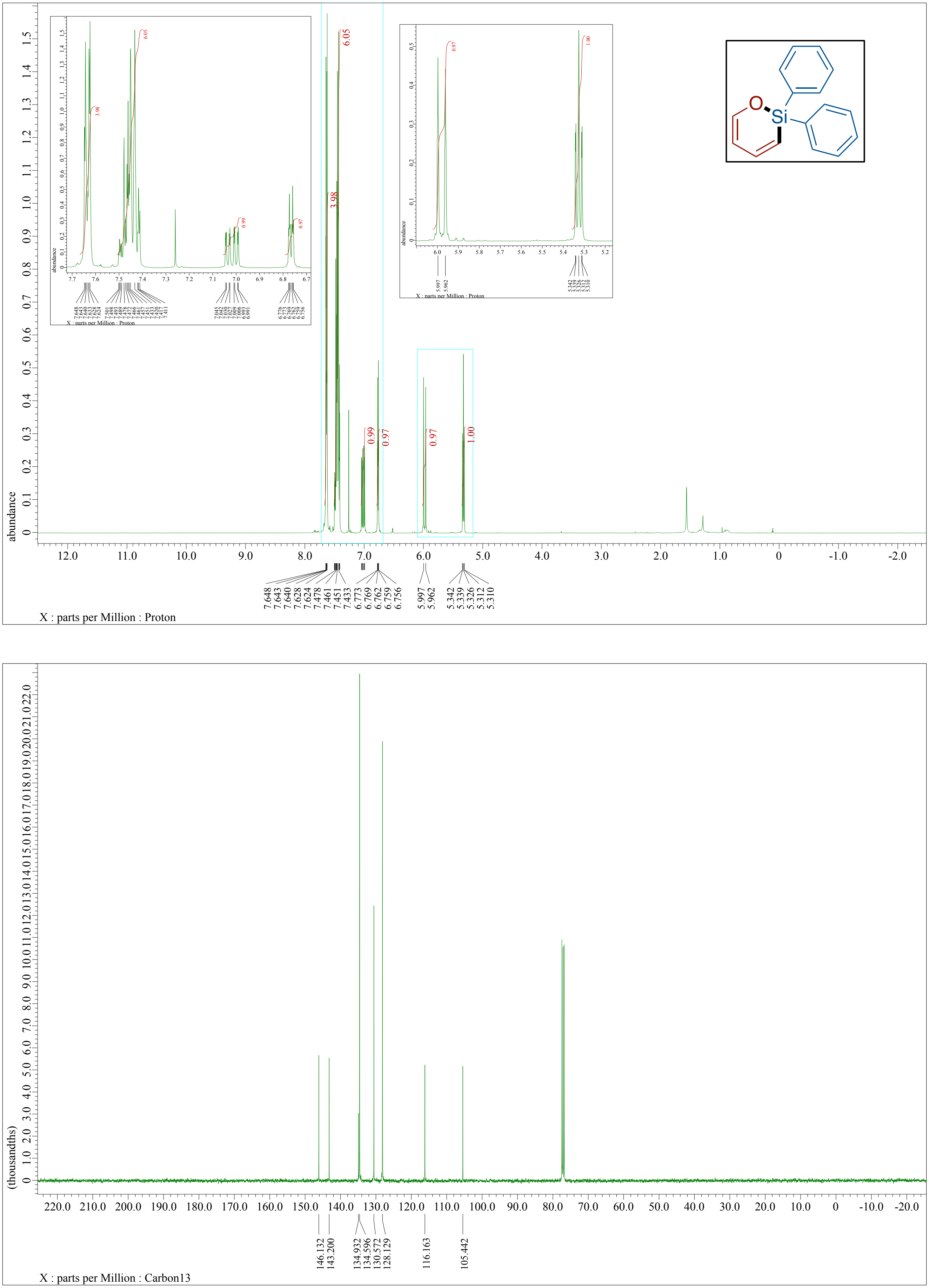

Figure S51. ^{1}H and ^{13}C NMR of **4aJ**.

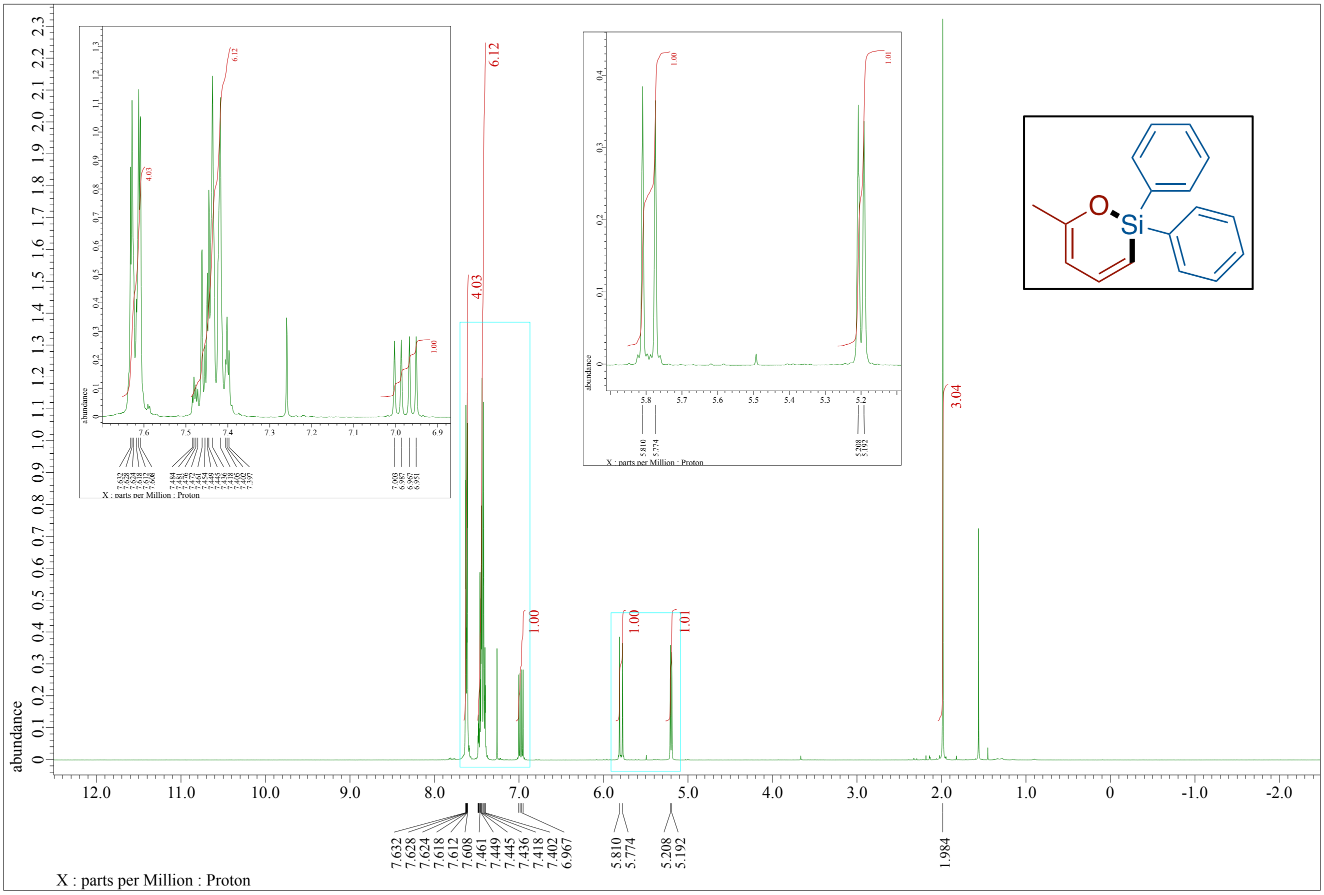

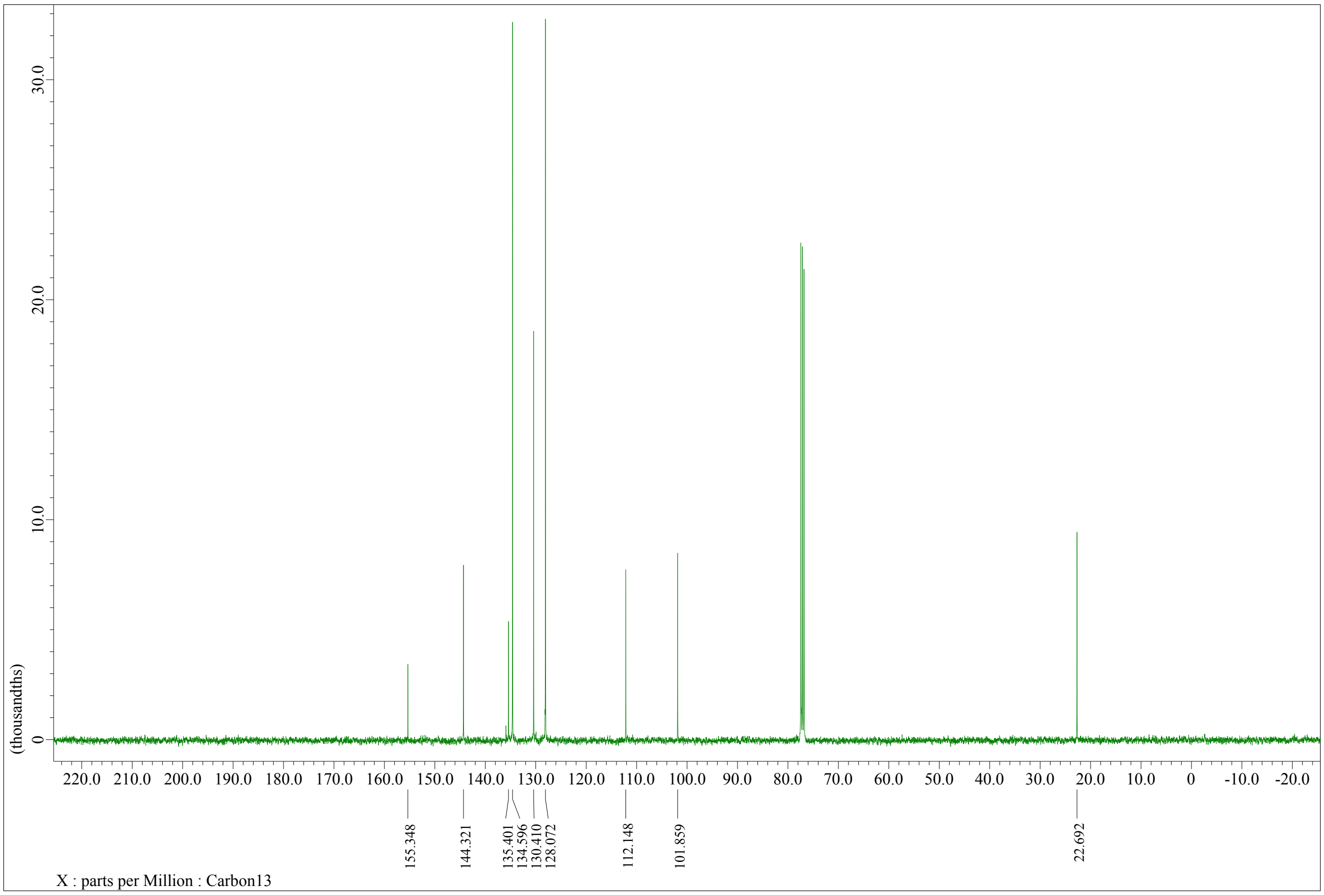

Figure S52. ^{1}H and ^{13}C NMR of **4bJ**.

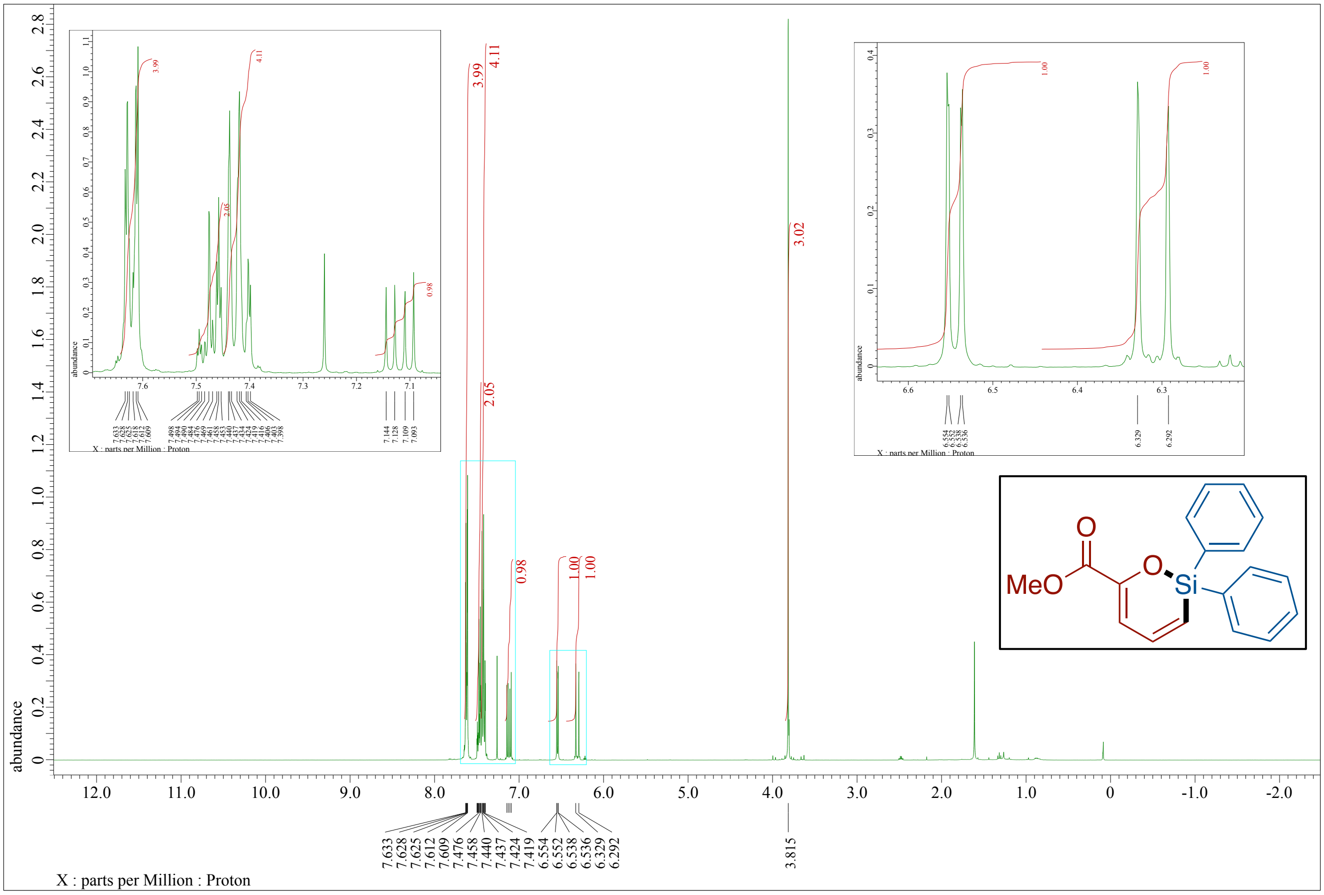

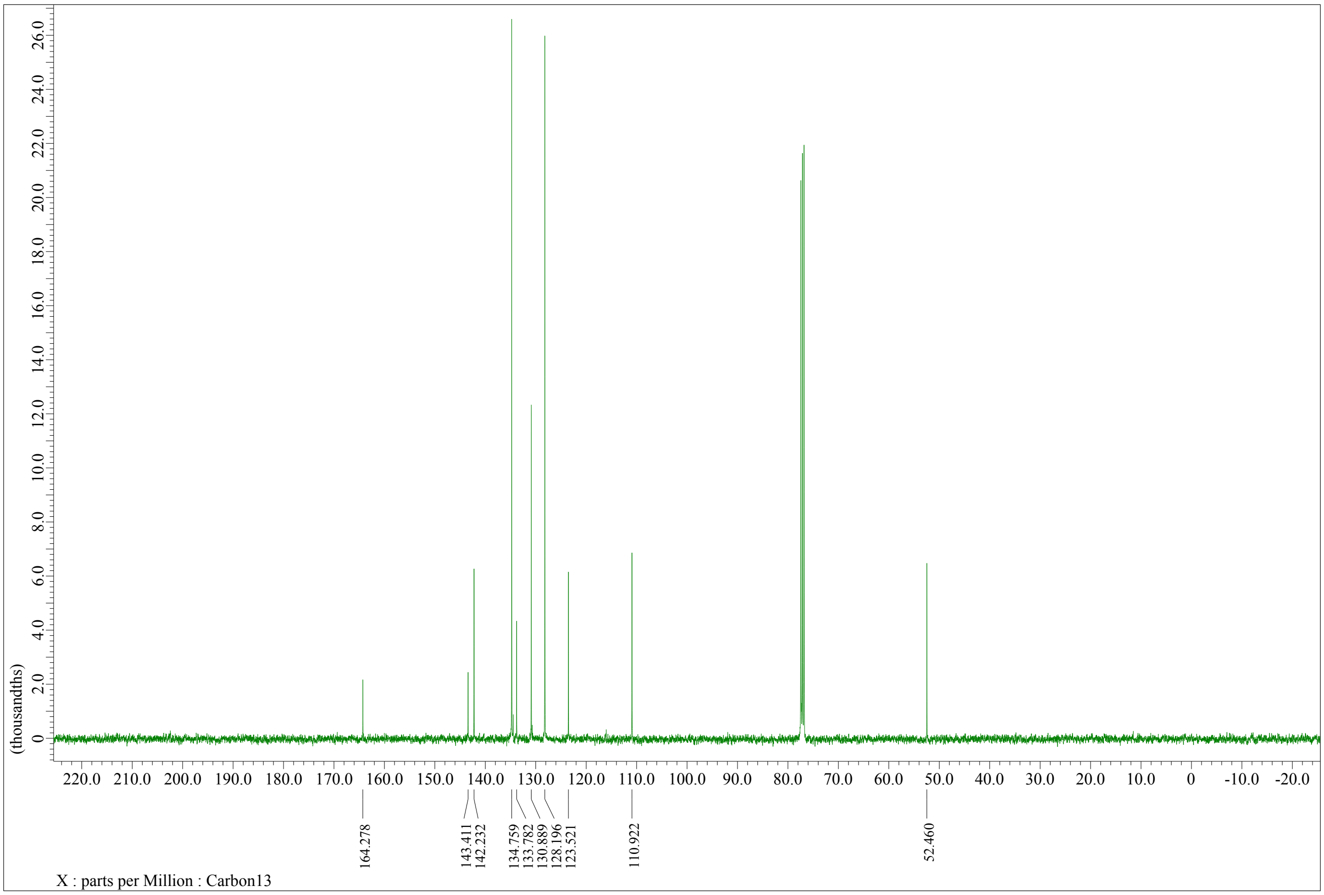

Figure S53. ^{1}H and ^{13}C NMR of **4cJ**

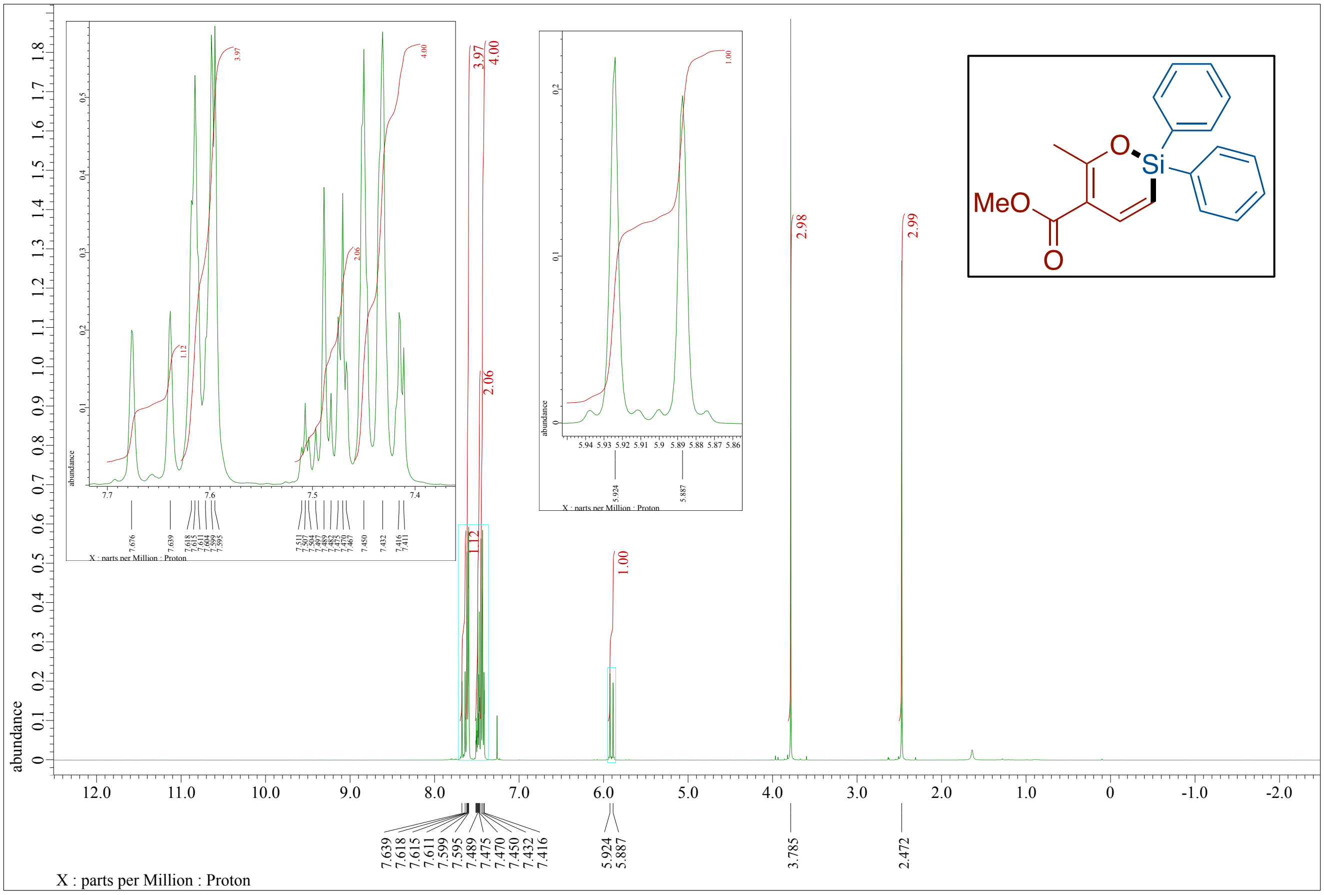

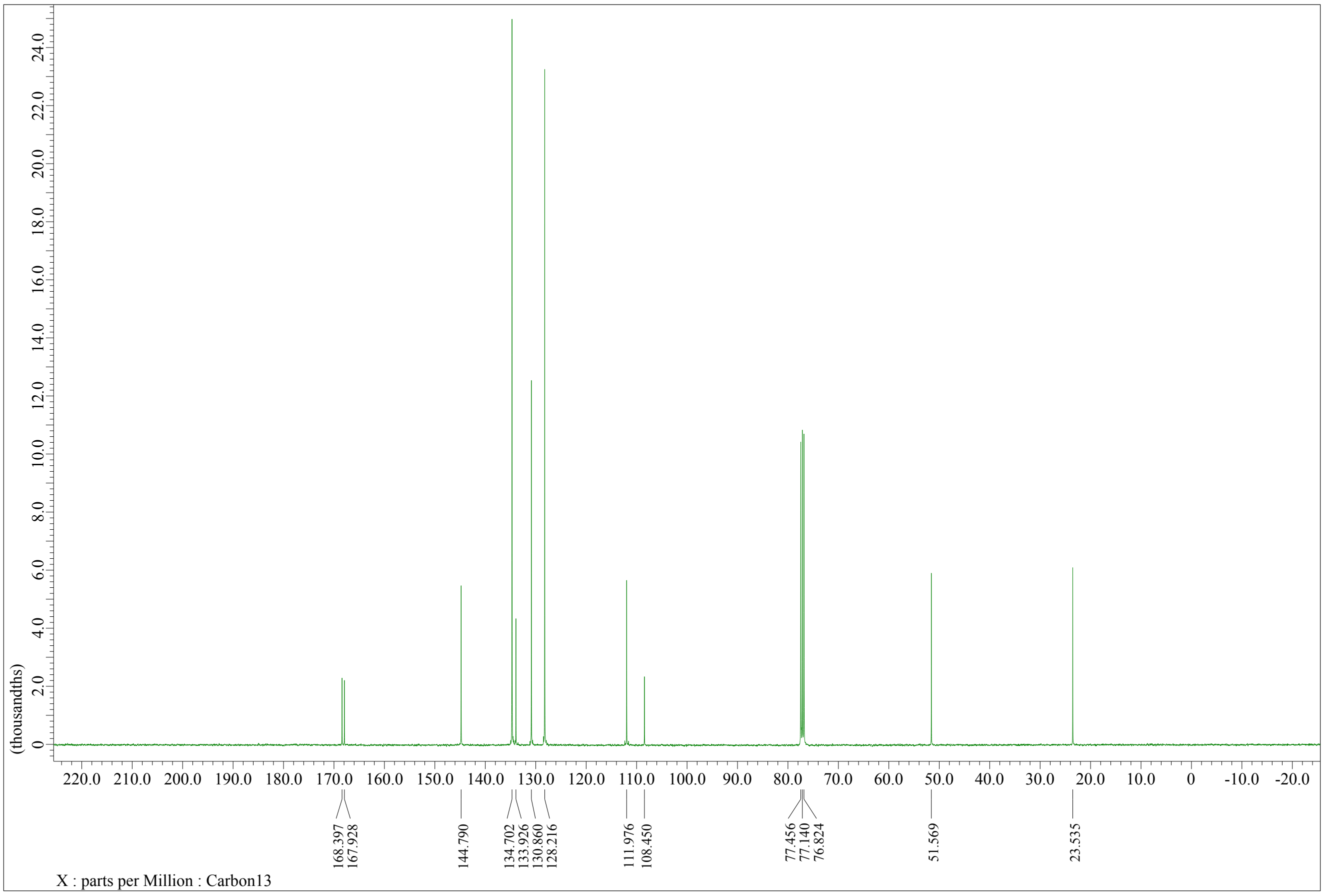

Figure S54. ^{1}H and ^{13}C NMR of **4dJ**.

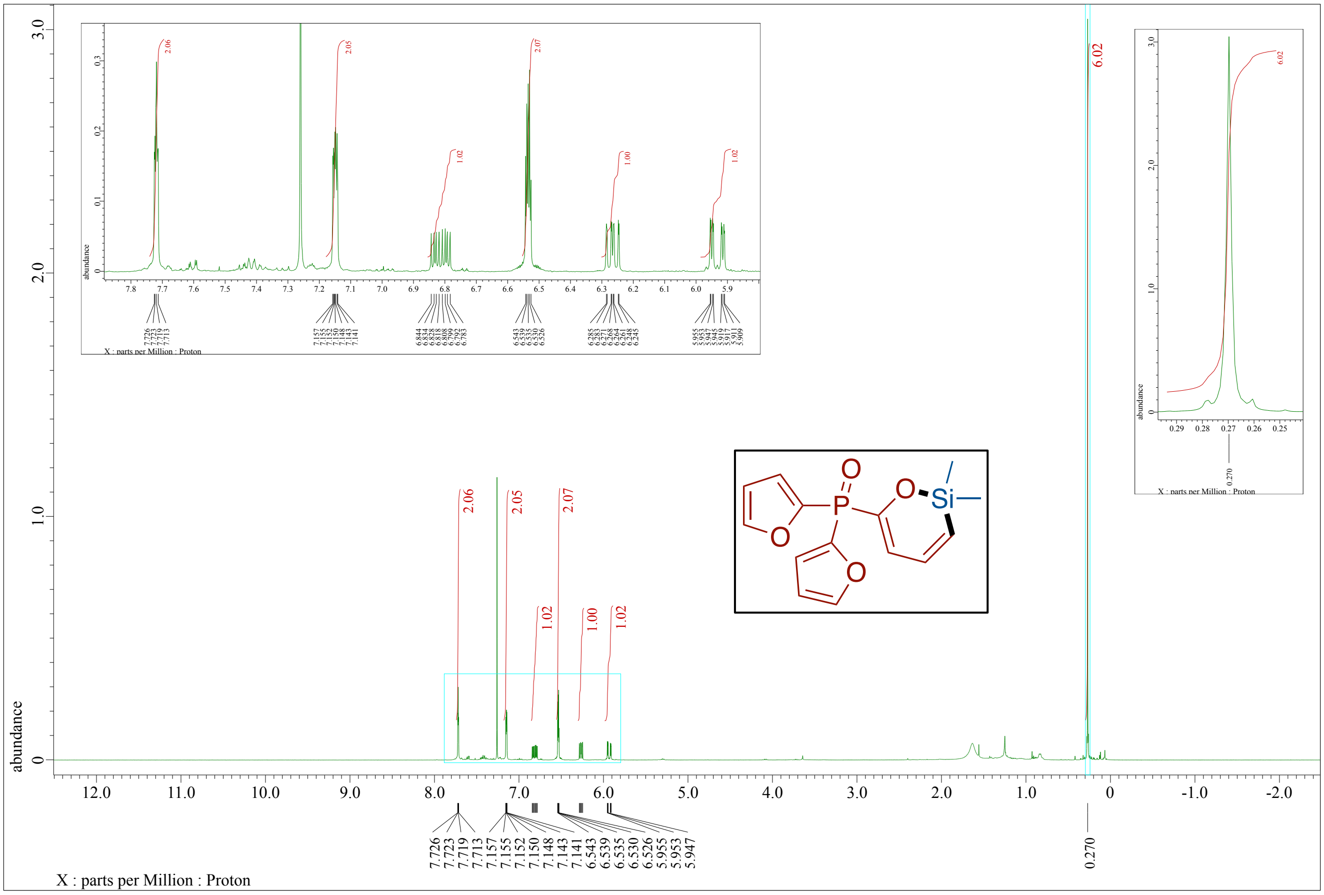

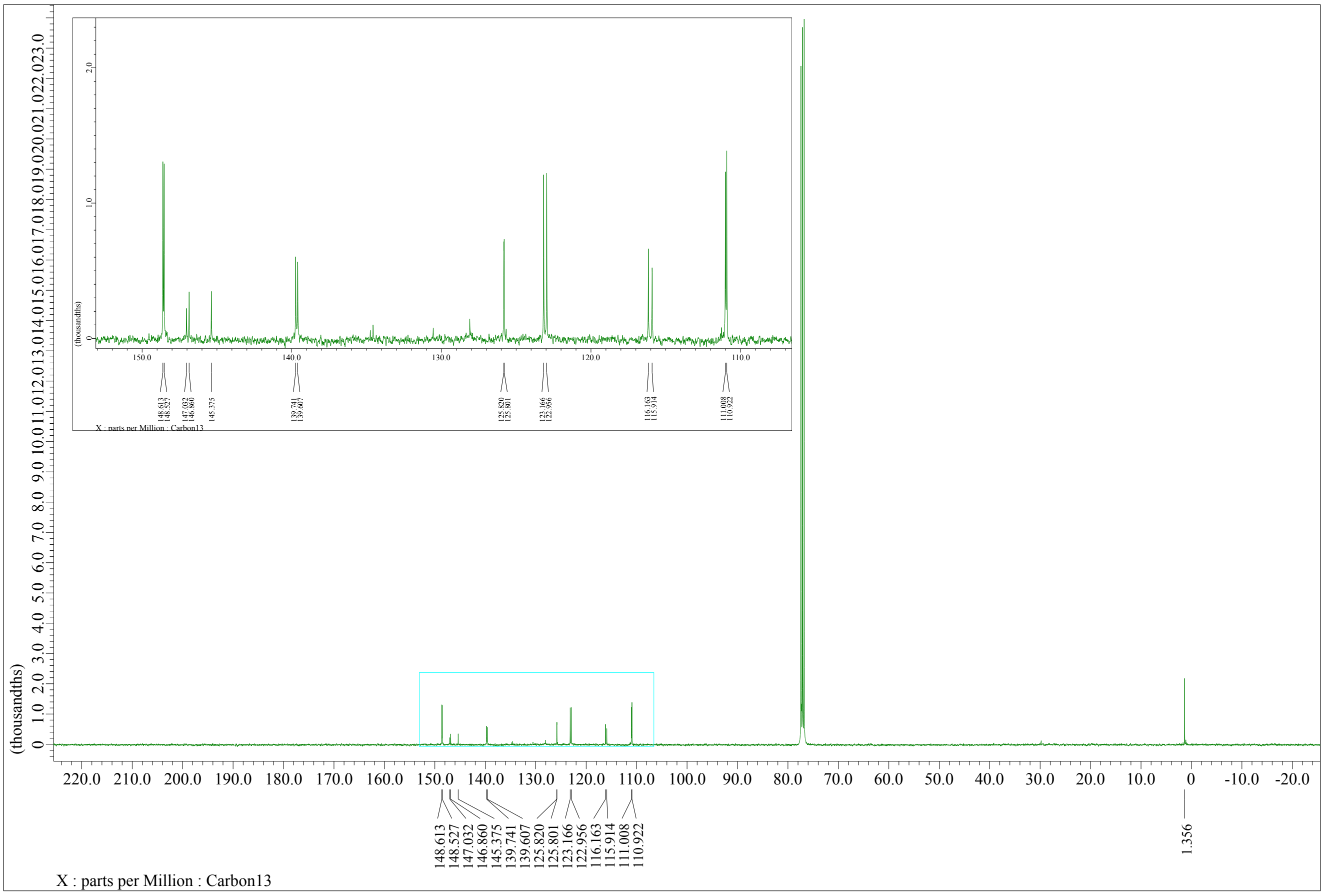

Figure S55. ^{1}H and ^{13}C NMR of **4eA**.

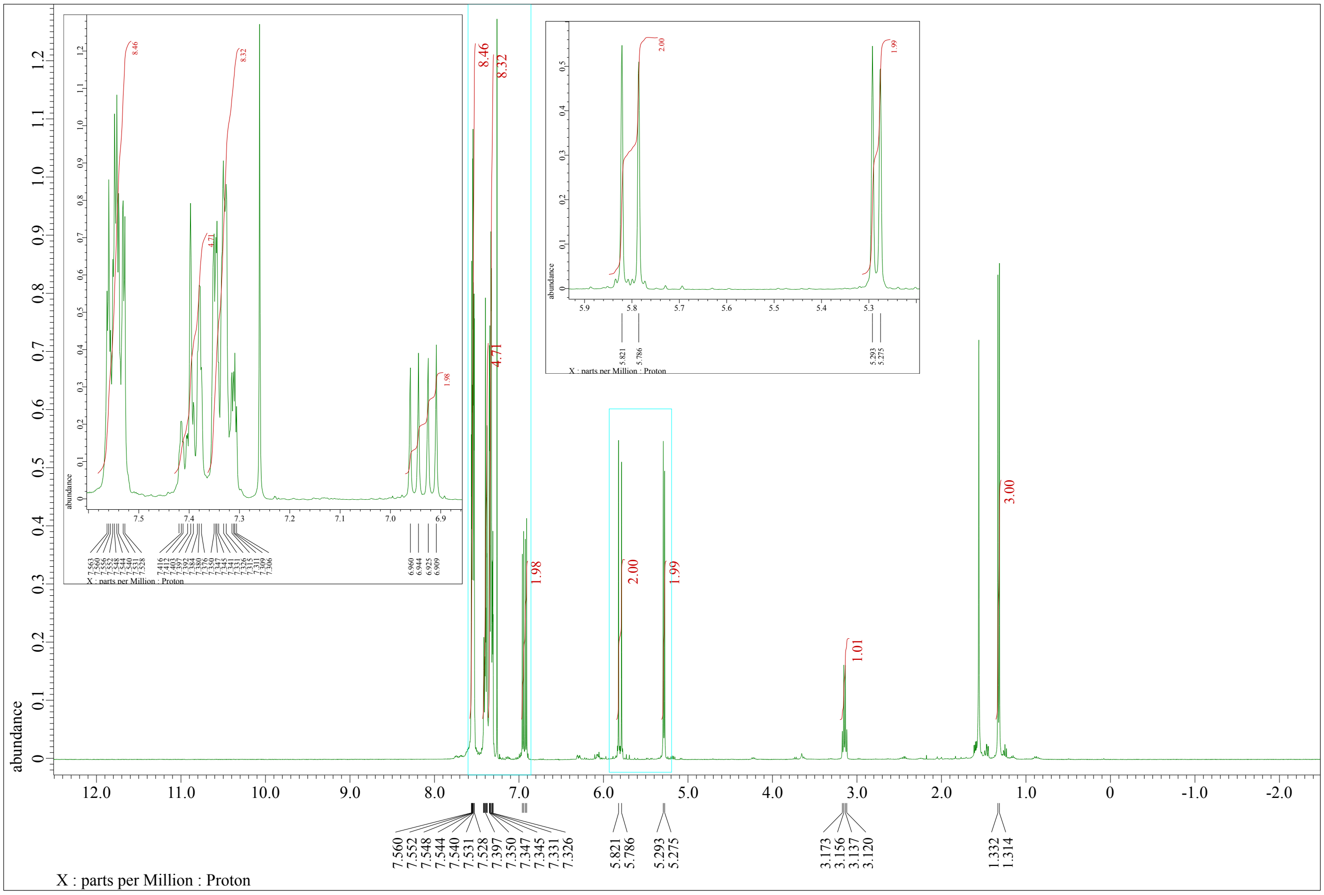

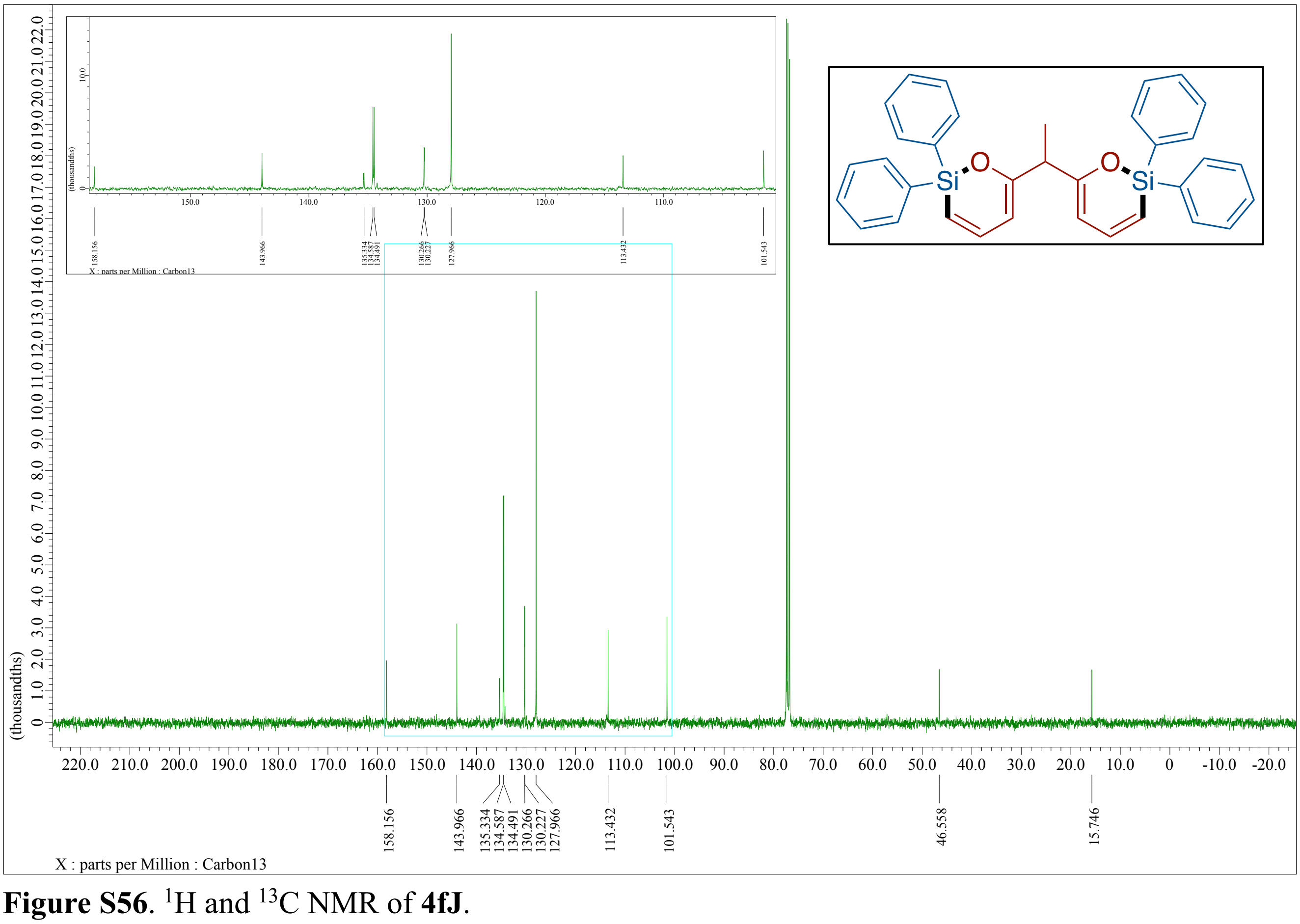

Figure S56. ^{1}H and ^{13}C NMR of **4fJ**.

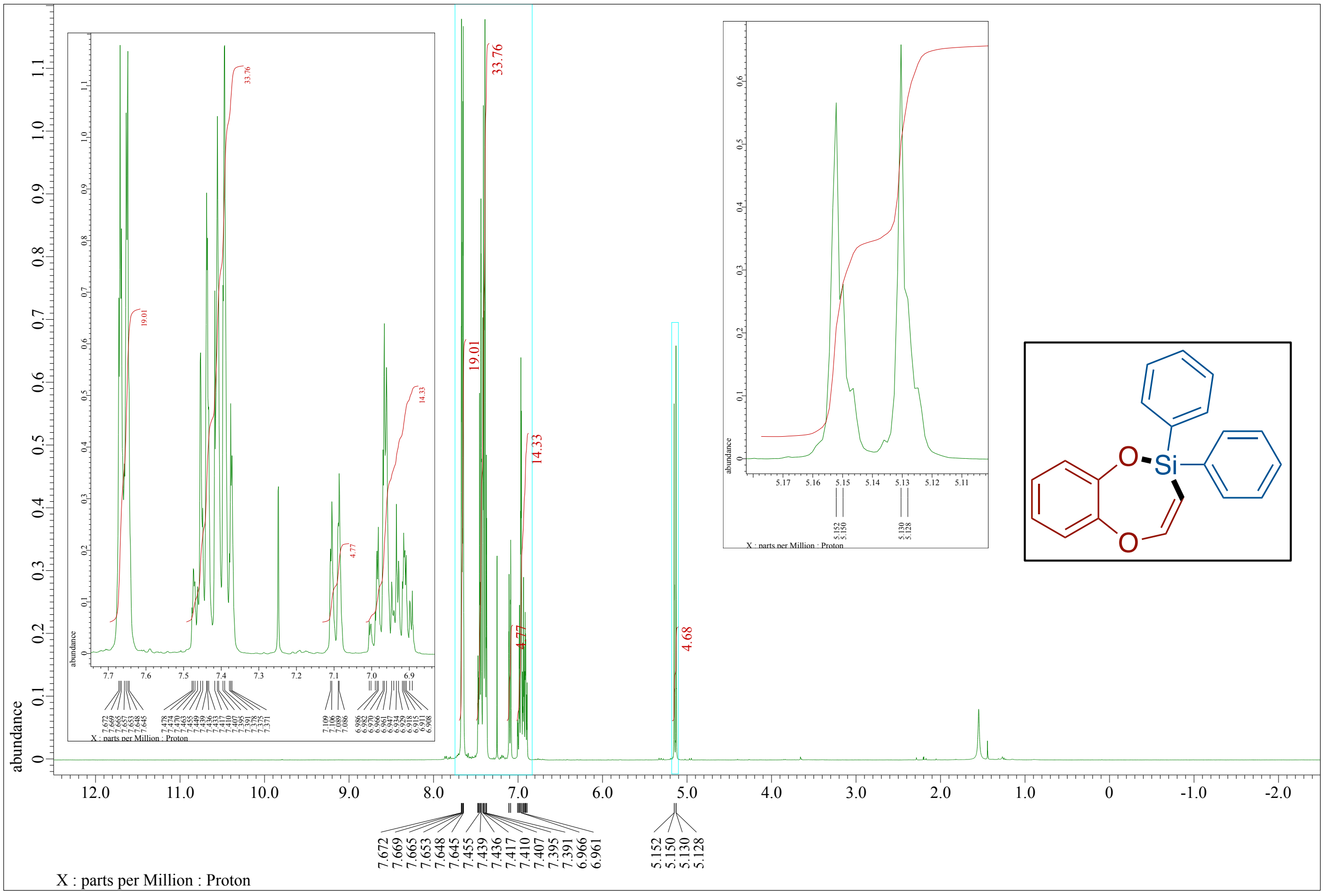

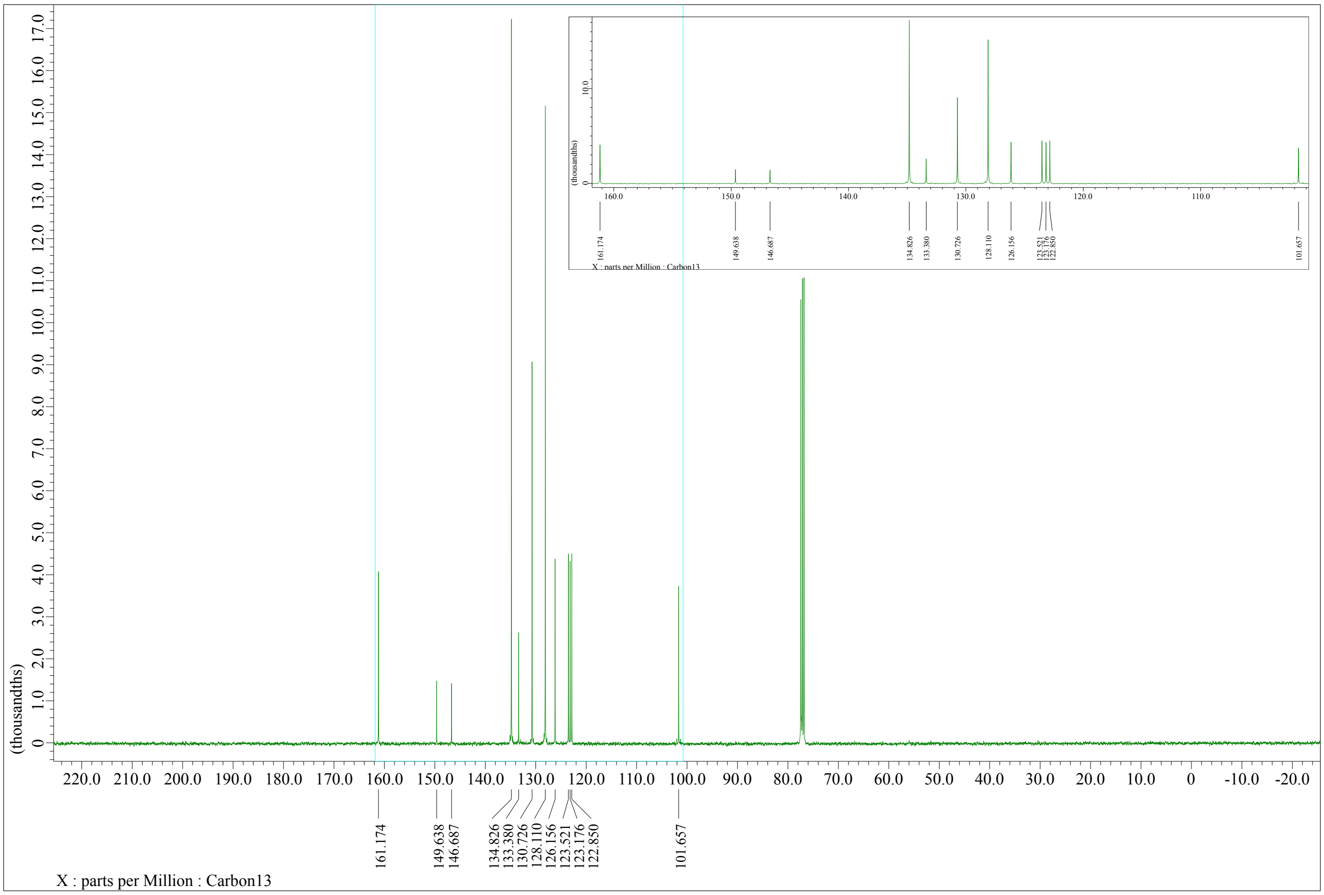

Figure S57. ^{1}H and ^{13}C NMR of **6J**.

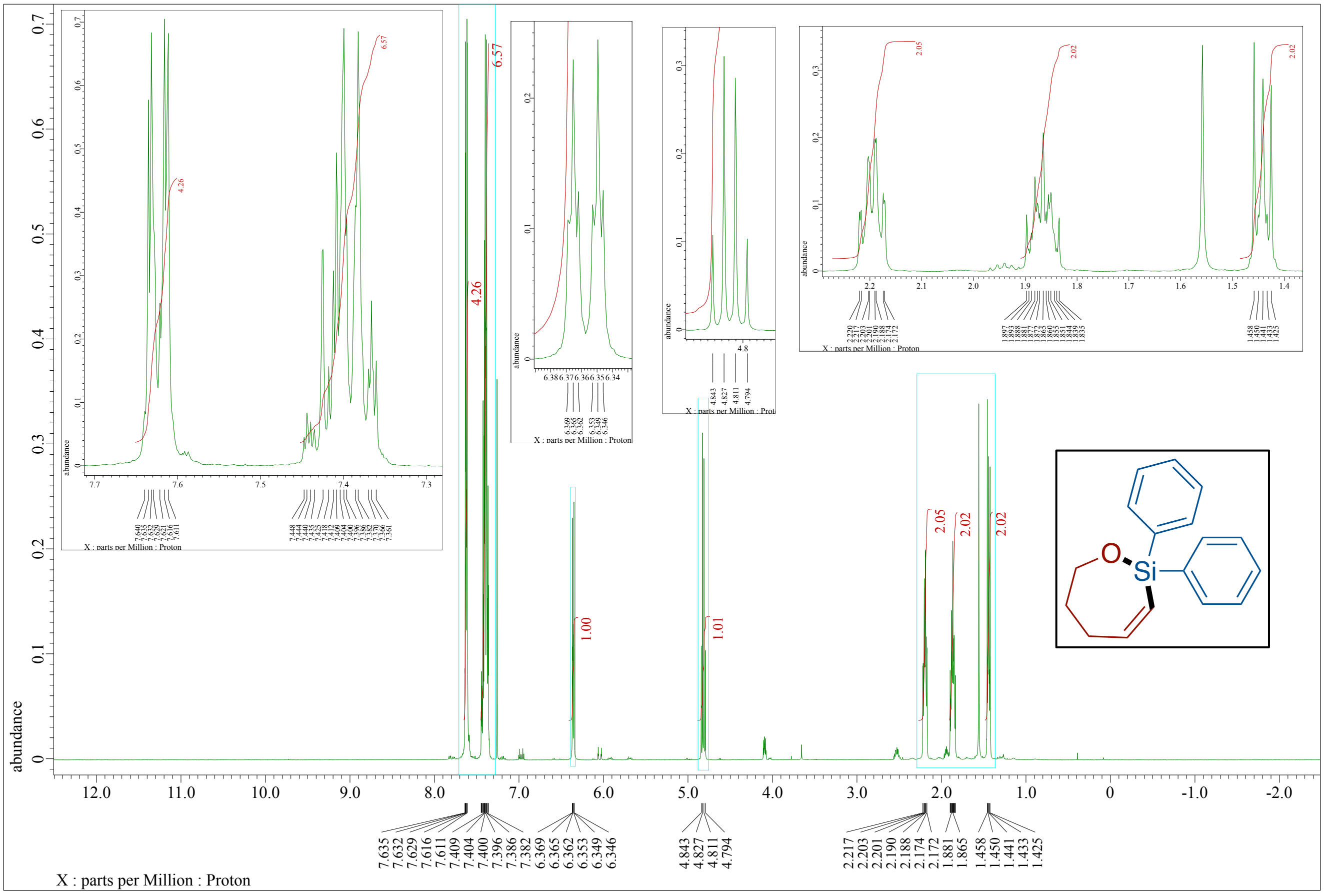

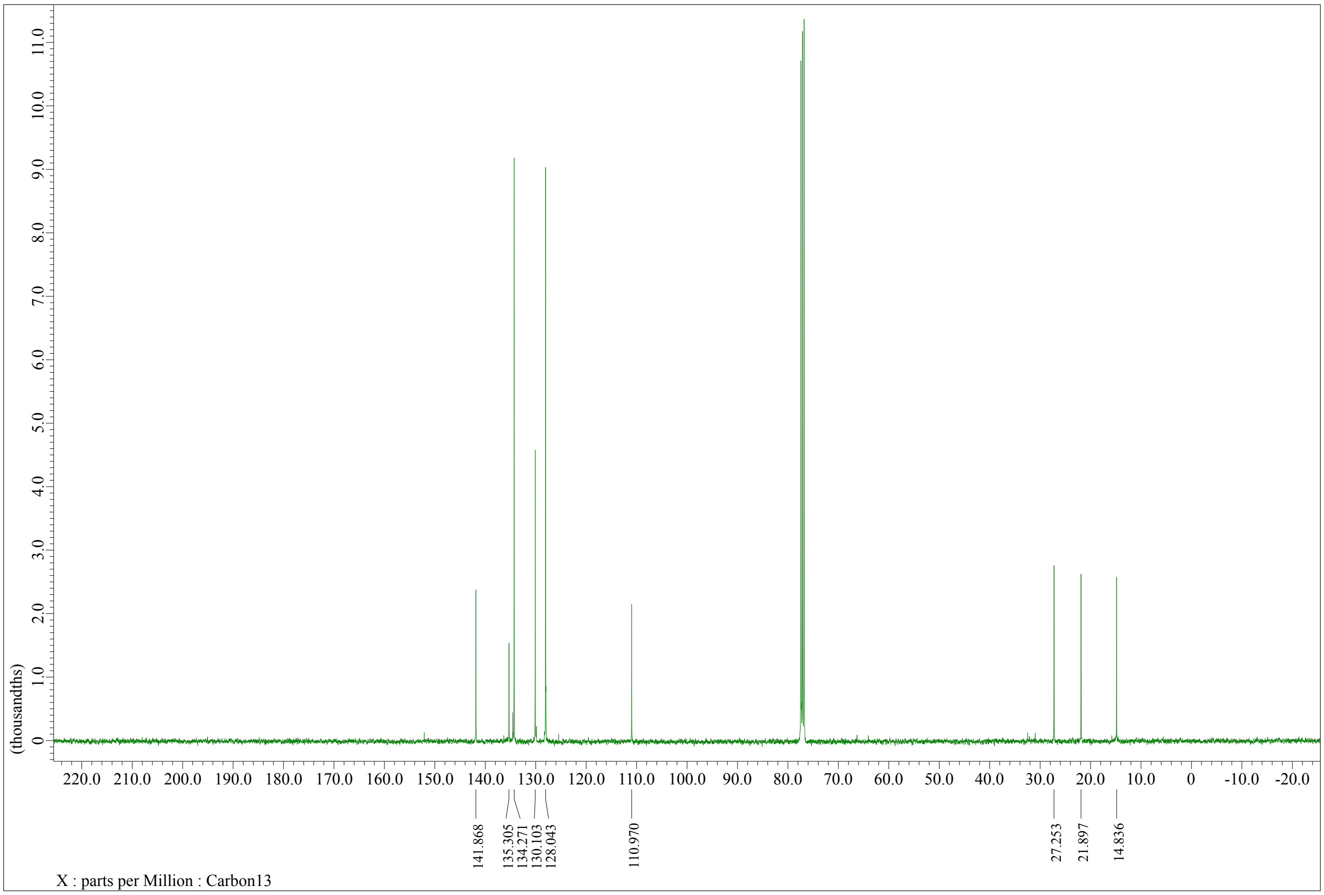

Figure S58. ^{1}H and ^{13}C NMR of **8J**.

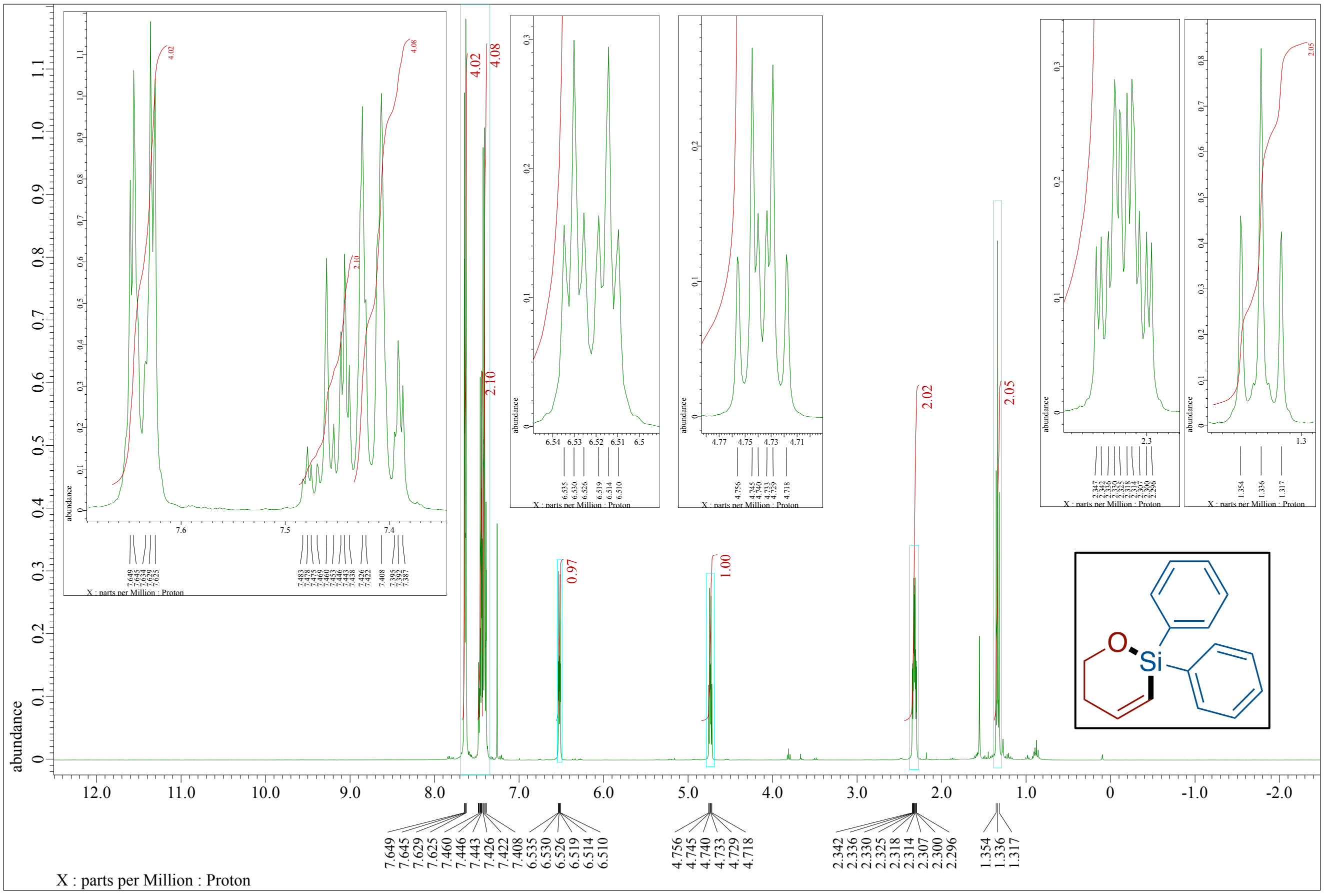

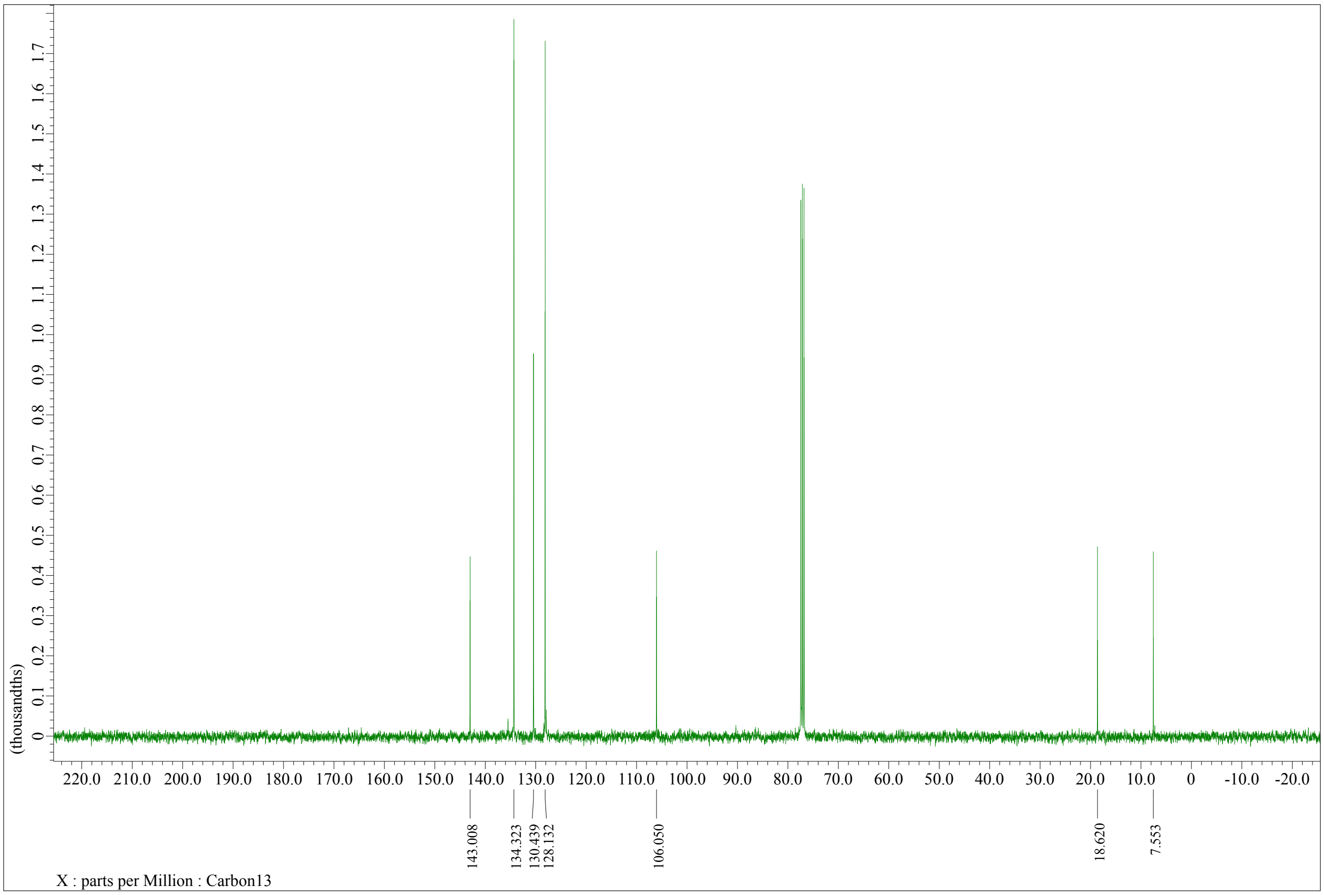

Figure S59. ^{1}H and ^{13}C NMR of **10J**.

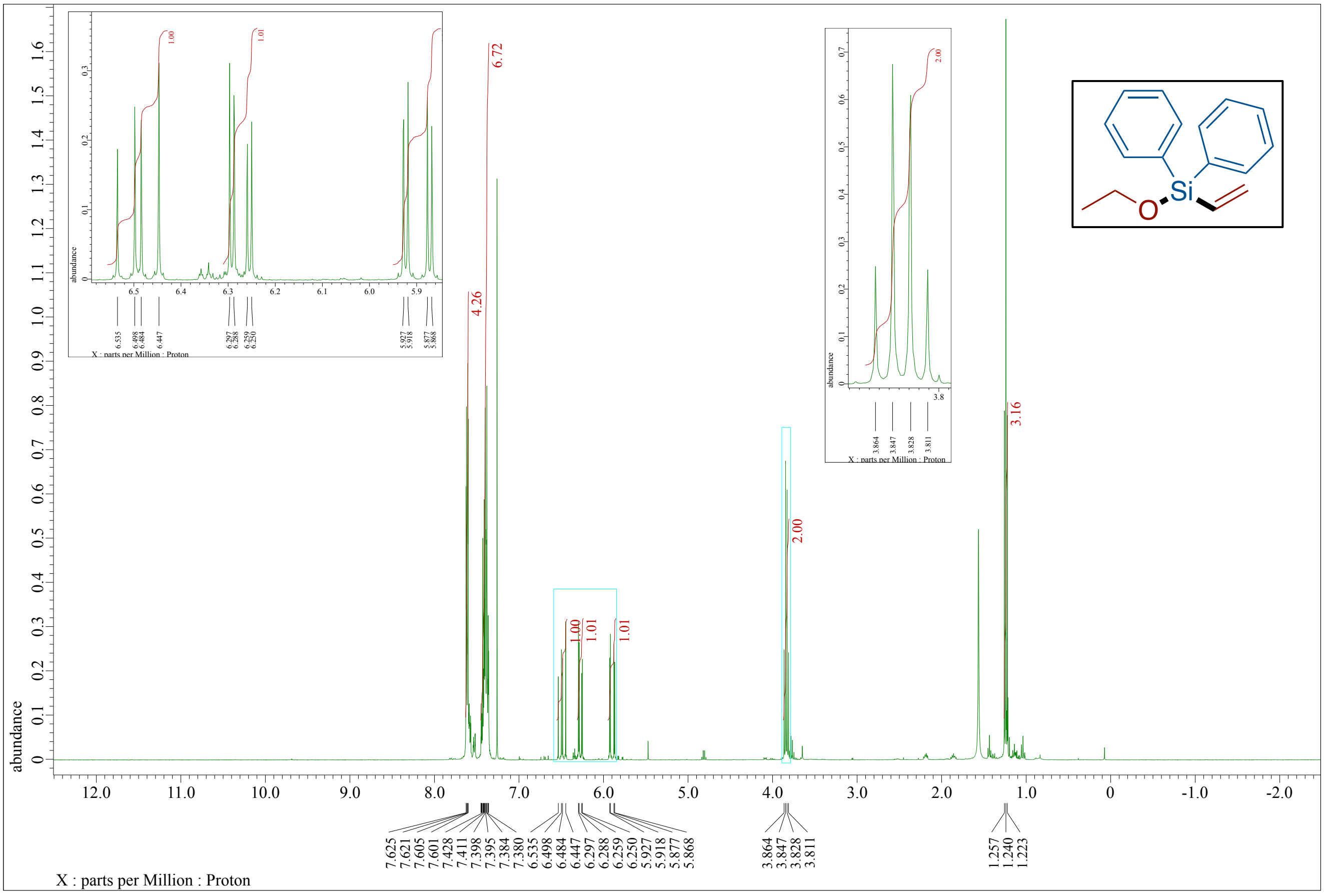

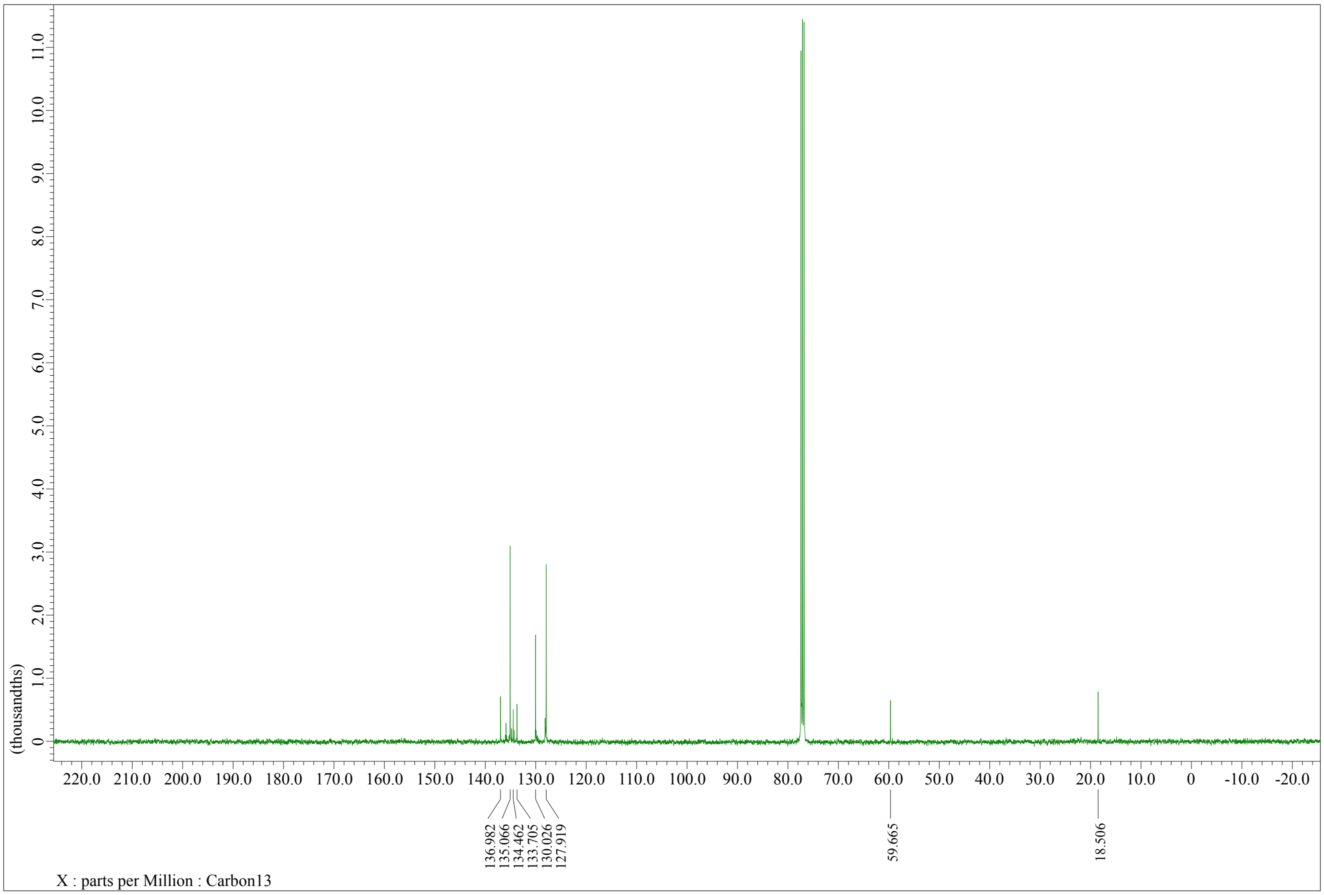

Figure S60. ^{1}H and ^{13}C NMR of **12J**.

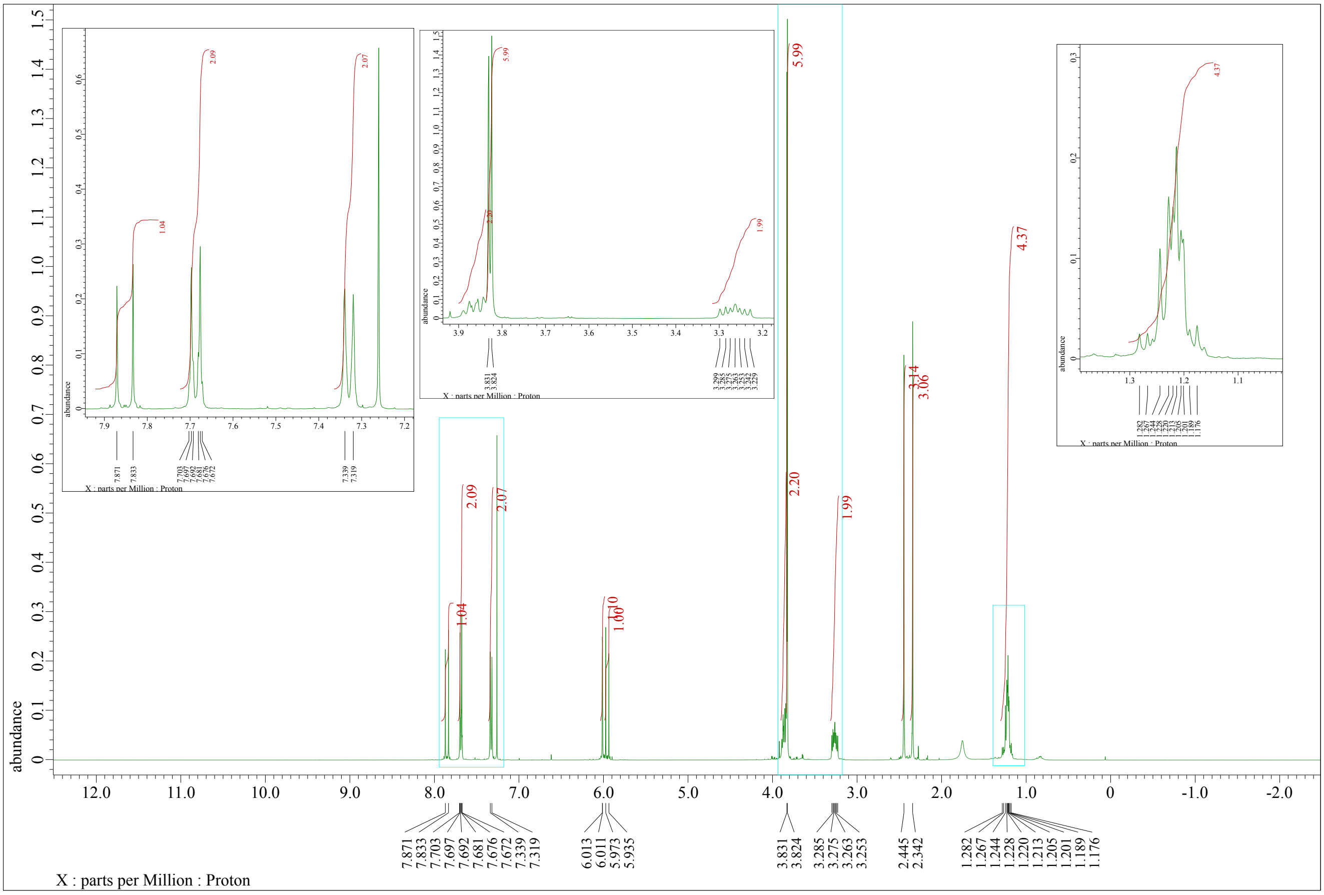

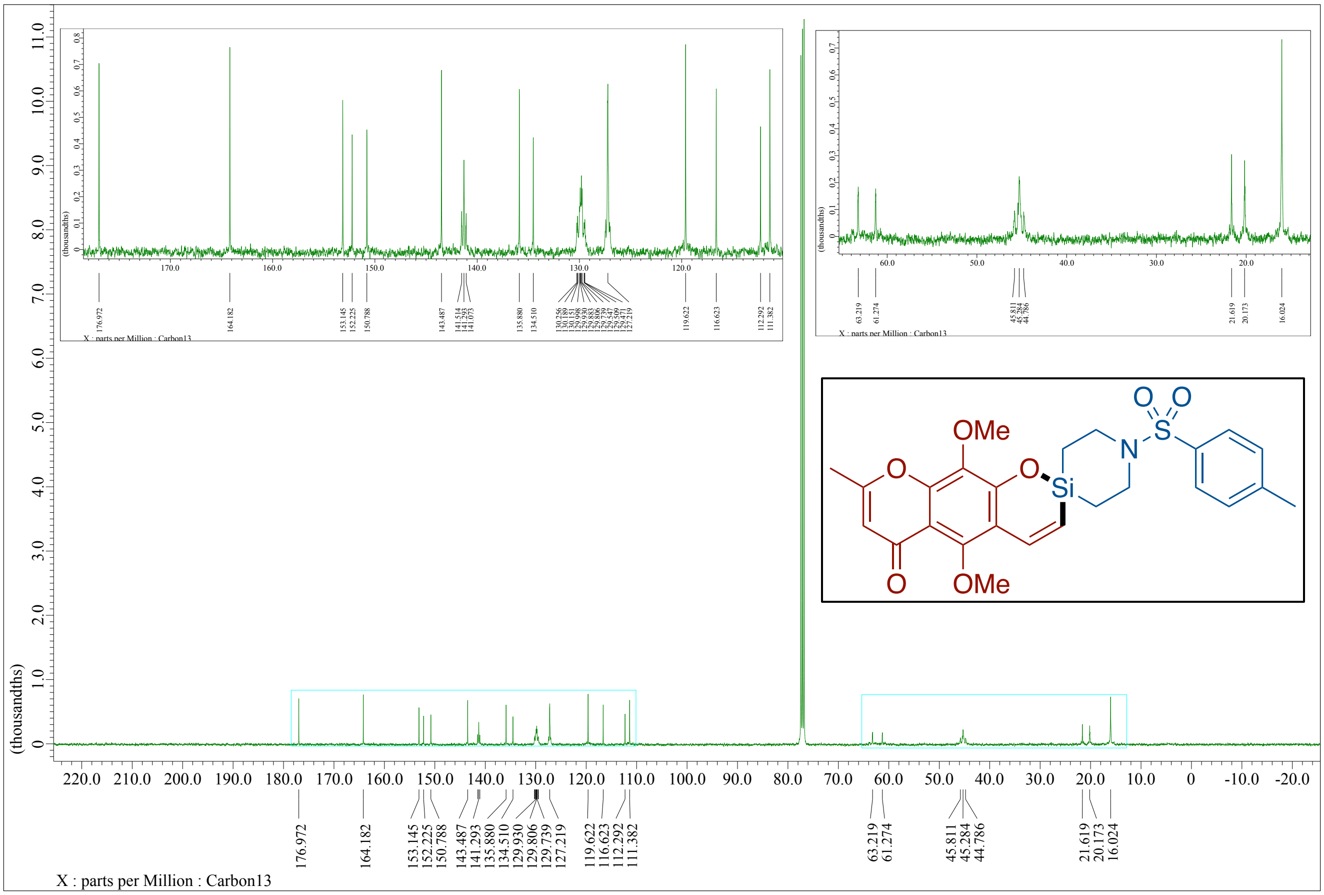

Figure S61. ^{1}H and ^{13}C NMR of **2mN**.

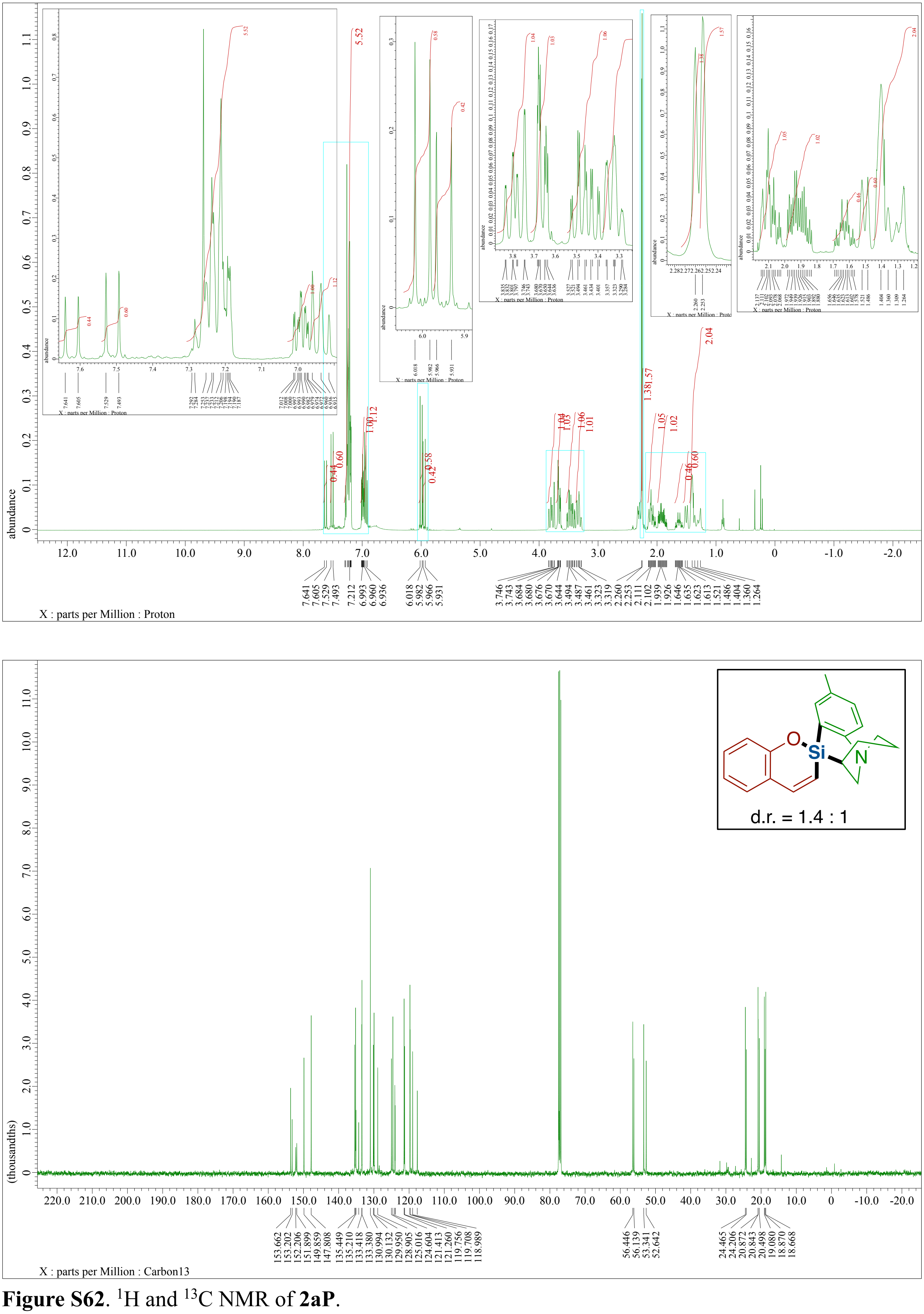

Figure S62. ^{1}H and ^{13}C NMR of **2aP**.

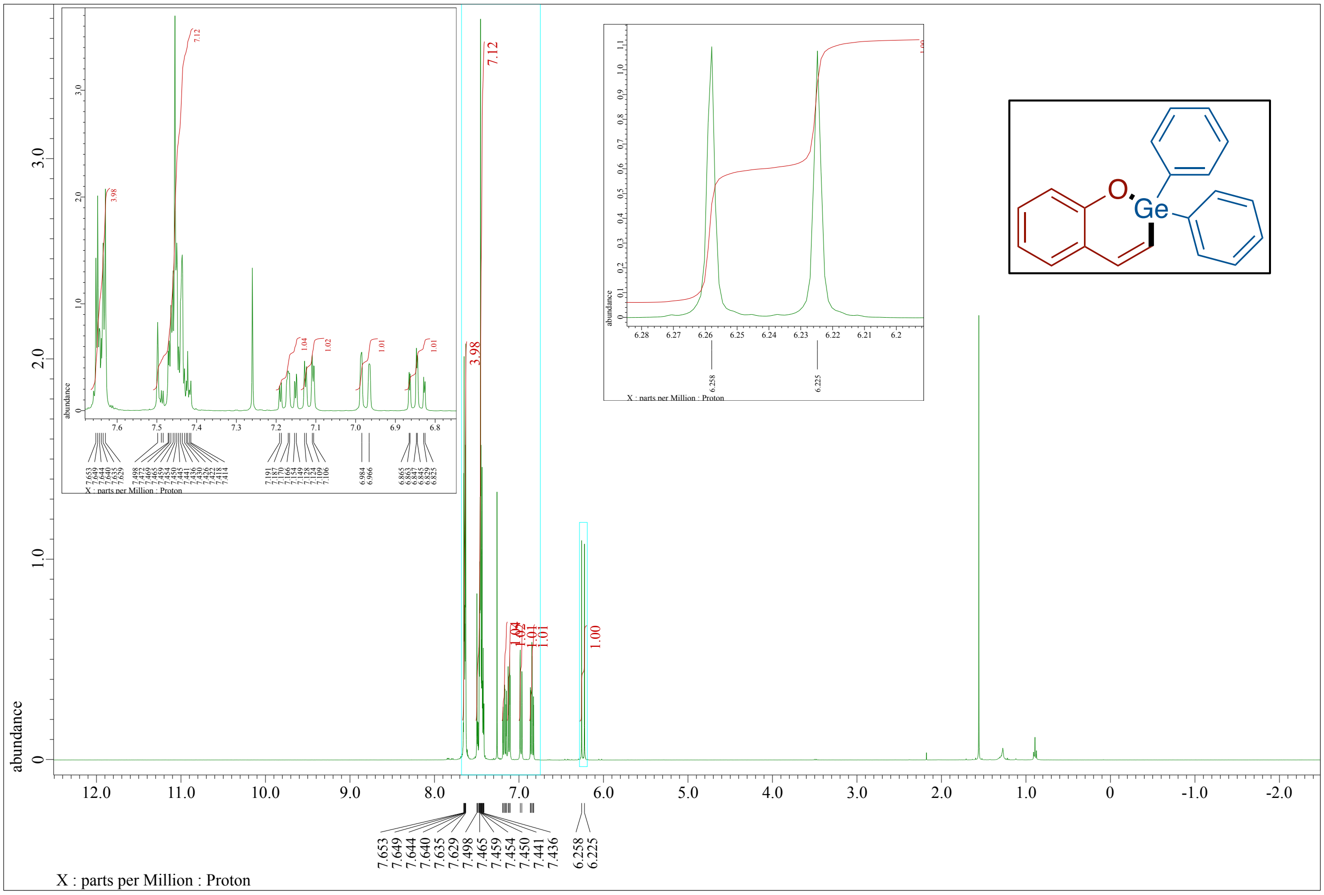

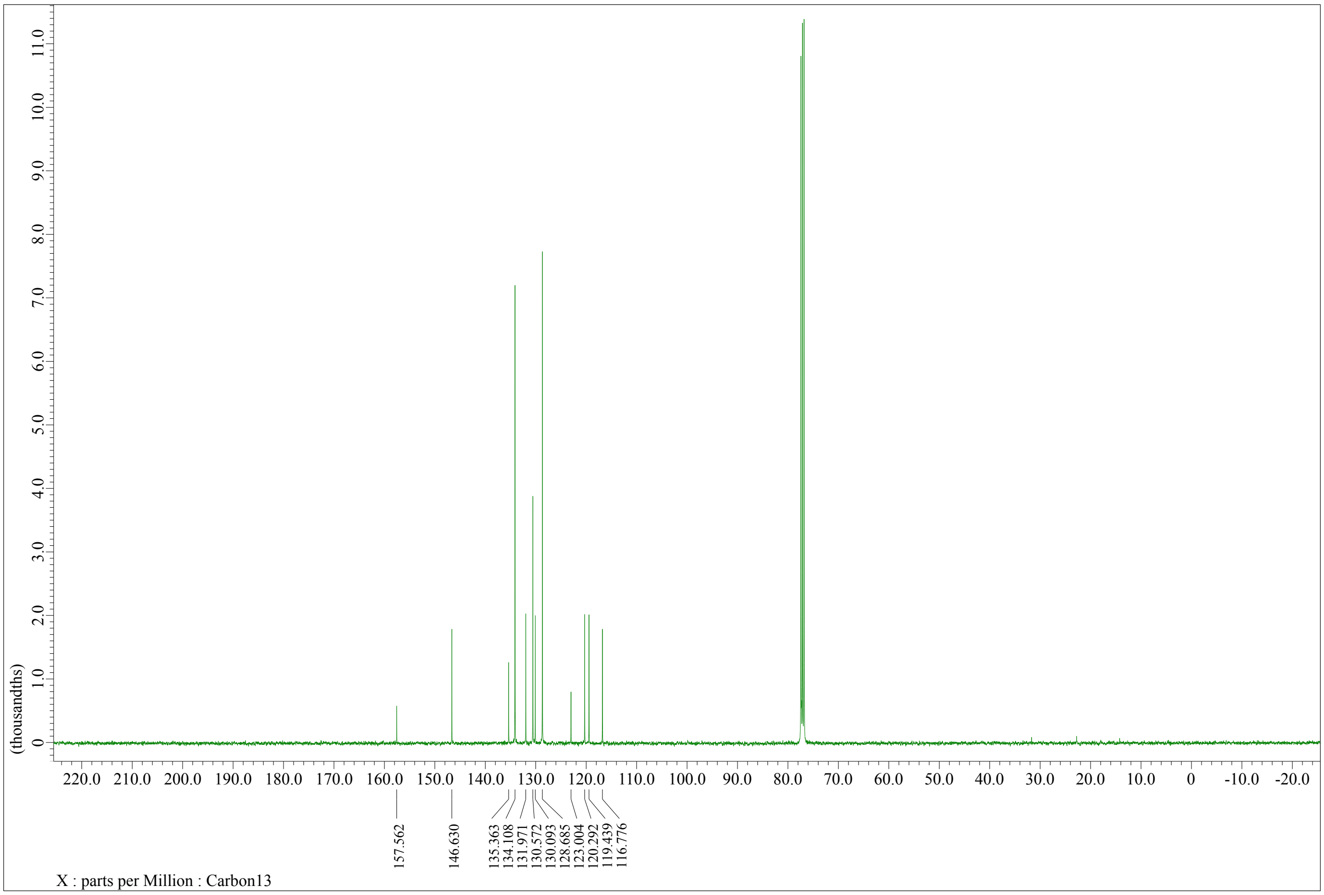

Figure S63. ^{1}H and ^{13}C NMR of **2aQ**.

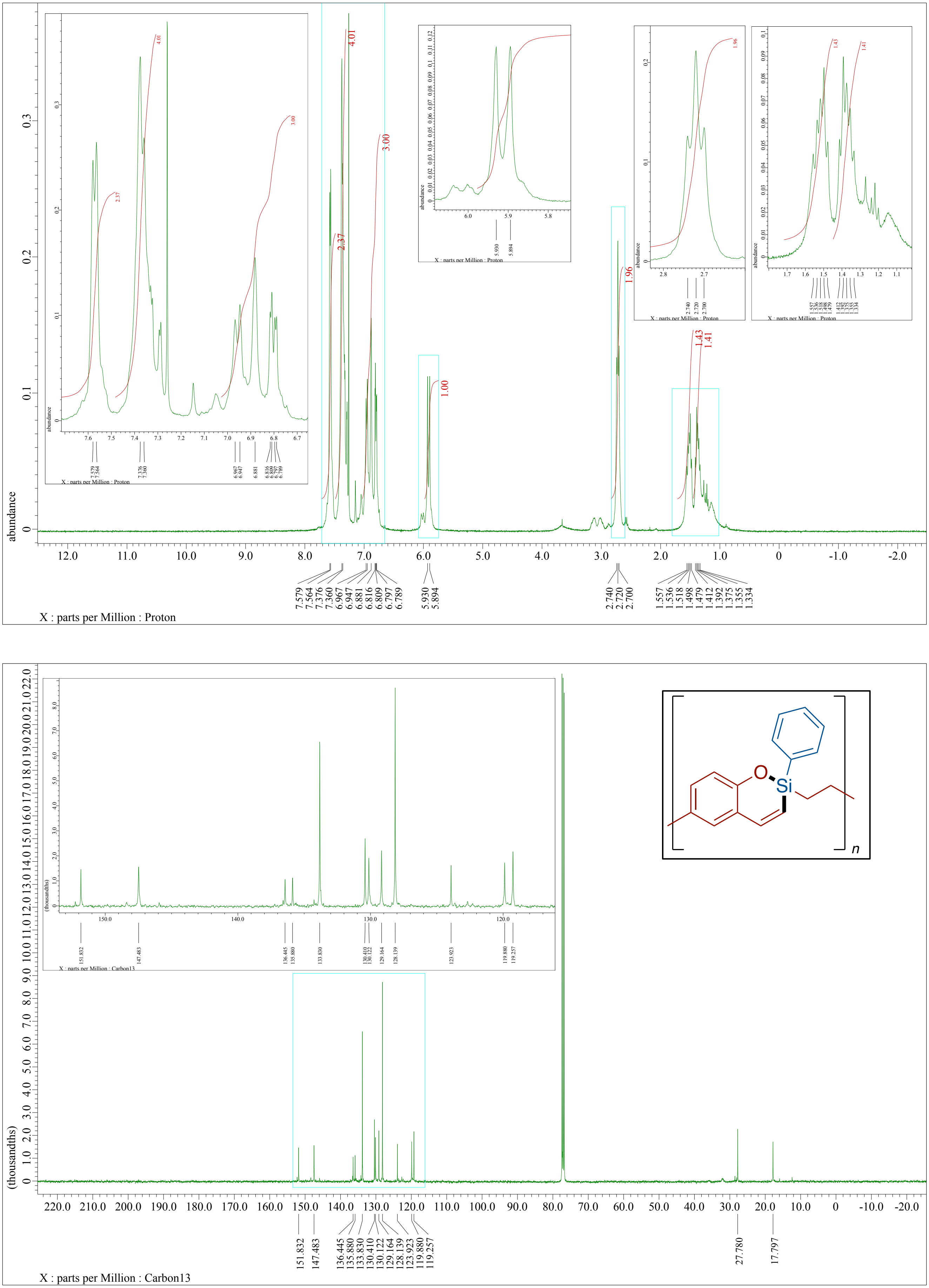

Figure S64. ^{1}H and ^{13}C NMR of **15**.

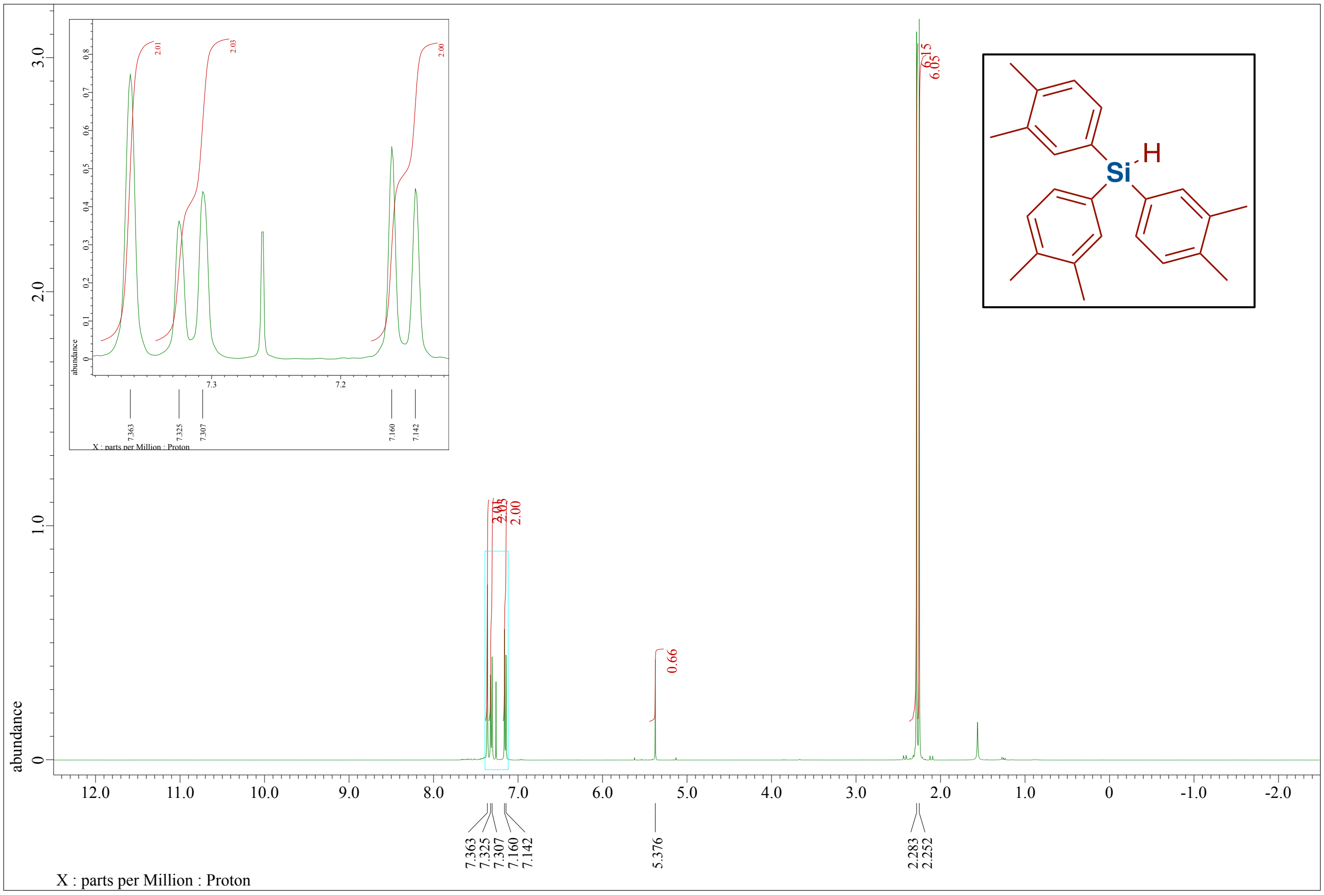

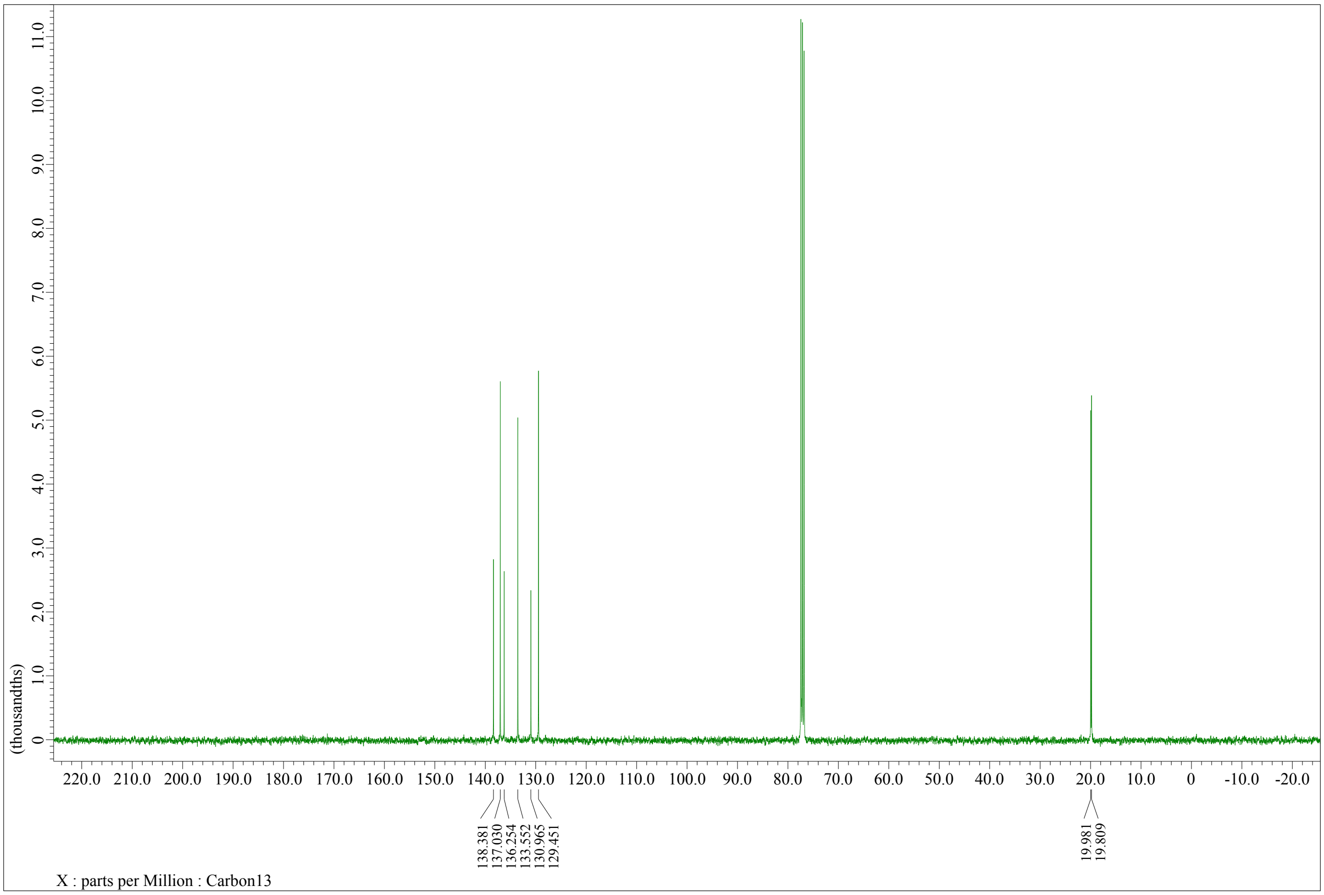

Figure S65. ^{1}H and ^{13}C NMR of **tris(3,4-dimethylphenyl)silane.**

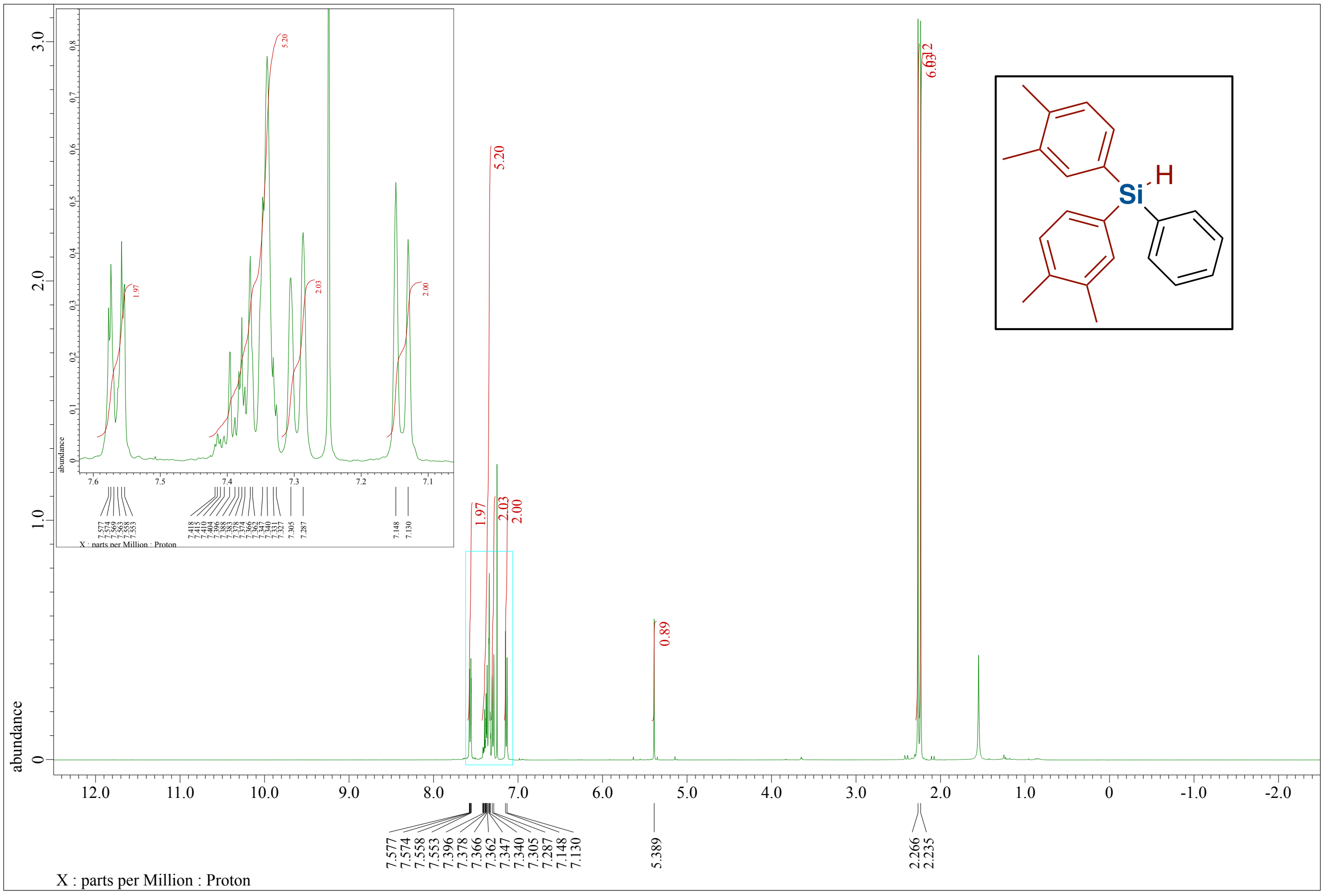

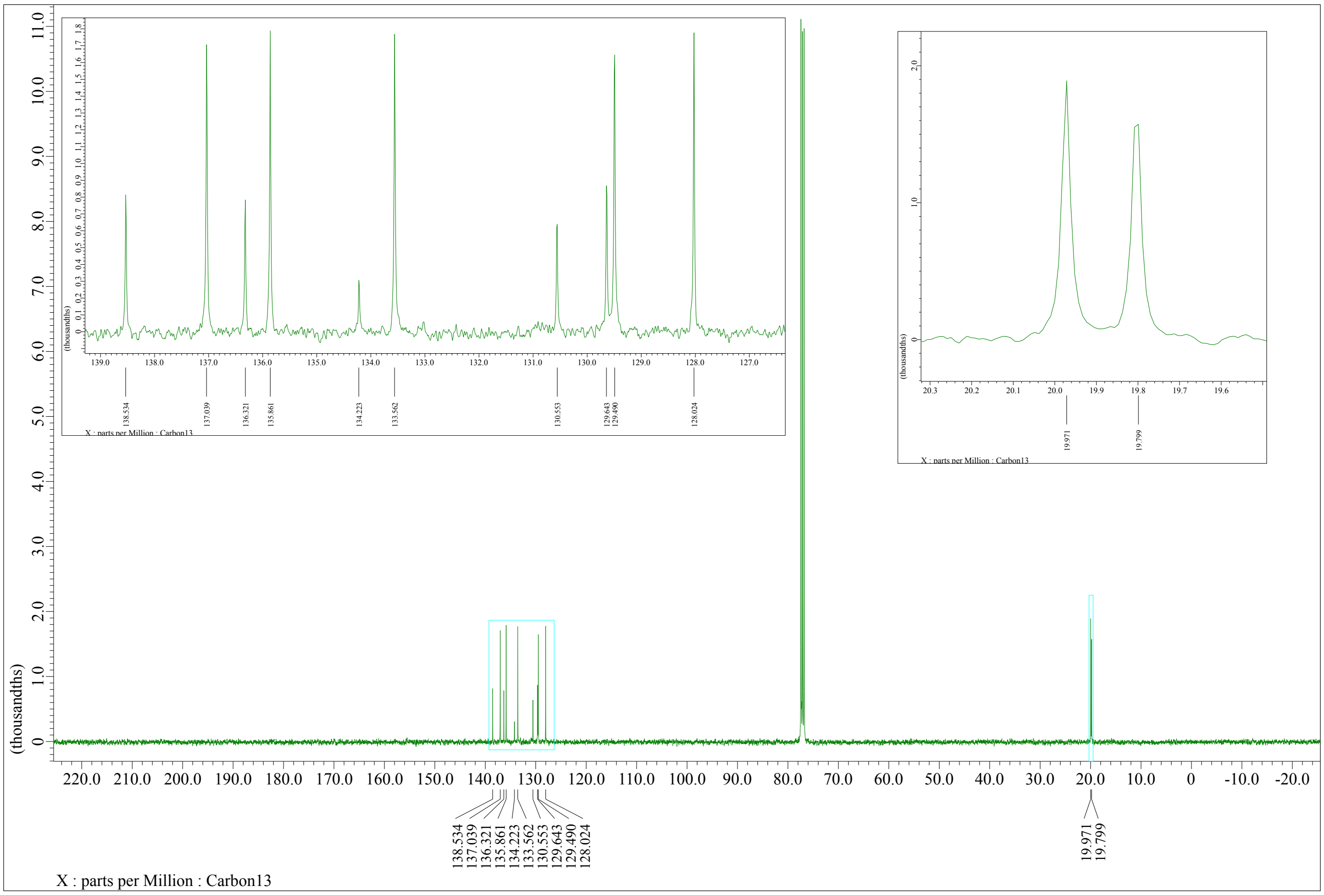

Figure S66. ^{1}H and ^{13}C NMR of **bis(3,4-dimethylphenyl)(phenyl)silane.**

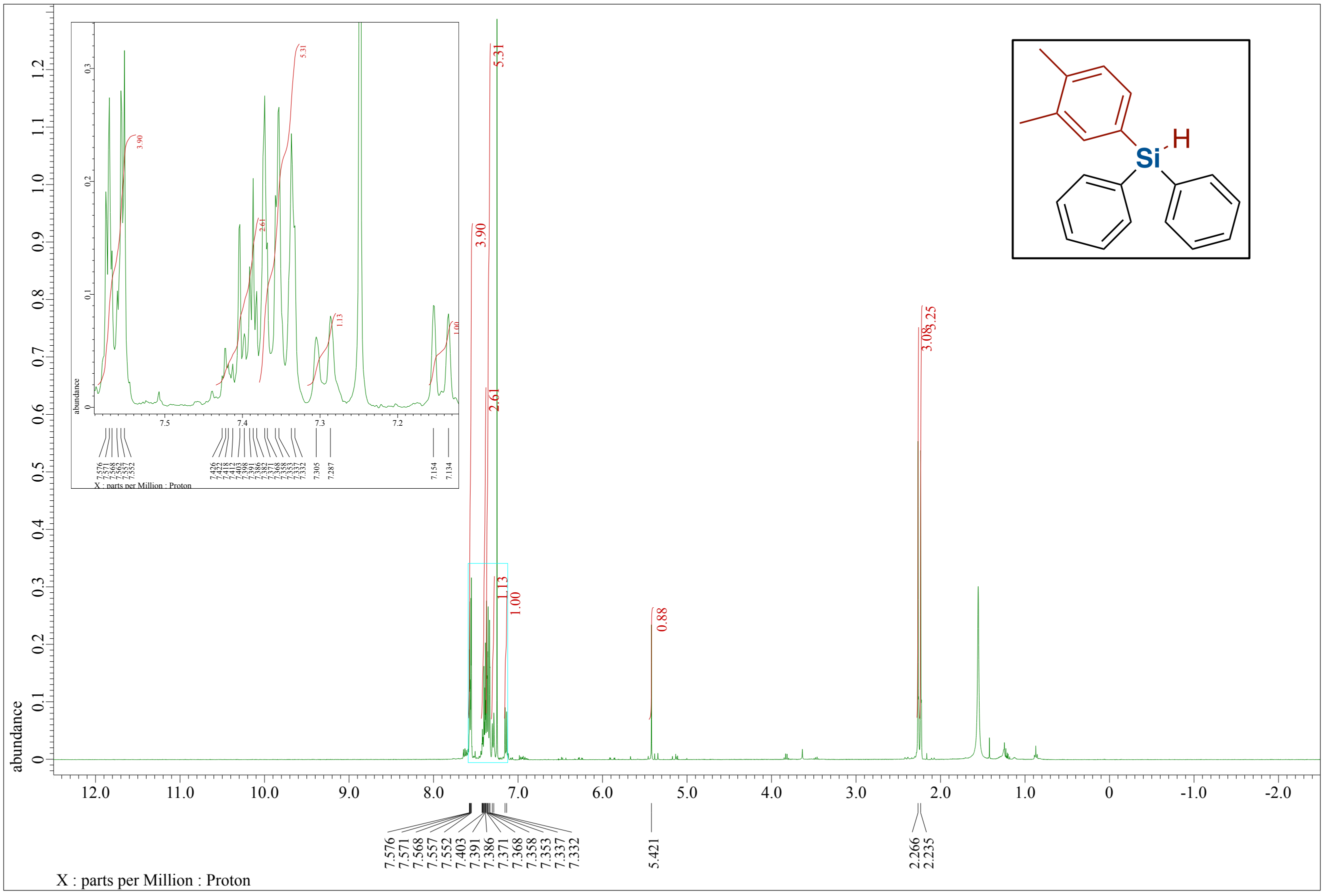

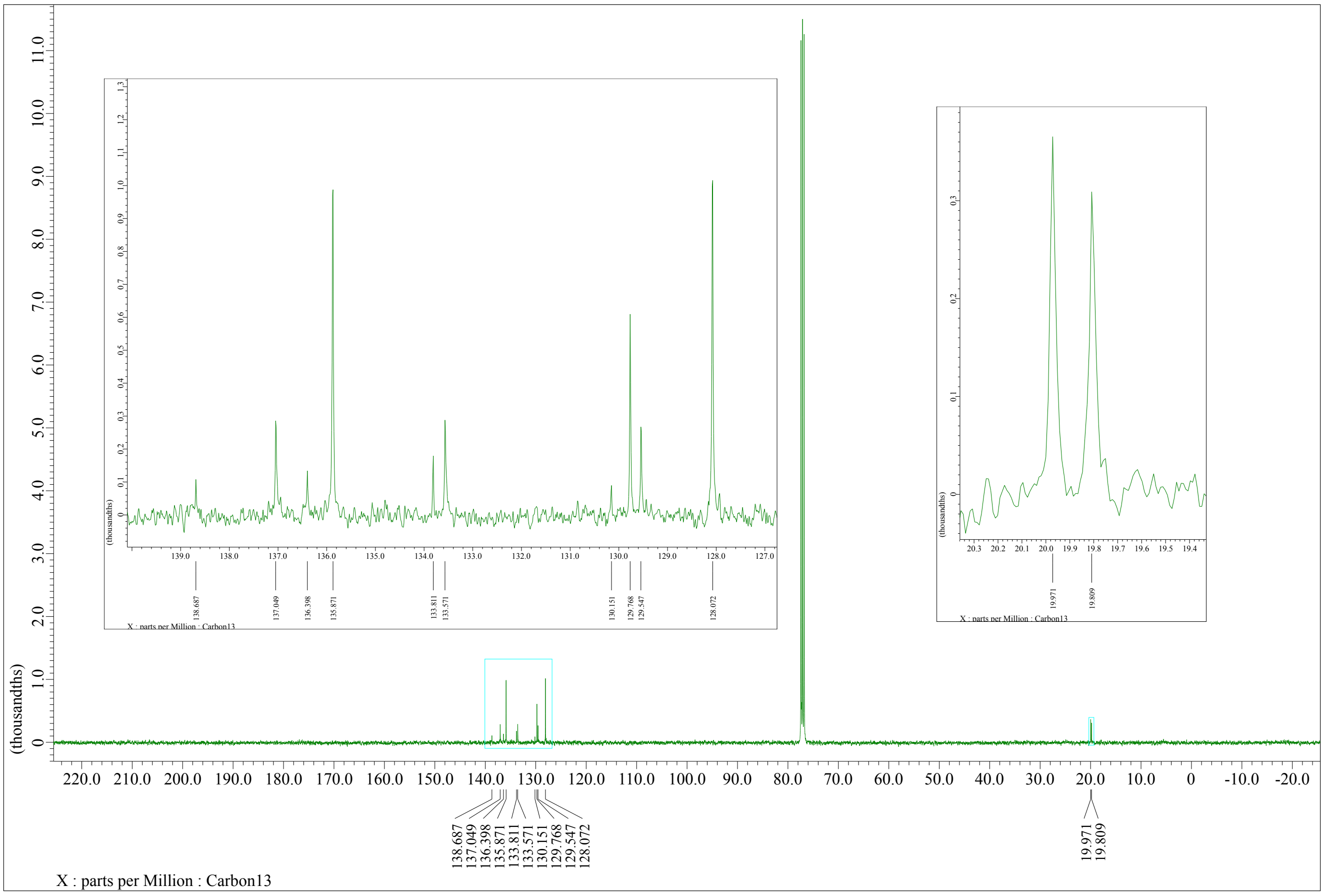

Figure S67. ^{1}H and ^{13}C NMR of **(3,4-dimethylphenyl)diphenylsilane.**

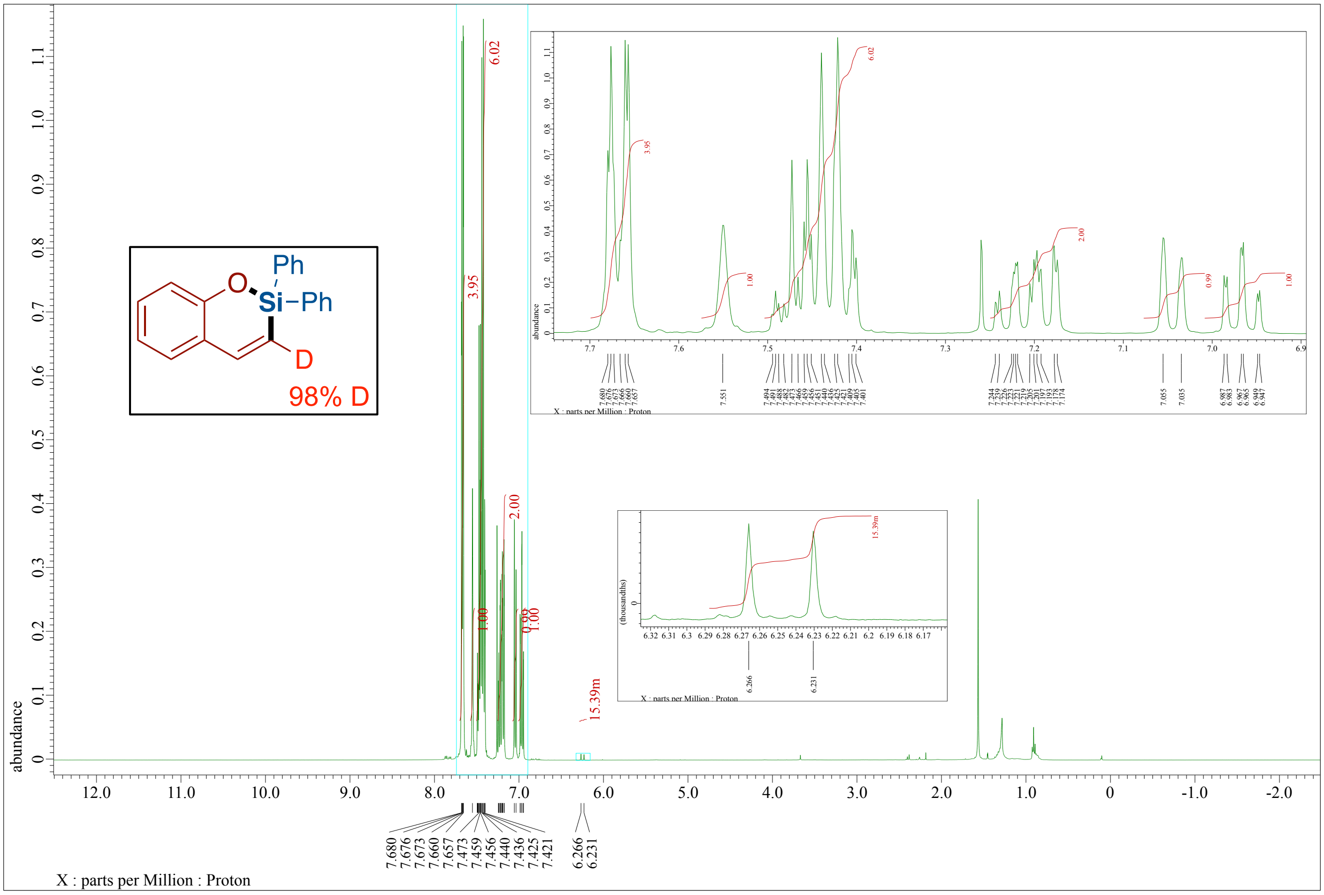

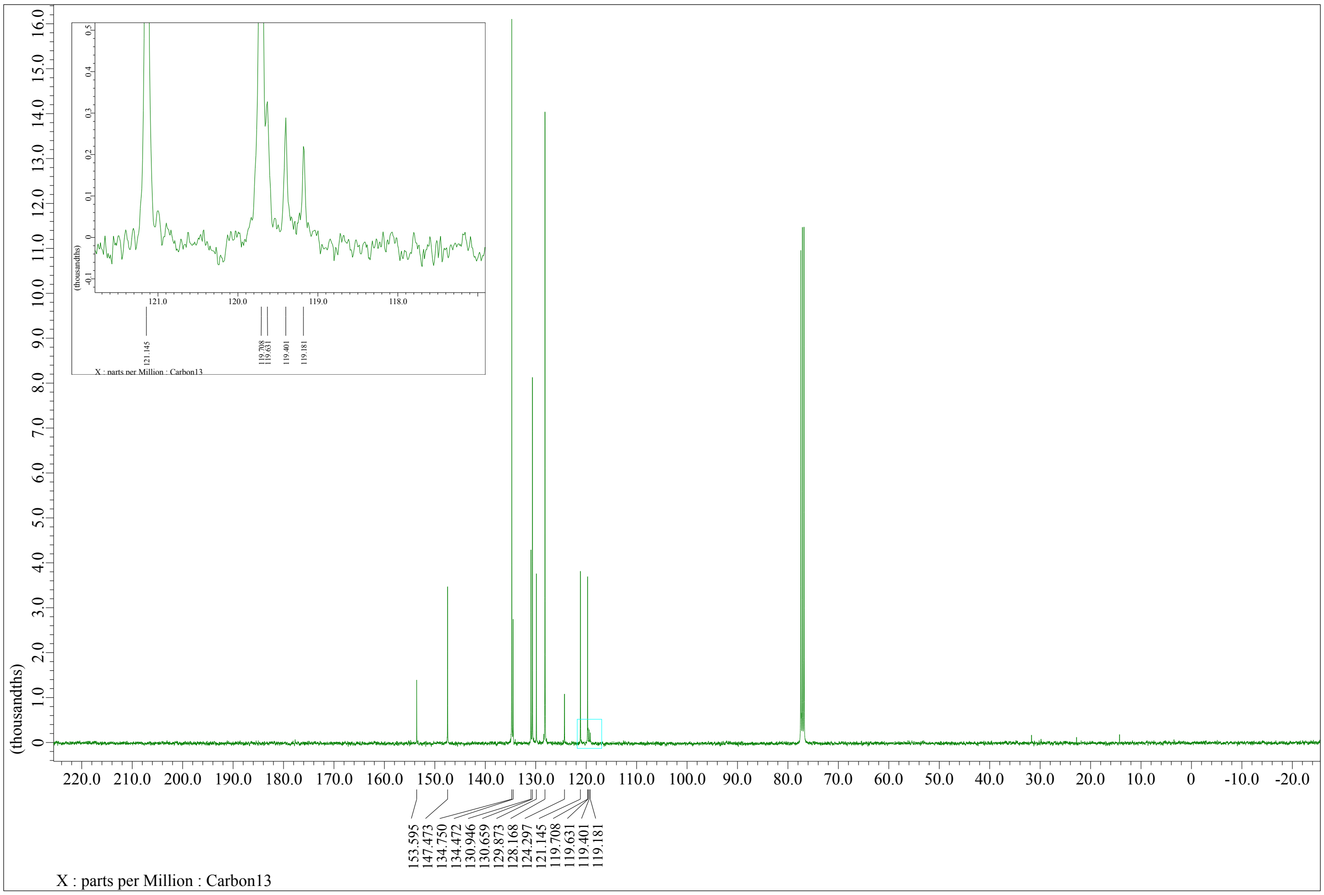

Figure S68. ^{1}H and ^{13}C NMR of **2aJ-*d***.

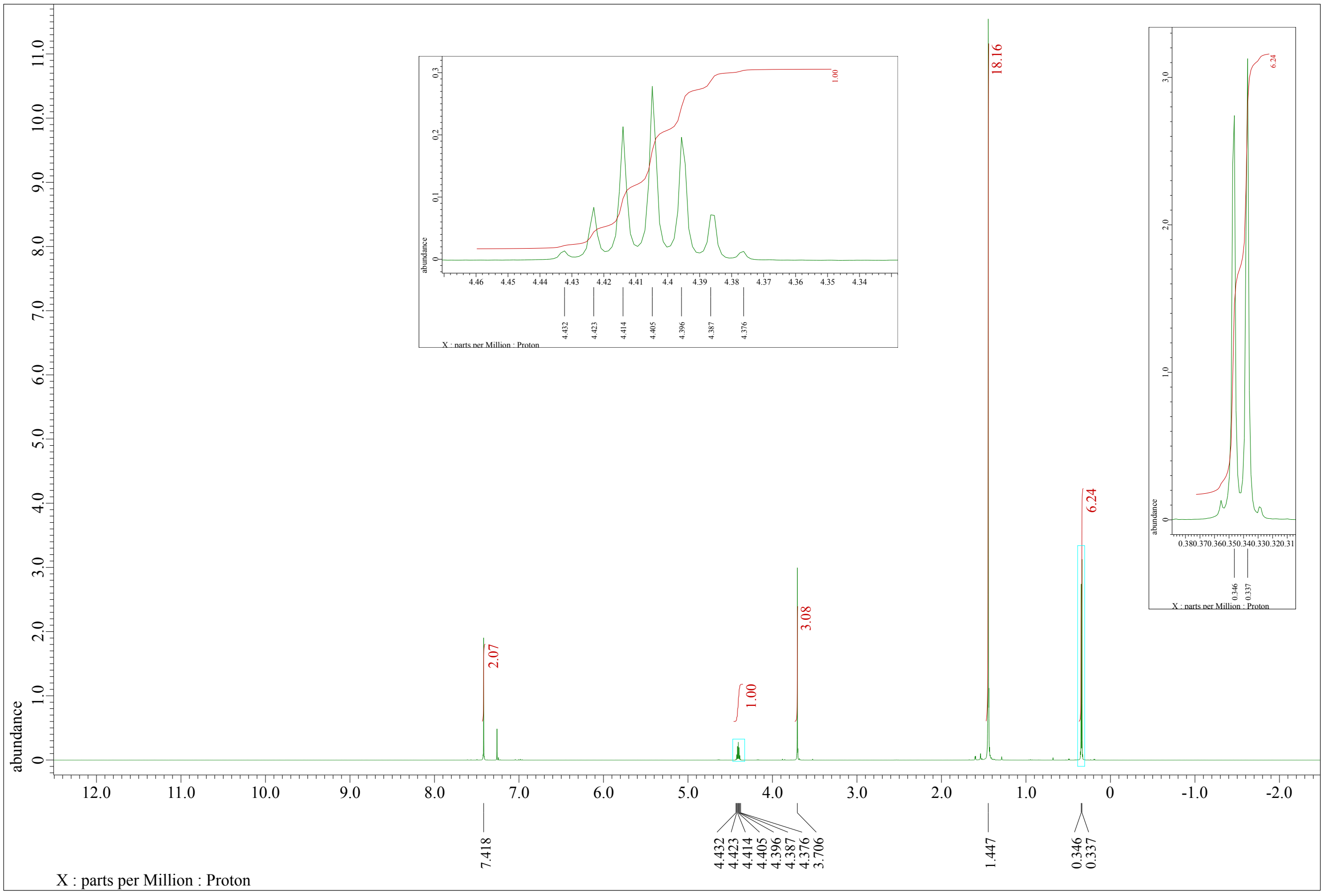

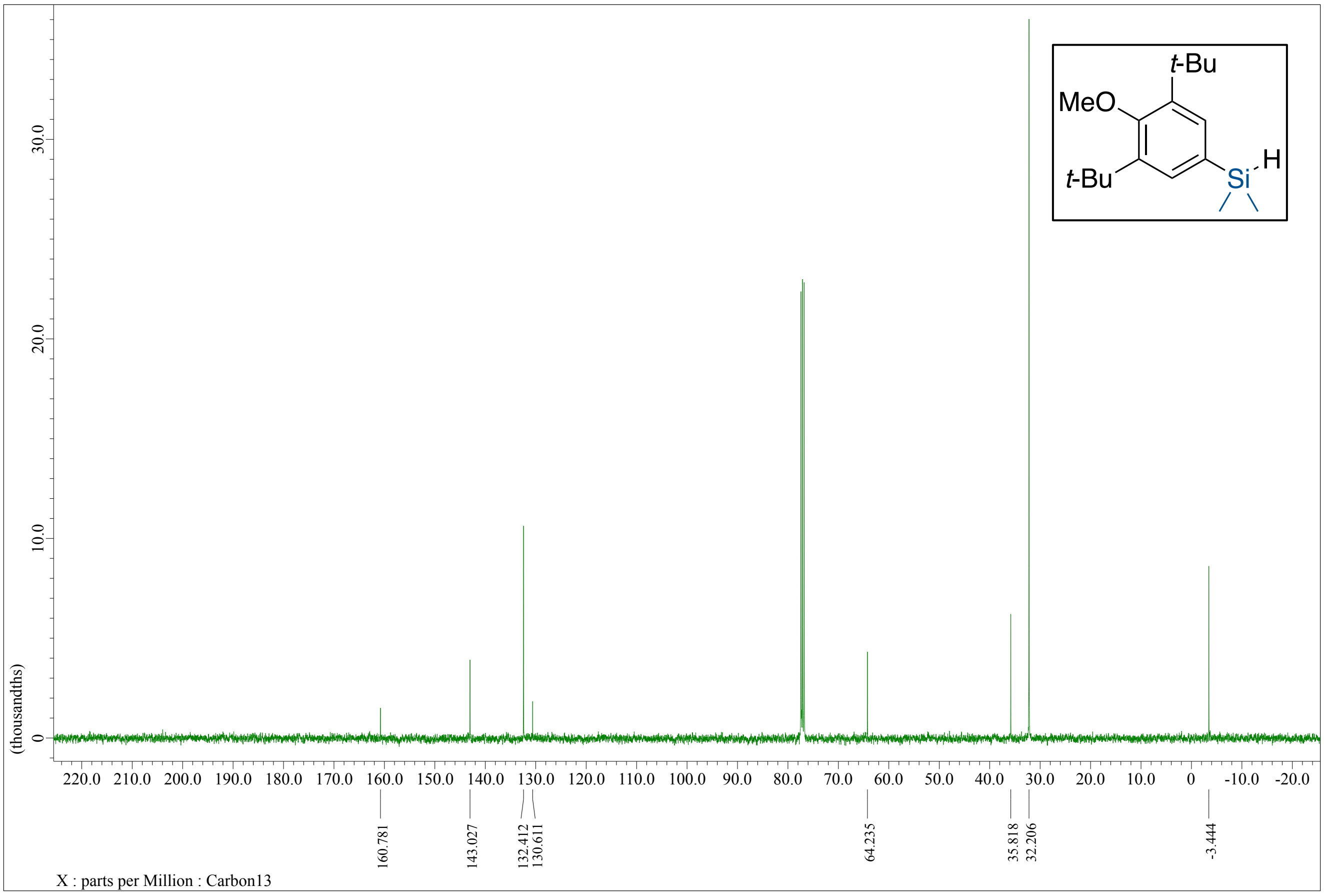

Figure S69. ^{1}H and ^{13}C NMR of **A11**.

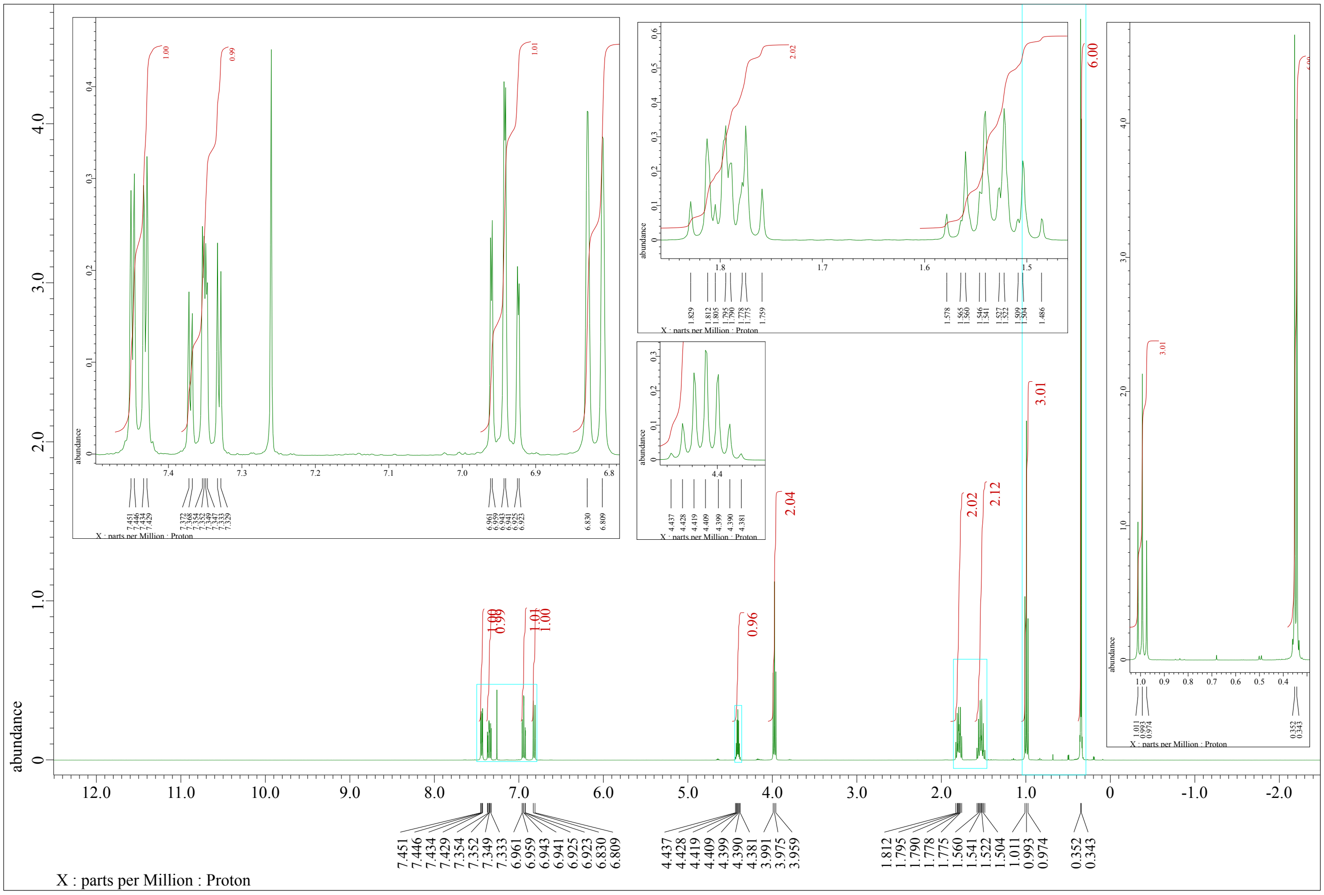

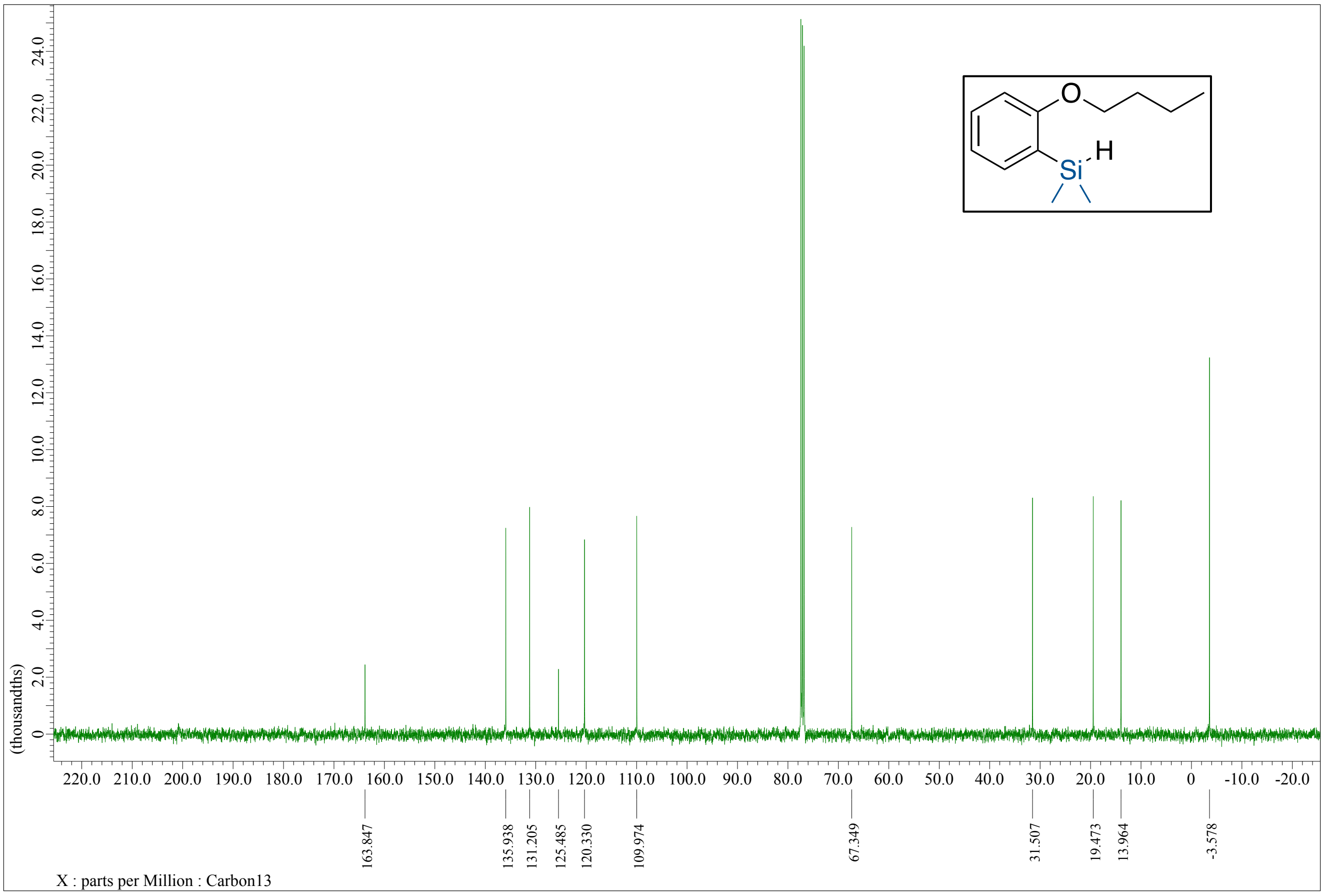

Figure S70. ^{1}H and ^{13}C NMR of **A15**.

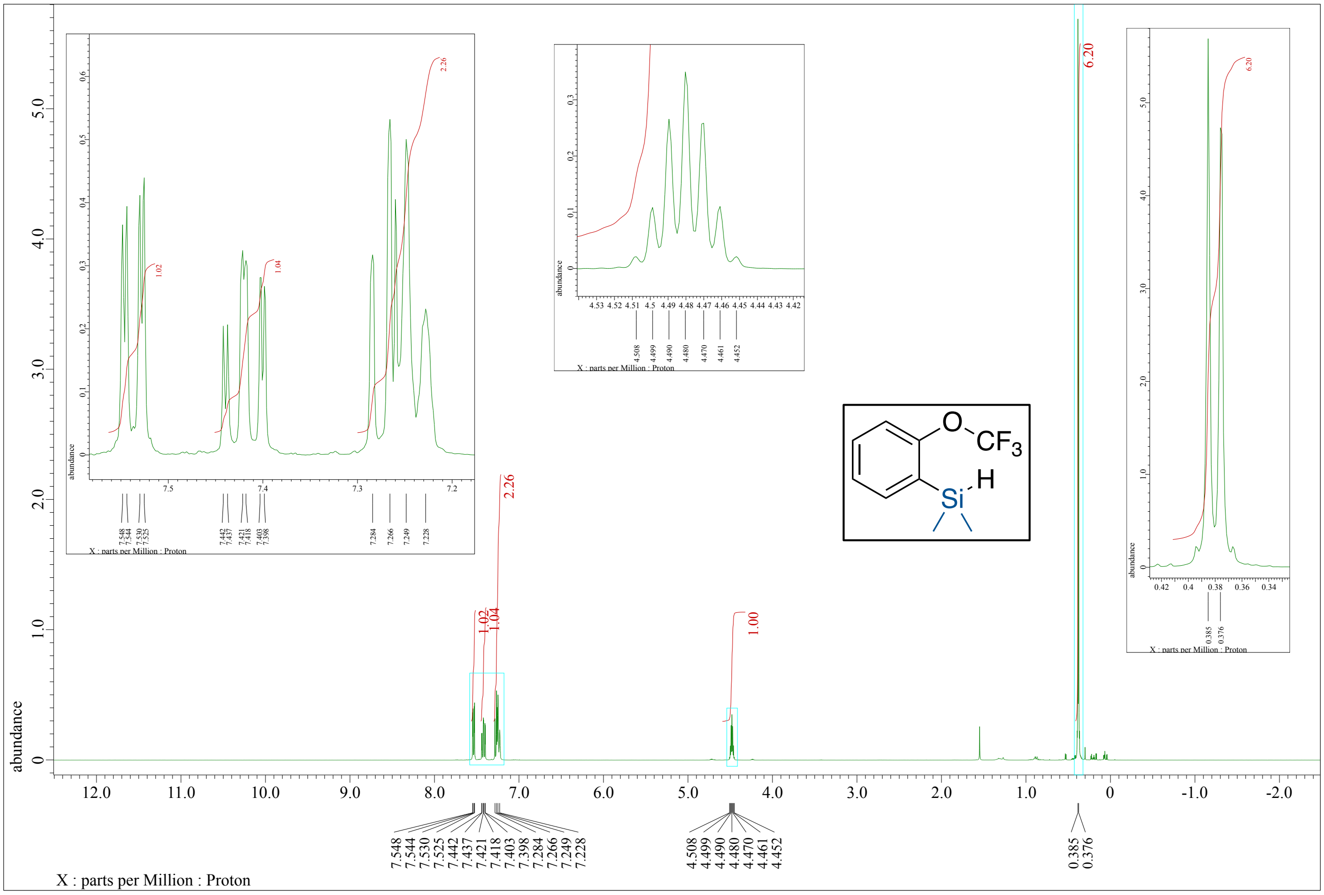

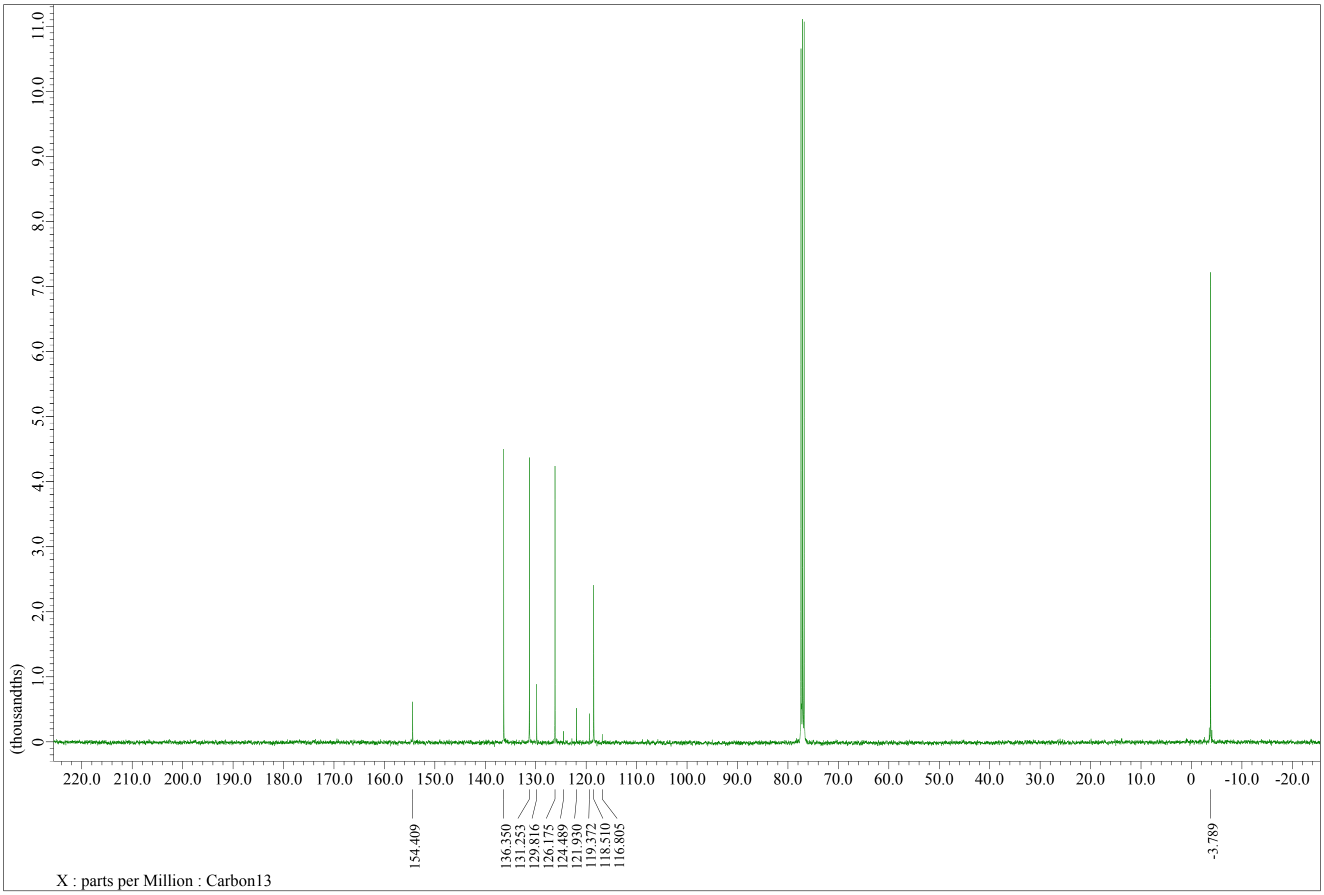

Figure S71. ^{1}H and ^{13}C NMR of **A16**.

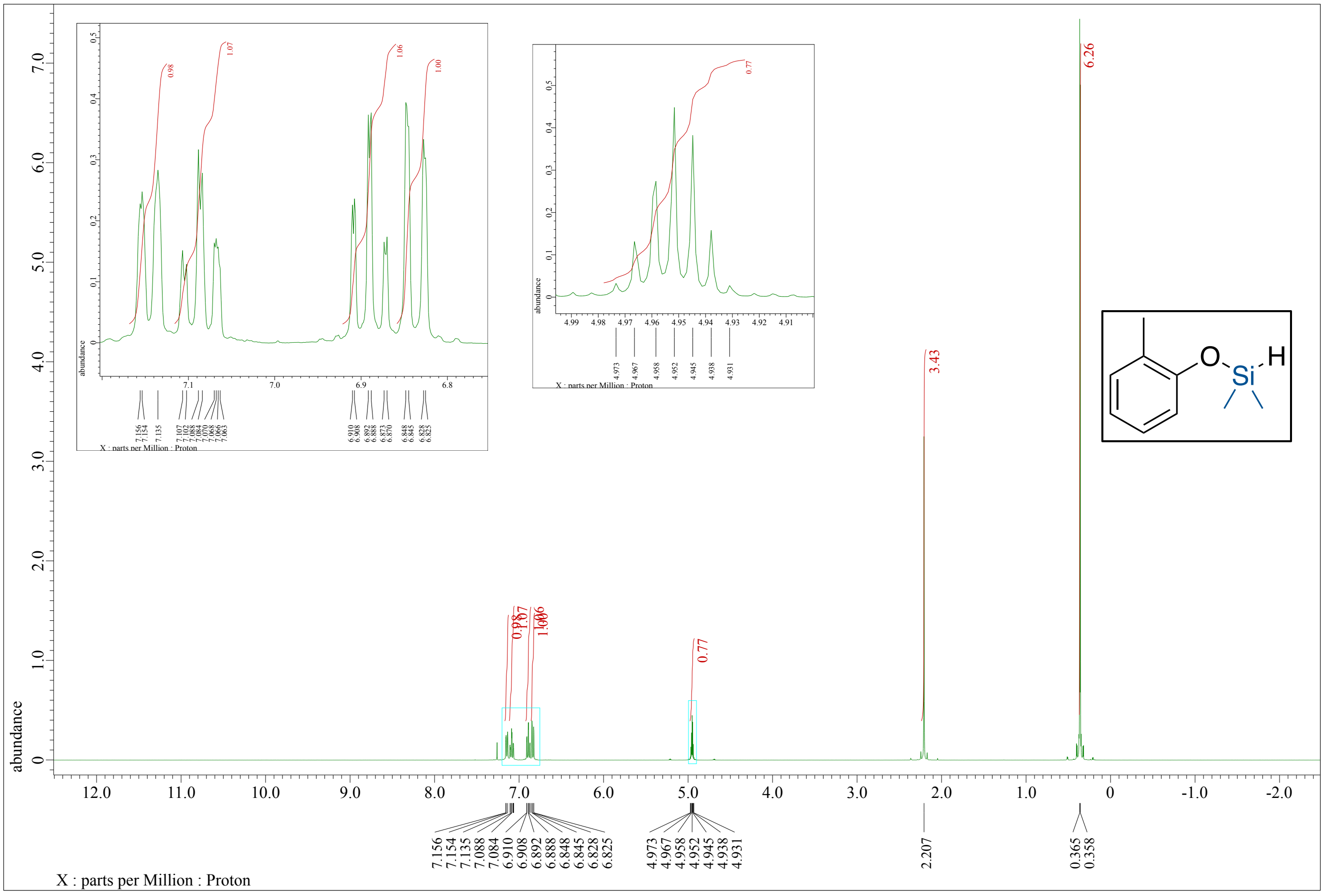

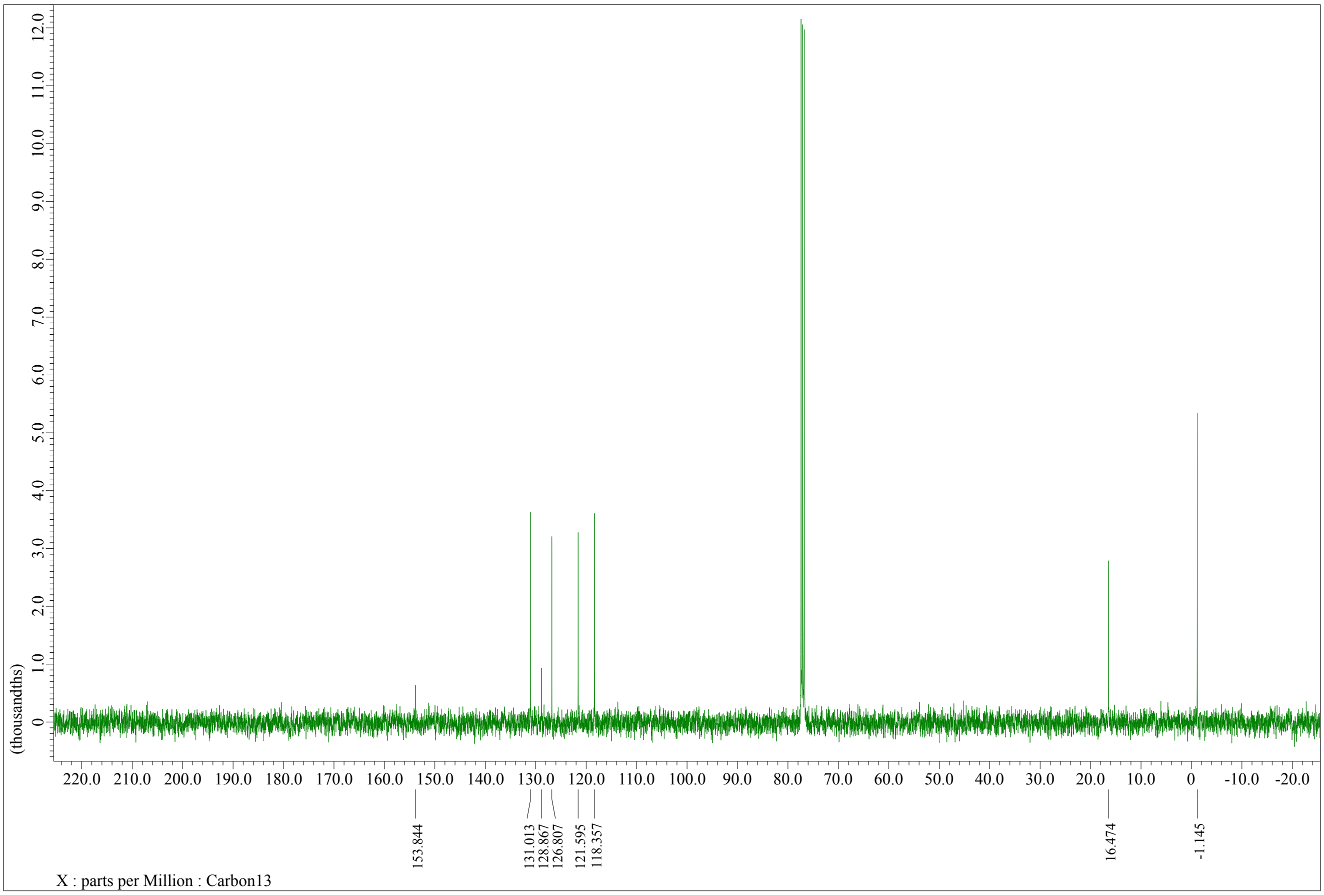

Figure S72. ^{1}H and ^{13}C NMR of **A19**.

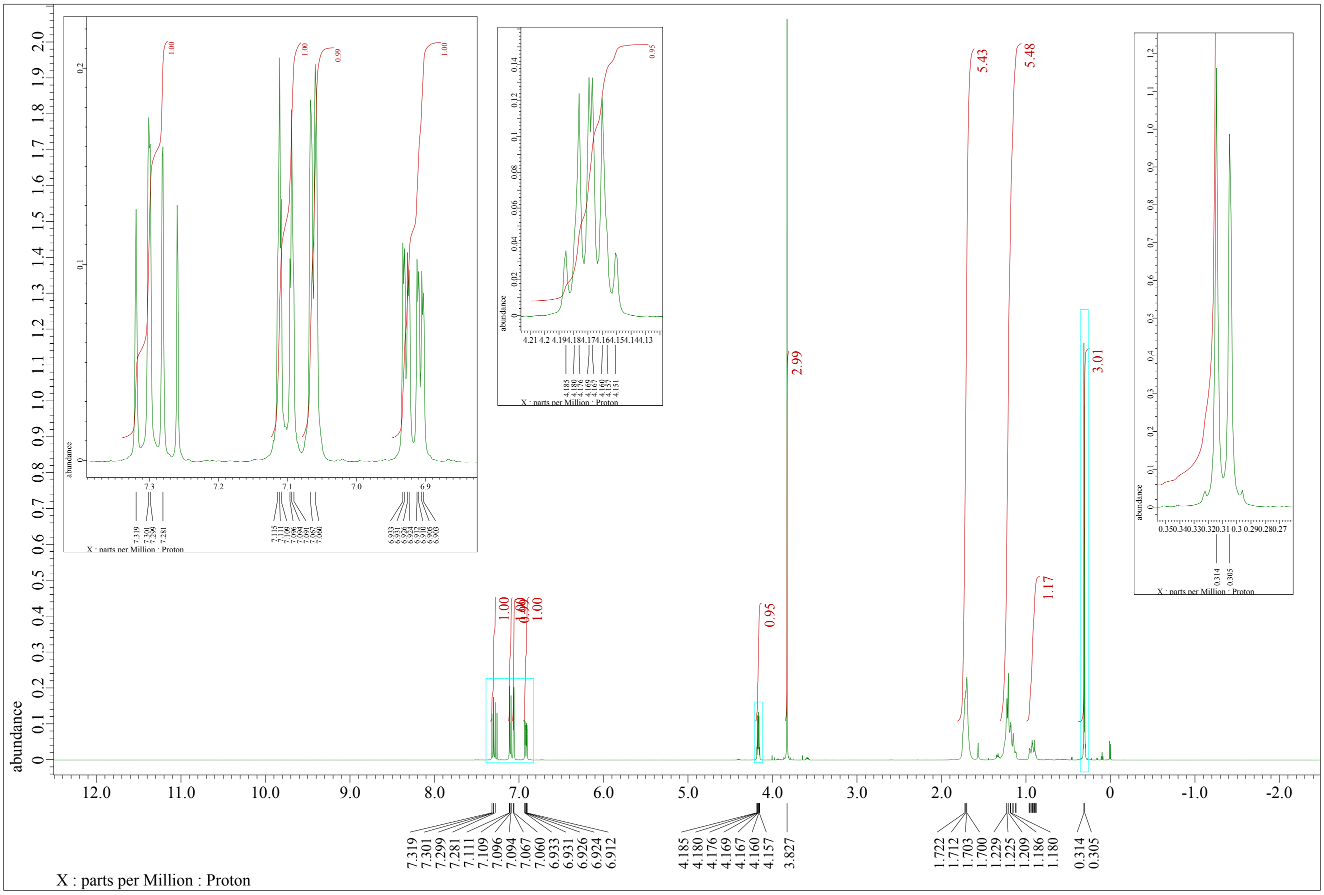

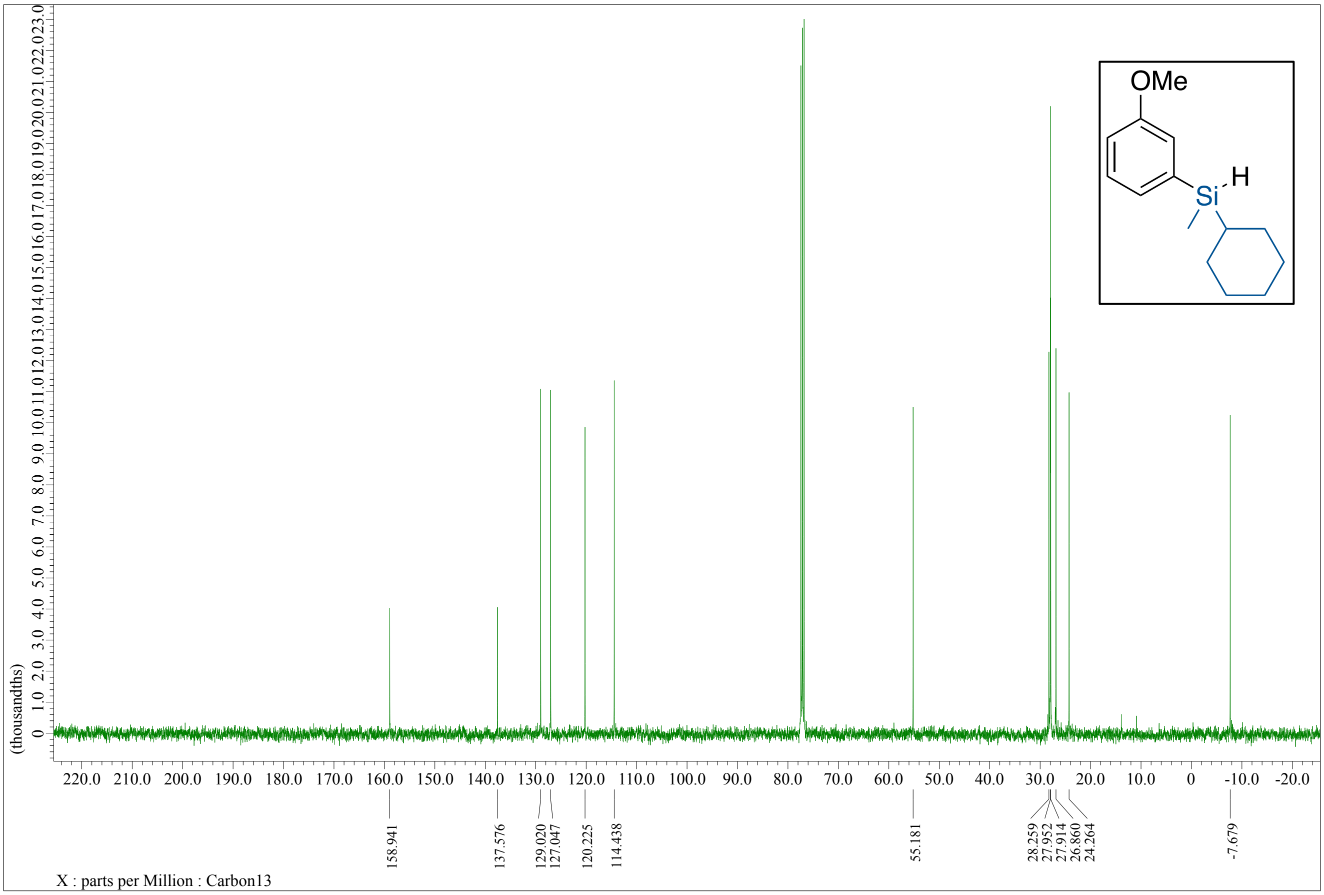

Figure S73. ^{1}H and ^{13}C NMR of **B**.

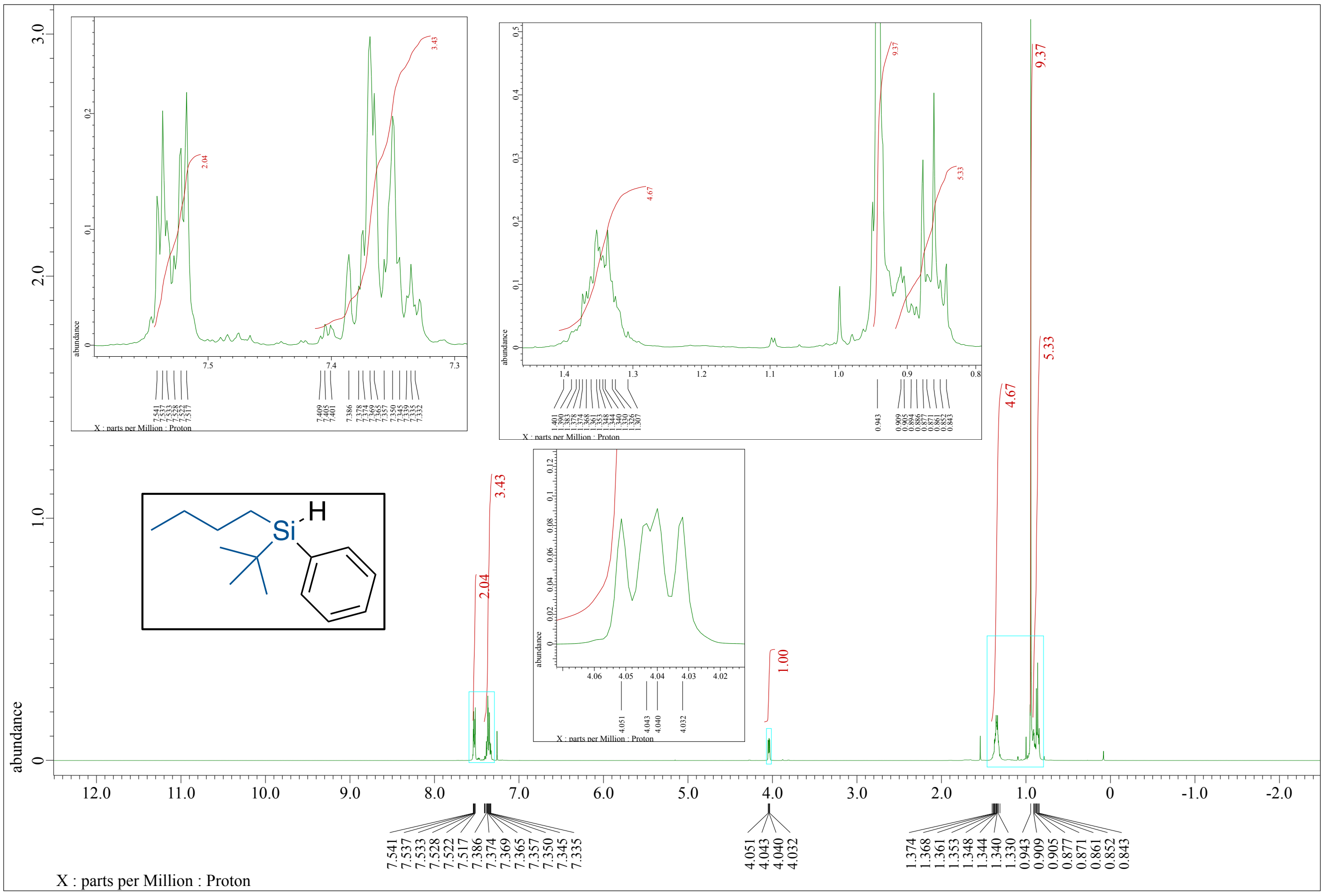

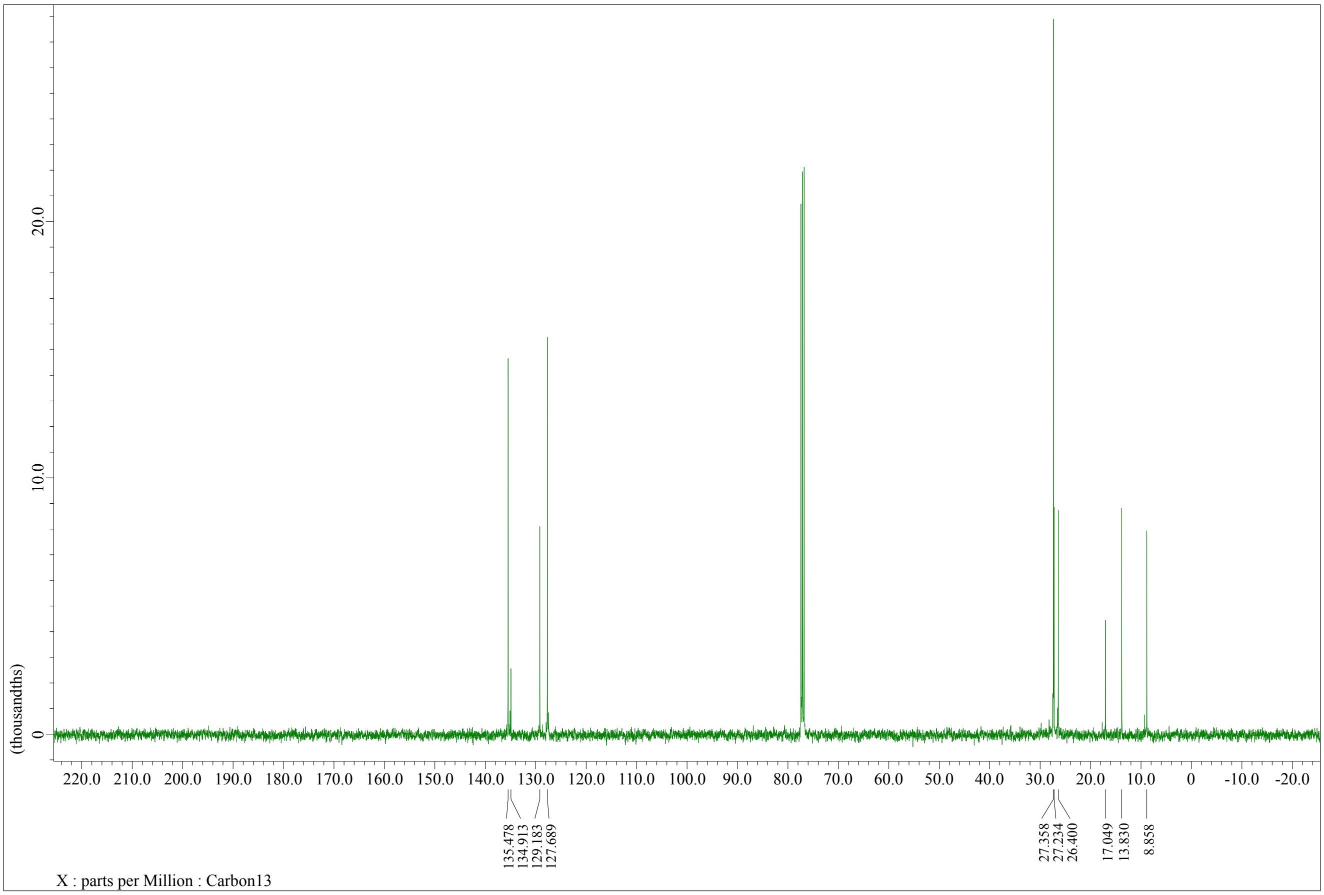

Figure S74. ^{1}H and ^{13}C NMR of **C**.

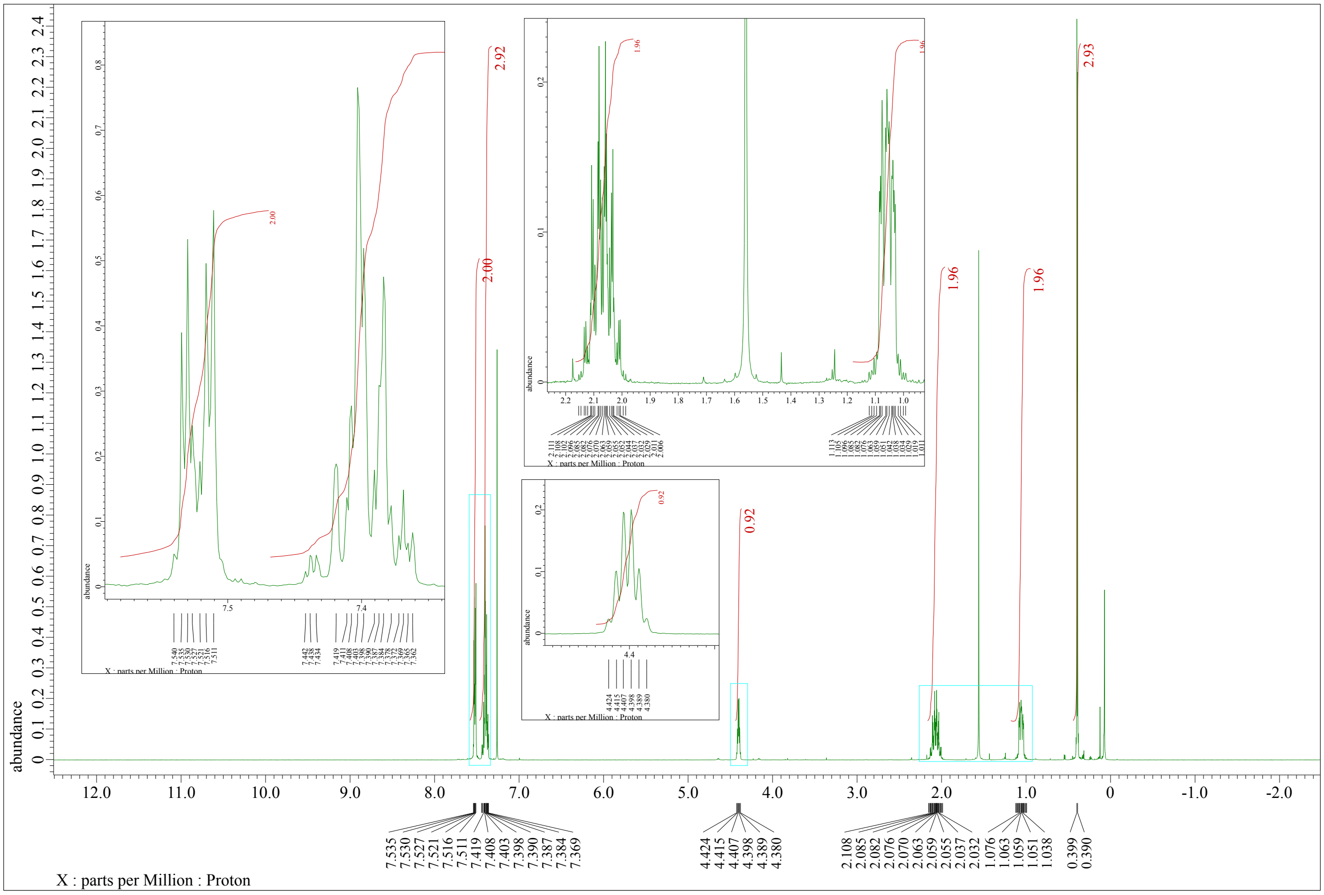

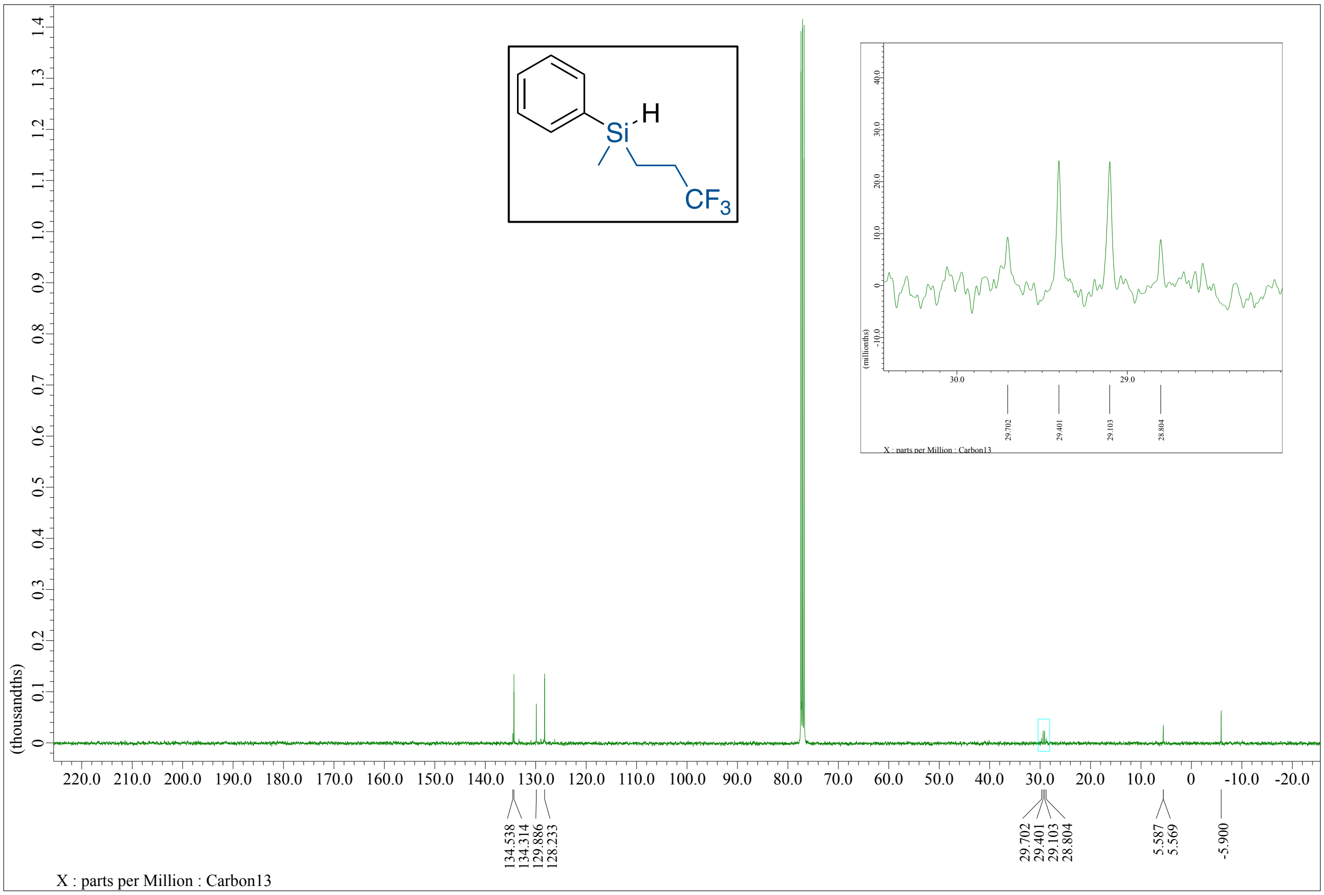

Figure S75. ^{1}H and ^{13}C NMR of **D**.

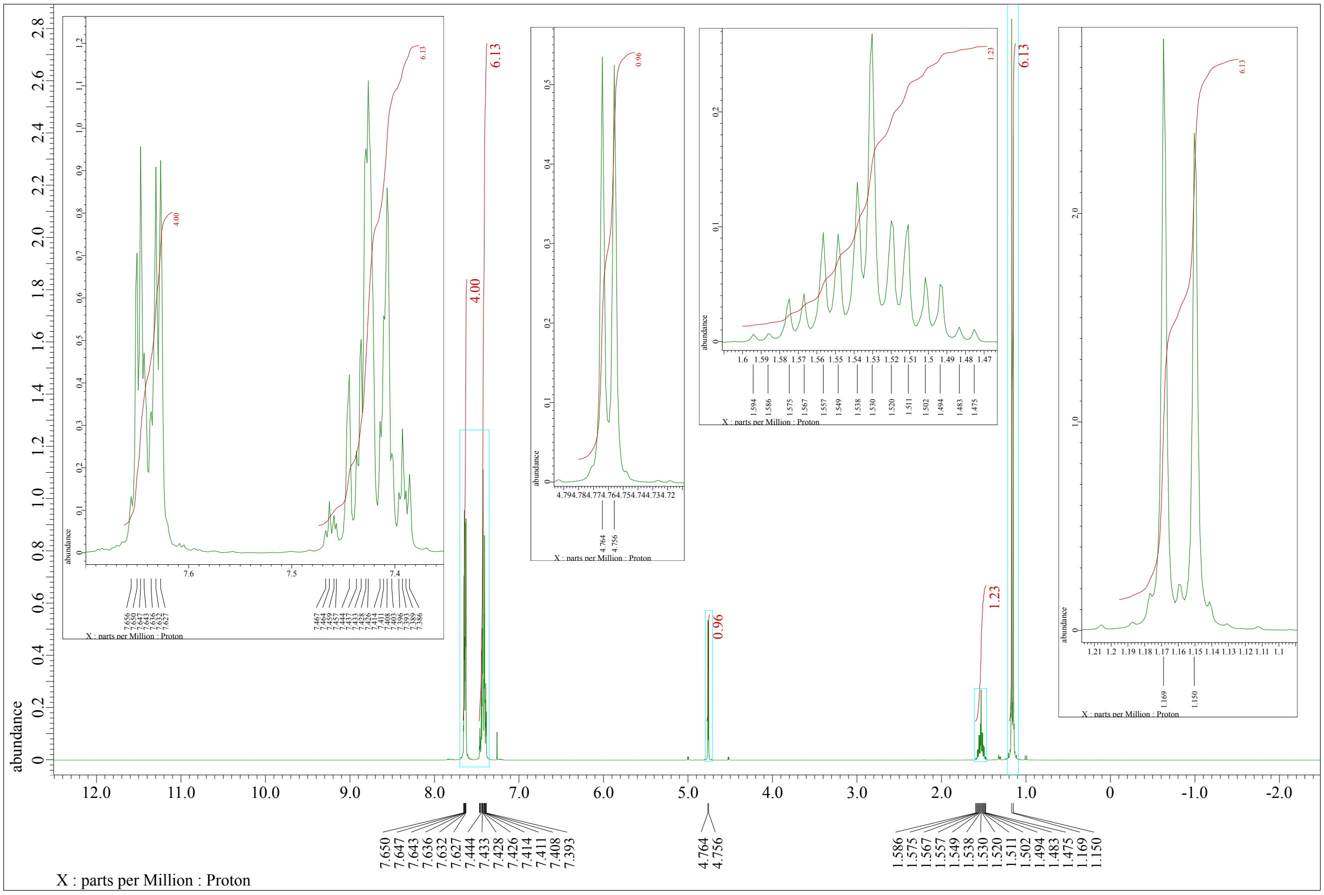

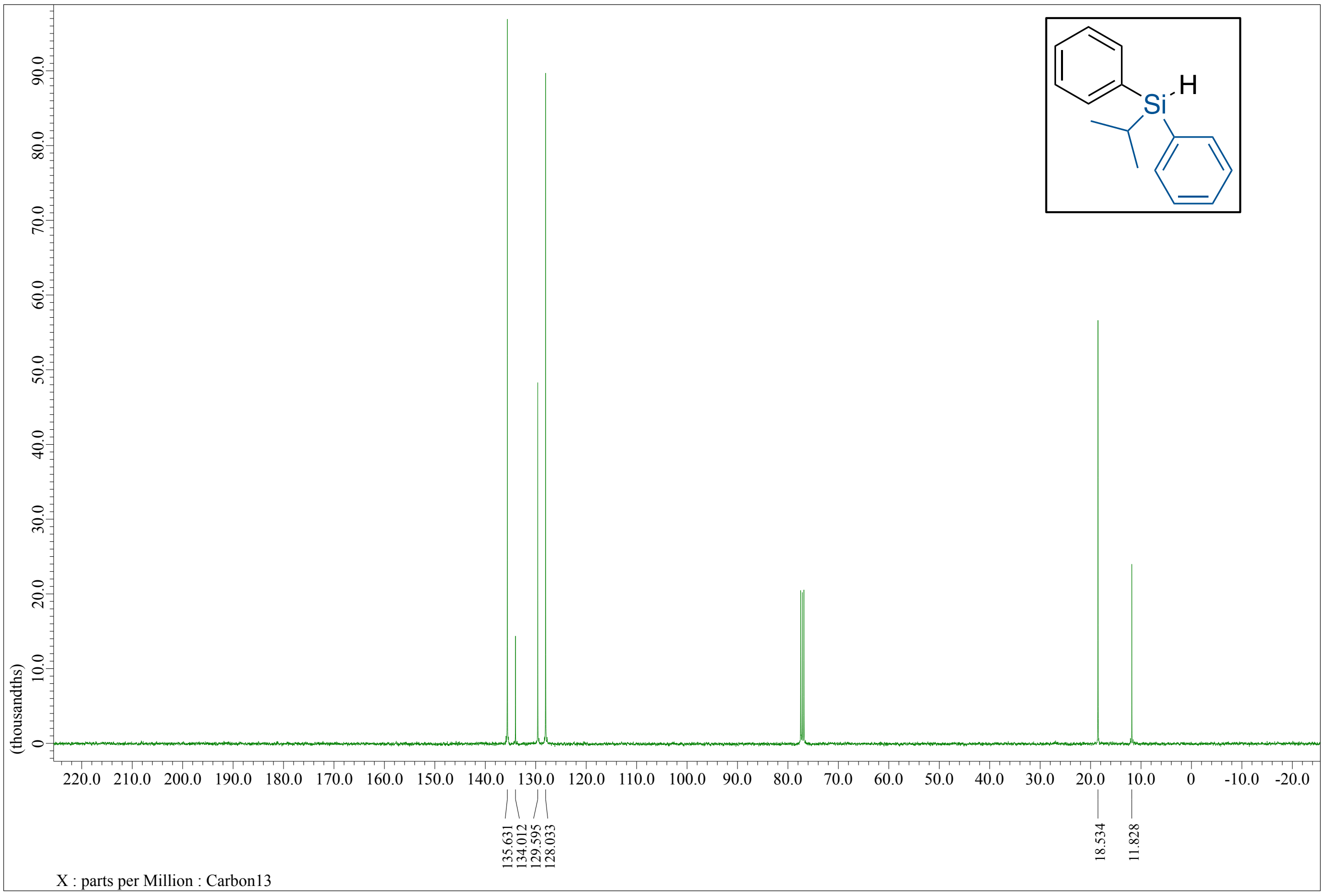

Figure S76. ^{1}H and ^{13}C NMR of **F**.

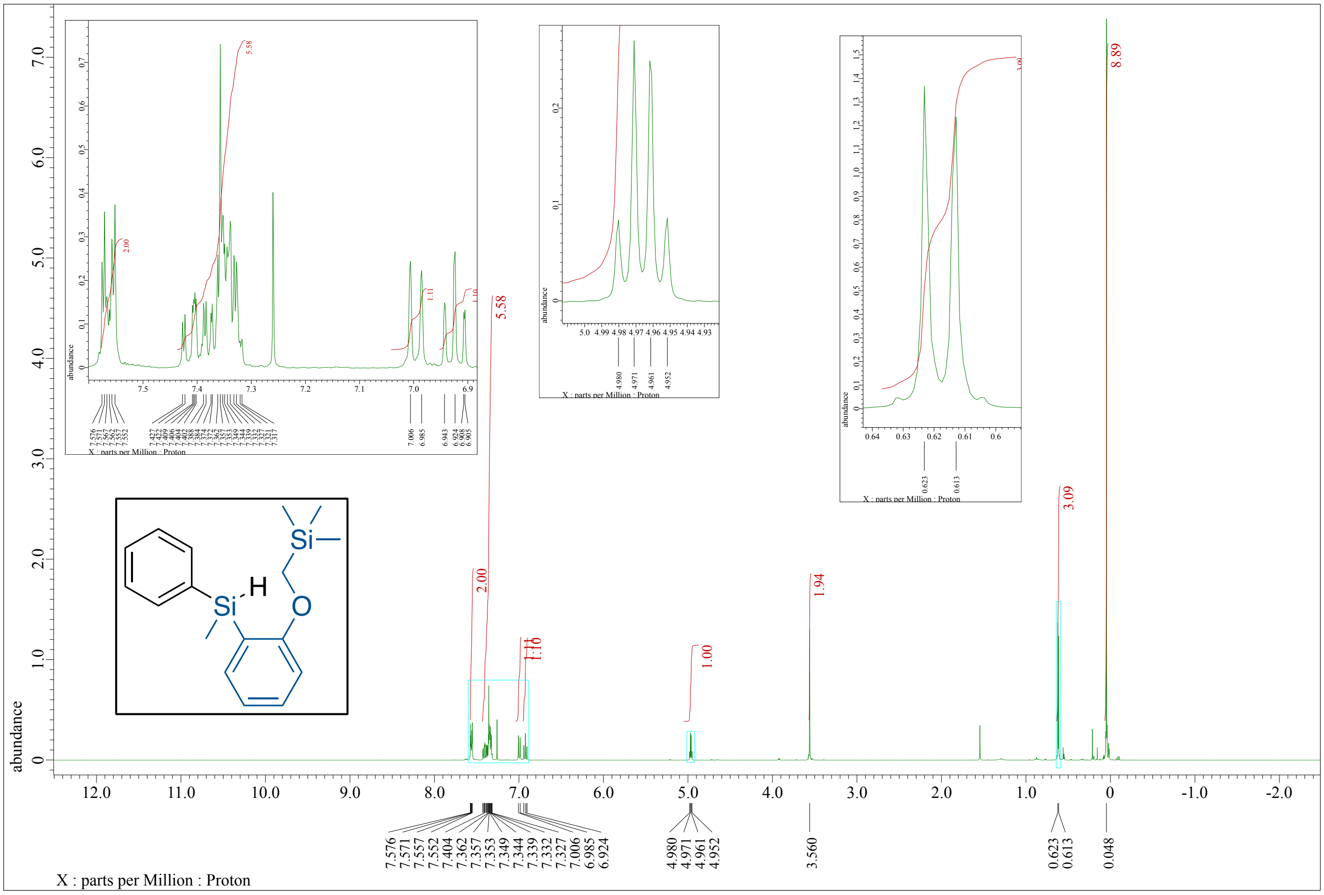

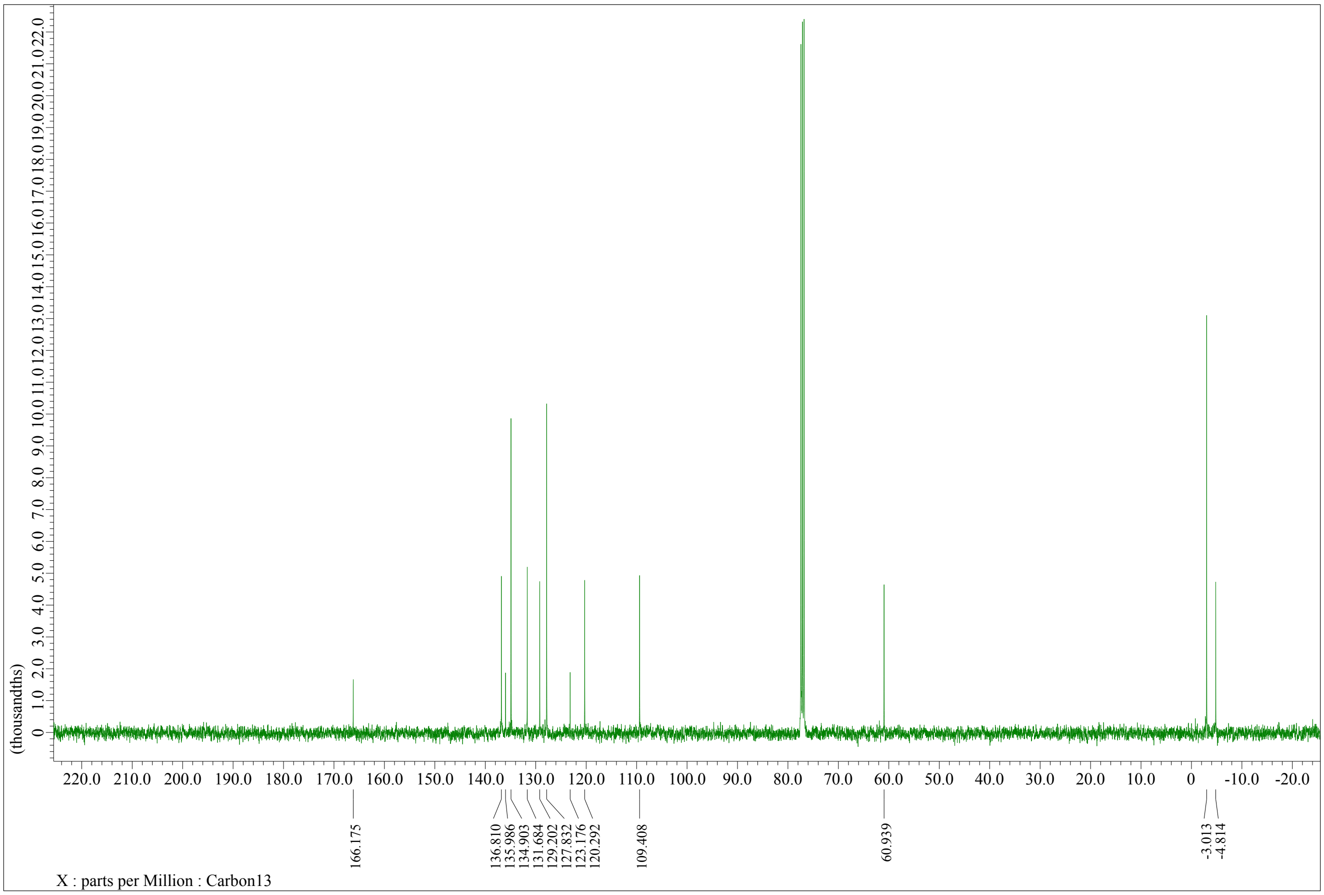

Figure S77. ^{1}H and ^{13}C NMR of **H**.

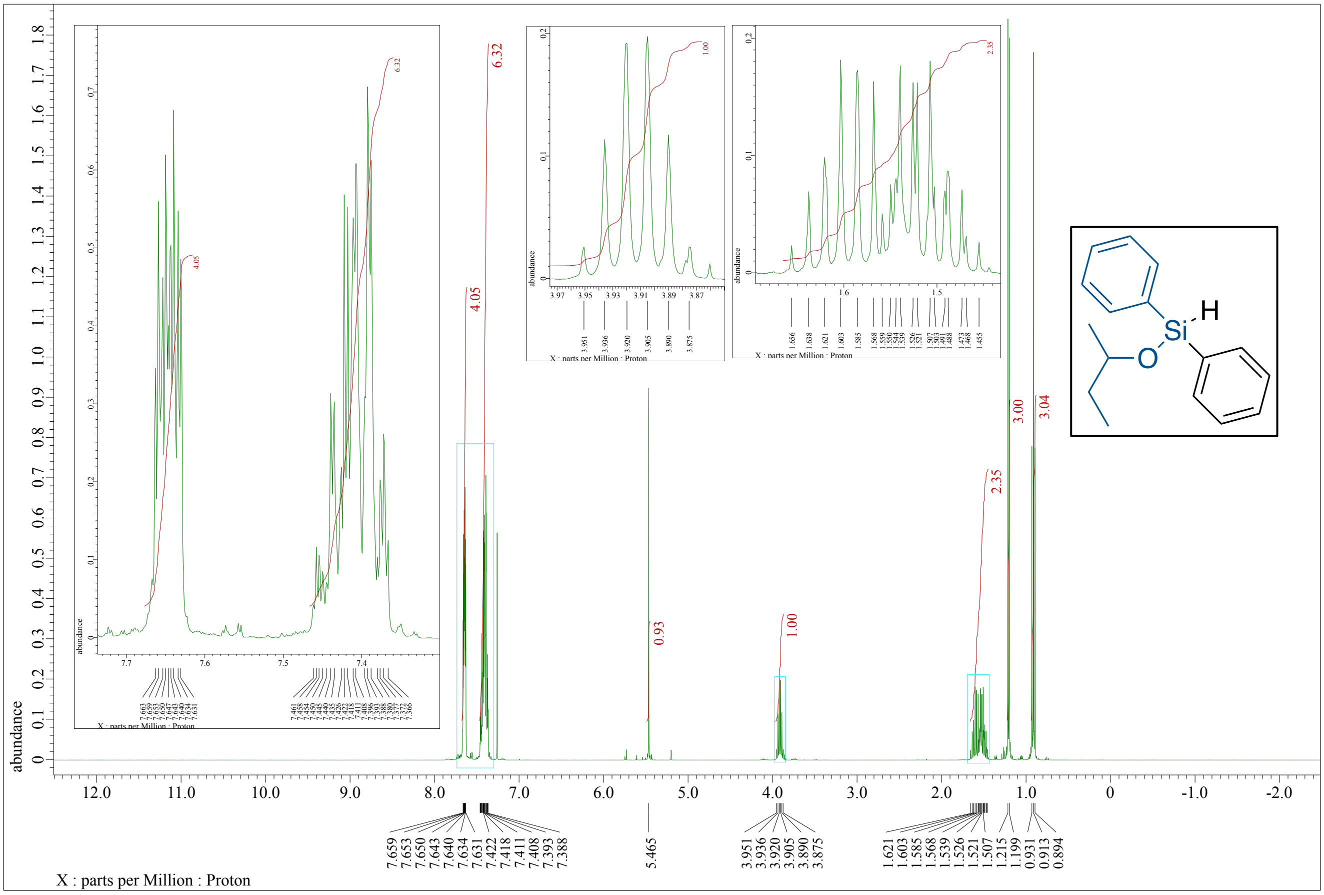

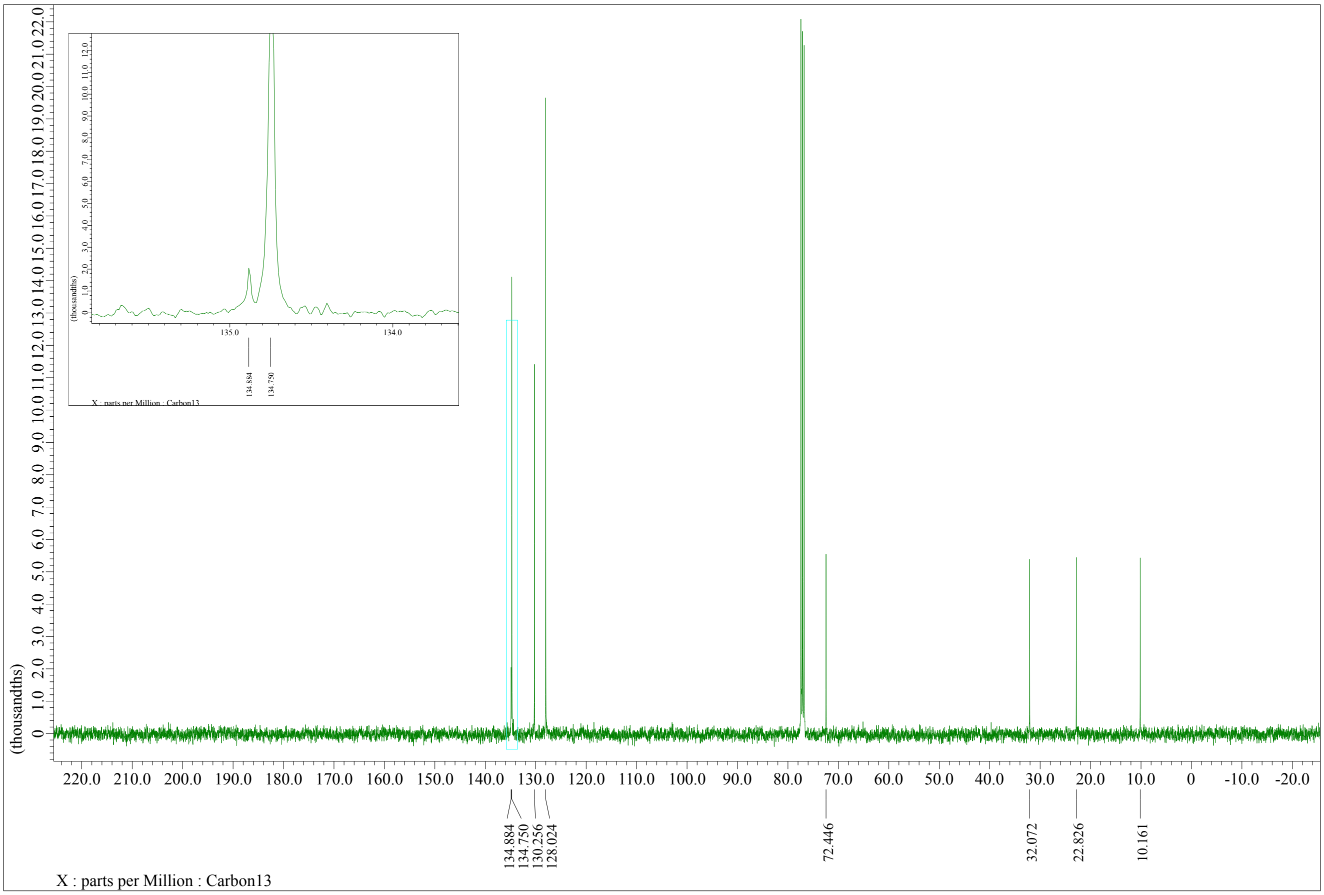

Figure S78. ^{1}H and ^{13}C NMR of **I**.

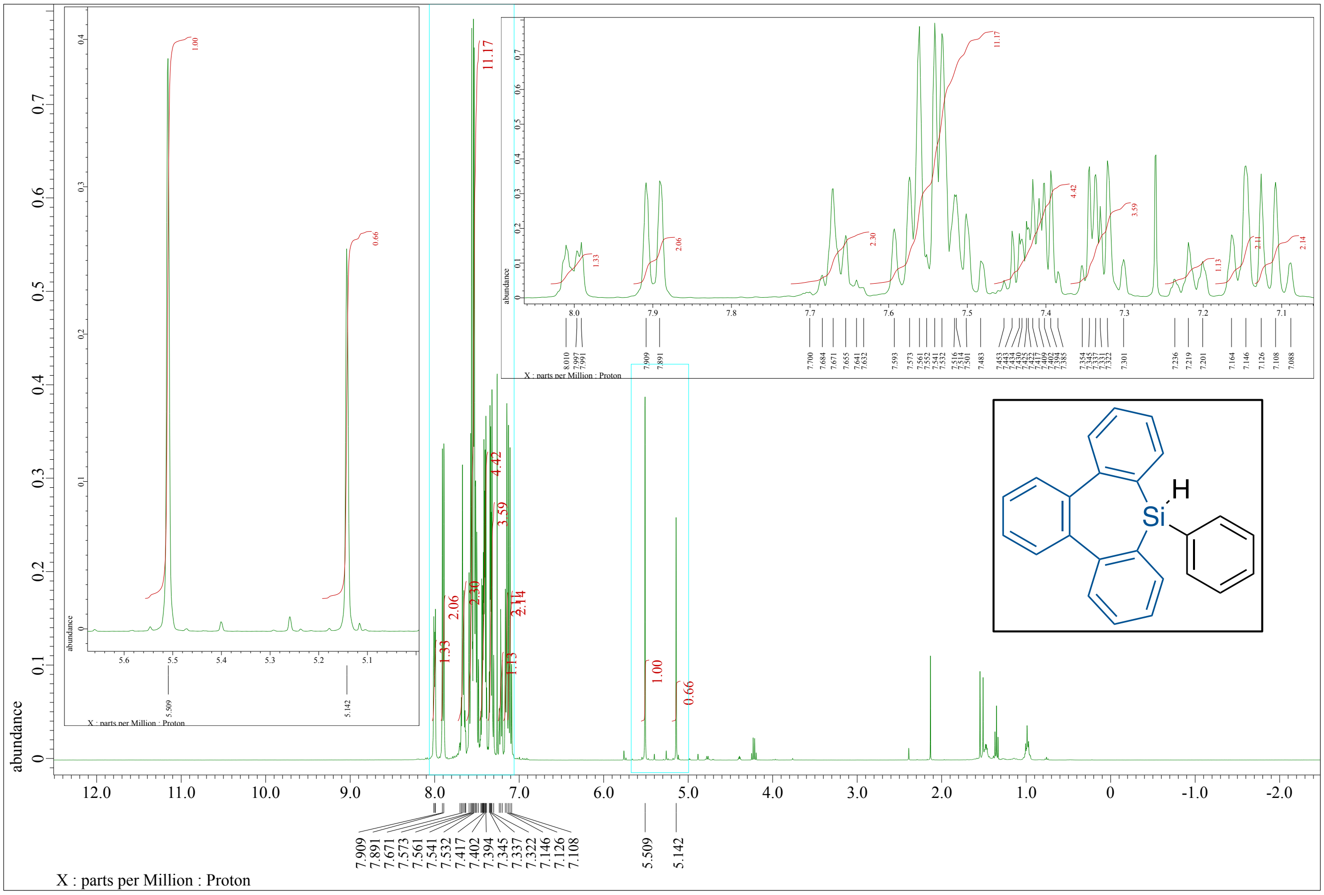

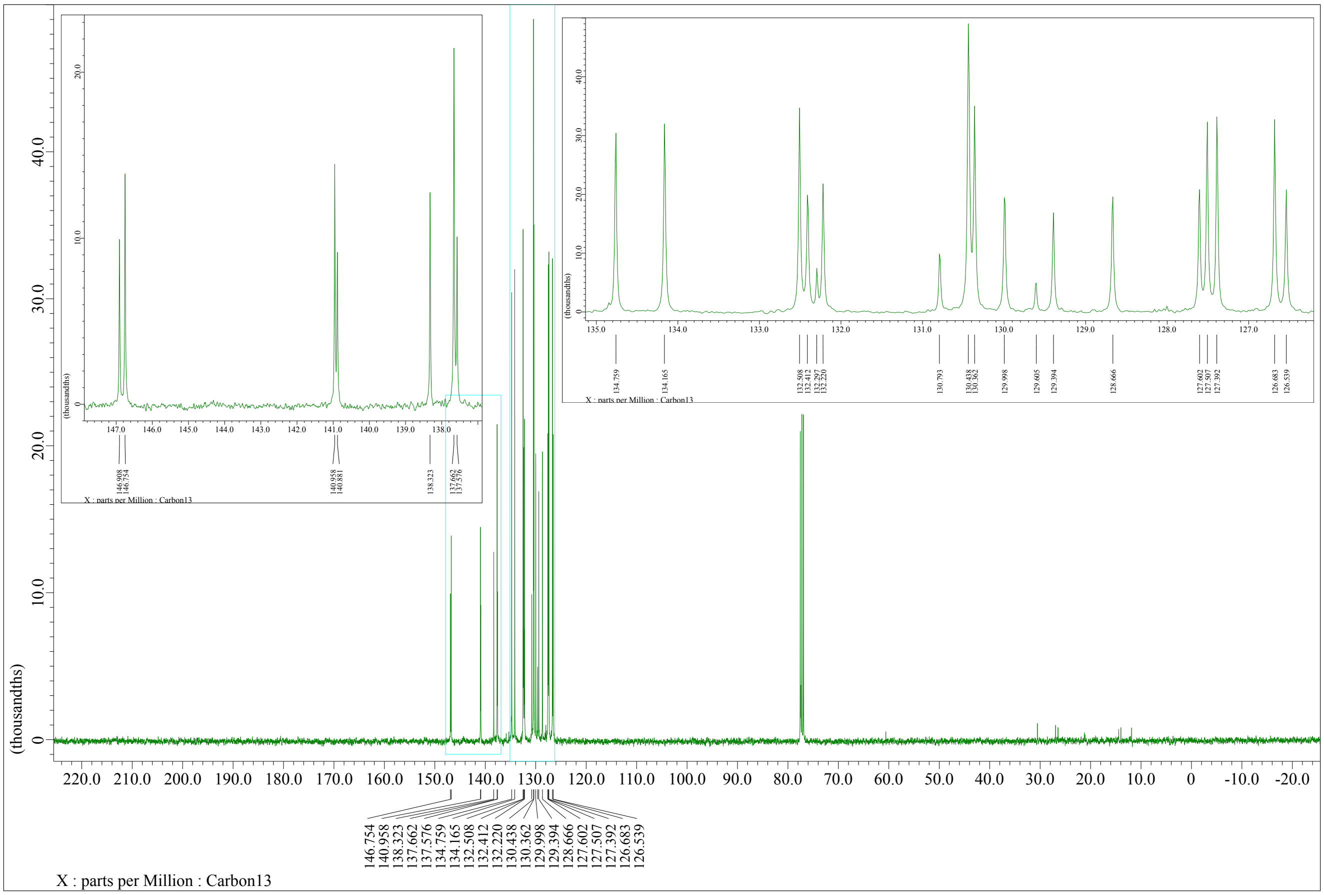

Figure S79. ^{1}H and ^{13}C NMR of **L**.

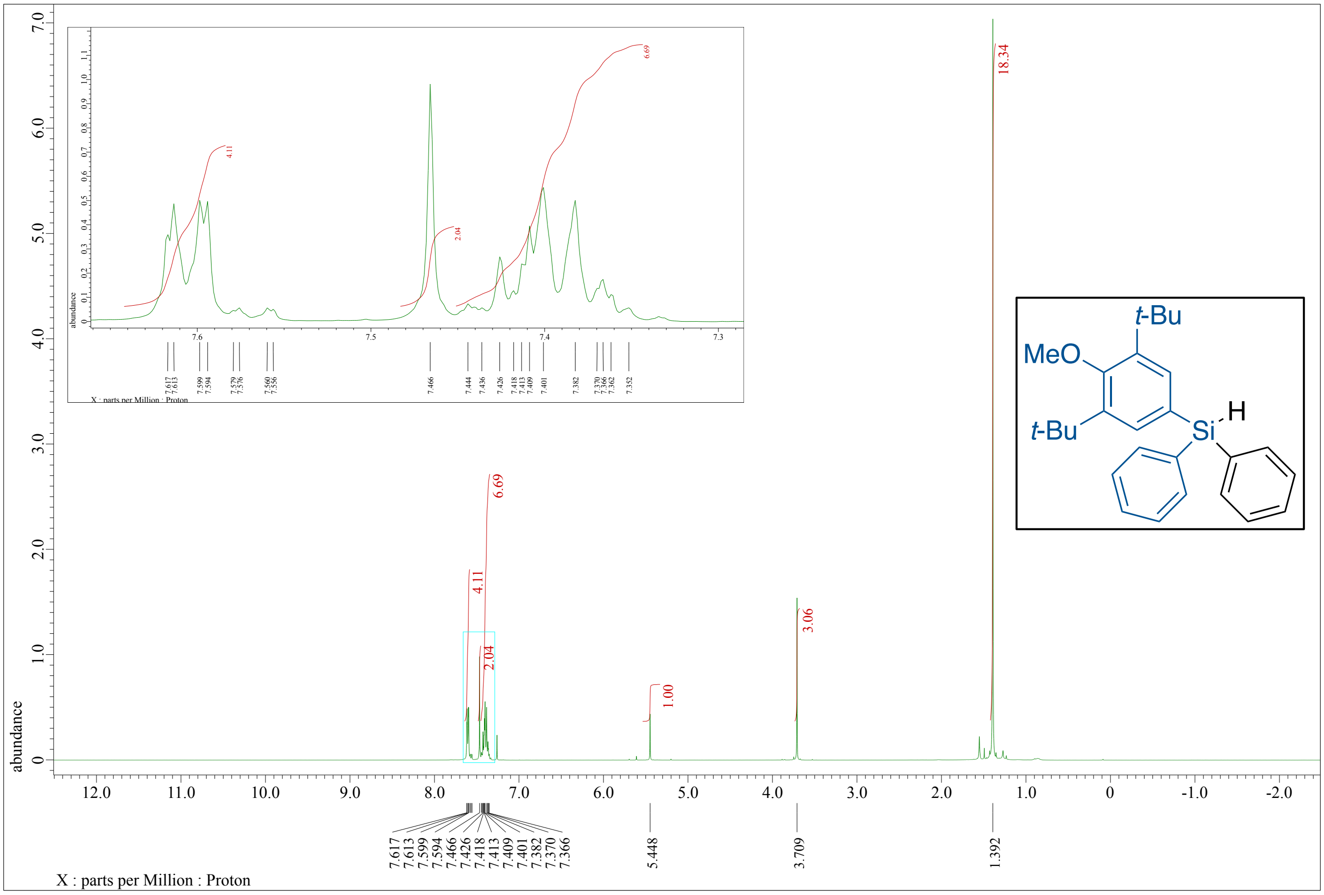

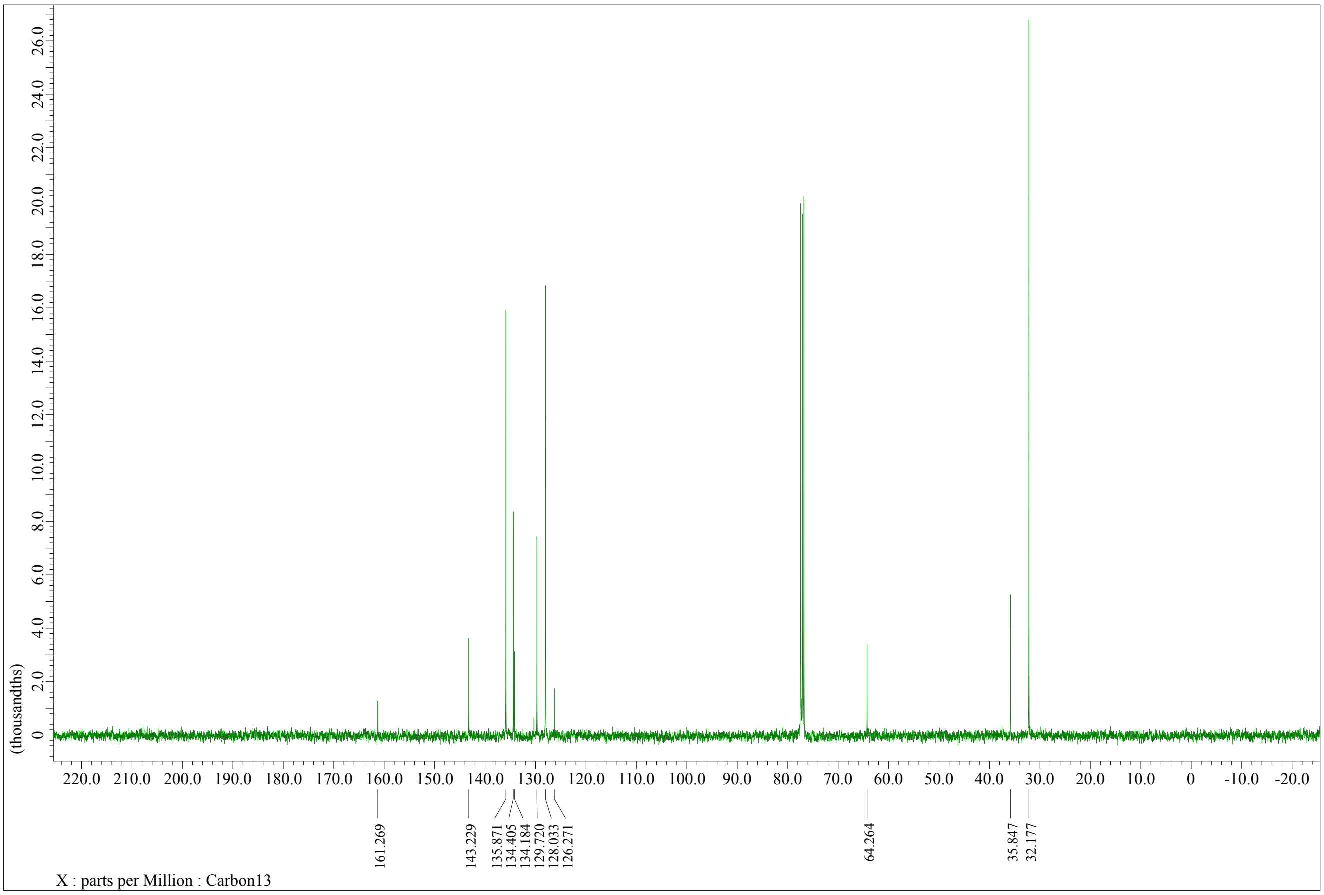

Figure S80. ^1H and ^{13}C NMR of **M**.

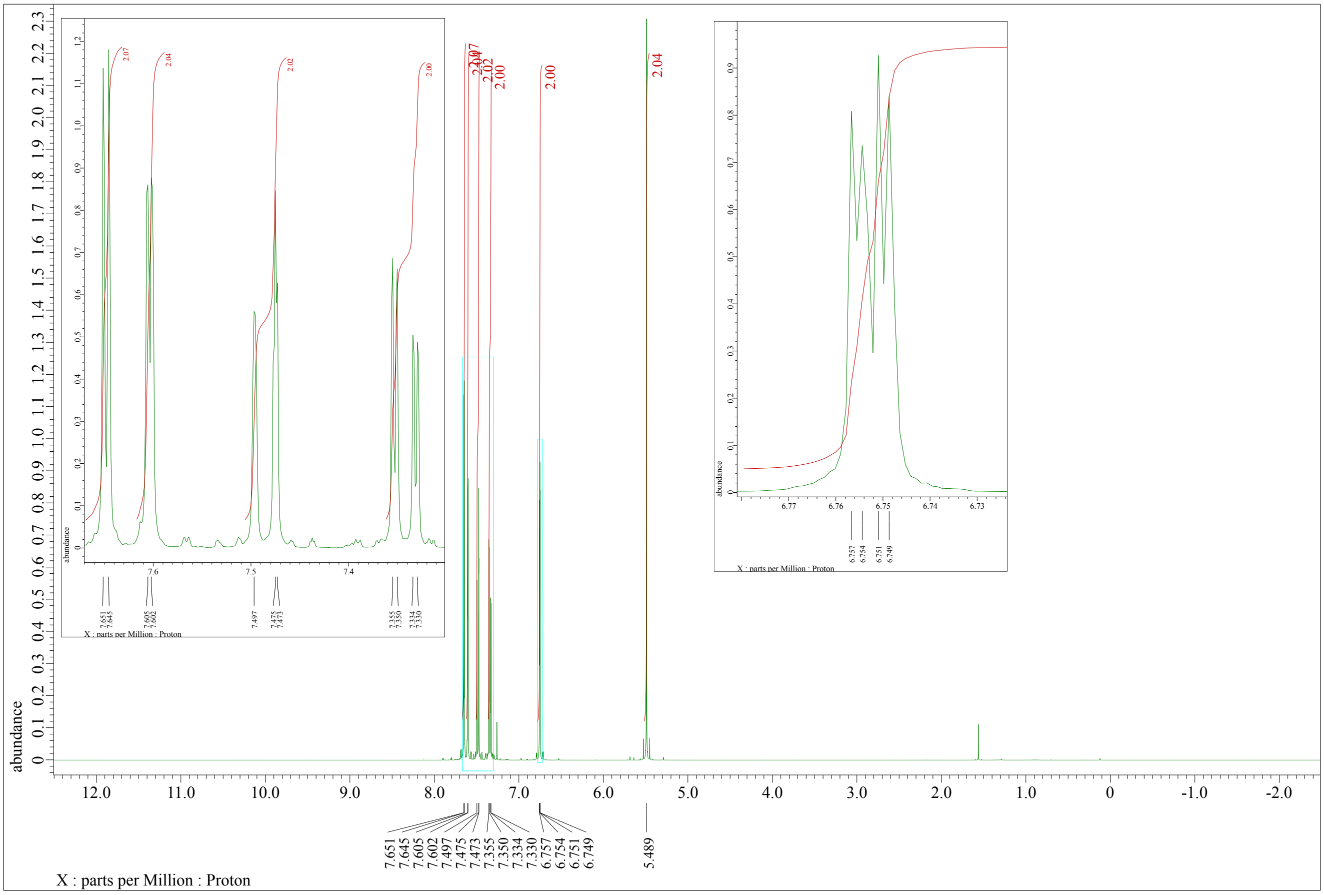

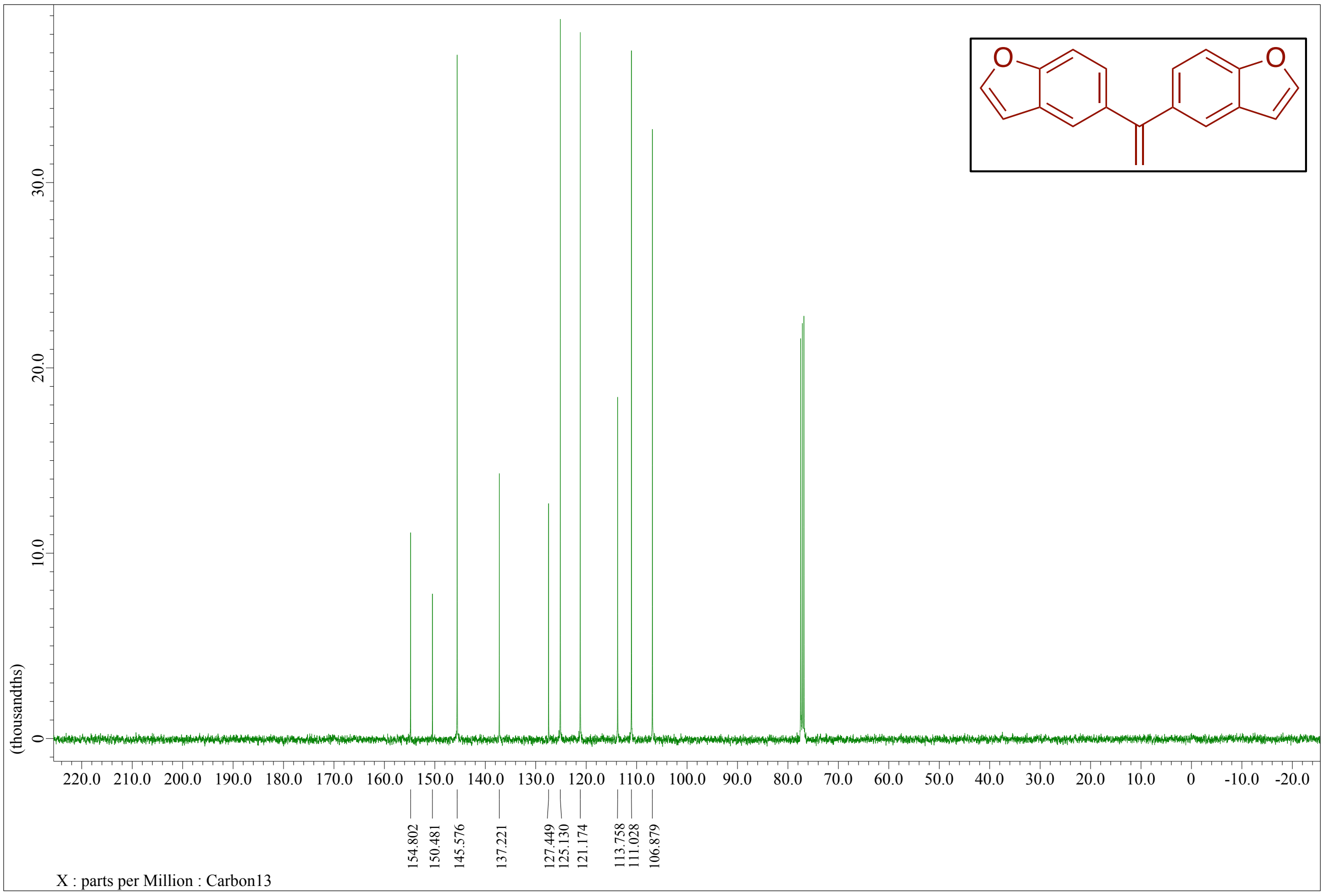

Figure S81. ^{1}H and ^{13}C NMR of **1l**.

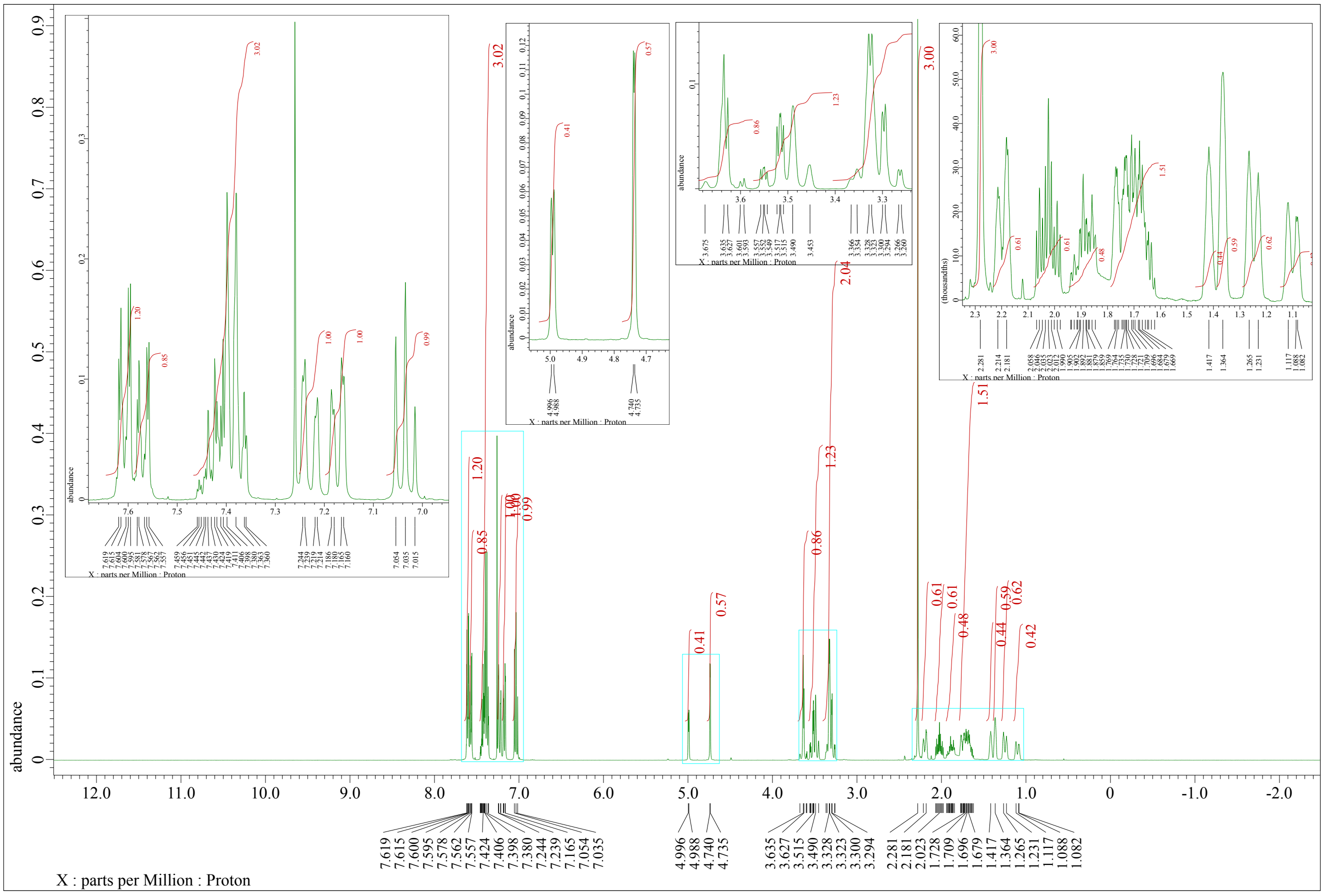

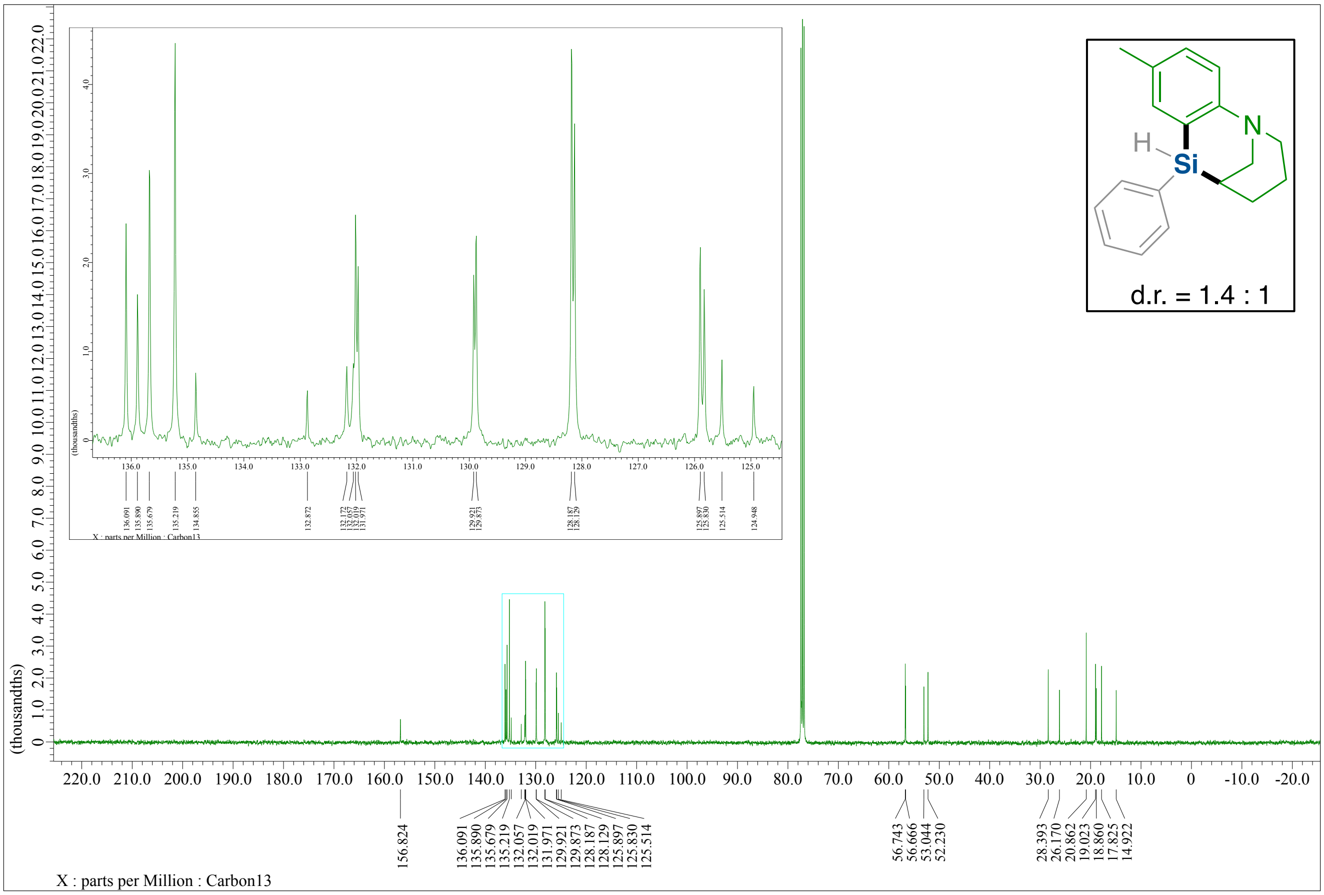

Figure S82. ^{1}H and ^{13}C NMR of **P**.

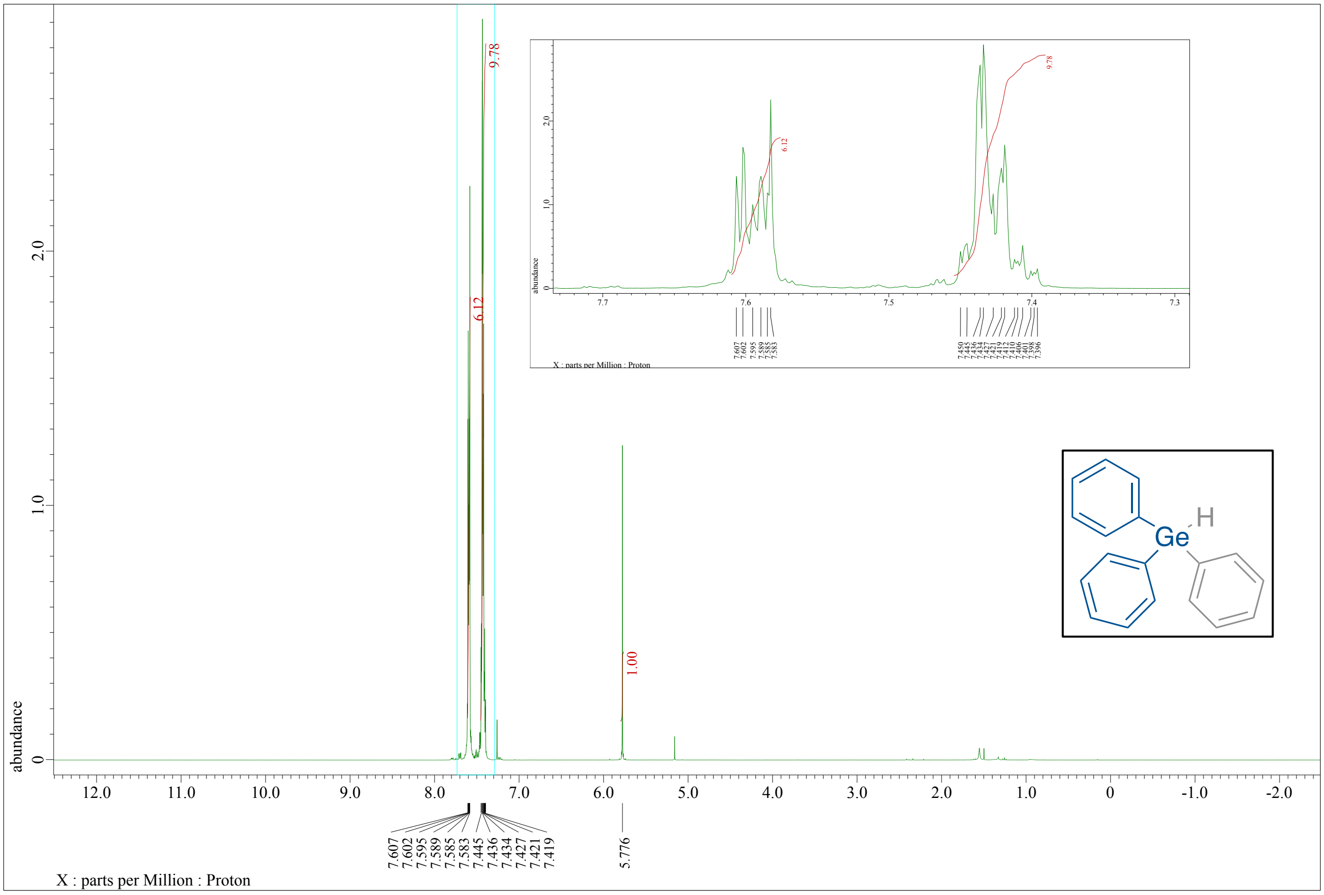

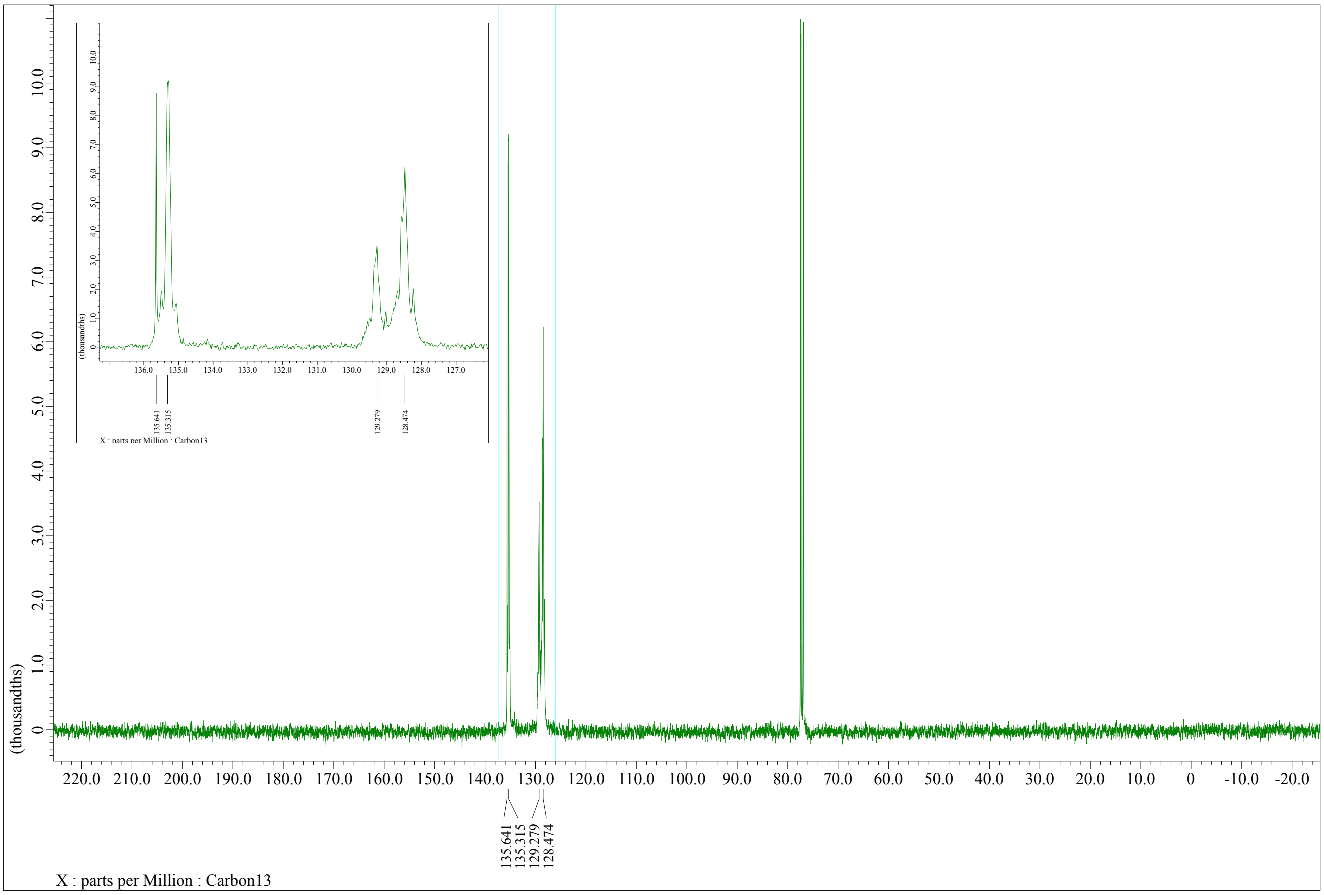

Figure S83. ^{1}H and ^{13}C NMR of **Q**.

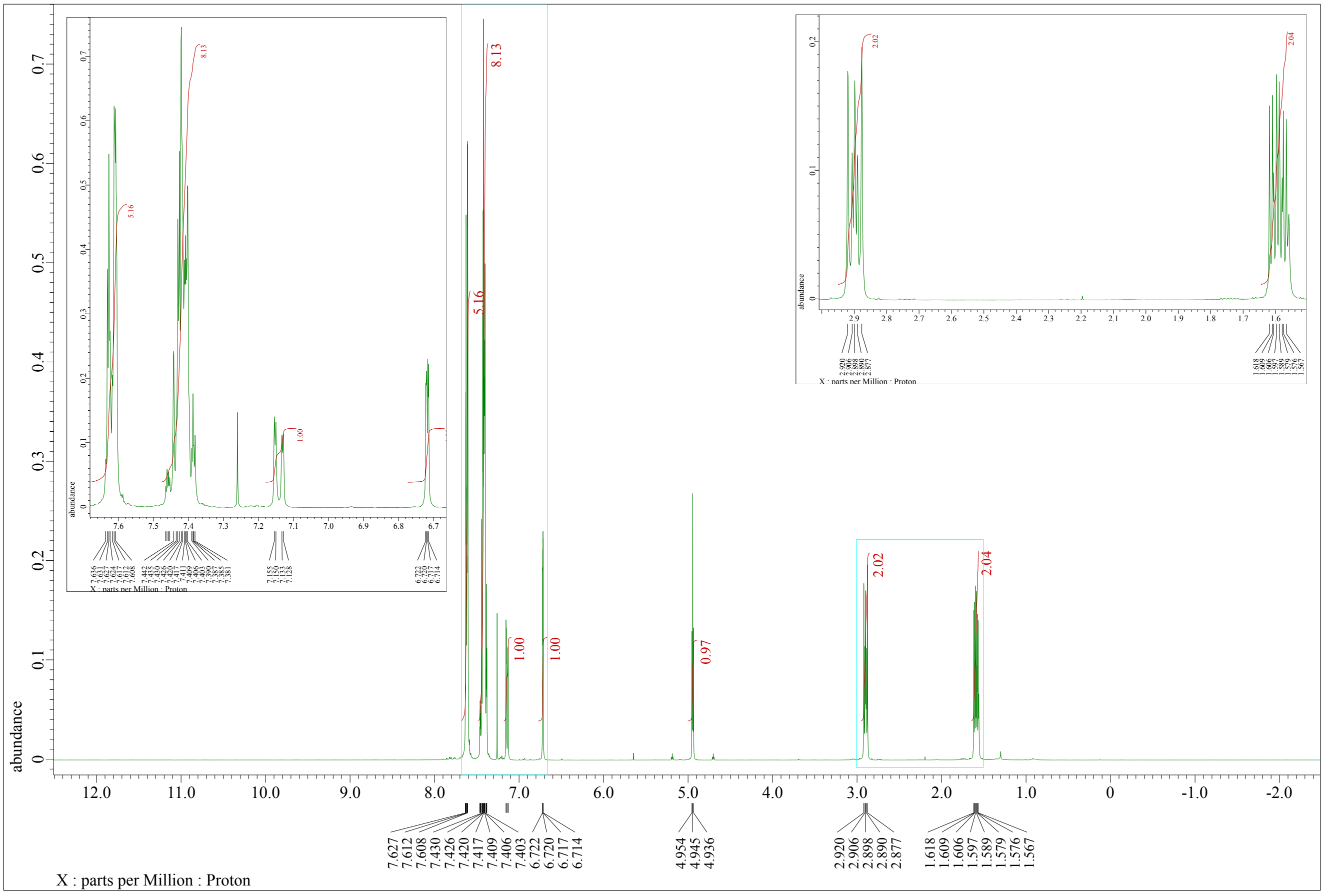

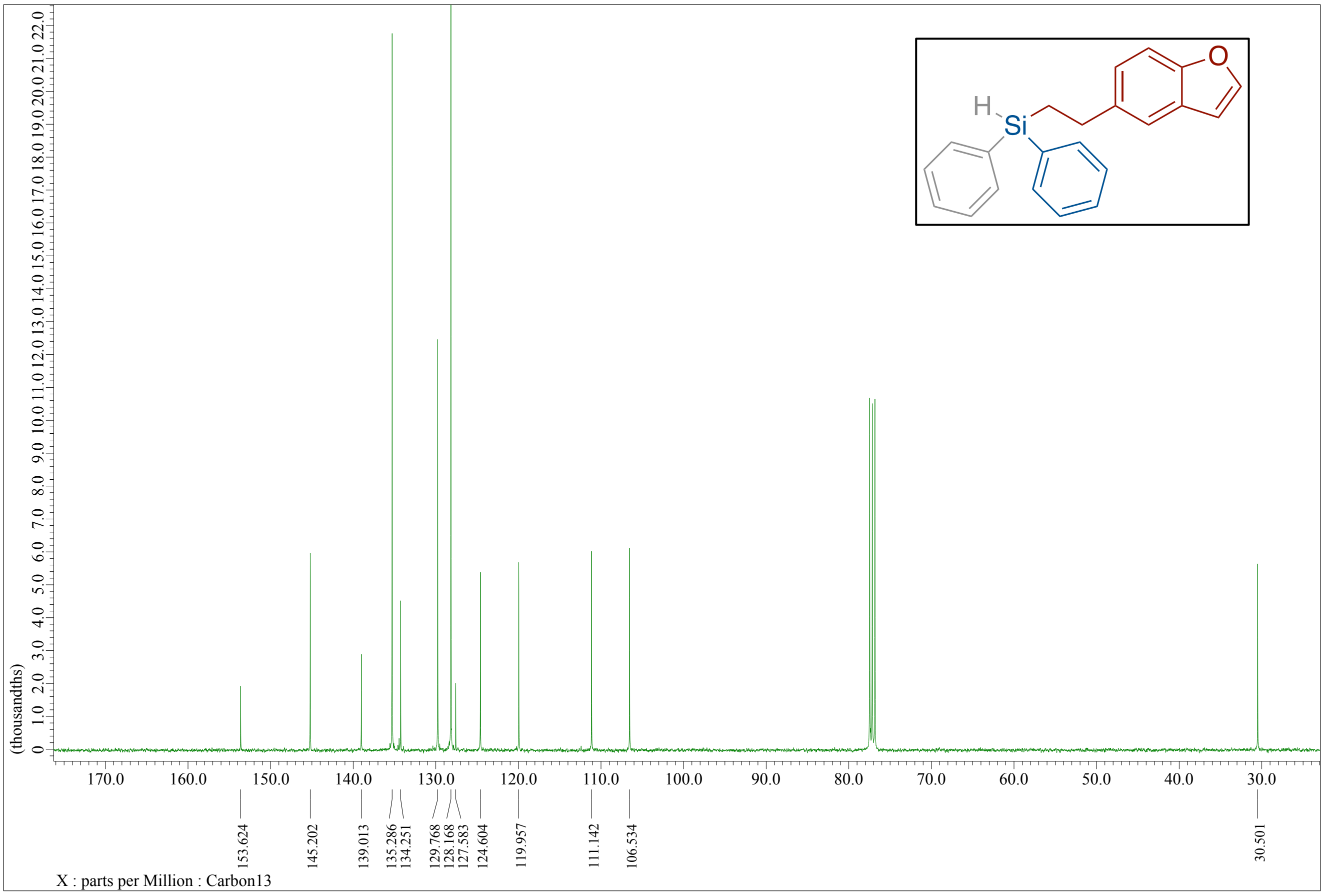

Figure S84. ^{1}H and ^{13}C NMR of **14**.

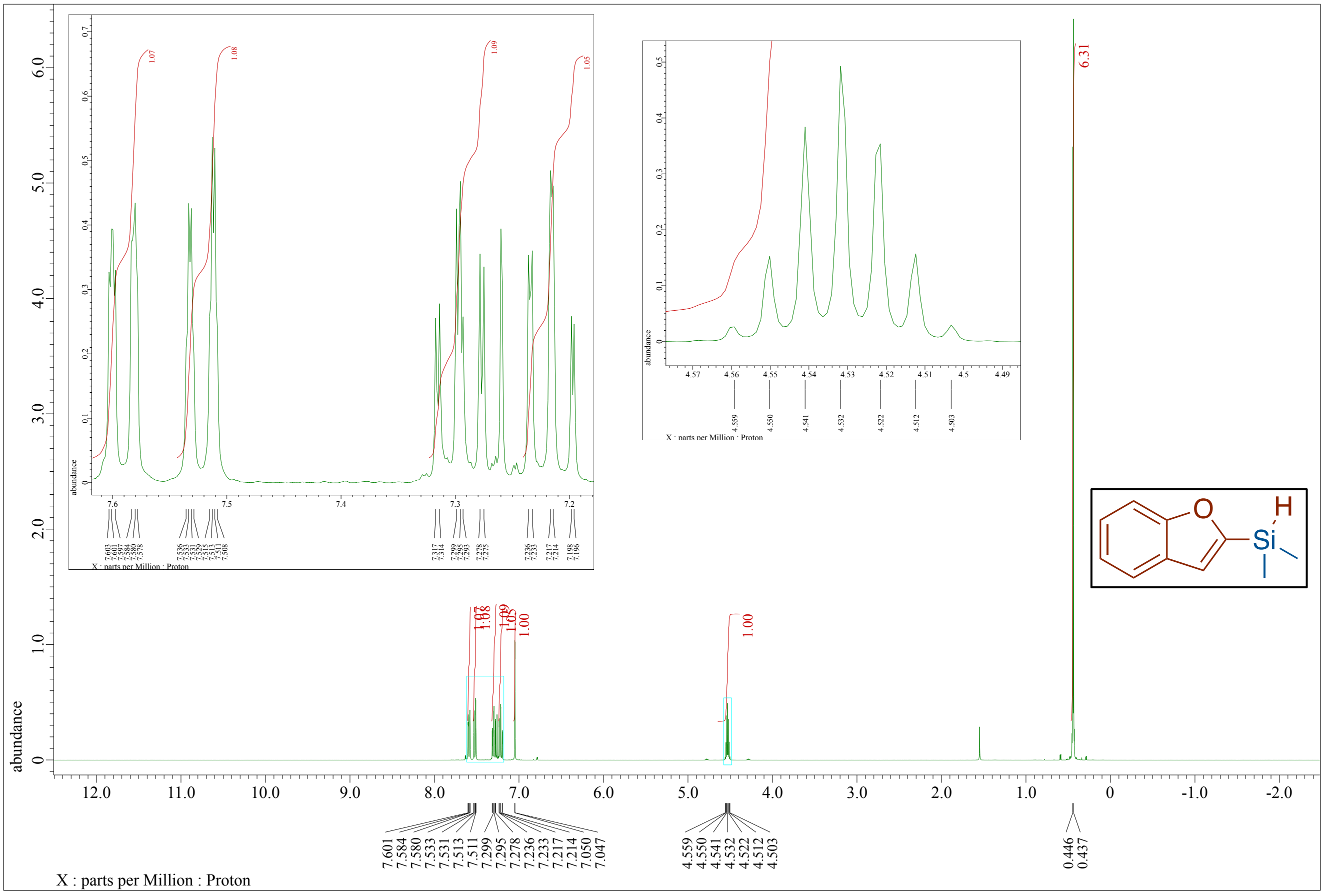

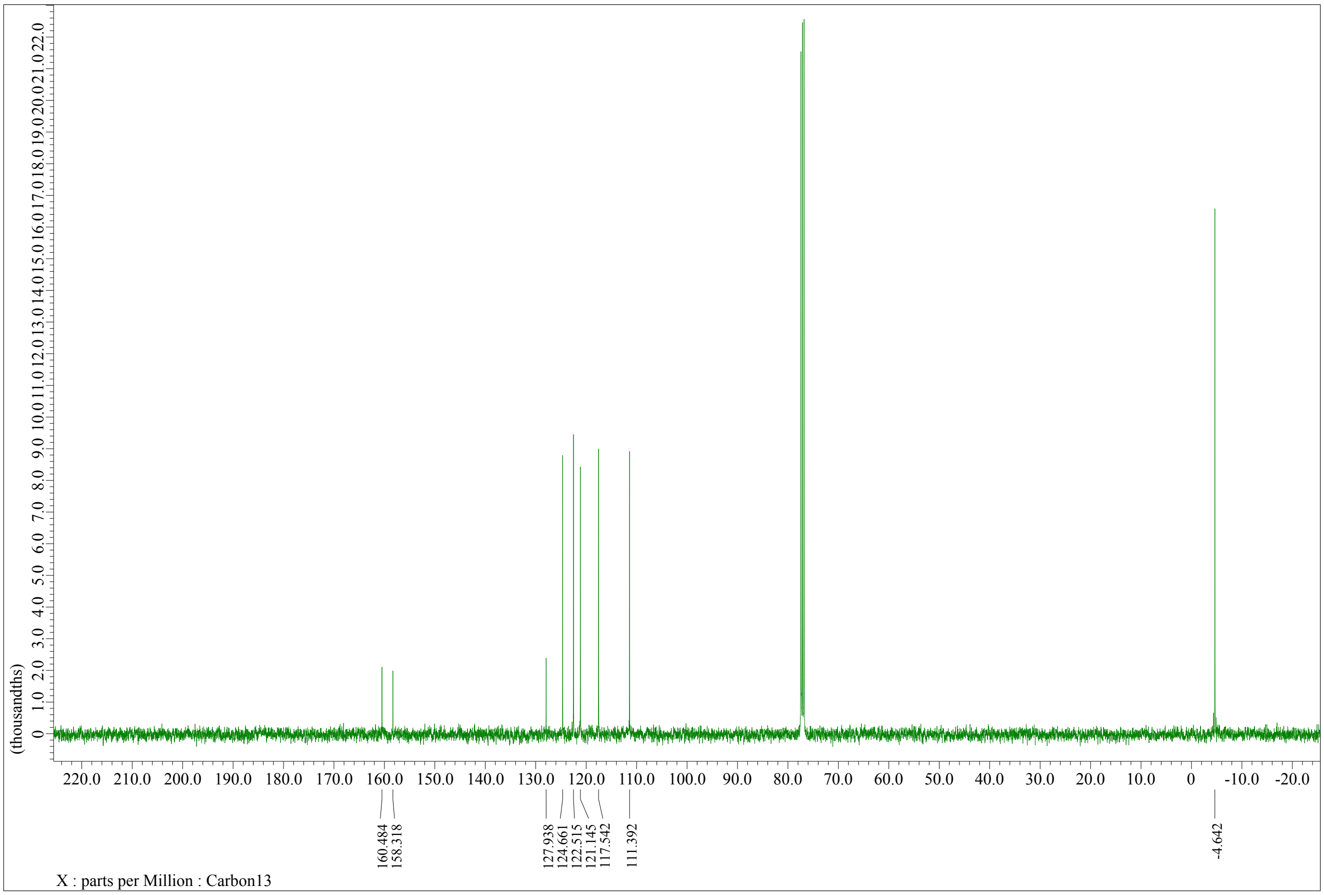

Figure S85. ^{1}H and ^{13}C NMR of **16**.

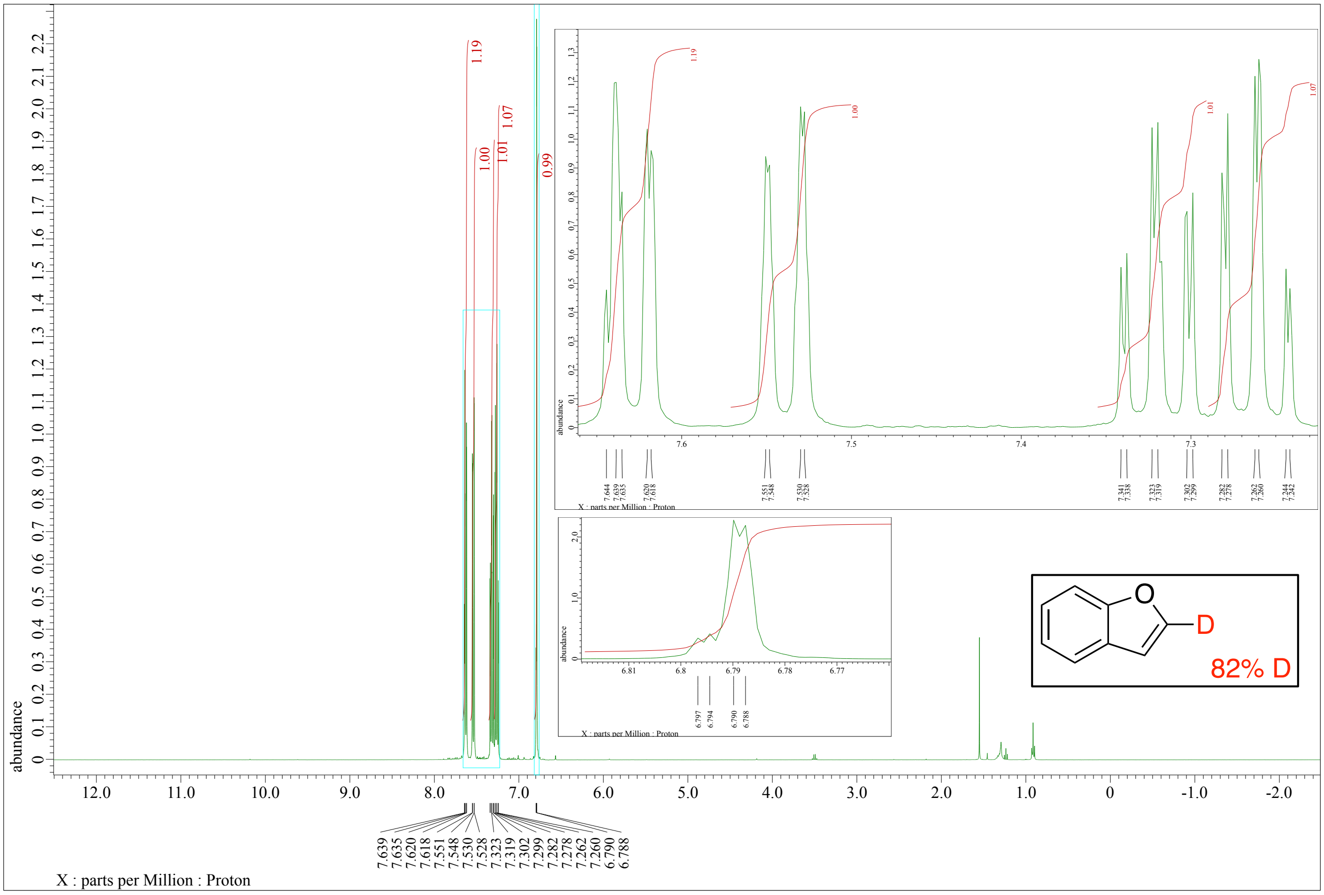

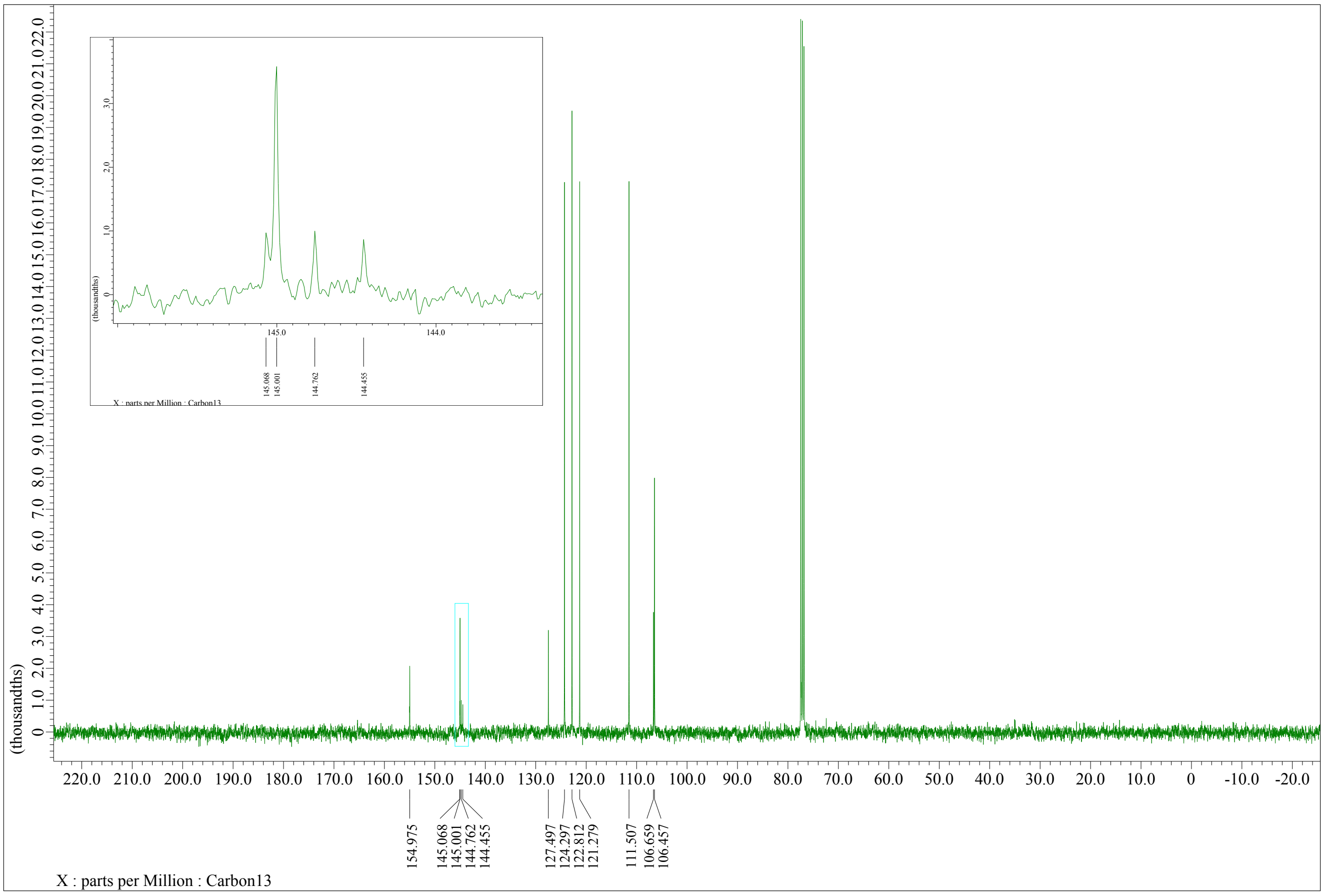

Figure S86. ^{1}H and ^{13}C NMR of **1a-*d***.

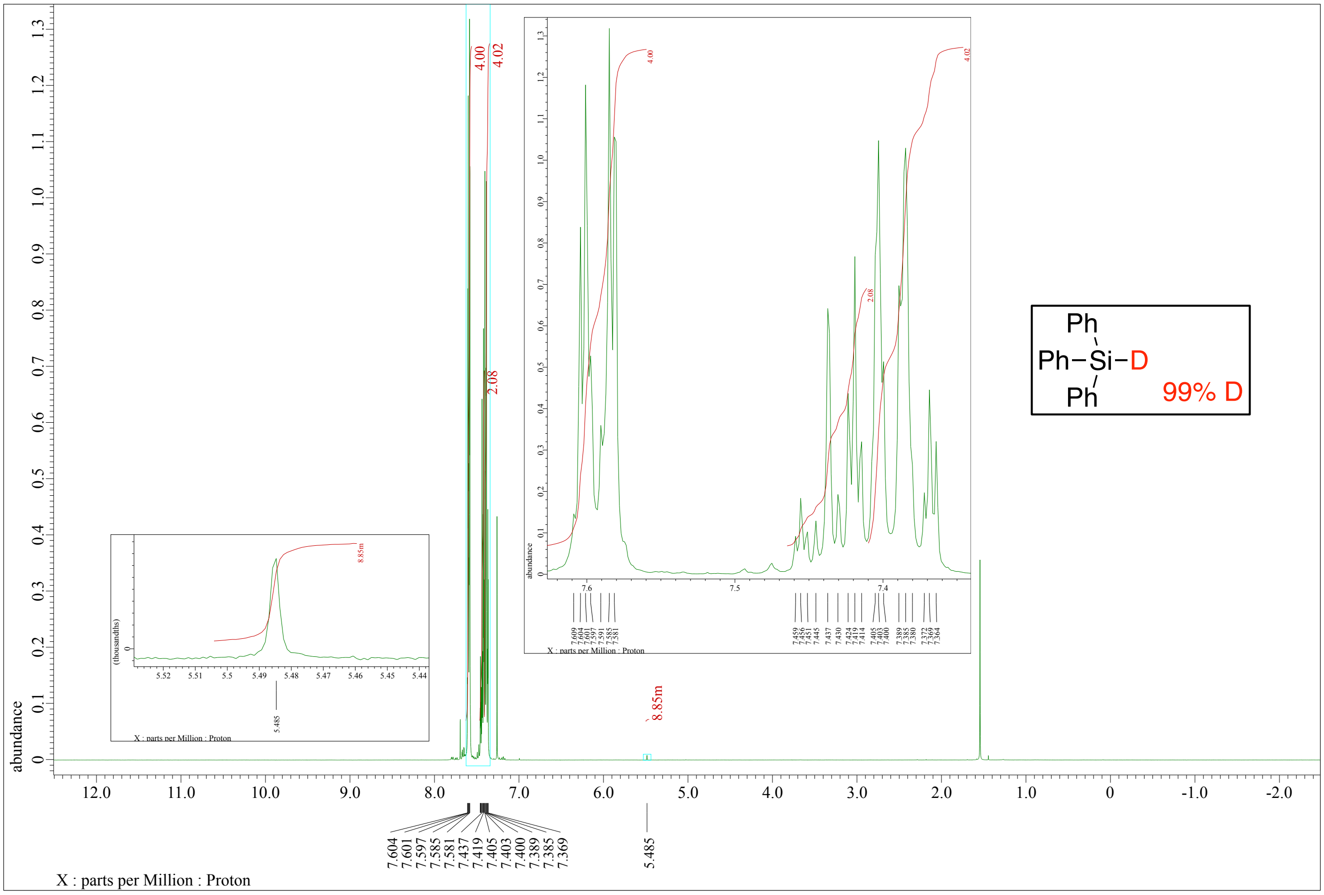

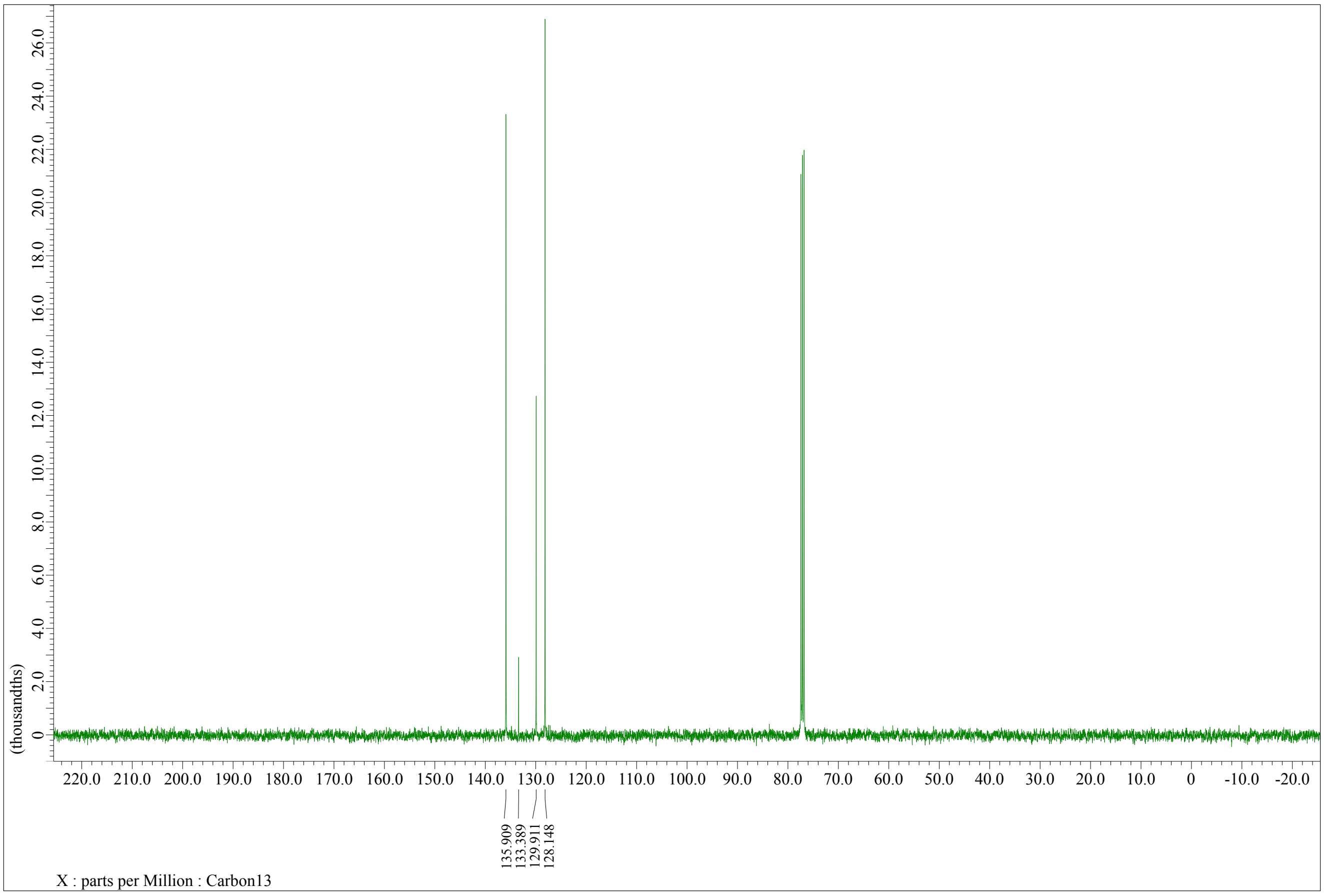

Figure S87. ^{1}H and ^{13}C NMR of ***J-d***.

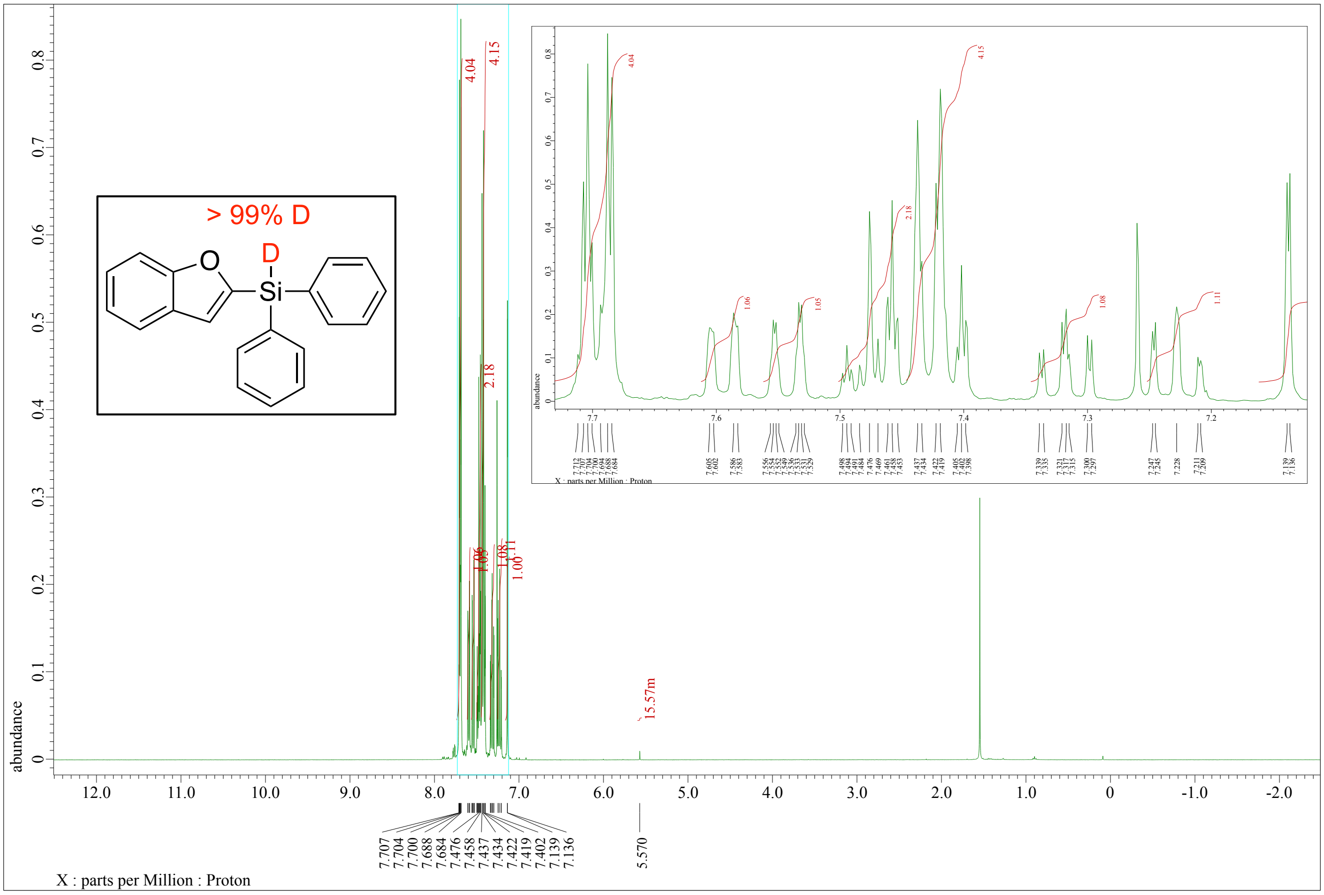

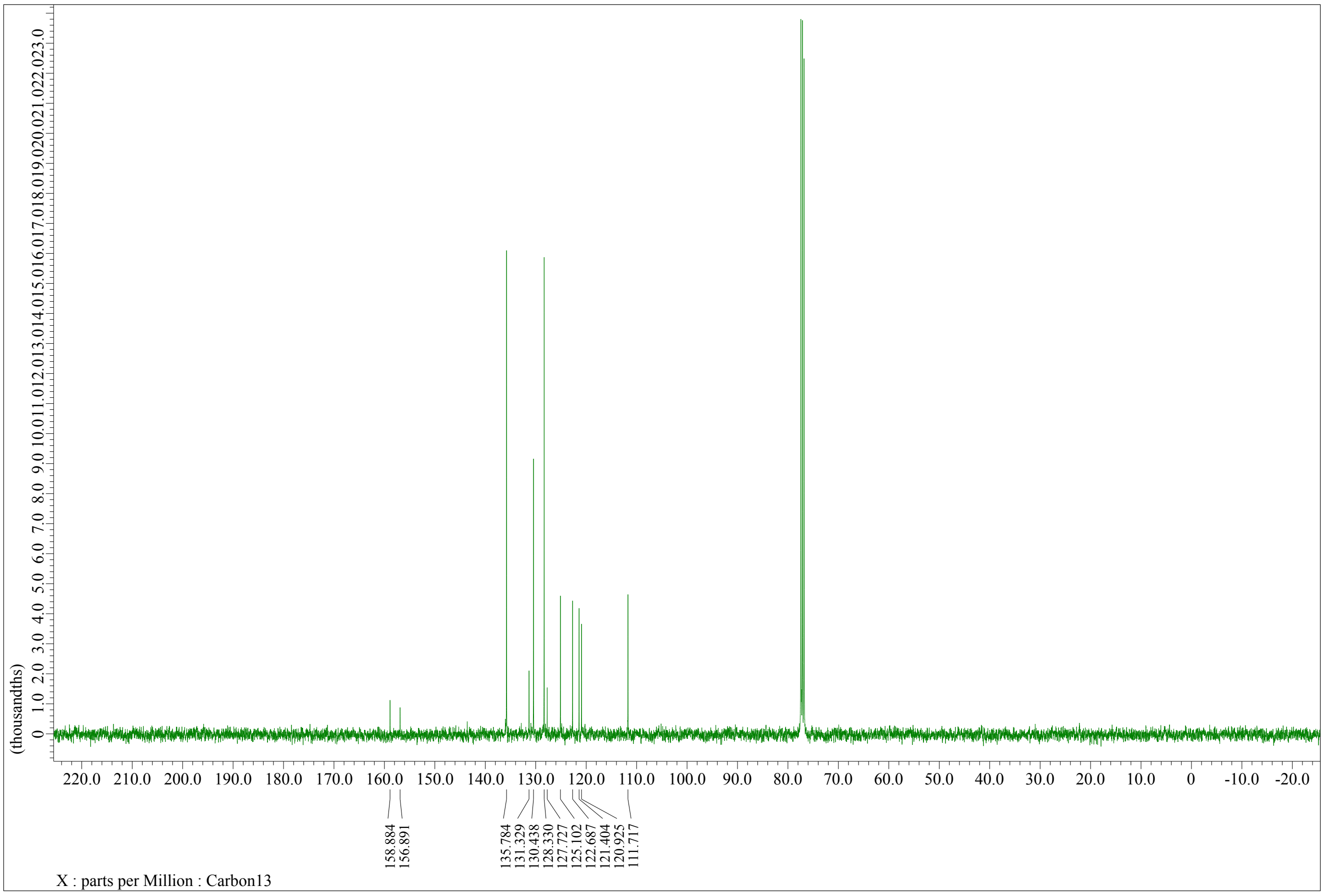

Figure S88. ^{1}H and ^{13}C NMR of **17aJ-*d***.
